# A triple farnesoid X receptor and peroxisome proliferator-activated receptor α/δ activator reverses hepatic fibrosis in diet-induced NASH in mice

**DOI:** 10.1038/s42004-020-00411-z

**Published:** 2020-11-13

**Authors:** Pascal Heitel, Giuseppe Faudone, Moritz Helmstädter, Jurema Schmidt, Astrid Kaiser, Amelie Tjaden, Martin Schröder, Susanne Müller, Simone Schierle, Julius Pollinger, Daniel Merk

**Affiliations:** 1 Institute of Pharmaceutical Chemistry, Goethe University Frankfurt, Max-von-Laue-Str. 9, 60438 Frankfurt, Germany; 2 Structural Genomics Consortium, BMLS, Goethe University Frankfurt, Max-von-Laue-Str. 15, 60438 Frankfurt, Germany

**Keywords:** Medicinal chemistry, Pharmacology, Target validation, Small molecules

## Abstract

Non-alcoholic steatohepatitis (NASH) - a hepatic manifestation of the metabolic syndrome - is a multifactorial disease with alarming global prevalence. It involves steatosis, inflammation and fibrosis in the liver, thus demanding multiple modes of action for robust therapeutic efficacy. Aiming to fuse complementary validated anti-NASH strategies in a single molecule, we have designed and systematically optimized a scaffold for triple activation of farnesoid X receptor (FXR), peroxisome proliferator-activated receptor (PPAR) α and PPARδ. Pilot profiling of the resulting triple modulator demonstrated target engagement in native cellular settings and in mice, rendering it a suitable tool to probe the triple modulator concept in vivo. In DIO NASH in mice, the triple agonist counteracted hepatic inflammation and reversed hepatic fibrosis highlighting the potential of designed polypharmacology in NASH.

## Abstract (web summary)

Non-alcoholic steatohepatitis demands multiple modes of action for robust therapeutic efficacy. Here the authors design and optimize a triple modulator of farnesoid X receptor and peroxisome proliferator-activated receptors α and δ that counteracts hepatic inflammation and reverses hepatic fibrosis in mice.

## Introduction

Non-alcoholic fatty liver disease (NAFLD) triggered by excessive body weight and a sedentary lifestyle is regarded as hepatic manifestation of the metabolic syndrome. The clinical picture of NAFLD comprises marked accumulation of fat in the liver, which is not related to extensive alcohol consumption and can progress to chronic hepatic inflammation termed non-alcoholic steatohepatitis (NASH). With an estimated global prevalence of 25% for NAFLD and a progression to NASH in 10–30% of NAFLD patients, the disease complex is a serious global health burden^1^. Yet, there is no satisfying pharmacological treatment.

**Fig. 1:**
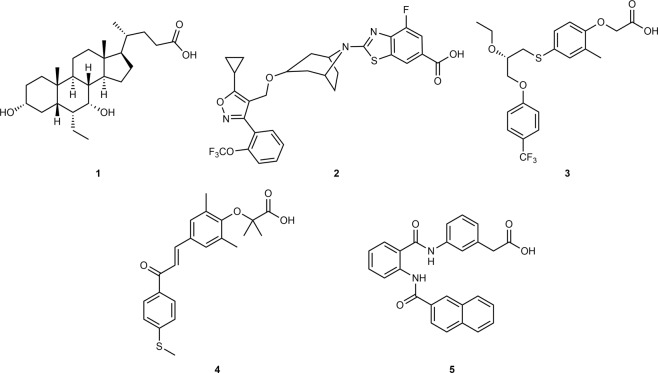
Literature FXR and PPAR agonists. Obeticholic acid (OCA, 6α-ethylchenodeoxycholic acid, **1**) and tropifexor (**2**) are FXR agonists that are currently undergoing clinical trials for NASH. Seladelpar (**3**), a selective PPARδ agonist, and the dual PPARα/δ agonist elafibranor (**4**) are additional investigational drugs for NASH. Phenylacetic acid **5**^20^ served as a lead structure for dual FXR/PPAR partial agonists.

The treatment pipeline for NASH therapy is currently dominated by modulators of the nuclear farnesoid X receptor (FXR, NR1H4) and the peroxisome proliferator-activated receptors (PPARs, NR1C1–3), which act as ligand-activated transcription factors. FXR is a bile acid sensor found in tissues with high bile acid exposure, such as the liver, intestine, and kidney^2,3^. It is a key metabolic regulator involved in bile acid, lipid, and glucose homeostasis^4^. Obeticholic acid (OCA, 6α-ethylchenodeoxycholic acid, **1**, Fig. 1) is a semisynthetic steroidal FXR agonist, which has validated FXR as a therapeutic target for NAFLD and NASH in clinical trials^5,6^. OCA (**1**) improved histological features of NASH and reduced markers of inflammation and fibrosis but its therapeutic efficacy was insufficient to resolve the hepatitis or reverse fibrosis^5^. Additionally, strong FXR agonism as exhibited by OCA causes repression of the enzyme cholesterol 7α-hydroxylase (CYP7A1), which catalyzes the rate-determining step in bile acid biosynthesis from cholesterol^4^. As a consequence, full FXR activation may trigger an undesirable disturbance of cholesterol homeostasis. Partial FXR agonists with reduced activation efficacy than the common FXR agonists OCA (**1**) or tropifexor (**2**) might avoid elevation of cholesterol levels while retaining therapeutic efficacy^7–10^.

PPARs are fatty acid and lipid-activated transcription factors^11^. While PPARγ (NR1C3) is associated with anabolic storage of lipids, PPARα (NR1C1) and PPARδ (NR1C2) promote catabolic lipid metabolism in brown adipose tissue, skeletal muscle, liver, intestine, heart, and kidney^11^. PPARα is the master regulator of hepatic β-oxidation and adapts lipogenesis and ketone body synthesis to the nutritional status^12^. Similarly, PPARδ governs fatty acid catabolism in the skeletal muscle with anti-atherogenic and insulin-sensitizing properties, thus qualifying the nuclear receptors as target for metabolic disorders^13,14^. The selective PPARδ agonist seladelpar (**3**) and the dual PPARα/δ ligand elafibranor (**4**) are currently studied in clinical trials for their ability to treat NASH^15,16^.

NASH is part of a cluster of pathologies referred to as metabolic syndrome and is associated with risk factors, such as obesity and type II diabetes. NASH manifestation and progression is multifactorial and involves hepatic steatosis, inflammation, and fibrosis as its hallmarks. This multifactorial nature of NASH may demand multimodal therapeutic intervention with multiple pharmacological modes of action to achieve sufficient therapeutic efficacy.

Elafibranor (**4**) and OCA (**1**) each represent promising clinical candidates to counteract NASH. Clinical trials have demonstrated their efficacy in reducing fibrosis and inflammation markers and improving lipid and glucose profiles^5,16^. Nevertheless, complete resolution of NASH or desired decreases in NAFLD activity scores as primary outcomes were mostly not achieved^5,16,17^. According to the Food and Drug Administration (FDA), OCA (**1**)’s uncertain benefit does not outweigh potential risks including pruritus and elevated cholesterol levels, what recently prompted the FDA to reject the approval for fibrosis due to NASH^18^. The lack of therapeutic efficiency may be due to the multifactorial nature of the disease complex. Thus we have proposed that dual FXR/PPAR modulation might be more efficacious in NASH treatment, as PPAR- and FXR-mediated effects result from different molecular mechanisms, occur in different tissues, and could support each other in promoting therapeutic effects.

FXR, as essential liver-protective transcription factor, mainly affects liver health directly and exhibits anti-steatotic, anti-fibrotic, and anti-inflammatory activity. PPARα supports these effects by improving hepatic lipid metabolism. Notably, PPARα expression is enhanced by FXR activation illustrating the integration of both nuclear receptors’ signaling networks. PPARδ contributes peripheral effects by improving metabolic balance, e.g., through promoting utilization of lipids for energy supply in the skeletal muscles. Simultaneous activation of all three factors could thus achieve superior, potentially synergistic efficacy in NASH therapy. Supporting our hypothesis, a recent study^19^ has demonstrated that combined OCA (**1**) and elafibranor (**4**) treatment was superior to monotherapy in a diet-induced mouse model of NASH. Therefore, we aimed to develop a triple FXR/PPARα/δ modulator with moderate activation efficacy to unite the anti-NASH activities of the three transcription factors while avoiding potential mechanism-based side effects.

In previous studies, we have identified phenylacetic acid **5** as a dual FXR/PPAR modulator with micromolar activity on all target receptors^20^. **5** demonstrated partial agonism on FXR and PPARs with slight preference for PPARα and PPARδ over PPARγ. Apart from FXR and PPARs, **5** was selective over related nuclear receptors qualifying the compound as suitable template for the design of FXR/PPAR activators. Using **5** as a lead, we systematically probed the structure–activity relationship (SAR) of the chemotype on FXR and PPARs with derivatives **6**–**41**. We succeeded in optimizing **5** to the balanced triple FXR/PPARα/δ modulator **41** endowed with nanomolar potency and selectivity over PPARγ as well as several related transcription factors. In a pilot animal study, **41** demonstrated target engagement in vivo. Encouraged by this profile, we studied the efficacy of **41** to counter NASH in diet-induced obese (DIO) mice and observed strong anti-fibrotic efficacy highlighting the potential of designed polypharmacology in NASH treatment.

## Results and discussion

### Chemistry

**Fig. 2:**
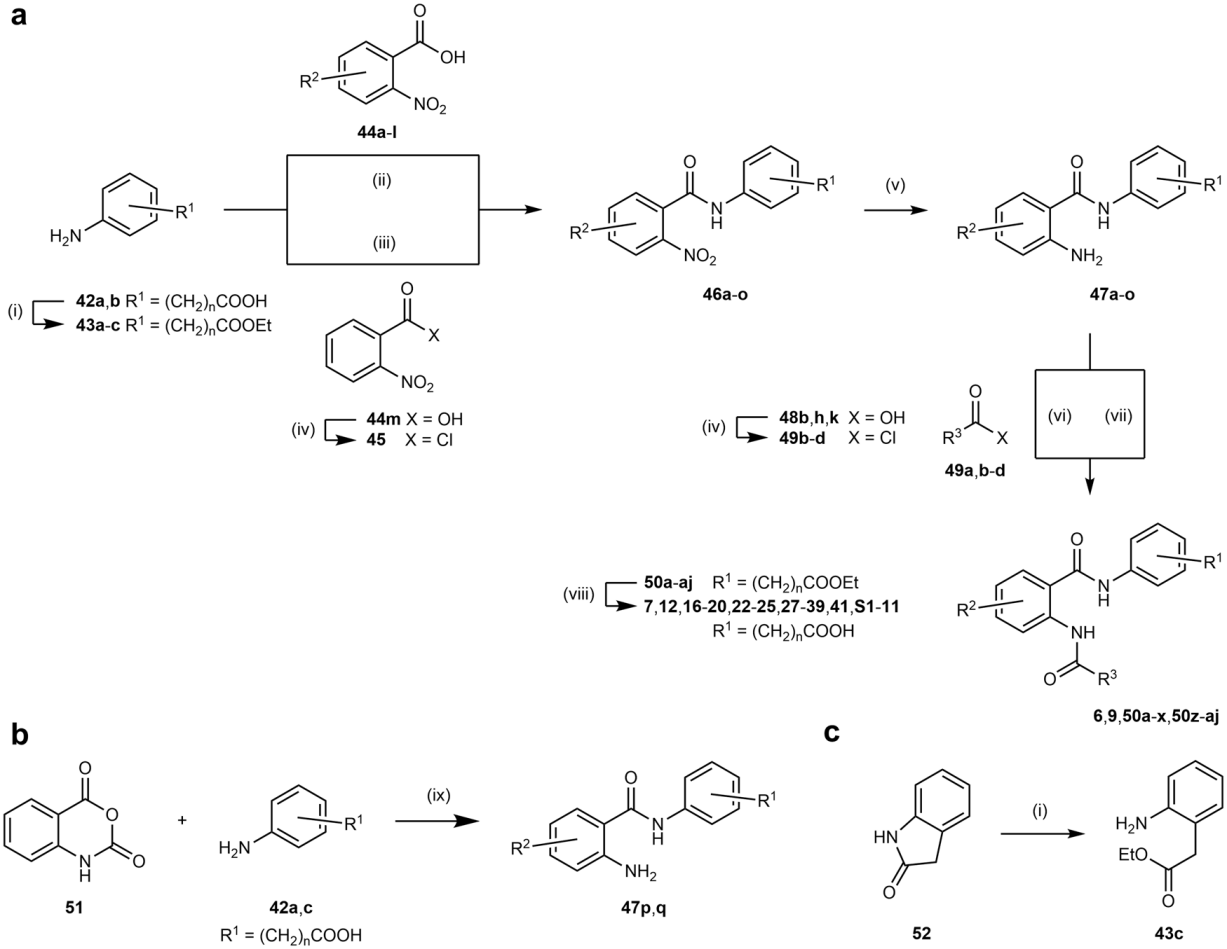
Synthetic pathways. Synthesis of **6**, **7**, **9**, **12**, **16**–**20**, **22**–**25**, **27**–**39**, **41**, **S1**–**11** (**a**), precursors **47p**, **q** (**b**), and precursor **43c** (**c**). Reagents and conditions: (i) EtOH, H_2_SO_4_, 90 °C, 39–94%. (ii) EDC·HCl, DMAP, CHCl_3_, 75 °C, 17–93%. (iii) pyridine, THF, 75 °C, 77–99%. (iv) SOCl_2_, 80 °C (v) H_2_, Pd/C, EtOAc, rt, or Fe, HOAc, EtOAc, 50 °C or SnCl_2_, 10% HCl, EtOAc, 50 °C, 33–98%. (vi) pyridine, THF, 75 °C, 35–86%. (vii) R^3^-COOH (**48a**,**c**–**g**,**i**,**j**,**l**–**u**), EDC·HCl, DMAP, CHCl_3_, 75 °C, 20–99%. (viii) (I) LiOH, THF, H_2_O, rt–50 °C (II) 5% HCl, 20–99%. (ix) EtOH, 90 °C, 74–98%.

Synthesis of compounds **5**, **8**, **10**, and **11** has been reported previously^20^. Compounds **6**, **7**, **9**, and **12**–**41** were prepared in five-step procedures (Fig. 2a) starting from aminophenylalkylcarboxylates **42a** or **42b**, which were protected via esterification with ethanol. Esters **43a**–**c** were subsequently reacted with either 2-nitrobenzoic acids **44a**–**l** or 2-nitrobenzoyl chloride (**45**) to anthranilamides **46a**–**o**. Reduction of the nitro group afforded amines **47a**–**o**, which underwent a second amide bond formation with carboxylic acids **48a**,**c**–**g**,**i**,**j**,**l**–**u** or acid chlorides **49a**,**b**,**d** to **6**, **9**, **50a**–**x**, **50z**–**aj**. The final FXR/PPAR modulator candidates were obtained by alkaline ester hydrolysis of **50a**–**aj**.

In an alternative synthetic route, anthranilamides **47p**,**q** were prepared directly by reaction of isatoic anhydride (**51**) with anilines **42a**,**c** (Fig. 2b). Ring opening and esterification of lactam **52** in a single reaction afforded ethyl 2-aminophenylacetate (**43c**, Fig. 2c).

**Fig. 3:**
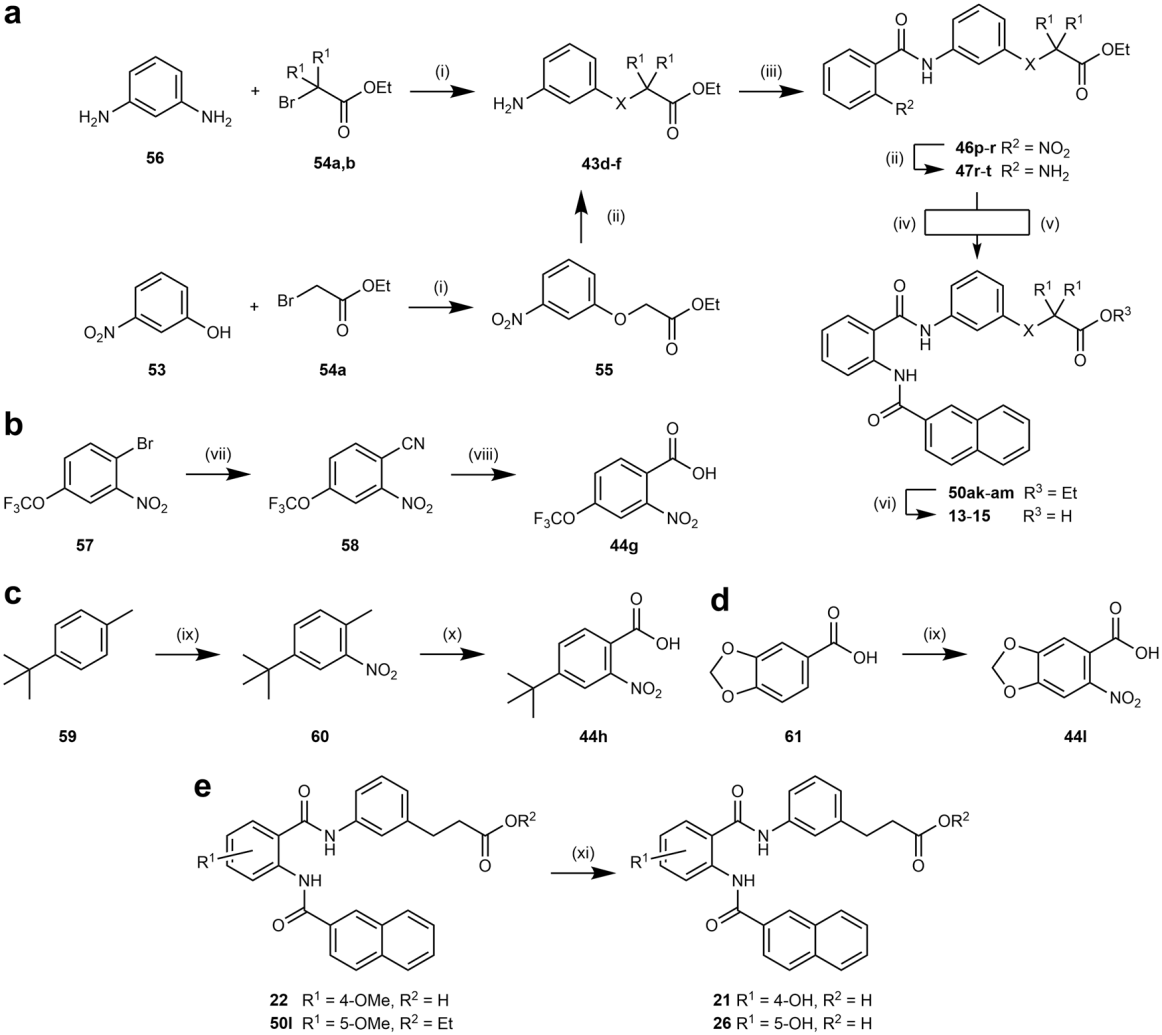
Synthetic pathways. Synthesis of **13**–**15** (**a**), nitrobenzoic acid precursors **44g** (**b**), **44h** (**c**) and **44l** (**d**), and **21** and **26** (**e**). Reagents and conditions: (i) K_2_CO_3_, DMF, 80 °C, 50–98%. (ii) H_2_, Pd/C, EtOAc, rt, 64–86%. (iii) 2-nitrobenzoic acid (**44** **m**), EDC·HCl, DMAP, CHCl_3_, 75 °C, 54–93%. (iv) 2-naphthoic acid (**48a**), EDC·HCl, DMAP, CHCl_3_, 75 °C, 65%. (v) 2-naphthoyl chloride (**49a**), pyridine, THF, 75 °C, 42–45%. (vi) (I) LiOH, THF, H_2_O, rt – 50 °C (II) 5% HCl, 98–99%. (vii) (I) CuCN, DMF, 150 °C (II) toluene, 130 °C, 92%. (viii) 55% H_2_SO_4_ (aq.), 120 °C, 45%. (ix) HNO_3_, HOAc, Ac_2_O, 0 °C, 52–76%. (x) KMnO_4_, pyridine, H_2_O, 110 °C, 18%. (xi) BBr_3_, CH_2_Cl_2_, 0 °C–rt, 22–65%. X = O, NH; R_1_ = H, CH_3_.

Analogs **13**–**15** comprising heteroatoms in the acidic side chain were prepared according to Fig. 3a. 3-Nitrophenol (**53**) was reacted with ethyl bromoacetate (**54a**) to ether **55** and subsequent Pd-catalyzed hydrogenation afforded aniline **43d**. Glycine derivatives **43e**,**f** were obtained from reaction of *m*-phenylenediamine (**56**) with bromides **54a**,**b**. The first amide bond resulted from conversion of 2-nitrobenzoic acid (**44** **m**) with anilines **43d**-**f**. After hydrogenation to the corresponding anthranilamides **47r**–**t**, the second amide bond was introduced using either 2-naphthoic acid (**48a**) and EDC or 2-naphthoyl chloride (**49a**). Esters **50ak**–**am** were then hydrolyzed under alkaline conditions to target compounds **13**–**15**.

2-Nitrobenzoic acids **44g**,**h**,**l** were not commercially available and thus prepared according to Fig. 3b–d. 1-Bromo-2-nitro-4-trifluoromethoxybenzene (**57**) reacted to nitrile **58** in a Rosenmund–von Braun reaction and hydrolysis of **58** afforded benzoic acid **44g**. *tert*-Butyl derivative **44h** was prepared by nitration of 4-*tert*-butyltoluene (**59**) followed by oxidation with KMnO_4_. 6-Nitrobenzo[1,3-*d*]dioxole-5-carboxylic acid (**44l**) was synthesized by nitration of **61**.

Phenols **21** and **26** were obtained by demethylation of methyl ethers **22** and **50l** with boron tribromide (Fig. 3e).

**Fig. 4:**
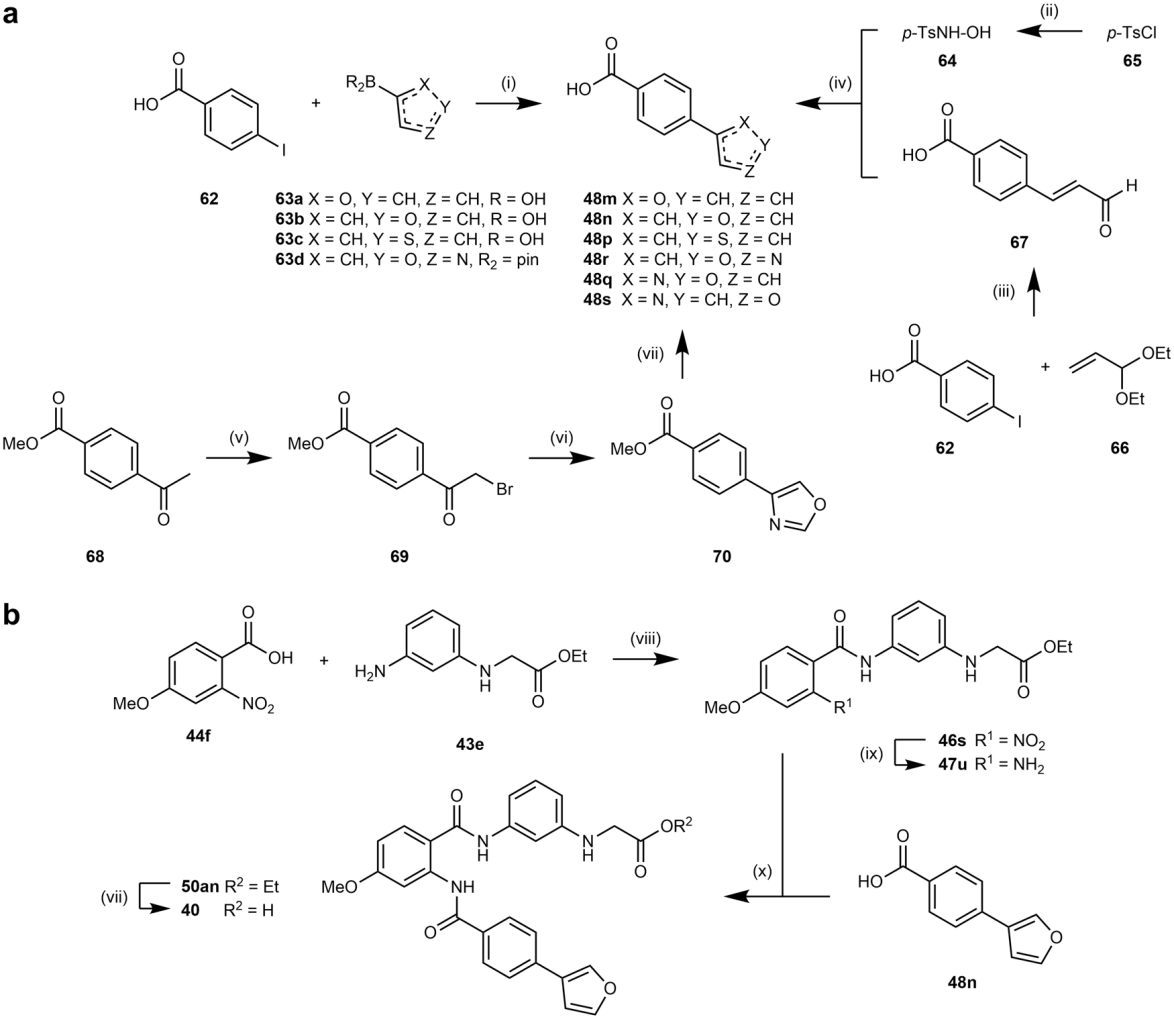
Synthetic pathways. Synthesis of 4-heteroarylbenzoic acid precursors **48m,n,p**–**s** (**a**) and compound **40** (**b**). Reagents and conditions: (i) Pd(PPh_3_)_4_, Na_2_CO_3_, dioxane, H_2_O, 90 °C, 81–90%. (ii) NH_2_OH·HCl, K_2_CO_3_, THF, MeOH, H_2_O, 0 °C–rt, 42%. (iii) Pd(OAc)_2_, K_2_CO_3_, KCl, Bu_4_NOAc, DMF, 90 °C, µw, 24%. (iv) K_2_CO_3_, MeOH, H_2_O, 60 °C, 55%. (v) Br_2_, HOAc, rt, 90%. (vi) ammonium formate, formic acid, DMF, rt, 27%. (vii) LiOH, THF, H_2_O, 40 °C, 36–99%. (viii) EDC·HCl, DMAP, CHCl_3_, 75 °C, 78%. (ix) H_2_, Pd/C, EtOAc, rt, 83%. (x) EDC·HCl, DMAP, CHCl_3_, 75 °C, 89%.

Suzuki reaction of 4-iodobenzoic acid (**62**) with boronic acids **63a**–**c** or boronic acid pinacol ester **63d** afforded 4-heteroarylbenzoic acids **48m**,**n**,**p**,**r** (Fig. 4a), which were further reacted according to Fig. 2a.

4-(Isoxazol-3-yl)benzoic acid (**48q**) and 4-(oxazol-4-yl)benzoic acid (**48s**) were prepared according to Fig. 4a. *N*-Tosylhydroxylamine (**64**) was generated from tosyl chloride (**65**) and hydroxylamine. Heck coupling of 4-iodobenzoic acid (**62**) and acrolein diethyl acetal (**66**) afforded cinnamon aldehyde **67**. **67** then cyclized with **64** to 4-(isoxazol-3-yl)benzoic acid (**48q**). Methyl 4-acetylbenzoate (**68**) was brominated to **69** and subsequently reacted with ammonium formate to oxazole **70**. Saponification yielded 4-(oxazol-4-yl)benzoic acid (**48s**).

Analog **40** was synthesized in a four-step procedure (Fig. 4b) from benzoic acid **44f** and amine **43e**, which were reacted to amide **46s**. Then the nitro group was reduced to amine **47u** and subsequent amide synthesis with **48n** afforded ethyl ester **50an**. **40** was obtained from alkaline hydrolysis of ester **50an**.

### Biological evaluation

Compounds **5**–**41** were characterized for their activity on FXR and PPARs in cellular luciferase-based reporter gene assays with transient transfection. Activity on PPARs was determined in hybrid reporter gene assays in HEK293T cells using chimeric receptors composed of the respective human PPAR subtype ligand-binding domain (LBD) and the DNA-binding domain of the yeast protein Gal4. A Gal4 inducible firefly luciferase construct served as reporter gene. FXR activation was determined in a full-length reporter gene assay in HeLa cells in which the firefly luciferase reporter was under the control of the FXR response element (RE) from the promoter region of human bile salt export protein (BSEP). This RE requires the entire human FXR:retinoid X receptor (RXR) heterodimer, and thus FXR and RXRα were overexpressed for the assay system. In both assays, a constitutively expressed renilla luciferase was co-transfected to serve for normalization of transfection efficiency and to observe test compound toxicity. Test compound activities were normalized to the reference agonists GW4064 (3 μM, FXR), GW7647 (PPARα, 1 µM), L165,041 (PPARδ, 1 µM), or rosiglitazone (PPARγ, 1 µM) to obtain relative activation efficacy.

### Structural optimization

Starting from **5**, we commenced our SAR analysis in the acidic head group region (Table 1), where the position and length of the acidic chain were systematically modified (**5**–**12**). The length and position of the acidic chain markedly affected potency on FXR and PPARs. PPARs generally favored a long acidic side chain in 3-position (**9**) while 4-substitution (**10**–**12**) was not tolerated by any PPAR subtype and 2-substitution (**7**) disrupted activity on PPARδ. On FXR, all 2-, 3- and 4-substitution patterns were tolerated with preference for 2- and 3-positions regarding activation efficacy. The 3-phenylpropanoate motif of **9** revealed the most balanced activity on PPARs and FXR with weak preference over PPARγ. In an attempt to reduce the lipophilicity of this residue and eventually promote aqueous solubility, heteroatoms were incorporated in the chain. Ether analog **13** displayed diminished activity on all PPARs and FXR and thus was not favored. Amine **14** comprised a more distinguished activity profile. On PPARα, **14** turned out inactive and potency on PPARγ was reduced as well, while activation efficacy on PPARδ and potency on FXR were enhanced resulting in FXR/PPARδ preference. Additional introduction of two methyl groups in α-position of the carboxylate (**15**) was strongly favored by FXR and reinstated activity on PPARα, but this modification also promoted activation efficacy on PPARγ resulting in poor selectivity among PPARs.

**Table 1:** Structure–activity relationship investigation of triple FXR/PPARα/δ activators 6–41.


				EC_50_ [μM] (max. rel. act.)			
ID	R^1^	R^2^	R^3^	PPARα	PPARδ	PPARγ	FXR
**6**	2-COOH	—	Naphth-2-yl	tox.	tox.	tox.	7 ± 1 (44 ± 3%)
**7**	2-CH_2_COOH	—	Naphth-2-yl	1.4 ± 0.1 (33 ± 1%)	> 10	1.7 ± 0.1 (17 ± 1%)	9.2 ± 0.2 (31 ± 1%)
**8**	3-COOH	—	Naphth-2-yl	0.15 ± 0.01 (11 ± 1%)	1.3 ± 0.1 (14 ± 1%)	1.6 ± 0.1 (17 ± 1%)	1.5 ± 0.2 (37 ± 1%)
**5**	3-CH_2_COOH	—	Naphth-2-yl	5 ± 1 (37 ± 3%)	4.9 ± 0.9 (34 ± 2%)	16 ± 1 (14 ± 1%)	2.5 ± 0.2 (21 ± 1%)
**9**	3-CH_2_CH_2_COOH	—	Naphth-2-yl	1.0 ± 0.2 (31 ± 2%)	0.65 ± 0.03 (28 ± 1%)	2.6 ± 0.3 (19 ± 2%)	2.6 ± 0.3 (20 ± 1%)
**10**	4-COOH	—	Naphth-2-yl	>30	>30	4.4 ± 0.9 (18 ± 2%)	1.0 ± 0.2 (23 ± 1%)
**11**	4-CH_2_COOH	—	Naphth-2-yl	>30	>30	>30	3.1 ± 0.3 (9.8 ± 0.4%)
**12**	4-CH_2_CH_2_COOH	—	Naphth-2-yl	>30	>30	>30	2.1 ± 0.1 (21 ± 1%)
**13**	3-OCH_2_COOH	—	Naphth-2-yl	10 ± 1 (35 ± 2%)	10 ± 1 (43 ± 4%)	19 ± 1 (11 ± 1%)	5 ± 1 (19 ± 1%)
**14**	3-NHCH_2_COOH	—	Naphth-2-yl	>30	3.5 ± 0.2 (83 ± 3%)	5.7 ± 0.1 (7.7 ± 0.1%)	1.3 ± 0.1 (31 ± 1%)
**15**	3-NHC(CH_3_)_2_COOH	—	Naphth-2-yl	1.7 ± 0.1 (71 ± 3%)	3.6 ± 0.2 (42 ± 1%)	5.1 ± 0.9 (18 ± 1%)	0.45 ± 0.01 (10 ± 1%)
**16**	3-CH_2_CH_2_COOH	3-CH_3_	Naphth-2-yl	6.2 ± 0.1 (31 ± 1%)	2.7 ± 0.3 (30 ± 1%)	7.1 ± 1.3 (6 ± 1%)	i.a.
**17**	3-CH_2_CH_2_COOH	4-CH_3_	Naphth-2-yl	1.7 ± 0.2 (67 ± 3%)	0.17 ± 0.02 (34 ± 1%)	1.4 ± 0.3 (21 ± 2%)	1.5 ± 0.1 (21 ± 1%)
**18**	3-CH_2_CH_2_COOH	5-CH_3_	Naphth-2-yl	0.80 ± 0.02 (25 ± 1%)	0.21 ± 0.01 (14 ± 1%)	3.3 ± 0.1 (23 ± 1%)	i.a.
**19**	3-CH_2_CH_2_COOH	6-CH_3_	Naphth-2-yl	7.4 ± 0.4 (15 ± 1%)	6.3 ± 0.9 (20 ± 1%)	11 ± 2 (2.5 ± 0.2%)	i.a.
**20**	3-CH_2_CH_2_COOH	4-Cl	Naphth-2-yl	3.0 ± 0.1 (16 ± 1%)	1.9 ± 0.2 (16 ± 1%)	1.4 ± 0.1 (6.0 ± 0.1%)	i.a.
**21**	3-CH_2_CH_2_COOH	4-OH	Naphth-2-yl	5.3 ± 0.5 (54 ± 3%)	2.4 ± 0.1 (34 ± 1%)	2.3 ± 0.3 (20 ± 2%)	4.5 ± 0.1 (16 ± 1%)
**22**	3-CH_2_CH_2_COOH	4-OCH_3_	Naphth-2-yl	0.30 ± 0.04 (43 ± 2%)	0.06 ± 0.01 (32 ± 1%)	1.3 ± 0.1 (27 ± 1%)	11 ± 3 (22 ± 1%)
**23**	3-CH_2_CH_2_COOH	4-OCF_3_	Naphth-2-yl	1.1 ± 0.3 (31 ± 4%)	0.38 ± 0.06 (30 ± 2%)	1.0 ± 0.2 (15 ± 1%)	1.1 ± 0.1 (12 ± 1%)
**24**	3-CH_2_CH_2_COOH	4-^*t*^Bu	Naphth-2-yl	0.31 ± 0.08 (63 ± 8%)	0.27 ± 0.02 (25 ± 1%)	1.5 ± 0.1 (20 ± 1%)	0.30 ± 0.01 (13 ± 1%)
**25**	3-CH_2_CH_2_COOH	5-Cl	Naphth-2-yl	0.9 ± 0.2 (42 ± 2%)	0.28 ± 0.03 (17 ± 1%)	6 ± 1 (26 ± 2%)	2.6 ± 0.4 (12 ± 1%)
**26**	3-CH_2_CH_2_COOH	5-OH	Naphth-2-yl	8.9 ± 0.8 (28 ± 2%)	10 ± 2 (24 ± 2%)	13 ± 1 (9 ± 1%)	i.a.
**27**	3-CH_2_CH_2_COOH	5-OCH_3_	Naphth-2-yl	0.23 ± 0.04 (53 ± 2%)	0.36 ± 0.01 (23 ± 1%)	2.0 ± 0.1 (15 ± 1%)	i.a.
**28**	3-CH_2_CH_2_COOH	5-OCF_3_	Naphth-2-yl	0.21 ± 0.02 (54 ± 1%)	0.10 ± 0.01 (19 ± 1%)	1.1 ± 0.2 (18 ± 2%)	i.a.
**29**	3-CH_2_CH_2_COOH	4,5-Methylenedioxy	Naphth-2-yl	0.46 ± 0.03 (25 ± 1%)	0.4 ± 0.1 (31 ± 3%)	0.9 ± 0.3 (15 ± 2%)	2.9 ± 0.6 (14 ± 1%)
**30**	3-CH_2_CH_2_COOH	—	1,1'-Biphenyl-4-yl	>30	2.5 ± 0.5 (38 ± 4%)	1.6 ± 0.2 (16 ± 1%)	1.3 ± 0.2 (33 ± 1%)
**31**	3-CH_2_CH_2_COOH	—	4-(Furan-2-yl)phenyl	1.8 ± 0.3 (22 ± 1%)	0.20 ± 0.01 (43 ± 1%)	2.2 ± 0.1 (9 ± 1%)	5.4 ± 0.1 (23 ± 1%)
**32**	3-CH_2_CH_2_COOH	—	4-(Furan-3-yl)phenyl	1.6 ± 0.3 (31 ± 2%)	2.4 ± 0.2 (57 ± 3%)	4.2 ± 0.9 (38 ± 5%)	0.23 ± 0.01 (20 ± 1%)
**33**	3-CH_2_CH_2_COOH	—	4-(Thiophen-2-yl)phenyl	1.5 ± 0.1 (27 ± 1%)	0.64 ± 0.06 (35 ± 1%)	1.4 ± 0.2 (11 ± 1%)	1.7 ± 0.4 (21 ± 1%)
**34**	3-CH_2_CH_2_COOH	—	4-(Thiophen-3-yl)phenyl	1.7 ± 0.1 (32 ± 1%)	1.6 ± 0.3 (59 ± 5%)	1.3 ± 0.2 (17 ± 1%)	0.6 ± 0.1 (18 ± 1%)
**35**	3-CH_2_CH_2_COOH	—	4-(Isoxazol-3-yl)phenyl	8.0 ± 0.4 (11 ± 1%)	5.6 ± 0.4 (29 ± 1%)	5.8 ± 0.8 (30 ± 2%)	6.8 ± 0.6 (32 ± 1%)
**36**	3-CH_2_CH_2_COOH	—	4-(Isoxazol-4-yl)phenyl	Unstable	Unstable	Unstable	Unstable
**37**	3-CH_2_CH_2_COOH	—	4-(Oxazol-4-yl)phenyl	20 ± 1 (36 ± 3%)	12 ± 1 (52 ± 2%)	6 ± 1 (15 ± 1%)	6.9 ± 0.3 (41 ± 1%)
**38**	3-CH_2_CH_2_COOH	—	4-(Oxazol-5-yl)phenyl	> 30	12 ± 1 (32 ± 3%)	5.3 ± 0.2 (16 ± 1%)	15 ± 4 (53 ± 4%)
**39**	3-CH_2_CH_2_COOH	—	4-(1,2,3-Triazol-1-yl)phenyl	i.a.	i.a.	>30	i.a.
**40**	3-NHCH_2_COOH	4-OCH_3_	4-(Furan-3-yl)phenyl	>30	1.6 ± 0.1 (54 ± 2%)	7.7 ± 0.5 (9 ± 1%)	0.29 ± 0.01 (43 ± 1%)
**41**	3-CH_2_CH_2_COOH	4-^*t*^Bu	4-(Furan-2-yl)phenyl	0.17 ± 0.04 (72 ± 5%)	0.14 ± 0.01 (34 ± 1%)	2.4 ± 0.1 (16 ± 1%)	0.015 ± 0.002 (19 ± 1%)

Results are expressed as the mean ± SEM; *n* = 3. Maximum relative activation (max. rel. act.) refers to the activity of reference compounds GW7647 (PPARα), rosiglitazone (PPARγ), L165,041 (PPARδ) at 1 µM, and GW4064 (FXR) at 3 µM.

*i.a.* inactive at 10 μM, *tox.* toxic at 30 μM.

For the subsequent structural optimization of the anthranilamide motif (Table 1), we retained the favored propanoic acid chain of **9**. To identify promising positions for additional substituents, a methyl group was first introduced in positions 3–6 of the central aromatic ring (**16**–**19**). FXR tolerated methylation only in 4-position (**17**), whereas the SAR was more distinguished on PPARs. 3-Methyl (**16**) or 6-methyl (**19**) substitutions were tolerated by all three subtypes despite loss in activity and 4-methylation (**17**) or 5-methylation (**18**) selectively promoted potency on PPARδ while hardly affecting PPARα/γ modulation.

Based on these preliminary SAR observations, we probed further substituents in 4- and 5-positions. A 4-chlorine substituent (**20**) as well as a hydroxyl group in 4-position (**21**) failed to increase potency on PPARs and were not favored by FXR. 4-Methoxy analog **22** gained in potency on PPARα and PPARδ but markedly diminished FXR agonism. 4-Trifluoromethoxy derivative **23**, in contrast, displayed balanced micromolar activity on all PPARs and FXR. The sterically more demanding 4-*tert*-butyl group (**24**) achieved further improvement in potency resulting in a nanomolar triple FXR/PPARα/δ modulator with approximately fivefold preference over PPARγ rendering the 4-*tert*-butyl substituent as most favored motif in 4-position of the anthranilamide scaffold. Among substituents in 5-position (**25**–**28**), only chlorine derivative **25** was active on FXR and neither chlorine (**25**) nor a hydroxyl group (**26**) enhanced potency on PPARs. 5-Methoxy (**27**) or 5-trifluoromethoxy (**28**) substituents, however, promoted activity on PPARα. Aiming to combine the favored methoxy groups in 4 and 5 positions, we studied the cyclized benzo-1,3-dioxole analog **29**, which displayed considerable balanced potency on PPARs and was also active on FXR but failed to outmatch **24** in terms of triple FXR/PPARα/δ modulation.

We then focused on the SAR of the *N*-acyl substituent with the objective to overcome the highly lipophilic and poorly soluble naphthalene motif of **9**. Initially, we aimed to replace the naphthalene moiety with a series of 2*H*-chromenes (**S1**–**S11**). In addition to enhanced polarity, this chemotype also offered straightforward access to introduction of further substituents into the heterocyclic system. However, **S1**–**S11** revealed a very flat SAR on PPARs and loss of activity on FXR (Supplementary Table 1 and Supplementary Figs. 1 and 2) prompting us to drop the 2*H*-chromene scaffold. Instead, we focused on 4-aryl-substituted benzoyl residues that promised preferable potency on PPARδ (Table 1)^21^. Biphenyl **30** retained PPARδ, PPARγ, and FXR potency of **9** but was not tolerated by PPARα. The compromised activity on PPARα could be restored by furan and thiophene derivatives (**31**–**34**) without preference for either isomer of the five-membered heterocycles. However, while the 2-isomers (**31**, **33**) of the five-membered ring residues enhanced potency on PPARδ, FXR favored the 3-isomers (**32**, **34**). Therein, the smaller furan derivatives **31** and **32** displayed higher potency. Aiming to combine FXR and PPARδ-favored modifications in a single molecule, we evaluated five-membered rings comprising two heteroatoms to potentially address directed contacts with PPARδ (2-position) and FXR (3-position). Isoxazole **35** and oxazoles **37** and **38**, however, turned out to be less active on PPARs and FXR, which is likely due to their enhanced polarity that is poorly tolerated by the lipophilic ligand-binding sites. 4-Isoxazolyl analog **36** was unstable and could not be characterized. Incorporation of another heteroatom in 1,2,3-triazole **39** further followed the trend of **35**, **37**, and **38** and markedly decreased activity on all receptors.

**Fig. 5:**
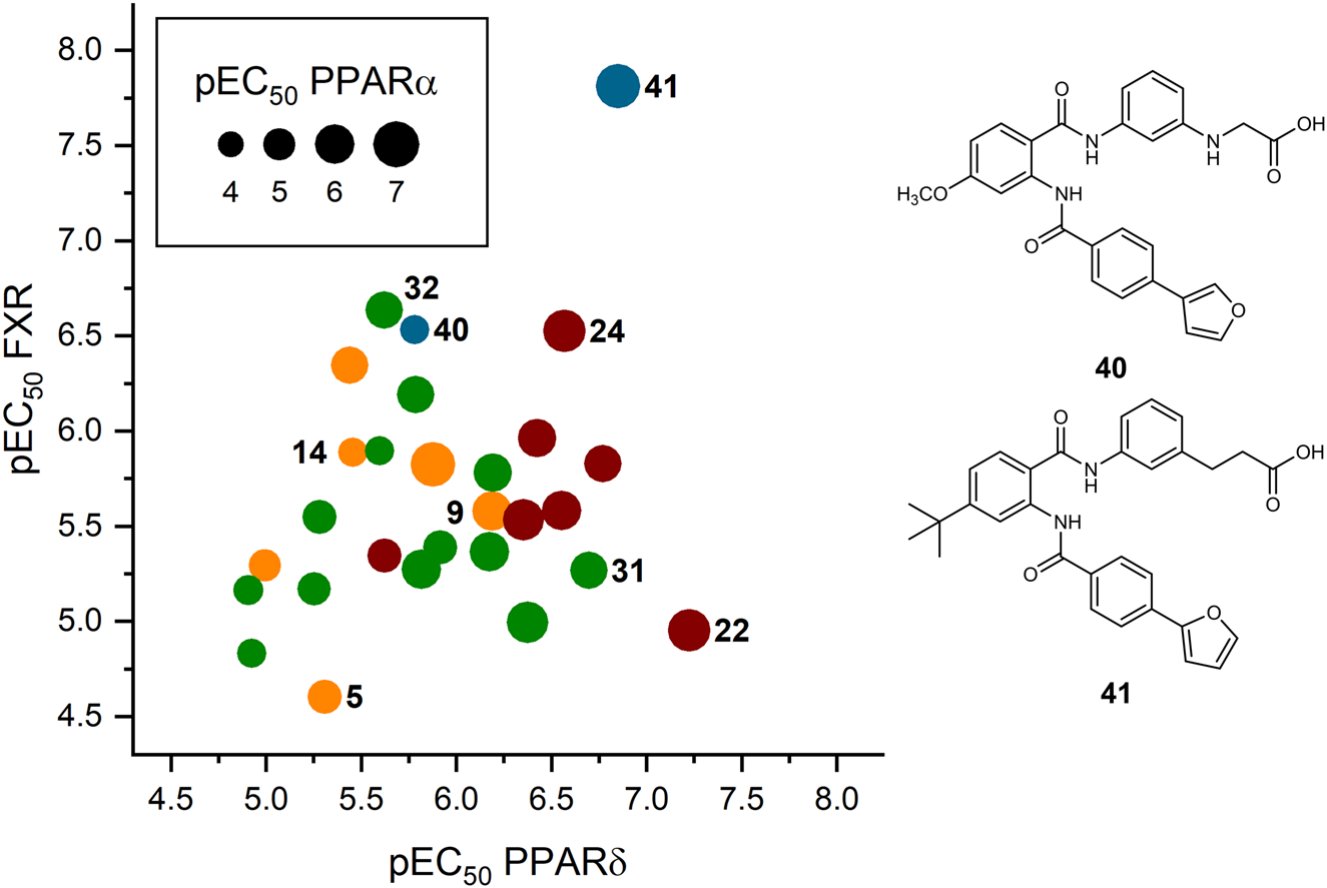
Activity plot for triple FXR/PPARα/δ activators. Graphical representation of pEC_50_ values on the targets PPARδ (abscissa), FXR (ordinate), and PPARα (bubble size). Color indicates the site of modification (orange—head group, red—anthranilamide, green—acyl substituent). The structural features of favored molecules were fused in **40** and **41** shown in blue.

Our systematic multi-objective SAR evaluation revealed several structural variations of lead compound **5** that promoted potency on individual PPARs or FXR. Since no single modification alone achieved the desired triple FXR/PPARα/δ modulatory profile with preference over the PPARγ subtype, we aimed to design a balanced nanomolar FXR/PPARα/δ partial agonist by fusing the obtained SAR knowledge. We sought to combine structural modifications individually increasing potency on one of the desired targets. Therein, the propanoic acid (**9**) and glycine (**14**) motifs evolved as preferred acidic side chain residues, 4-methoxy (**22**) and 4-*tert*-butyl (**24**) were the favored substituents on the anthranilamide core, and the 2-furyl (**31**) as well as 3-furyl (**32**) residues evolved as preferable terminal moieties. From these motifs, we designed the fused derivatives **40** and **41** and rationally combined structural features that were favored by the desired protein targets while sparing the PPARγ subtype (Fig. 5 and Table 1).

**40** was designed as dual FXR/PPARδ agonist with selectivity over PPARα from the acidic side chain of **14** to incorporate selectivity over PPARα and PPARγ, the PPARδ-favored 4-methoxy-anthranilamide of **22**, and the FXR-favored 3-furyl residue of **32**. The resulting compound **40** retained selectivity for PPARδ over PPARα/γ and displayed nanomolar activity on FXR but failed to achieve submicromolar potency on PPARδ. Combination **41** was designed as triple FXR/PPARα/δ modulator incorporating the 4-*tert*-butyl substituent of **24**, which was favored by all three targets, the 2-furyl motif of **31** was chosen to promote potency on PPARδ, and the FXR-favored propanoic acid side chain of **9** was incorporated to promote FXR modulation. **41** revealed the desired profile of a triple FXR/PPARα/δ modulator with balanced nanomolar potency and marked preference over PPARγ.

Molecular docking of **41** (Supplementary Fig. 3) to the ligand-binding sites of FXR (pdb code 4QE8^10^), PPARα (pdb code 2P54^22^), and PPARδ (pdb code 5U3R^21^) rationalized the SAR of the chemotype and the polypharmacology of **41**. The triple modulator favorably occupied the lipophilic ligand-binding sites of all three nuclear receptors and aligned well with the co-crystallized ligands. In FXR and PPARα, the central *tert*-butylbenzene motif extended to lipophilic sub-pockets (Ile352 in FXR, Val324 and Leu331 in PPARα) that were not occupied by the reference ligands. The phenylpropionic acid engaged the canonical neutralizing interaction with Arg331 of FXR and participated in hydrogen bond networks with the activation functions of PPARα (Tyr314, His440, Tyr464) and PPARδ (His287, His413, Tyr437). Additionally, the amide oxygen of **41** formed polar contacts with Met328 in FXR, and with Ser280 and Met330 in PPARα, some of which were water mediated. No other polar interactions were observed in the predicted binding modes of **41** to the lipophilic-binding sites of the nuclear receptors.

### In vitro profiling and target engagement of the triple FXR/PPARα/δ modulator

**Fig. 6:**
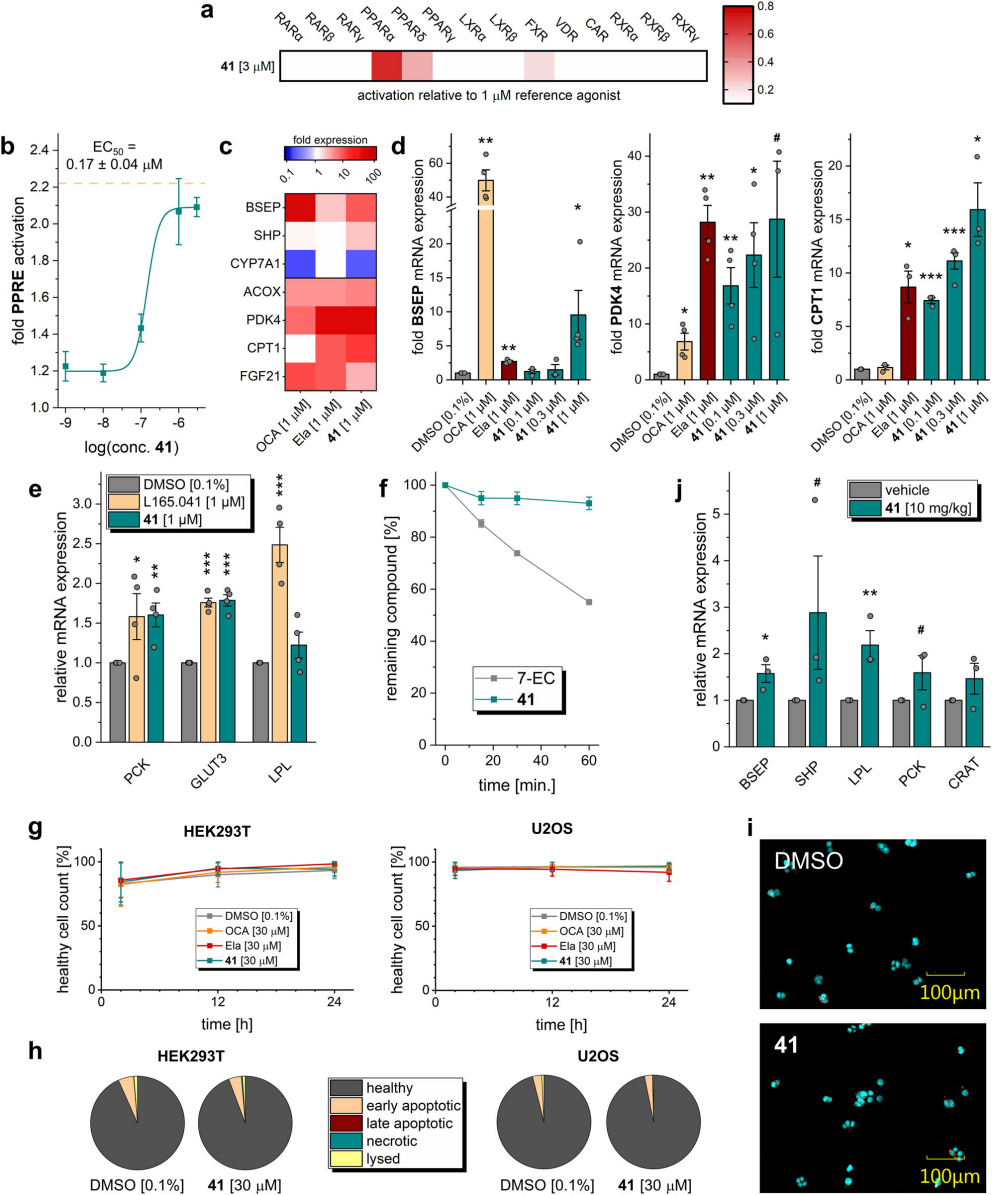
In vitro and pilot in vivo profiling of **41**. **a** **41** displayed high selectivity over nuclear receptors related to PPARs and FXR. Heatmap shows mean relative nuclear receptor activation of three independent experiments. **b** **41** activated a full-length human PPAR response element (PPRE) reporter in HEK293T cells with equal potency as observed in the Gal4 hybrid setting. The dashed line corresponds to the activation of reference agonist L165,041 (1 μM). Results are the mean ± SEM, *n* = 3. **c** **41** (1 µM) modulated mRNA expression of FXR-regulated (BSEP, SHP, and CYP7A1) and PPAR-regulated (ACOX, PDK4, CPT1, FGF21) genes in HepG2 cells thereby fusing the activities of obeticholic acid (OCA) and elafibranor (Ela). Heatmap shows mean fold mRNA induction compared to vehicle-treated cells, *n* = 4. Individual genes are shown in **d** and Supplementary Fig. 4. **d** **41** induced expression of FXR-regulated BSEP, PPARδ-regulated PDK4, and PPARα-regulated CPT1 in a dose-dependent fashion. Results are mean ± SEM fold mRNA expression compared to vehicle-treated cells, *n* = 3. ***p < 0.001, **p < 0.01, *p < 0.05, ^#^*p* < 0.1 (*t* test vs. DMSO-treated cells). **e** **41** stimulated mRNA expression of PPARδ-regulated genes PCK, GLUT3, and LPL in C2C12 cells (reference L165,041). Results are mean ± SEM fold mRNA expression compared to vehicle-treated cells, *n* = 4. ***p < 0.001, **p < 0.01, *p < 0.05 (*t* test vs. DMSO-treated cells). **f** **41** was very stable against microsomal degradation. 7-Ethoxycoumarin (7-EC) for comparison. Results are the mean ± SEM, *n* = 3. **g** Healthy cell count [%] after 2, 12, and 24 h of 30 µM compound exposure (**41**, obeticholic acid, elafibranor) or 0.1% DMSO control in HEK293T and U2OS cells. **h** Rates of healthy, early apoptotic, late apoptotic, necrotic, and lysed cells after 24 h exposure of HEK293T or U2OS cells to 30 µM **41**. **i** Fluorescent image of HEK293T cells after 24 h exposure to 30 µM **41** compared to 0.1% DMSO control (blue: Hoechst33342, green: Annexin V, red: Yo-Pro3). Full multiplex toxicity data in Supplementary Fig. 5. **j** Pilot profiling of **41** (10 mg/kg p.o.) in mice also confirmed target engagement in vivo as observed by upregulation of FXR (BSEP, SHP) and PPAR (LPL, PCK, CRAT) regulated genes in liver compared to vehicle-treated animals 12 h after administration. Results are the mean ± SEM, *n* = 3. **p < 0.01, *p < 0.05, ^#^*p* < 0.1 (*t* test vs. vehicle).

With the discovery of **41**, we succeeded in designing a triple modulator of PPARα (EC_50_ 168 nM), PPARδ (EC_50_ 141 nM), and FXR (EC_50_ 15 nM) comprising high potency on all desired protein targets. Thus **41** appeared sufficiently active to probe the therapeutic potential of this designed polypharmacological profile. To further evaluate the suitability of **41** for sophisticated in vivo experiments, we broadly profiled the compound in vitro. Selectivity screening of **41** among nuclear receptors related to PPARs and FXR revealed no off-target liability demonstrating that the favorable selectivity of the chemotype was conserved (Fig. 6a). In agreement with its potency on Gal4 hybrids of PPARα and PPARδ, **41** also activated a human full-length PPAR response element (PPRE)^23^ reporter in HEK293T cells with 0.17 µM EC_50_ value (Fig. 6b).

To further probe target engagement in a cellular setting, we treated human liver cells (HepG2) with **41** (1 µM) and observed changes in FXR- and PPAR-regulated gene expression (Fig. 6c, d and Supplementary Fig. 4). OCA (1 µM) and elafibranor (1 µM) were used as references. **41** and OCA-stimulated FXR activity in HepG2 cells as observed by upregulation of BSEP and small heterodimer partner (SHP) mRNA as well as downregulation of CYP7A1. Compared to OCA, the effects of **41** on FXR-regulated BSEP, SHP, and CYP7A1 were moderate, further confirming the partial FXR agonist profile of **41**, which was already observed in the reporter gene assay and is a characteristic of this FXR modulator chemotype^10^.

Except for a weak upregulation of BSEP, elafibranor had no effect on FXR activity. Instead, the dual PPARα/δ agonist strongly promoted the expression of PPAR-regulated genes peroxisomal acyl-coenzyme A oxidase 1 (ACOX), mitochondrial pyruvate dehydrogenase lipoamide kinase 4 (PDK4), carnitine palmitoyltransferase 1 (CPT1), and fibroblast growth factor 21 (FGF21) in HepG2 cells. Treatment with **41** caused a similar expression profile of PPAR-regulated genes as elafibranor. OCA slightly induced ACOX and FGF21 expression as well, which is likely due to FXR-mediated PPARα upregulation. For FXR-regulated BSEP, PPARδ-regulated PDK4, and PPARα-regulated CPT1, we observed dose-dependent effects of **41** on gene expression further confirming the designed polypharmacology of **41** resembling a combination of OCA and elafibranor (Fig. 6d).

Since PPARδ may also contribute to this polypharmacology through peripheral effects, e.g., in the skeletal muscle, we probed PPARδ activation by **41** in murine C2C12 myoblasts, which were differentiated to myotubes before treatment. **41** upregulated phosphoenol-pyruvate carboxykinase 1 (PCK) and the glucose transporter 3 (GLUT3) with comparable efficacy as the selective PPARδ agonist L165,041 while its effect on lipoprotein lipase (LPL) expression was less pronounced (Fig. 6e). In summary, the triple modulator **41** achieved the desired pharmacodynamic effects in relevant cellular settings and appeared suitable for in vivo studies.

Before proof-of-concept in vivo evaluation, we profiled stability and cytotoxicity of the triple modulator **41** in vitro. **41** displayed very high stability against microsomal degradation (Fig. 6f) further suggesting suitability for in vivo experiments. To determine cytotoxicity, we employed a multiplex high-content life-cell imaging screen^24^ on HEK293T and U2OS cells. For this, the cells were treated with fluorescent probes to detect Annexin V as apoptosis marker (Annexin V Alexa488), dead cells (Yo-Pro3), and DNA/nucleus (Hoechst33342). Cells were then incubated with **41**, OCA, or elafibranor at seven concentrations between 100 nM and 100 µM, and cell health was determined by fluorescence imaging after 2, 12, and 24 h (Fig. 6g–i and Supplementary Fig. 5). The triple modulator exhibited no notable toxic effects up to high 100 µM concentration and did not markedly promote apoptosis, necrosis, or lysis of cells during the 24 h exposure time. Also the two reference compounds OCA and elafibranor were non-toxic up to 100 µM concentration over 24 h.

The attractive selectivity profile, robust target engagement in native cellular setting, favorable stability in vitro, and lack of cytotoxicity prompted us to evaluate the ability of **41** to modulate FXR- and PPAR-regulated gene expression in vivo in a pilot animal experiment. We treated six mice with a single oral dose of **41** (10 mg/kg) and three mice with vehicle and analyzed FXR and PPARα/δ-regulated mRNA expression in the mouse livers 12 h after treatment (three mice each). Expression levels of FXR-regulated genes BSEP and SHP as well as PPAR-regulated genes LPL, PCK, and carnitine *O*-acetyltransferase (CRAT) were increased in animals receiving **41**, which demonstrated that the triple modulator efficiently engaged its targets in vivo as well (Fig. 6j). After 18 h, the effect on FXR- and PPAR-regulated gene expression in the mouse livers was significantly reduced (Supplementary Fig. 6) suggesting a need for a 12h dosing interval at a 10 mg/kg dose.

### Efficacy of the triple FXR/PPARα/δ modulator in DIO NASH

**Fig. 7:**
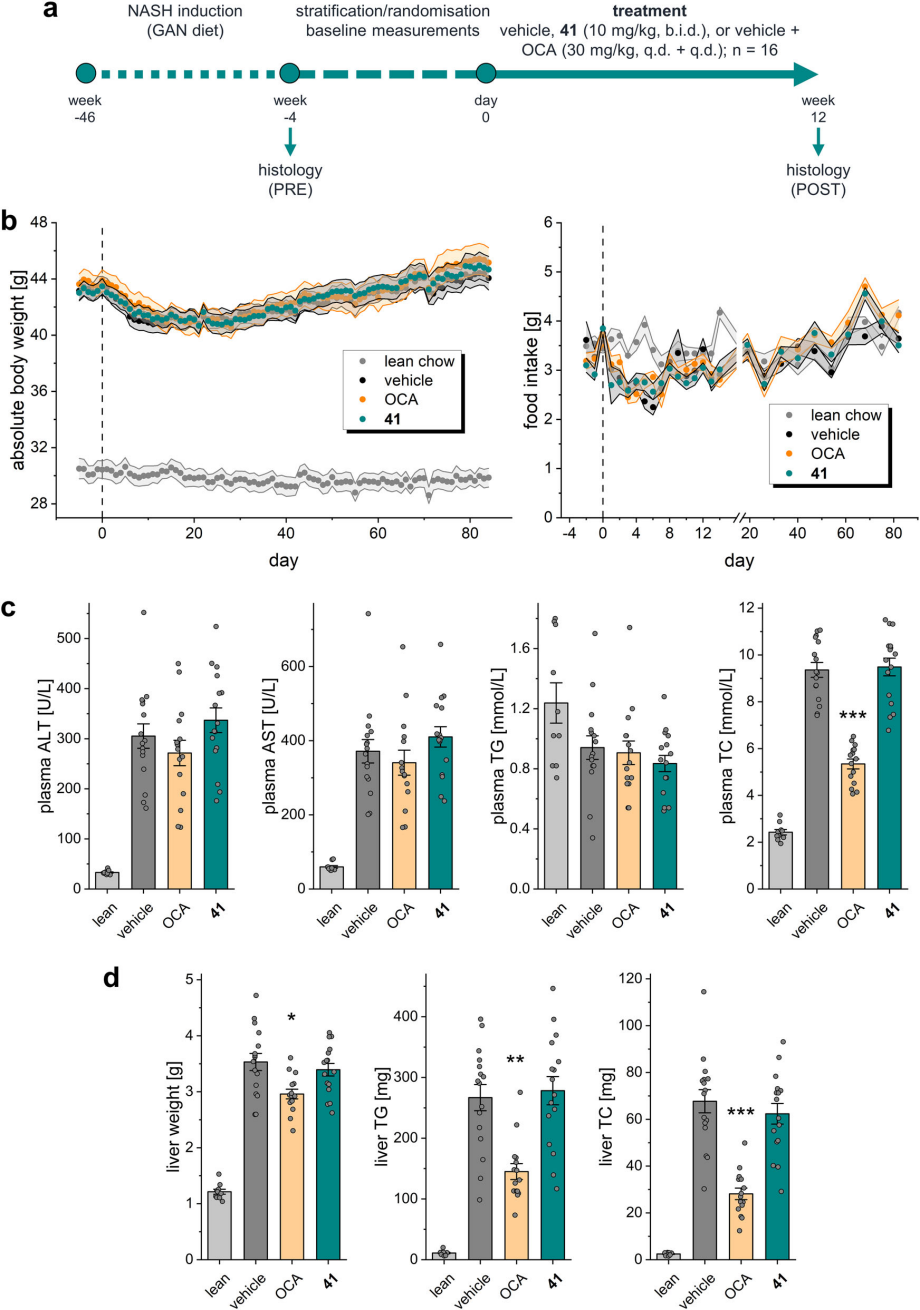
Characterization of **41** in diet-induced obese (DIO) NASH in mice. **a** Study outline. **b** Absolute body weight and food intake of mice during the treatment phase. **c** Biochemical parameters at termination (12 weeks of treatment) in the treatment groups. Results are the mean ± SEM. ***p < 0.001 vs. vehicle (Dunnett’s test one-factor linear model). **d** Liver parameters at termination (12 weeks of treatment) in the treatment groups. Results are the mean ± SEM. **p < 0.01, *p < 0.05 vs. vehicle (Dunnett’s test one-factor linear model). Sample sizes: *n* = 10 for lean chow, *n* = 15 for obeticholic acid, *n* = 16 for vehicle and **41**.

In vitro and pilot in vivo profiling of **41** demonstrated suitability of the triple FXR/PPARα/δ modulator for more sophisticated in vivo studies, and thus we applied **41** to the DIO NASH model in mice (Fig. 7). We chose this rodent model of NASH since it is considered as highly reliable disease model and has also served to evaluate the effects of combined OCA/elafibranor treatment^19^. In this experiment, NASH was induced in mice over 46 weeks by a diet containing 40% fat (primarily palm oil), 40% carbohydrate (20% fructose), and 2% cholesterol (Gubra AMLN NASH diet, GAN diet). Forty-eight mice (16 animals per group) with pre-biopsy-confirmed NASH were included in the treatment phase and subsequently received **41** (10 mg/kg, b.i.d.), OCA (30 mg/kg), or vehicle with continued GAN diet for 12 weeks (Fig. 7a). All animals behaved normally throughout the treatment phase. There was no difference in food intake and body weight (Fig. 7b) between mice receiving **41** and OCA or vehicle-treated animals. All animals completed the study except one from the OCA group, which was found dead during the second week of the treatment phase. After 4 weeks of treatment, biochemical parameters were not significantly different between the groups except for reduced total cholesterol (TC) plasma levels in the OCA group (Supplementary Fig. 7). At termination (12 weeks of treatment), plasma levels of alanine transaminase (ALT), and aspartate transaminase (AST) as well as plasma triglycerides (TG) were not different between groups (Fig. 7c). Animals receiving OCA displayed reduced TC levels.

**Fig. 8:**
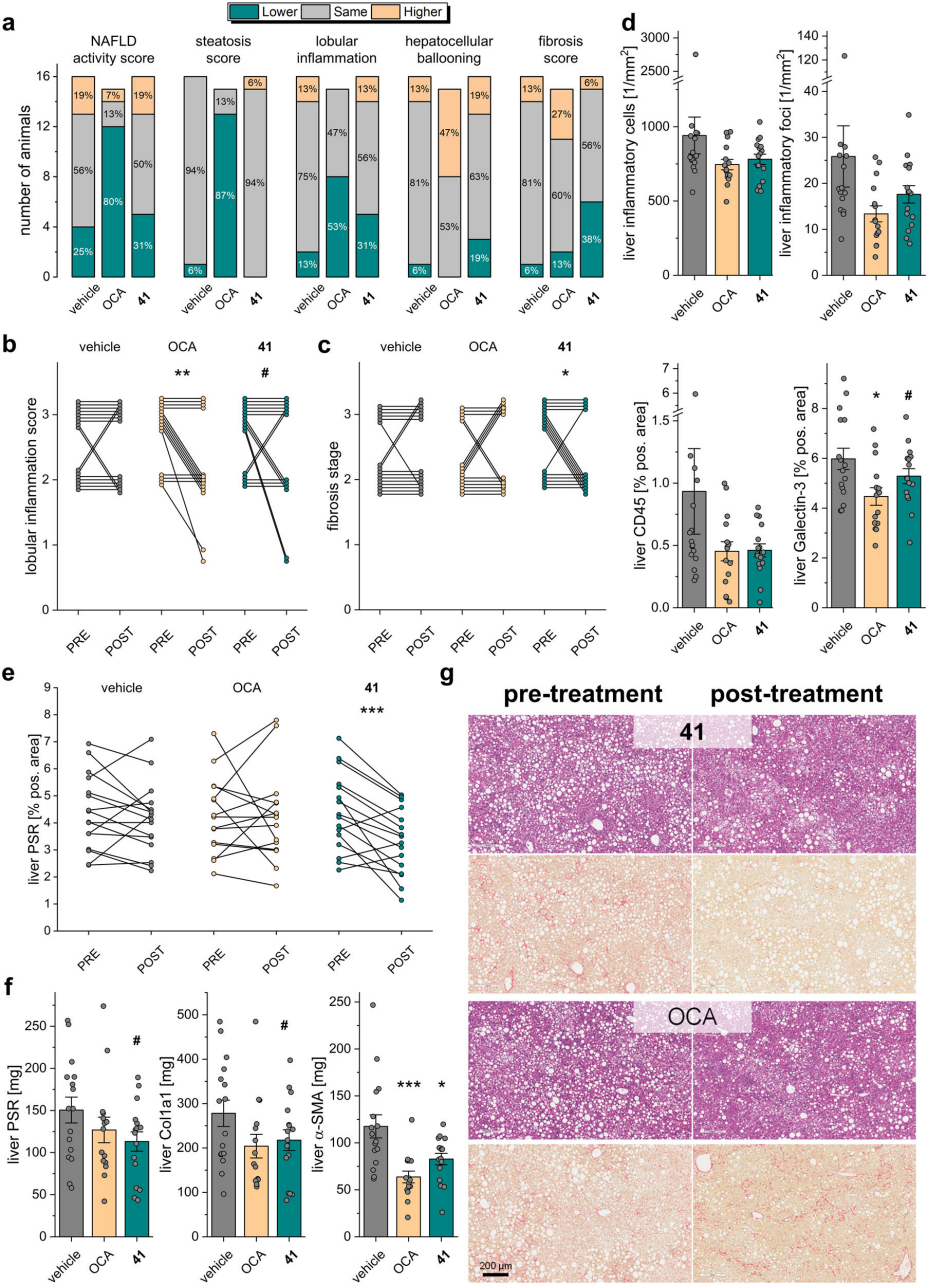
Therapeutic effects of **41** in diet-induced obese (DIO) NASH in mice. **a** NAFLD-related scores for the treatment groups. **b** Pre- and post-treatment lobular inflammation scores for the individual animals in the treatment groups. **p < 0.01, ^#^*p* < 0.1 (paired *t* test). **c** Pre- and post-treatment fibrosis scores for the individual animals in the treatment groups. *p < 0.05 (paired *t* test). **d** Effect of treatments on liver inflammatory markers. *p < 0.05, ^#^*p* < 0.1 vs. vehicle (two-way ANOVA). **e** Pre- and post-treatment liver PSR-positive area for the individual animals in the treatment groups. ***p < 0.001 (paired *t* test). **f** Effect of treatments on liver fibrosis markers. ***p < 0.001, *p < 0.05, ^#^*p* < 0.1 vs. vehicle (two-way ANOVA). **g** Representative histology images from pre- and post-treatment biopsies with H&E and PSR staining for the **41** and obeticholic acid treatment groups. Each set of images was taken from biopsies of the same animal. Sample sizes: *n* = 15 for obeticholic acid, *n* = 16 for vehicle and **41**.

After 12 weeks of treatment, animals were sacrificed, and the livers were studied for NAFLD markers. We found that treatment with **41** had no pronounced effect on steatosis while OCA strongly reduced hepatic fat content and steatosis score (Fig. 8a) as described previously^19^. Livers of animals receiving **41** did not differ from vehicle-treated mice in terms of liver weight, TG, and TC content (Fig. 7d). The potent and efficacious FXR agonist OCA lowered liver weight, liver TG, and liver TC. Of note, **41** behaves as partial agonist on FXR^10^ with approximately 1/5 FXR activation efficacy compared to OCA as observed in the reporter gene assay (Table 1) and for mRNA expression levels (Fig. 6d). Thus, comparing this observation also to the previously reported effects of OCA, elafibranor or combined OCA/elafibranor treatment in an equivalent model^19^ may suggest that full FXR agonism is required to counter hepatic steatosis. High-dose OCA (30 mg/kg) and especially the combination OCA/elafibranor (30 mg/kg + 10 mg/kg) caused marked reductions in steatosis while the anti-steatotic effect of sole elafibranor (10 mg/kg) treatment was less pronounced pointing to a crucial role of FXR activation in reversing hepatic steatosis.

Animals receiving **41** and animals receiving OCA experienced an improvement in lobular inflammation compared to vehicle (**41**: 5/16; OCA: 8/15; vehicle: 2/16; Fig. 8a). Two mice treated with **41** and one receiving OCA displayed a strongly pronounced anti-inflammatory effect with improvement from stage 3 to stage 1 (Fig. 8b). Both **41** and OCA decreased the number of inflammatory cells and inflammatory foci in the liver compared to vehicle (Fig. 8d). Liver Galectin-3 (Gal3) and particularly liver CD45 as markers of inflammation were reduced by both treatments (Fig. 8d).

The most striking effect of **41** in DIO NASH was its anti-fibrotic activity. The triple modulator **41** reversed fibrosis in 6/16 animals (OCA: 2/15; vehicle: 1/16; Fig. 8a, c). Fibrosis worsening was observed in only 1/16 animals (OCA: 4/15; vehicle: 2/16). Animals receiving **41** displayed a significant reduction of liver picrosirius red (PSR) after the treatment period (Fig. 8e). Compared to vehicle-treated animals, fibrosis markers PSR, collagen1a1 (Col1a1), and α-smooth muscle actin (α-SMA) were decreased by treatment with **41** (Fig. 8f). Notably, OCA and elafibranor achieved only weak anti-fibrotic activity compared to vehicle in an equivalent, previously reported study^19^. Their combined administration revealed increased anti-fibrotic efficacy but did not reverse fibrosis^19^ further highlighting the promising anti-fibrotic activity of triple modulator **41**. The attractive anti-NASH activity of **41** is also obvious from representative histology images showing hematoxylin–eosin (H&E) and PSR staining in pre- and post-treatment biopsies (Fig. 8g and Supplementary Fig. 8) as well as α-SMA, Col1a1, Gal3, and CD45 staining in post-treatment biopsies (Supplementary Figs. 9 and 10).

## Conclusions

The concept of designed polypharmacology^25^, i.e., the concerted modulation of multiple molecular targets with a single molecule for the treatment of one pathology, is attracting considerable interest. Such approaches hold great promise especially in multi-factorial diseases where a single mode of action fails to exhibit sufficient therapeutic efficacy. NAFLD/NASH is such a multifactorial disease that might particularly benefit from combined modes of action. Both FXR and PPARα/δ agonists have been studied in late-stage clinical trials to treat NASH, and while attractive therapeutic effects were observed for OCA and elafibranor, the individual efficacy of these compounds appears insufficient to reverse NASH.

Sophisticated animal models of NASH have revealed various effects of selective agonists of FXR, PPARα, and PPARδ. The FXR agonist OCA exhibited pronounced anti-steatotic activity and slight anti-fibrotic effects in DIO NASH^26^, which aligns well with our observations. The selective PPARα activator pemafibrate was recently found to counter hepatocyte ballooning in STAM NASH without affecting hepatic steatosis and inflammation^27^, and the selective PPARδ agonist seladelpar improved steatosis and hepatocyte ballooning in (*foz*/*foz*) mice under atherogenic diet^15^ without a pronounced effect on hepatic inflammation. Together, these observations suggest that activation of FXR, PPARα, or PPARδ contributes differential anti-NASH effects and render these modes of action attractive for combination. Previous in vivo studies with the dual PPARα/γ agonist saroglitazar have already demonstrated superior activity of such multi-mode-of-action compound in NASH treatment. In choline-deficient, L-amino acid-defined, high-fat diet-induced NASH in mice, saroglitazar exhibited stronger anti-steatosis efficacy than the selective agents fenofibrate (PPARα) and pioglitazone (PPARγ)^28^.

Following these considerations, we have designed a triple activator of FXR, PPARα, and PPARδ to join the pharmacodynamic effects of the leading anti-NASH drug candidates OCA (**1**) and elafibranor (**4**). To succeed in this multi-objective optimization effort, several minor structural modifications were necessary to tune the activity toward balanced triple modulation. The resulting compound **41** was metabolically stable and non-toxic and engaged its molecular targets in cellular and in vivo settings demonstrating its suitability to further probe the concept of FXR/PPARα/δ activation in sophisticated in vivo models of NASH. Consequently, we applied **41** to DIO NASH in mice as accredited model of the pathology and employed the FXR agonist OCA as control. In this model, the disease was diet induced over 46 weeks before the pharmacological intervention was initiated, which allows evaluation of the compound in a curative fashion. The results of this large in vivo study draw two important conclusions. First, the triple modulator **41**, which exhibits only approximately 1/5 of the FXR activation efficacy of OCA, did not markedly reverse steatosis as first hallmark of NAFLD suggesting that strong FXR activation efficacy is required to reduce hepatic fat content. Accordingly, strong anti-steatotic effects have been reported for the strong FXR activator OCA (**1**) in equivalent studies^19,26^. Second, despite this lack of pronounced effects on hepatic lipid content, the triple modulator **41** counteracted hepatic inflammation and reversed fibrosis as second and third hallmarks of the disease. Especially the reversal of hepatic fibrosis observed in animals treated with **41** is remarkable since fibrosis is considered as major predictor of further disease progression of NASH toward liver cirrhosis. While several rodent models of NASH have demonstrated anti-steatosis and anti-inflammatory activity for FXR and PPAR agonists^15,19,26–28^, fibrosis-reversing effects are a rare feature of experimental anti-NASH agents. Although our observations require translation to other preclinical models and to human, the anti-fibrotic activity we have discovered for the triple modulator **41** is very encouraging. Triple FXR/PPARα/δ activation, therefore, evolves as a promising concept for more efficacy in NASH treatment warranting further preclinical development of **41**.

## Methods

### Chemistry

#### General

All chemicals and solvents were obtained from commercial sources in reagent grade and used without further purification. Thin-layer chromatography (TLC) was performed using TLC plates (silica gel 60 F254, 0.2 mm, Merck or Alugram Xtra Sil G/UV 0.2 mm, Macherey-Nagel) with detection under ultraviolet (UV) light (254 and 366 nm). Preparative column chromatography was performed using Silicagel 60 (Macherey-Nagel) and solvents of technical grade. Reactions with air- or moisture-sensitive compounds were carried out under argon atmosphere and in anhydrous solvents. Nuclear magnetic resonance (NMR) spectra were recorded on Bruker AM 250 XP, AV 300, AV 400, and AV 500 spectrometers (Bruker Corporation, Billerica, MA, USA). Chemical shifts (*δ*) are reported in ppm relative to tetramethylsilane and coupling constants (*J*) in Hz. Multiplicity of signals is indicated as s for singulet, d for doublet, t for triplet, q for quartet, and m for multiplet. Aromatic signals resembling a triplet that stem from protons with two unequal neighbors but similar or equal coupling constants are denoted as doublets of doublet (dd). Mass spectra were obtained on a VG Platform II (Thermo Fischer Scientific, Inc., Waltham, MA, USA) using electrospray ionization (ESI). High-resolution mass spectra (HRMS) were recorded on a MALDI LTQ ORBITRAP XL instrument (Thermo Fisher Scientific) or on a Bruker maXis ESI-Qq-TOF-MS instrument (Bruker). Compound purity was analyzed on a Varian ProStar HPLC (SpectraLab Scientific Inc., Markham, ON, Canada) equipped with a MultoHigh100 Phenyl-5µ 240 x 4 mm column (CS-Chromatographie Service GmbH, Langerwehe, Germany) using a gradient (H_2_O/MeOH 80:20 + 0.1% formic acid isocratic for 5 min to MeOH + 0.1% formic acid after additional 45 min and MeOH + 0.1% formic acid for additional 10 min) at a flow rate of 1 mL/min and UV detection at 245 and 280 nm. All final compounds for biological evaluation had a purity >95% according to area under the curve at 245 and 280 nm UV detection.

#### Synthesis of 41

*3-(3-[4-{tert-Butyl}-2-{4-(2-furyl)}benzamido]phenyl)propanoic acid (****41****)*: An aqueous solution of LiOH (9.61 mL, 9.61 mmol, 5.0 eq) was added to a solution of **50aj** (1.04 g, 1.92 mmol, 1.0 eq) in tetrahydrofuran (35 mL). The resulting mixture was stirred at ambient temperature for 14 h. Subsequently, the solution was acidified with 5% aqueous hydrochloric acid and extracted three times with EtOAc. The title compound was obtained as a beige solid (973 mg, 99%) by column chromatography on silica using 25% EtOAc in hexane + 2% HOAc as mobile phase. ^1^H-NMR (500 MHz, dimethyl sulfoxide (DMSO)-*d*_6_): *δ* = 1.35 (s, 9H), 2.55 (t, ^*3*^*J* = 7.6 Hz, 2H), 2.83 (t, ^*3*^*J* = 7.6 Hz, 2H), 6.65 (dd, ^*3*^*J* = 3.4 Hz, ^*4*^*J* = 1.8 Hz, 1H), 7.02 (d, ^*3*^*J* = 7.6 Hz, 1H), 7.13 (dd ^*3*^*J* = 3.3 Hz, 1H), 7.28 (dd, ^*3*^*J* = 7.8, 7.8 Hz, 1H), 7.32 (dd, ^*3*^*J* = 8.3 Hz, ^*4*^*J* = 1.9 Hz, 1H), 7.55 (s, 1H), 7.58 (d, ^*3*^*J* = 8.8 Hz, 1H), 7.83 (d, ^*4*^*J* = 1.5 Hz, 1H), 7.85 - 7.92 (m, 3H), 7.98 (d, ^*3*^*J* = 8.5 Hz, 2H), 8.64 (d, ^*4*^*J* = 1.9 Hz, 1H), 10.42 (s, 1H), 11.84 (s, 1H), 12.12 (br s, 1H) ppm. ^13^C-NMR (126 MHz, DMSO-*d*_6_): *δ* = 30.4, 30.8, 34.9, 35.1, 108.0, 112.4, 118.0, 119.0, 119.8, 120.3, 120.9, 123.6, 124.1, 127.8, 128.6, 128.8, 132.9, 133.3, 138.5, 138.8, 141.4, 144.0, 152.1, 155.4, 164.1, 167.4, 173.6 ppm. ESI-MS: *m/z* = 509.06 ([M-H]^-^). HRMS (MALDI): *m*/*z* calculated 533.20469 for C_31_H_30_N_2_O_5_Na, found 533.20371 ([M + Na]^+^).

*Ethyl 3-(3-aminophenyl)propanoate (****43a****)*. 3-(3-Aminophenyl)propanoic acid (**42a**, 3.00 g, 18.2 mmol, 1.0 eq) was dissolved in a 20:1 (v/v) mixture of EtOH and concentrated H_2_SO_4_ (21 mL) and heated to reflux for 2 h. Then the solution was neutralized with Na_2_CO_3_ and extracted three times with CH_2_Cl_2_. The organic layers were combined, dried over Na_2_SO_4_ and the solvent was evaporated under vacuum. Purification by column chromatography on silica using 50% EtOAc in hexane as mobile phase yielded the title compound as a brown oil (3.27 g, 93%). ^1^H-NMR (400 MHz, CDCl_3_): *δ* = 1.24 (t, ^*3*^*J* = 7.1 Hz, 3H), 2.59 (t, ^*3*^*J* = 7.9 Hz, 2H), 2.86 (t, ^*3*^*J* = 7.9 Hz, 2H), 3.62 (br s, 2H), 4.13 (q, ^*3*^*J* = 7.1 Hz, 2H), 6.50 - 6.56 (m, 2H), 6.60 (d, ^*3*^*J* = 7.8 Hz, 1H), 7.07 (dd, ^*3*^*J* = 8.1, 8.1 Hz, 1H) ppm. ^13^C-NMR (75 MHz, CDCl_3_): *δ* = 14.4, 31.1, 36.0, 65.0, 113.2, 115.2, 118.7, 129.5, 142.0, 146.6, 173.2 ppm.

*4-(tert-Butyl)-2-nitrobenzoic acid (****44h****)*. 4-(*tert*-Butyl)-1-methyl-2-nitrobenzene (**60**, 3.50 g, 18.1 mmol, 1.0 eq) was added to a 1:1 (v/v) mixture of pyridine and water (100 mL) and heated to 100 °C. KMnO_4_ (28.6 g, 181 mmol, 10 eq) was added in portions to the solution, which was stirred for additional 4 h. The resulting suspension was filtered after cooling, acidified with 10% aqueous hydrochloric acid, and extracted three times with EtOAc. The combined organic layers were dried over Na_2_SO_4_, and the solvent was removed under reduced pressure. The crude product was purified by column chromatography on silica using 25% EtOAc in hexane as mobile phase to obtain the title compound as a pale-yellow solid (2.04 g, 51%). ^1^H-NMR (300 MHz, CDCl_3_): *δ* = 1.32 (s, 9H), 7.79 - 7.82 (m, 2H), 7.90 (dd, ^*4*^*J* = 1.3, 0.9 Hz, 1H), 13.71 (br s, 1H) ppm. ^13^C-NMR (75 MHz, CDCl_3_): *δ* = 30.5, 35.2, 120.4, 123.8, 129.5, 130.0, 149.1, 156.3, 165.5 ppm.

*Ethyl 3-(3-[4-{tert-butyl}-2-nitrobenzamido]phenyl)propanoate (****46h****)*. A mixture of **44h** (1.04 g, 4.67 mmol, 1.1 eq), EDC·HCl (1.06 g, 5.52 mmol, 1.3 eq), and 4-DMAP (104 mg, 849 μmol, 0.2 eq) was dissolved in CHCl_3_ (15 mL). Then a solution of **43a** (820 mg, 4.24 mmol, 1.0 eq) in CHCl_3_ (5 mL) was added slowly and the mixture was stirred under reflux for 19 h. After cooling to room temperature, water was added and the mixture was extracted three times with CH_2_Cl_2_. The combined organic layers were dried over Na_2_SO_4_, and the solvent was evaporated in vacuo. The title compound was obtained as a brown solid (1.35 g, 80%) by column chromatography on silica using 25% EtOAc in hexane + 2% HOAc as mobile phase. ^1^H-NMR (300 MHz, CDCl_3_): *δ* = 1.23 (t, ^*3*^*J* = 7.1 Hz, 3H), 1.37 (s, 9H), 2.61 (t, ^*3*^*J* = 7.7 Hz, 2H), 2.93 (t, ^*3*^*J* = 7.7 Hz, 2H), 4.11 (q, ^*3*^*J* = 7.1 Hz, 2H), 7.00 (d, ^*3*^*J* = 7.6 Hz, 1H), 7.26 (dd, ^*3*^*J* = 7.8, 7.8 Hz, 1H), 7.37 - 7.49 (m, 2H), 7.54 (d, ^*3*^*J* = 8.0 Hz, 1H), 7.70 (dd, ^*3*^*J* = 8.0 Hz, ^*4*^*J* = 1.6 Hz, 1H), 7.74 (s, 1H), 8.07 (d, ^*4*^*J* = 1.5 Hz, 1H) ppm. ^13^C-NMR (75 MHz, CDCl_3_): *δ* = 14.3, 31.0, 31.1, 35.4, 35.9, 60.7, 118.4, 120.4, 121.8, 125.1, 128.5, 129.4, 130.2, 131.0, 137.8, 141.9, 146.6, 155.3, 164.7, 173.1 ppm.

*Ethyl 3-(3-[2-amino-4-{tert-butyl}benzamido]phenyl)propanoate (****47h****)*. Palladium (10%) on charcoal (358 mg, 336 μmol, 0.1 eq) was added to a solution of **46h** (1.34 g, 3.36 mmol, 1.0 eq) in EtOAc (100 mL) and stirred under hydrogen atmosphere overnight. On the next day, the charcoal was filtered off over celite and the solvent was evaporated. Column chromatography on silica using 25% EtOAc in hexane as mobile phase gave the title compound as a brown solid (1.05 g, 85%). ^1^H-NMR (500 MHz, CDCl_3_): *δ* = 1.24 (t, ^*3*^*J* = 7.2 Hz, 3H), 1.30 (s, 9H), 2.63 (t, ^*3*^*J* = 7.8 Hz, 2H), 2.95 (t, ^*3*^*J* = 7.8 Hz, 2H), 4.13 (q, ^*3*^*J* = 7.1 Hz, 2H), 5.40 (br s, 2H), 6.75 (d, ^*4*^*J* = 1.6 Hz, 1H), 6.77 (dd, ^*3*^*J* = 8.3 Hz, ^*4*^*J* = 1.8 Hz, 1H), 6.98 (d, ^*3*^*J* = 7.6 Hz, 1H), 7.27 (dd, ^*3*^*J* = 7.8, 7.8 Hz, 1H), 7.38 - 7.43 (m, 2H), 7.45 (s, 1H), 7.78 (s, 1H) ppm. ^13^C-NMR (126 MHz, CDCl_3_): *δ* = 14.3, 31.1, 31.1, 34.9, 36.0, 60.6, 113.9, 114.9, 115.1, 118.5, 120.5, 124.5, 127.1, 129.3, 138.2, 141.8, 148.5, 156.7, 167.5, 173.0 ppm.

*4-(2-Furyl)benzoic acid (****48m****)*. 4-Iodobenzoic acid (**62**, 1.20 g, 4.85 mmol, 1.0 eq) and Na_2_CO_3_ (1.54 g, 14.6 mmol, 3.0 eq) were dissolved in a 4:1 mixture of 1,4-dioxane and water (20 mL) under argon atmosphere and degassed. A catalytic amount of Pd(PPh_3_)_4_ (280 mg, 242 μmol, 0.05 eq) and 2-furylboronic acid (**63a**, 624 mg, 5.58 mmol, 1.15 eq) were added. The mixture was stirred under reflux for 4 h. After cooling to room temperature, the mixture was acidified by addition of 5% aqueous hydrochloric acid and extracted three times with EtOAc. The combined organic layers were dried over Na_2_SO_4_, and the solvent was evaporated under reduced pressure. The title compound was isolated as a brown solid (821 mg, 90%) after column chromatography on silica using 25% EtOAc in hexane + 2% HOAc as mobile phase. ^1^H-NMR (300 MHz, CDCl_3_): *δ* = 6.65 (dd, ^*3*^*J* = 3.4, 1.8 Hz, 1H), 7.13 (dd, ^*3*^*J* = 3.4 Hz, ^*4*^*J* = 0.6 Hz, 1H), 7.78 - 7.86 (m, 3H), 7.90 (ddd, ^*3*^*J* = 8.5 Hz, ^*4*^*J* = 1.8, 1.8 Hz, 2H), 12.92 (br s, 1H) ppm. ^13^C-NMR (75 MHz, CDCl_3_): *δ* = 108.2, 112.4, 123.2, 129.2, 130.0, 134.0, 144.1, 152.1, 166.9 ppm.

*Ethyl 3-(3-[4-{tert-butyl}-2-{4-(2-furyl)}benzamido]phenyl)propanoate (****50aj****)*. A mixture of **48m** (576 mg, 3.06 mmol, 1.1 eq), EDC·HCl (694 mg, 3.62 mmol, 1.3 eq), and 4-DMAP (68 mg, 0.56 mmol, 0.2 eq) was dissolved in CHCl_3_ (15 mL). Then a solution of **47** **h** (1.03 g, 2.78 mmol, 1.0 eq) in CHCl_3_ (10 mL) was added slowly and the mixture was stirred under reflux for 2 h. After cooling to room temperature, water was added and the mixture was extracted three times using CH_2_Cl_2_. The organic layers were dried over Na_2_SO_4_, and the solvent was evaporated. The product was obtained as a beige solid (1.19 g, 79%) from column chromatography on silica using 5% EtOAc in toluene + 2% HOAc as mobile phase. ^1^H-NMR (300 MHz, CDCl_3_): *δ* = 1.20 (s, 9H), 1.25 (t, ^*3*^*J* = 7.1 Hz, 3H), 2.64 (t, ^*3*^*J* = 7.8 Hz, 2H), 2.97 (t, ^*3*^*J* = 7.8 Hz, 2H), 4.15 (q, ^*3*^*J* = 7.1 Hz, 2H), 6.51 (dd, ^*3*^*J* = 3.3 Hz, ^*4*^*J* = 1.8 Hz, 1H), 6.80 (d, ^*3*^*J* = 3.3 Hz, 1H), 6.92 (dd, ^*3*^*J* = 8.3 Hz, ^*4*^*J* = 1.8 Hz, 1H), 7.02 (d, ^*3*^*J* = 7.6 Hz, 1H), 7.32 (dd, ^*3*^*J* = 7.8 Hz, 1H), 7.48 - 7.57 (m, 2H), 7.58 - 7.68 (m, 2H), 7.80 (d, ^*3*^*J* = 8.4 Hz, 2H), 8.03 (d, ^*3*^*J* = 8.5 Hz, 2H), 8.76 (d, ^*4*^*J* = 1.7 Hz, 1H), 8.88 (s, 1H), 11.80 (s, 1H) ppm. ^13^C-NMR (75 MHz, CDCl_3_): *δ* = 14.3, 30.9, 31.1, 35.2, 36.0, 60.6, 107.1, 112.1, 118.5, 118.71, 118.74, 120.45, 120.51, 123.9, 124.7, 127.4, 128.1, 129.3, 133.1, 134.1, 138.4, 139.7, 141.8, 143.1, 153.1, 156.8, 165.4, 167.9, 173.0 ppm.

*4-(tert-Butyl)-1-methyl-2-nitrobenzene (****60****)*. To a solution of 4-(*tert*-butyl)-toluene (5.00 mL, 29.0 mmol, 1.0 eq) in acetic anhydride (50 mL) was dropped an ice-cold mixture of concentrated nitric acid (6.63 mL), glacial acetic acid (7.13 mL), and concentrated H_2_SO_4_ (7.96 mL) at 0 °C. After 2 h, the mixture was poured onto an ice bath and then extracted three times with EtOAc. The combined organic layers were dried over Na_2_SO_4_, the solvent was removed under reduced pressure, and the title compound was obtained as a yellow liquid (3.51 g, 63%) after column chromatography on silica using 2.5% EtOAc in hexane as mobile phase. ^1^H-NMR (500 MHz, CDCl_3_): *δ* = 1.34 (s, 9H), 2.56 (s, 3H), 7.26 (d, ^*3*^*J* = 8.0 Hz, 1H), 7.52 (dd, ^*3*^*J* = 8.0 Hz, ^*4*^*J* = 2.0 Hz, 1H), 7.97 (d, ^*4*^*J* = 2.0 Hz, 1H) ppm. ^13^C-NMR (126 MHz, CDCl_3_): *δ* = 20.1, 31.2, 34.8, 121.6, 130.4, 130.7, 132.6, 149.2, 150.8 ppm.

Synthesis and analytical characterization of **6**, **7**, **9**, **12**–**40**, and their precursors are described in Supplementary Methods.

### Reporter gene assays

#### Plasmids

For the hybrid reporter gene assays, the previously reported Gal4-fusion receptor plasmids pFA-CMV-hPPARα-LBD^29^, pFA-CMV-hPPARδ-LBD^29^, pFA-CMV-hPPARγ-LBD^29^, pFA-CMV-hLXRα-LBD^30^, pFA-CMV-hLXRβ-LBD^30^, pFA-CMV-hRXRα-LBD^31^, pFA-CMV-hRXRβ-LBD^31^, pFA-CMV-hRXRγ-LBD^31^, pFA-CMV-hRARα-LBD^31^, pFA-CMV-hRARβ-LBD^31^, pFA-CMV-hRARγ-LBD^31^, pFA-CMV-hVDR-LBD^31^, and pFA-CMV-hCAR-LBD^31^ coding for the hinge region and LBD of the canonical isoform of the respective nuclear receptor were used. pFR-Luc (Stratagene) was used as reporter plasmid for Gal4 hybrid reporter gene assays. pcDNA3-hFXR^32^ and pSG5-hRXR^33^ served for receptor overexpression in the full-length FXR assay and pGL3basic (Promega Corporation, Fitchburg, WI, USA) with a shortened construct of the BSEP promoter cloned into the SacI/NheI cleavage site in front of the luciferase gene^34^ was used as reporter. PPRE-pGL3^35^ contains the human full-length PPRE (cloned into the SacI/NheI cleavage site in front of the luciferase gene) and was used as reporter plasmid for the full-length PPRE reporter gene assay. pRL-SV40 (Promega) was used in all assays for normalization of transfection efficiency and cell growth.

#### Assay procedures

HEK293T cells (German Collection of Microorganisms and Cell Cultures, DSMZ) were used for the Gal4 hybrid reporter gene assays and the PPRE assay. HEK293T cells were grown in Dulbecco’s modified Eagle’s medium (DMEM) high glucose, supplemented with 10% fetal serum bovine (FCS), sodium pyruvate (1 mM), penicillin (100 U/mL), and streptomycin (100 μg/mL) at 37 °C and 5% CO_2_. The day before transfection, HEK293T cells were seeded in 96-well plates (3 × 10^4^ cells/well). Before transfection, medium was changed to Opti-MEM without supplements. Transient transfection was carried out using Lipofectamine LTX reagent (Invitrogen) according to the manufacturer’s protocol with pFR-Luc (Stratagene), pRL-SV40 (Promega), and the corresponding Gal4-fusion nuclear receptor plasmid. Five hours after transfection, medium was changed to Opti-MEM supplemented with penicillin (100 U/mL), streptomycin (100 μg/mL), and now additionally containing 0.1% DMSO and the respective test compound or 0.1% DMSO alone as untreated control. Each concentration was tested in duplicates, and each experiment was repeated in at least three biologically independent repeats. Following overnight (12–14 h) incubation with the test compounds, cells were assayed for luciferase activity using Dual-Glo™ Luciferase Assay System (Promega) according to the manufacturer’s protocol. HeLa cells (DSMZ) were used for the full-length FXR assay and were grown in DMEM high glucose supplemented with 10% FCS, sodium pyruvate (1 mM), penicillin (100 U/mL), and streptomycin (100 μg/mL) at 37 °C and 5% CO_2_. Twenty-four hours before transfection, cells were seeded in 96-well plates with a density of 8000 cells per well. Three and a half hours before transfection, medium was changed to DMEM high glucose, supplemented with sodium pyruvate (1 mM), penicillin (100 U/mL), streptomycin (100 μg/mL), and 0.5% charcoal-stripped FCS. Transient transfection of HeLa cells with pcDNA3-hFXR, pSG5-hRXR, pGL3basic-BSEP, and pRL-SV40 was carried out with the calcium phosphate transfection method. Sixteen hours after transfection, medium was changed to DMEM high glucose, supplemented with sodium pyruvate (1 mM), penicillin (100 U/mL), streptomycin (100 μg/mL), and 0.5% charcoal-stripped FCS. Twenty-four hours after transfection, medium was changed to DMEM without phenol red, supplemented with sodium pyruvate (1 mM), penicillin (100 U/mL), streptomycin (100 μg/mL), L glutamine (2 mM), and 0.5% charcoal-stripped FCS, now additionally containing 0.1% DMSO and the respective test compound or 0.1% DMSO alone as untreated control. Each concentration was tested in triplicate wells, and each experiment was repeated in at least three biologically independent experiments. Following 24 h incubation with the test compounds, cells were assayed for luciferase activity using Dual Glo Luciferase Assay System (Promega) according to the manufacturer’s protocol. Luminescence was measured with a Spark 10 M luminometer (Tecan Deutschland GmbH). Normalization of transfection efficiency and cell growth was done by division of firefly luciferase data by renilla luciferase data multiplied by 1000 resulting in relative light units (RLU). Fold activation was obtained by dividing the mean RLU of the tested compound at a respective concentration by the mean RLU of untreated control. Relative activation was obtained by dividing the fold activation of the tested compound at a respective concentration by the fold activation of the respective reference agonist (PPARα: GW7647, 1 µM; PPARγ: rosiglitazone or pioglitazone, 1 µM; PPARδ: L165,041, 1 µM; LXRα/β: T0901317, 1 µM; RXRα/β/γ: bexarotene, 1 µM; RARα/β/γ: tretinoin, 1 µM; VDR: calcitriol, 1 µM; CAR: CITCO, 1 µM; FXR: GW4064, 1 µM). Maximum relative activations on PPARγ refer to the activity of rosiglitazone (1 μM) as the reference agonist. For compounds that were characterized relative to pioglitazone (1 μM) previously, a factor between the activation efficacies of rosiglitazone (1 μM) and pioglitazone (1 μM) was determined to adjust the maximum relative activation values accordingly. EC_50_ and standard error of the mean values were calculated with the mean relative activation values by OriginPro, Version 2020 (OriginLab Corporation, Northampton, MA, USA) fitting a dose–response curve with variable Hill slope (Levenberg–Marquardt algorithm). All assays were validated with the above-mentioned reference agonists, which yielded EC_50_ values in agreement with the literature.

### Quantification of FXR- and PPAR-regulated gene expression in HepG2 or C2C12 cells

HepG2 cells (DSMZ) were grown in DMEM high glucose, supplemented with 10% FCS, 1 mM SP, penicillin (100 U/mL), and streptomycin (100 μg/mL) at 37 °C and 5% CO_2_ and seeded in 6-well plates (2 × 10^6^ per well). After 24 h, medium was changed to Minimal Essential Medium supplemented with 1% charcoal-stripped FCS, penicillin (100 U/mL), streptomycin (100 μg/mL), and 2 mM L-glutamine. After an additional 24 h, cells were incubated with test compound **41** (0.1 µM, 0.3 µM, 1 μM), OCA (1 μM), or elafibranor (1 µM) dissolved in the same medium with 0.1% DMSO or medium with 0.1% DMSO alone as untreated control for 12 h, harvested, washed with cold phosphate-buffered saline (PBS), and then directly used for RNA extraction. C2C12 cells (American Type Culture Collection, ATCC) were grown in DMEM high glucose, supplemented with 10% FCS, 1 mM SP, penicillin (100 U/mL), and streptomycin (100 μg/mL) at 37 °C and 5% CO_2_ and seeded in 6-well plates (1 × 10^6^ per well). Upon reaching confluence, medium was changed to Eagle’s Minimal Essential Medium supplemented with 2% horse serum, penicillin (100 U/mL), streptomycin (100 μg/mL), and 2 mM L-glutamine. Cells were then differentiated in this medium for 5 days before incubation with test compounds **41** (1 μM) or L165,041 (1 μM) dissolved in the same medium with 0.1% DMSO or medium with 0.1% DMSO alone as untreated control for 8 h. Cells were harvested, washed with cold PBS, and then directly used for RNA extraction. Total RNA was extracted from HepG2 cells or C2C12 cells using the total RNA Mini Kit (R6834-02, Omega Bio-Tek, Inc., Norcross, GA, USA). Two micrograms of extracted RNA were then reverse-transcribed into cDNA with the High-Capacity cDNA Reverse Transcription Kit (4368814, Thermo Fischer Scientific, Inc.). FXR- and PPAR-regulated gene expression was then studied by quantitative real-time PCR (qRT-PCR) analysis with a StepOnePlus System (Life Technologies, Carlsbad, CA, USA) using PowerSYBRGreen (Life Technologies; 12.5 μL per well). Each sample was set up in duplicates and repeated in four independent experiments. Data were analyzed by the comparative ΔΔC_T_ method with glyceraldehyde 3-phosphate dehydrogenase (GAPDH) as reference gene. The following primers were used for HepG2 cells (human genes): hGAPDH: 5 ′-ATA TGA TTC CAC CCA TGG CA (fw), 5′-GAT GAT GAC CCT TTT GGC TC (rev); hSHP: 5′-GCT GTC TGG AGT CCT TCT GG (fw), 5′-CCA ATG ATA GGG CGA AAG AAG AG (rev); hCYP7A1: 5′-CAC CTT GAG GAC GGT TCC TA (fw), 5′-CGA TCC AAA GGG CAT GTA GT (rev); hBSEP: 5′-CAT GGT GCA AGA AGT GCT GAG T (fw), 5′-AAG CGA TGA GCA ACT GAA ATG AT (rev); hACOX: 5′-ACT CGC AGC CAG CGT TAT G (fw), 5′-AGG GTC AGC GAT GCC AAA C (rev); hPDK4: 5′-AGA GCC TGA TGG ATT TGG TG (fw), 5′-GCT TGG GTT TCC TGT CTG TG (rev); hCPT1: 5′-ACA GTC GGT GAG GCC TCT TAT (fw), 5′-TCT TGC TGC CTG AAT GTG AGT (rev); hFGF21: 5′-ATG GAT CGC TCC ACT TTG ACC (fw), 5′-GGG CTT CGG ACT GGT AAA CAT (rev). The following primers were used for C2C12 cells (murine genes)^36^: mGAPDH: 5′-CGA CTT CAA CAG CAA CTC CCA CTC TTC C (fw), 5′-TGG GTG GTC CAG GGT TTC TTA CTC CTT (rev); mPCK: 5′-TGT TTA CTG GGA AGG CAT CG (fw), 5′-CAG AAT CTC GAG TTG GGA TGG (rev); mGLUT3: 5′-CGT CCT TGA AGA TTC CTG TTG A (fw), 5′-GTC ACC CAA CTA CGT CCA G (rev); mLPL: 5′-GTC AGG TTC TCT CTT GTA CAG G (fw), 5′-TCT AAC TGC CAC TTC AAC CAC (rev).

### Pilot animal experiment

#### Animals and compound application

Nine male RjOrl:Swiss (CD-1) mice (38–41 g body weight, purchased from Janvier Labs, France) were used for the pilot in vivo study. The animals were housed in a temperature-controlled room (20–24 °C) and maintained in a 12 h light/12 h dark cycle. Food and water were available ad libitum. The in-life phase was performed by the contract research organization Pharmacelsus (Saarbrücken, Germany). All experimental procedures were approved by and conducted in accordance with the regulations of the local Animal Welfare authorities (Landesamt für Gesundheit und Verbraucherschutz, Abteilung Lebensmittel- und Veterinärwesen, Saarbrücken). Six animals received a single oral dose of 10 mg/kg body weight of **41**, and three animals received the vehicle. Water containing 1% hydroxypropyl methylcellulose (HPMC)/Tween 80 (99:1) served as vehicle. All animals behaved normal throughout the study and showed no adverse effects.

#### Tissue sampling

Twelve or 18 hours after test compound application, mice were anesthetized under isoflurane and then sacrificed by cervical dislocation for tissue collection. Complete livers were obtained, immediately snap-frozen, and stored at −80 °C until further evaluation.

#### Quantification of mRNA levels from mouse tissue

Liver tissue samples from mice were used to study the mRNA expression of FXR- and PPAR-regulated genes. To homogenize the tissue samples for qRT-PCR analysis, one-third of each liver was placed on a Falcon Cell Strainer with 40-μm pore size (BD Bioscience, Erembodegem, Belgium) in a 50-mL Falcon tube. Every tissue was rinsed with PBS buffer containing 10% FCS, penicillin (100 U/mL), and streptomycin (100 μg/mL) and pressed through the cell strainer until 5 mL cell suspension had been collected. The samples were centrifuged at 1200 rpm for 10 min at 4 °C. The supernatant was discarded, and the pellets were washed twice with 2 mL fresh cold PBS buffer and centrifuged. After discarding the supernatant, total RNA was extracted using the EZA Total RNA Kit I (Omega Bio-Tek Inc., Norcross, GA, USA) following the Animal Tissue Protocol. Two micrograms per sample of the extracted RNA were reverse-transcribed into cDNA using the High-Capacity cDNA Reverse Transcription Kit (4368814, Thermo Fischer Scientific, Inc.) according to the manufacturer’s protocol. mRNA expression was studied by quantitative real-time PCR analysis with a StepOnePlus System (Applied Biosystems) based on the 2^−ΔCT^ method (Supplementary Fig. 6) or the 2^−ΔΔCT^ method (Fig. 6) using Power SYBR Green PCR Master Mix (Life Technologies). Each biological sample was set up in two technical replicates. GAPDH served as reference gene. PCR primers for the murine genes were: mGAPDH: 5′-CGA CTT CAA CAG CAA CTC CCA CTC TTC C (fw), 5′-TGG GTG GTC CAG GGT TTC TTA CTC CTT (rev); mBSEP: 5′-GCC ATT GCC GAC CAG ATG (fw), 5′-CCT AAA AGG AGC CCA GAC AAA G (rev); mSHP: 5′-GCA GGT CGT CCG ACT ATT CTG TAT (fw), 5′-GCA GTG GCT GTG AGA TGC A (rev); mLPL: 5′-GTC AGG TTC TCT CTT GTA CAG G (fw), 5′-TCT AAC TGC CAC TTC AAC CAC (rev); mPCK: 5′-TGT TTA CTG GGA AGG CAT CG (fw), 5′-CAG AAT CTC GAG TTG GGA TGG (rev); mCRAT: 5′-ATC CCA GTT ACC ATC TTC AGT G (fw), 5′-CTA TTG CCG TTC GAT TCT CCA (rev).

### DIO NASH model in mice

The DIO NASH model was performed by the contract research organization Gubra (Hørsholm, Denmark) on a fee-for-service basis. All animal experiments were conducted in accordance with Gubra’s bioethical guidelines, which are fully compliant to internationally accepted principles for the care and use of laboratory animals. The animals were checked minimum once daily for signs of abnormal behavior, abnormal locomotor activity, ataxia, or clinical signs of disease (lack of grooming, raised fur, signs of pain upon handling, loss of excessive body weight).

#### Animals and treatment

Male C57BL/6JRj mice (purchased from Janvier Labs, France at 5 weeks of age) were used for the study. The animals were housed in a temperature (19–23 °C) and humidity (40–60%) controlled room, and maintained in a 12 h light/12 h dark cycle. Mice received the Gubra AMLN NASH (GAN; D09100310, Research Diet, US; 40% fat (primarily palm oil), 40% carbohydrate (20% fructose), and 2% cholesterol) diet for 46 weeks before start of the treatment. Prior to treatment, all animals underwent liver biopsy for histological conformation of liver disease (steatosis score ≥2 and fibrosis stage ≥1) using the non-alcoholic fatty liver disease activity scoring (NAS) and fibrosis staging system. Randomization and stratification to treatment was performed according to quantitative collagen staining (PSR). GAN DIO-NASH mice (*n* = 16 per group) received treatment (PO) with vehicle, OCA (30 mg/kg), or **41** (10 mg/kg, BID) for 12 weeks. Water containing 1% HPMC/Tween 80 (99:1) served as vehicle. Mice were then sacrificed by cardiac puncture under isoflurane anesthesia for histopathological and biochemical analysis.

#### Evaluation

For pre-biopsies, mice were anesthetized by inhalation anesthesia using isoflurane (2–3%). A small abdominal incision was made in the midline and the left lateral lobe of the liver was exposed. A cone-shaped wedge of liver tissue (approximately 50 mg) was excised from the distal portion of the lobe and fixated in 10% neutral buffered formalin (10%) for histology. The cut surface of the liver was instantly electrocoagulated using bipolar coagulation (ERBE VIO 100 electrosurgical unit). The liver was returned to the abdominal cavity, the abdominal wall was sutured, and the skin was closed with staplers. For post-operative recovery, mice received carprofen (5 mg/kg) administered subcutaneously on operation (OP) day and post-OP days 1 and 2. TG, TC, ALT, and AST levels were determined from blood samples collected at week 4 of treatment in heparinized tubes. Plasma was separated and stored at −80 °C until analysis. TG, TC, ALT, and AST were measured using commercial kits (Roche Diagnostics) on the cobas c 501 autoanalyzer according to the manufacturer’s instructions. After termination by heart puncture, livers were collected and weighed. Specific liver samples and biopsies were dissected and processed. The liver was divided into left lateral lobe, medial lobe, right lateral lobe, and caudal lobe. The left lateral lobe was used for the pre-biopsy (not applicable at termination). The liver post biopsy (~200 mg, <0.7 × 0.5 cm) was cut 4 mm from the pre-biopsy site and with an edge. The tissue was collected in paraformaldehyde. The medial lobe was sectioned and snap-frozen in liquid nitrogen for later analysis. One piece (25 ± 5 mg) was dissected and used for TG/TC analysis. Biopsy tissues were cut at 10 μm on a Cryostat, and the sections were mounted on pre-cooled PEN membrane frame slides (ThermoFisher), quickly transferred to pre-cooled 75% EtOH and stored at −20 °C. Liver samples were fixed in formalin, paraffin embedded, and sections were stained. For H&E staining, the slides were incubated in Mayer’s Hematoxylin (Dako), washed in tap water, stained in Eosin Y solution (Sigma-Aldrich), hydrated, and cover-slipped. For PSR staining, the slides were incubated in Weigert’s iron hematoxylin (Sigma-Aldrich), washed in tap water, stained in PSR (Sigma-Aldrich), and washed twice in acidified water. Excess water was removed by shaking the slides, and the slides were then dehydrated in three changes of 100% ethanol, cleared in xylene, and cover-slipped. For type I collagen (Southern Biotech, Cat. 1310-01), α-SMA (Abcam, Cat. Ab124964), and Galectin-3 analysis (Biolegend, Cat. # 125402), immunohistochemistry was performed using standard procedures. Briefly, after antigen retrieval and blocking of endogenous peroxidase activity, slides were incubated with primary antibody. The primary antibody was detected using a polymeric horseradish peroxidase–linker antibody conjugate. Next, the primary antibody was visualized with 3,3′diaminobenzidine as chromogen. Finally, sections were counterstained in hematoxylin and cover-slipped. Samples were scored for NAS following the criteria outlined in^37^. The total NAS score represents the sum of scores for steatosis, inflammation, and ballooning and ranges from 0 to 8.

Experimental procedures for the multiplex toxicity assay, the microsomal stability assay, and molecular docking are described in Supplementary Methods.

### Reporting summary

Further information on research design is available in the Nature Research Reporting Summary linked to this article.

## Supplementary information

Supplementary Information

Reporting Summary

## Data Availability

The datasets generated and analyzed during the current study are available from the corresponding author on reasonable request.
