# Supplementary material for: A triple farnesoid X receptor and peroxisome proliferator-activated receptor α/δ activator reverses hepatic fibrosis in diet-induced NASH in mice
Source: Commun Chem. 2020 Nov 13;3:174. doi: 10.1038/s42004-020-00411-z (PMC9814779; doi:10.1038/s42004-020-00411-z)
Supplement: Supplementary file 1 — Supplementary Information [file 42004_2020_411_MOESM1_ESM.pdf]

A Triple Farnesoid X Receptor and Peroxisome Proliferator-Activated Receptor α/δ Activator Reverses Hepatic Fibrosis in Diet-Induced NASH in Mice

Pascal Heitel[a], Giuseppe Faudone[a], Moritz Helmstädter[a], Jurema Schmidt[a], Astrid Kaiser[a], Amelie Tjaden[b], Martin Schröder[b], Susanne Müller-Knapp[b], Simone Schierle[a], Julius Pollinger[a], Daniel Merk[a]*

[a] Goethe University Frankfurt, Institute of Pharmaceutical Chemistry, Max-von-Laue-Str. 9, D-60438 Frankfurt, Germany. * merk@pharmchem.uni-frankfurt.de

[b] Structural Genomics Consortium, BMLS, Goethe-University Frankfurt, Max-von-Laue-Str. 15, D-60438 Frankfurt, Germany.

# - Supplementary Information -

Table of Contents

Supplementary Figures 2
Supplementary Tables 10
Supplementary Methods 11
Chemistry 11
General Procedures 11
List of Compounds 16
Analytical Characterization Data of **6**, **7**, **9**, and **12-40** and their precursors 30
Supplementary Methods for in Vitro Characterization 56
Molecular Docking 57
Supplementary References 58

Supplementary Figures

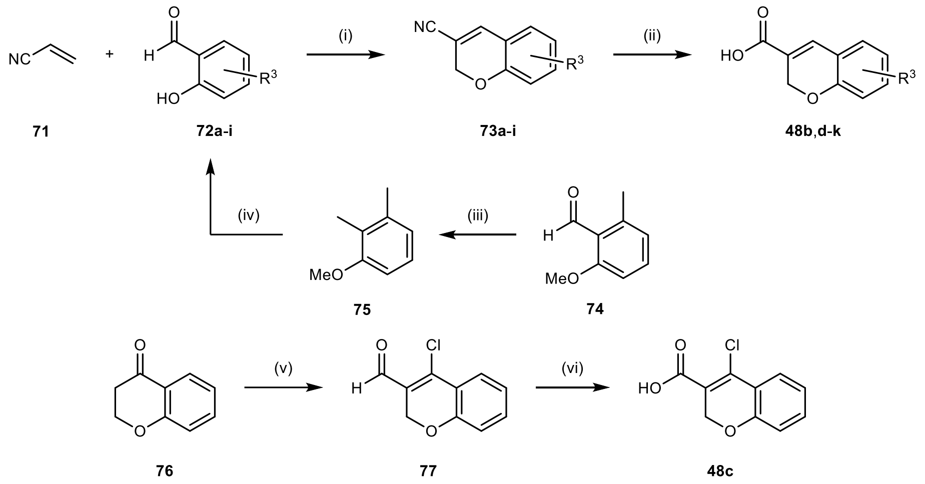

Supplementary Figure 1: Reagents and conditions: (i) DABCO, neat, 85 °C, 35 - 83%. (ii) 10% NaOH (aq.), 110 °C, 45-92%. (iii) $(NH_4)_2S_2O_8$, CH_3CN, H_2O, $CuSO_4$, 90 °C, 38%. (iv) $AlCl_3$, CH_2Cl_2, rt, 69%. (v) $POCl_3$, DMF, 0 °C - 60 °C, 97%. (vi) $AgNO_3$, NaOH, H_2O, EtOH, 85 °C, 45%.

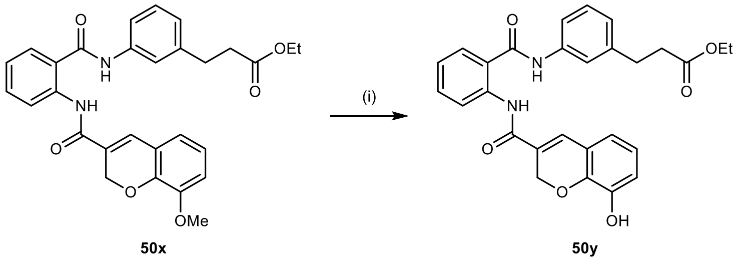

Supplementary Figure 2: Reagents and conditions: (i) BBr_3, CH_2Cl_2, 0 °C - rt, 19%.

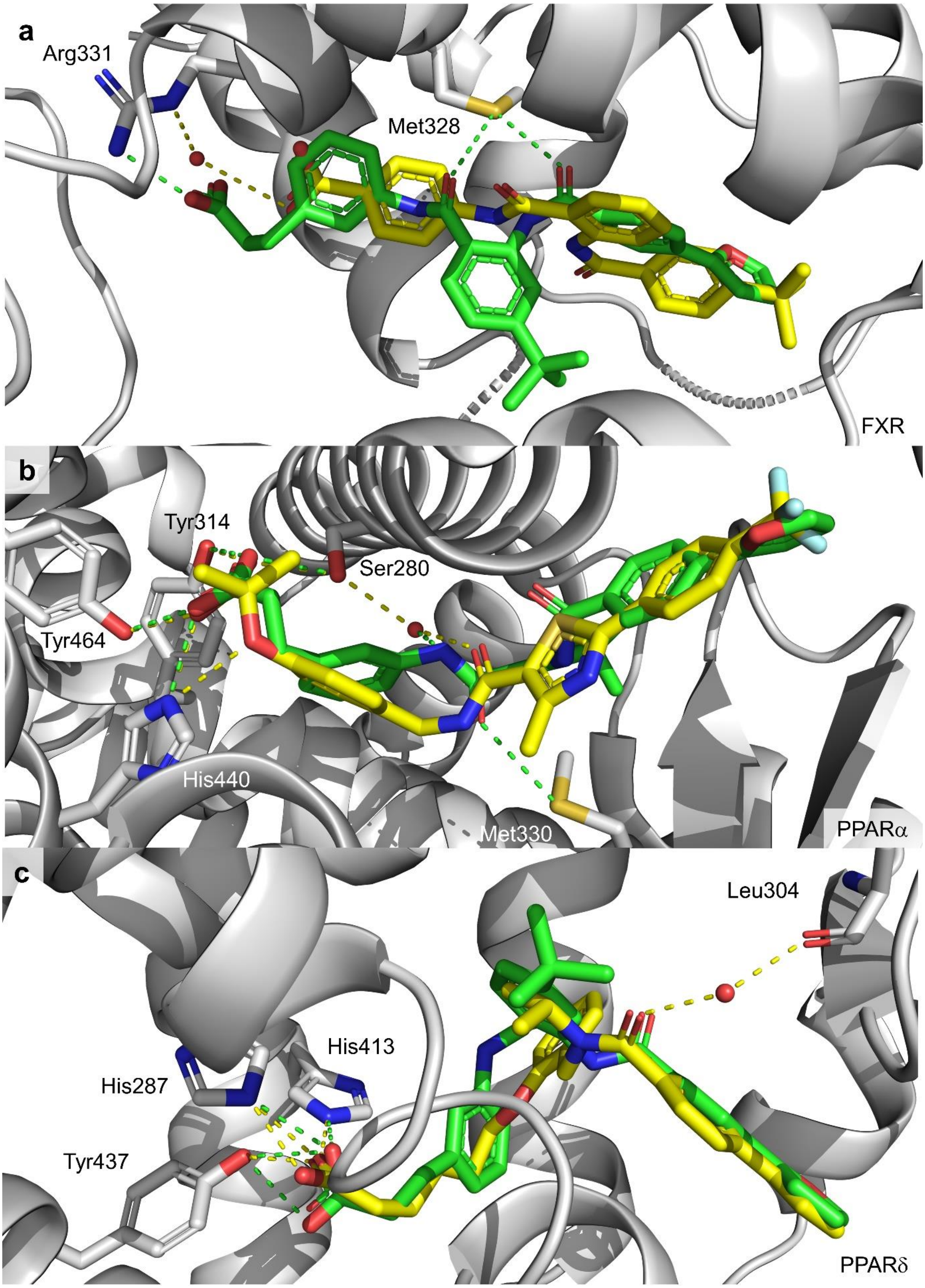

Supplementary Figure 3: Molecular docking of the triple modulator **41** to the ligand binding domains of its nuclear receptor targets (a) FXR (PDB code 4QE8[1]), (b) PPARα (2P54[2]), and (c) PPARδ (5U3R[3]). Docking was performed using AutoDock Vina.[4]

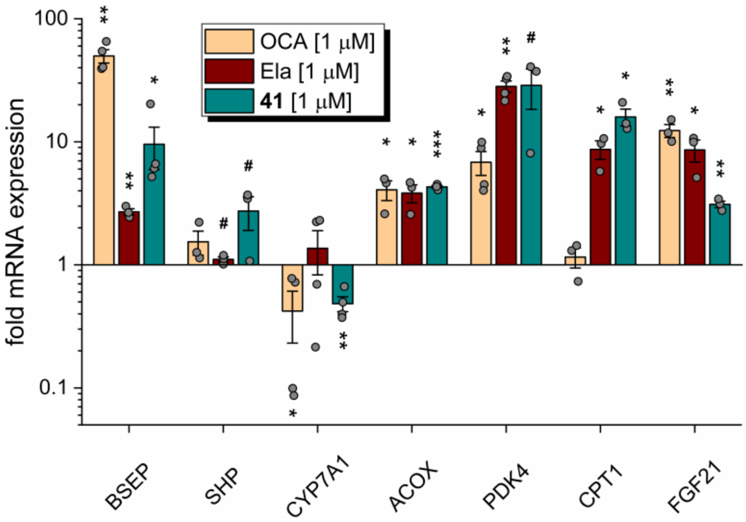

Supplementary Figure 4: Effects of **41** on FXR- and PPAR-regulated gene expression in HepG2 cells. **41** (1 μM) modulated FXR-regulated genes (BSEP, SHP and CYP7A1) and PPAR-regulated (ACOX, PDK4, CPT1, FGF21) genes, thus combining the effects of obeticholic acid (OCA) and elafibranor (Ela). Results are mean ± SEM fold mRNA expression compared to cells treated with 0.1% DMSO, n = 3-4. *** $p < 0.001$, ** $p < 0.01$, * $p < 0.05$, $^{\#}$ $p < 0.1$, t-test vs. DMSO-treated cells.

a

20 40 60 80 100 %

HEK293T

Compound	[µM]	healthy	early apoptosis	late apoptosis	necrosis	lysed	healthy	early apoptosis	late apoptosis	necrosis	lysed	healthy	early apoptosis	late apoptosis	necrosis	lysed
**41**	100	80	12	1	7	1	89	7	1	2	2	85	11	0	2	2
	30	84	12	0	2	2	95	5	0	0	0	94	4	0	0	1
	10	83	13	0	3	2	92	7	0	0	1	95	3	0	0	1
	3	83	11	0	4	2	91	7	0	0	1	95	3	0	1	2
	1	81	13	0	3	3	88	9	0	2	1	95	4	0	0	1
	0.3	86	9	0	3	2	87	9	0	1	2	92	7	0	0	1
	0.1	84	8	0	6	2	88	9	0	0	3	92	6	0	0	2
obeticholic acid	100	83	11	0	4	2	85	9	0	4	3	89	8	0	0	3
	30	82	11	1	5	1	92	6	0	0	2	96	3	0	0	1
	10	85	9	0	2	4	92	6	0	1	2	92	6	0	0	2
	3	81	11	1	5	2	91	7	0	0	1	94	5	0	0	1
	1	83	11	0	3	3	94	6	0	0	0	95	4	0	0	1
	0.3	81	12	0	5	2	91	6	0	1	2	94	5	0	0	1
	0.1	83	9	0	6	1	91	8	0	0	1	92	7	0	0	1
elafibranor	100	83	11	0	4	2	95	3	0	2	0	90	5	0	4	0
	30	86	11	0	2	2	95	5	0	0	0	98	1	0	0	0
	10	82	13	0	2	2	90	7	0	1	3	93	5	0	0	2
	3	85	9	0	3	3	89	8	0	1	2	93	6	0	0	2
	1	81	12	0	4	3	90	8	0	1	2	93	4	0	0	2
	0.3	87	8	0	4	2	91	7	0	0	2	95	4	0	0	1
	0.1	83	10	0	4	4	92	7	0	0	1	94	5	0	0	1
DMSO (0.1%)		83	12	0	3	2	90	8	0	1	2	93	5	0	0	1

U2OS

Compound	[µM]	healthy	early apoptosis	late apoptosis	necrosis	lysed	healthy	early apoptosis	late apoptosis	necrosis	lysed	healthy	early apoptosis	late apoptosis	necrosis	lysed
**41**	100	91	9	0	0	0	93	6	0	0	1	92	4	0	0	4
	30	94	4	0	1	1	96	3	0	0	1	97	3	0	0	0
	10	95	4	0	0	1	96	2	1	0	1	96	2	0	0	2
	3	93	5	1	0	1	94	4	0	0	1	97	3	0	0	1
	1	98	1	0	0	1	97	3	0	0	0	97	2	0	0	1
	0.3	96	3	0	0	1	95	4	0	0	1	95	4	0	0	1
	0.1	97	3	0	0	1	98	2	0	0	0	97	3	0	0	1
obeticholic acid	100	96	4	0	0	0	97	2	0	0	1	93	6	0	0	2
	30	94	4	0	0	1	96	3	0	0	1	96	3	0	0	1
	10	96	2	0	0	1	97	3	0	0	0	98	2	0	0	0
	3	95	4	0	0	0	95	4	0	0	1	96	2	0	0	2
	1	99	1	0	0	0	96	4	0	0	1	97	2	0	0	1
	0.3	96	3	0	0	1	97	2	0	0	1	96	4	0	0	0
	0.1	97	2	0	0	1	98	1	0	0	1	97	1	0	0	1
elafibranor	100	92	7	0	0	1	92	6	0	0	2	84	6	1	1	9
	30	95	3	1	0	1	94	4	0	0	2	92	6	0	0	2
	10	95	4	0	0	1	94	3	0	0	3	93	5	0	0	2
	3	93	5	0	0	1	96	2	0	0	2	98	1	0	0	1
	1	94	4	1	0	1	95	5	0	0	0	96	3	0	0	1
	0.3	97	2	0	0	1	96	4	0	0	1	97	2	0	0	1
	0.1	96	3	0	0	1	96	3	0	0	1	95	5	0	0	0
DMSO (0.1%)		96	3	0	0	1	96	3	0	0	1	96	3	0	0	1

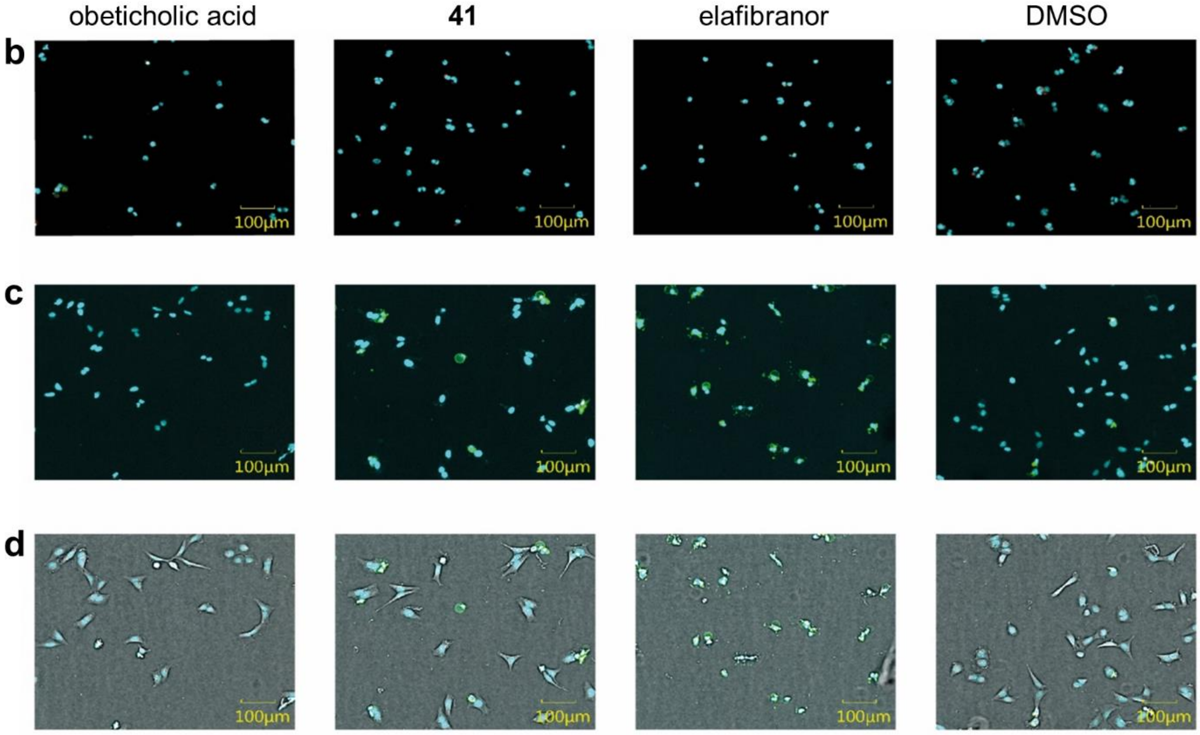

Supplementary Figure 5: Multiplex high-content life-cell cytotoxicity screen. (a) Fraction of healthy, early apoptotic, late apoptotic, necrotic and lysed cells after 2 h, 12 h and 24 h exposure of HEK293T or U2OS cells to respective compound (**41**, obeticholic acid, elafibranor). (b-d) Fluorescence confocal image of stained HEK293T (b) or U2OS (c) cells and brightfield image of stained U2OS cells (d) after 24 h exposure to 100 µM of the respective compound (obeticholic acid, **41**, elafibranor) compared to 0.1% DMSO control.

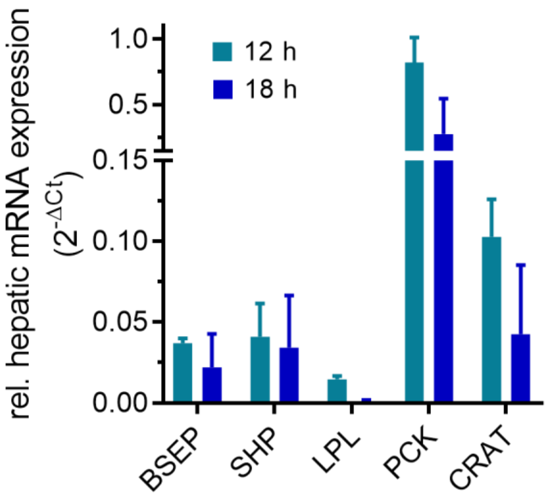

Supplementary Figure 6: Relative hepatic mRNA expression of FXR- and PPAR-regulated genes in mice 12 h and 18 h after receiving a single oral dose of 10 mg/kg of **41**. mRNA levels are shown as $2^{-\Delta Ct}$ with GAPDH as reference gene. Data are the mean ± SEM; n = 3.

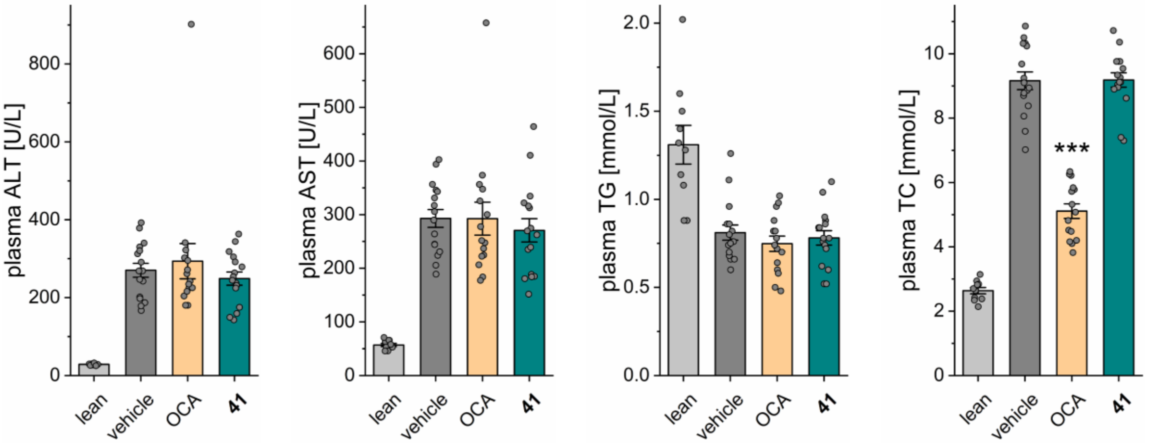

Supplementary Figure 7: Characterization of **41** in a diet-induced obese (DIO) NASH model in mice. Plasma parameters at week 4 of the treatment phase. Results are mean ± SEM; n = 10 (lean chow), 15 (OCA), 16 (vehicle, **41**). ALT – alanine transaminase, AST – aspartate transaminase, TG – total triglycerides, TC – total cholesterol. *** $p < 0.001$ vs. vehicle (Dunnett's test one-factor linear model).

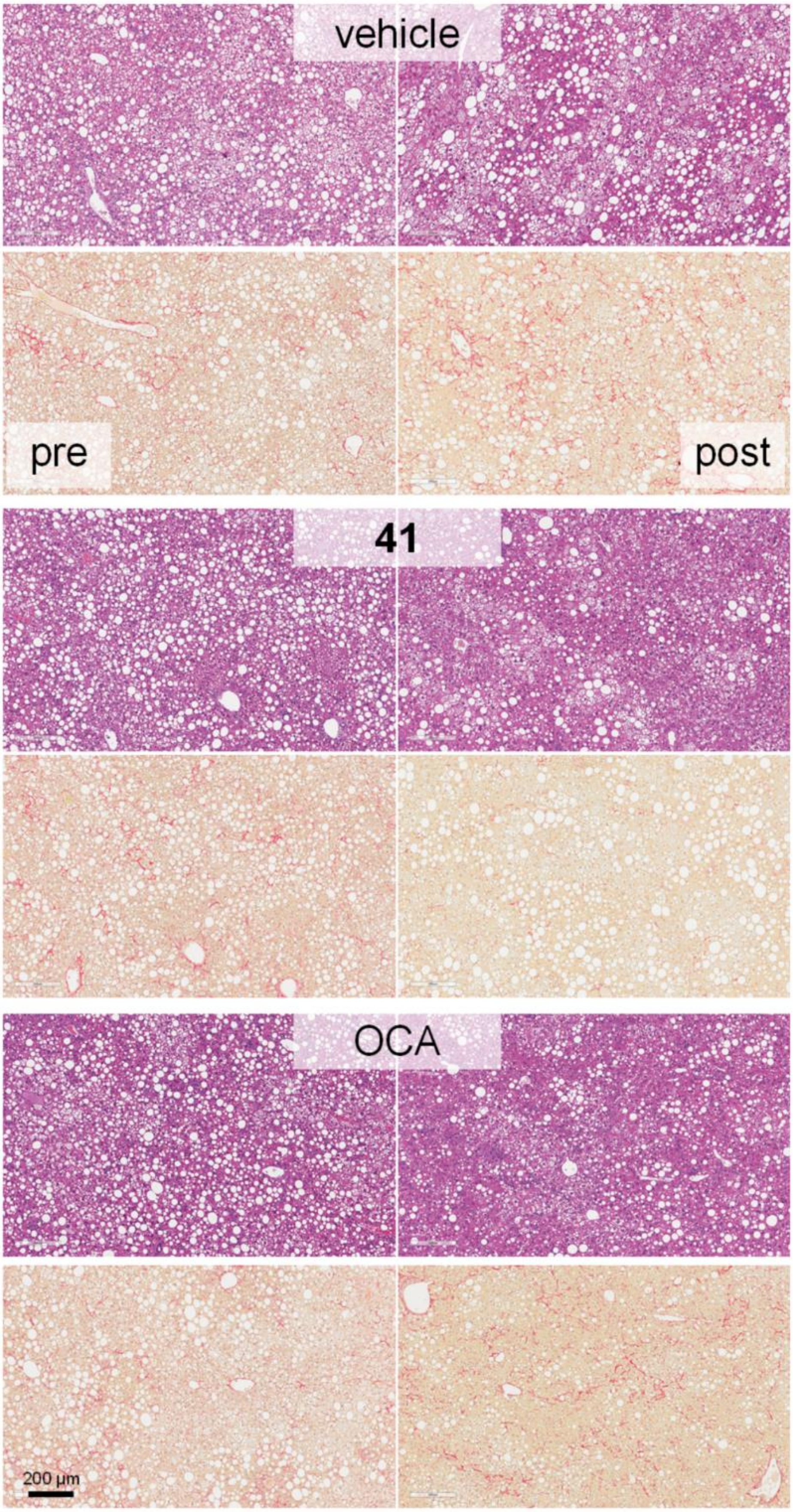

Supplementary Figure 8: Representative immune histochemistry images from DIO NASH mouse liver biopsies before (left images) and after (right images) the treatment phase with the indicated treatment. Upper images show hematoxylin & eosin (H&E) staining, lower images show picrosirius red (PSR) staining. Each set of images was taken from biopsies of the same animal.

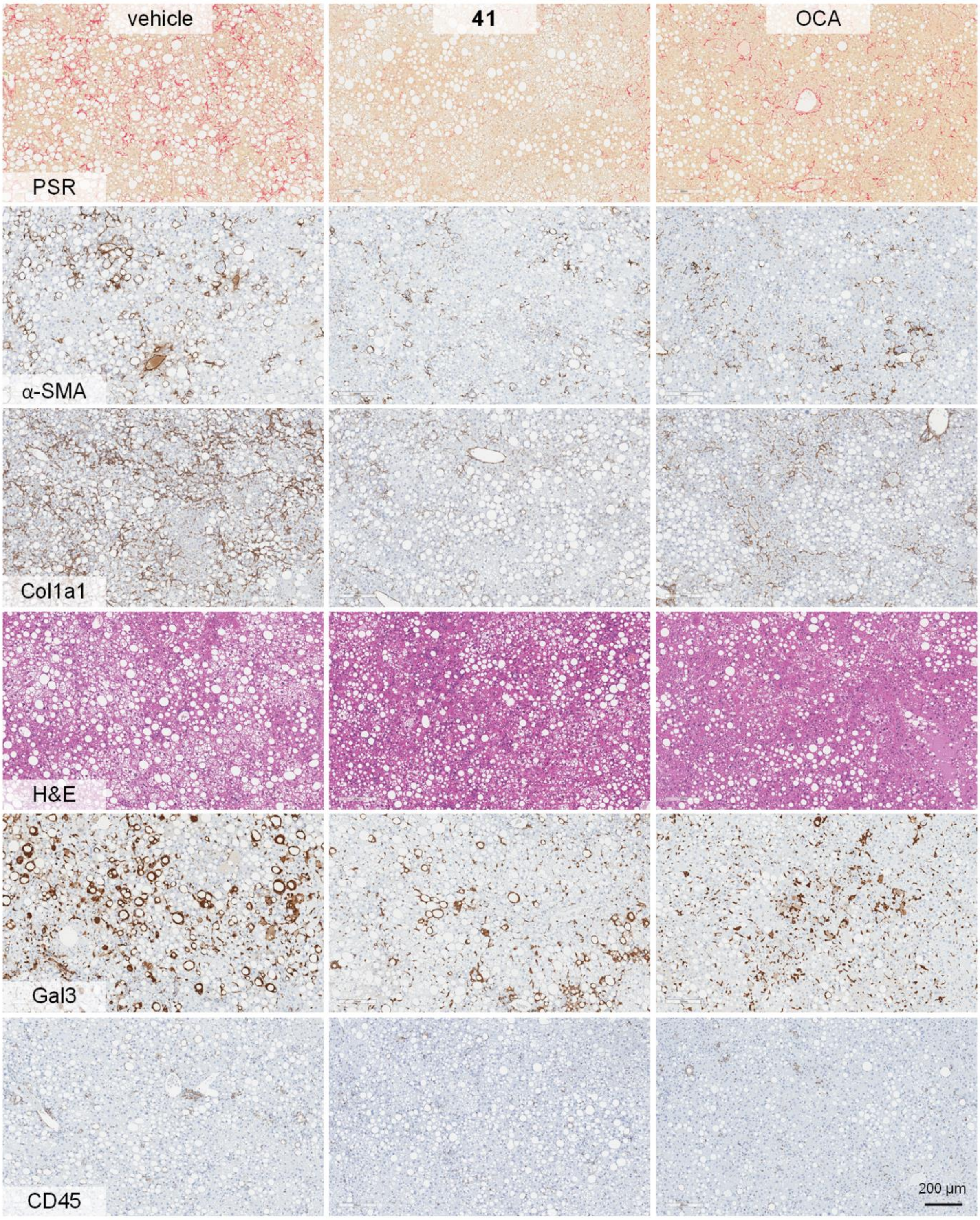

Supplementary Figure 9: Representative immune histochemistry images, set 1.

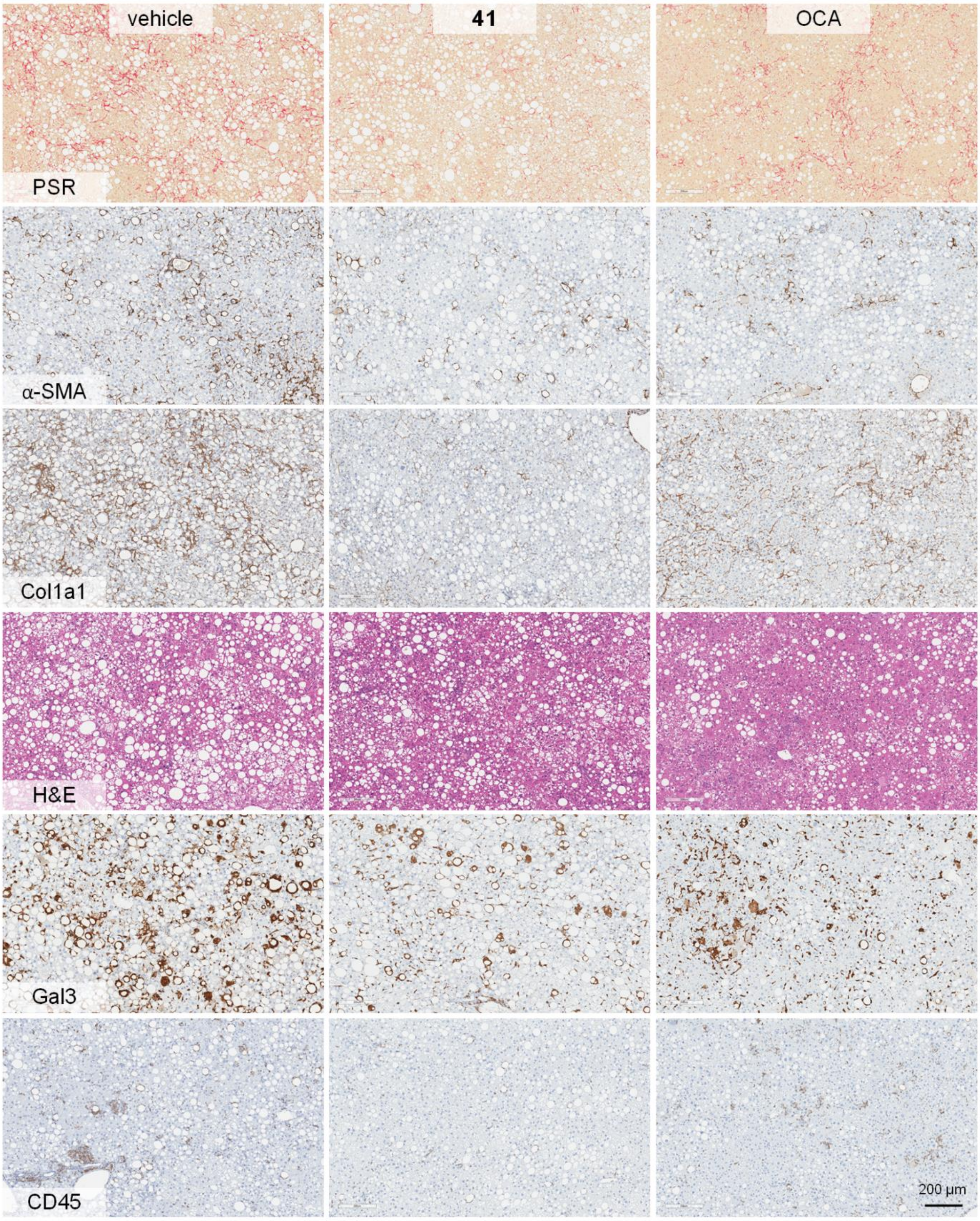

Supplementary Figure 10: Representative immune histochemistry images, set 2.

Supplementary Tables

Supplementary Table 1: Structural optimization of chromene derivatives. Results are expressed as the mean ± SEM; n ≥ 2. Maximum relative activation (max. rel. act.) refers to the activity of reference compounds GW7647 (PPARα), rosiglitazone (PPARγ), L165,041 (PPARδ) at 1 µM and GW4064 (FXR) at 3 µM. i.a. – inactive at 10 µM.

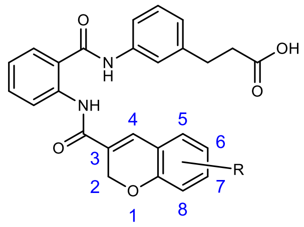

ID	R	PPARα EC_{50} [µM] (max. rel. act.)	PPARδ EC_{50} [µM] (max. rel. act.)	PPARγ EC_{50} [µM] (max. rel. act.)	FXR EC_{50} [µM] (max. rel. act.)
**S1**	-	5.2 ± 0.8 (26 ± 3%)	1.2 ± 0.1 (26 ± 1%)	7.7 ± 0.6 (6.6 ± 0.2%)	4.1 ± 0.8 (14 ± 1%)
**S2**	4-Cl	0.46 ± 0.03 (31 ± 1%)	0.42 ± 0.01 (31 ± 1%)	2.3 ± 0.1 (9.0 ± 0.1%)	11 ± 1 (16 ± 1%)
**S3**	5-CH_3	0.83 ± 0.10 (24 ± 1%)	0.67 ± 0.04 (27 ± 1%)	8.0 ± 0.8 (14 ± 1%)	4.3 ± 0.3 (11 ± 1%)
**S4**	6-CH_3	0.84 ± 0.10 (29 ± 1%)	1.5 ± 0.2 (16 ± 1%)	5.0 ± 1.6 (36 ± 8%)	5.4 ± 0.1 (12 ± 1%)
**S5**	6-F	1.8 ± 0.6 (20 ± 1%)	2.6 ± 0.8 (20 ± 3%)	4.6 ± 0.8 (17 ± 1%)	i.a.
**S6**	6-Cl	3.1 ± 0.4 (27 ± 2%)	1.6 ± 0.5 (14 ± 2%)	3.0 ± 0.5 (5.1 ± 0.3%)	i.a.
**S7**	7-CH_3	3.4 ± 0.1 (38 ± 1%)	1.1 ± 0.1 (30 ± 1%)	5.0 ± 1.6 (7.9 ± 1.4%)	i.a.
**S8**	8-CH_3	3.5 ± 0.2 (29 ± 1%)	1.2 ± 0.4 (18 ± 2%)	3.1 ± 0.2 (9.3 ± 0.5%)	i.a.
**S9**	8-Cl	2.3 ± 0.1 (35 ± 1%)	0.91 ± 0.36 (16 ± 2%)	2.4 ± 0.2 (7.6 ± 0.2%)	i.a.
**S10**	8-OCH_3	6.6 ± 1.8 (26 ± 5%)	5.2 ± 0.2 (30 ± 1%)	23 ± 2 (8.6 ± 0.6%)	2.8 ± 0.3 (14 ± 1%)
**S11**	8-OH	> 30	> 30	7.9 ± 1.2 (6.3 ± 0.5%)	i.a.

Supplementary Methods

Chemistry

General

All chemicals and solvents were obtained from commercial sources in reagent grade and used without further purification. TLC was performed using TLC-plates (silica gel 60 F254, 0.2 mm, Merck or Alugram Xtra Sil G/UV 0.2 mm, Macherey-Nagel) with detection under UV-light (254 nm and 366 nm). Preparative column chromatography was performed using Silicagel 60 (Macherey-Nagel) and solvents of technical grade. Reactions with air- or moisture-sensitive compounds were carried out under argon atmosphere and in anhydrous solvents. NMR spectra were recorded on Bruker AM 250 XP, AV 300, AV 400, AV 500 spectrometers (Bruker Corporation, Billerica, MA, USA). Chemical shifts (δ) are reported in ppm relative to TMS, coupling constants (*J*) in Hz. Multiplicity of signals is indicated as s for singulet, d for doublet, t for triplet, q for quartet, and m for multiplet. Aromatic signals resembling a triplet that stem from protons with two unequal neighbors but similar or equal coupling constants, are denoted as doublets of doublet (dd). Mass spectra were obtained on a VG Platform II (Thermo Fischer Scientific, Inc., Waltham, MA, USA) using electrospray ionization (ESI). High resolution mass spectra were recorded on a MALDI LTQ ORBITRAP XL instrument (Thermo Fisher Scientific) or on a Bruker maXis ESI-Qq-TOF-MS instrument (Bruker). Compound purity was analyzed on a Varian ProStar HPLC (SpectraLab Scientific Inc., Markham, ON, Canada) equipped with a MultoHigh100 Phenyl-5 µ 240+4 mm column (CS-Chromatographie Service GmbH, Langerwehe, Germany) using a gradient (H_2O/MeOH 80:20 + 0.1% formic acid isocratic for 5 min to MeOH + 0.1% formic acid after additional 45 min and MeOH + 0.1% formic acid for additional 10 min) at a flow rate of 1 mL/min and UV-detection at 245 nm and 280 nm. All final compounds for biological evaluation had a purity > 95% according to the AUC at 245 nm and 280 nm UV detection.

General Procedures

(A) Esterification

The corresponding acid (1.0 eq) was dissolved in a 20:1 (v/v) mixture of EtOH and concentrated H_2SO_4 (c = 0.5 M) and heated to reflux for two hours. Then, the solution was neutralized with Na_2CO_3 and the crude product was extracted three times with CH_2Cl_2. The organic layers were combined, dried over Na_2SO_4 and the solvent was evaporated. If required, the product was further purified by column chromatography on silica.

(B) Amide synthesis

A mixture of the respective carboxylic acid (1.1 eq), EDC·HCl (1.2 eq), and 4-DMAP (0.2 eq) were dissolved in CH_2Cl_2 or $CHCl_3$ (c = 0.2 M) under argon atmosphere. Then, a solution of the corresponding amine (1.0 eq) in CH_2Cl_2 or $CHCl_3$ (c = 0.4 M) was added slowly and the mixture was heated to reflux for six hours. After cooling to room temperature, the solvent was evaporated and the product was purified by column chromatography on silica.

(C) Amide synthesis II

If the acid chloride was not available commercially, a solution of the corresponding acid (1.3 eq) in $SOCl_2$ (c = 1.0 M) was heated to reflux for two hours. Excessive $SOCl_2$ was removed by distillation and

the acid chloride was washed twice with toluene and dried. Subsequently, the acid chloride was dissolved in THF (c = 0.6 M) under argon atmosphere. Pyridine (3.0 eq) and a solution of the corresponding amine (1.0 eq) in THF (c = 1 M) were added and the mixture was heated to reflux for one hour. After cooling to room temperature, 5% HCl was added and the product was extracted three times with EtOAc. The combined organic layers were dried over Na_2SO_4 and the solvent removed by evaporation under reduced pressure. The product was purified by column chromatography on silica.

(D) Reduction of nitro group

The nitro compound (1.0 eq) was dissolved in EtOAc (c = 0.05 M). Palladium (10%) on charcoal (0.1 eq) was added and stirred under hydrogen atmosphere overnight. On the next day, the charcoal was filtered off on celite or silica, the solvent was evaporated. If necessary, the product was further purified by column chromatography on silica.

(E) Reduction of nitro group II

The nitroarene (1.0 eq) was dissolved in a 2:1 mixture of EtOAc and acetic acid (c = 0.05 M). Then, iron turnings (5.0 eq) were added. The resulting suspension was stirred at 50 °C. After TLC had indicated complete reaction, the suspension was filtered over celite. Water was added to the filtrate and the crude product was extracted three times with EtOAc. The combined organic layers were dried over Na_2SO_4 and subsequently, the solvent was removed under reduced pressure. The pure product was obtained after column chromatography on silica.

(F) Reduction of nitro group III

The nitroarene (1.0 eq) was dissolved in THF/10% HCl (c = 0.1 M). Then, tin(II) chloride (4.2 eq) was added and the resulting suspension was stirred at 50 °C. After TLC had indicated complete reaction, the suspension was filtered over celite. Water was added to the filtrate and the crude product was extracted three times with EtOAc. The combined organic layers were dried over Na_2SO_4 and subsequently, the solvent was removed under reduced pressure. The pure product was obtained after column chromatography on silica.

(G) Anthranilamide preparation

The amine (1.0 eq) was dissolved in boiling EtOH (c = 0.2 M) and isatoic anhydride (2.0 eq) was added. The mixture was heated for 1.5 hours under reflux, cooled to room temperature and filtered. Subsequently, the solvent was evaporated under reduced pressure and the product was isolated by column chromatography on silica.

(H) Alkaline hydrolysis

An aqueous solution of LiOH (6.0 eq, c = 1.0 M) was added to a solution of the respective ester (1.0 eq) in THF (c = 0.05 M). The resulting mixture was either heated to 50 °C or stirred at ambient temperature until TLC indicated complete conversion. Subsequently, the solution was acidified with 5% HCl and extracted three times with EtOAc. If necessary, further purification was achieved by column chromatography on silica.

(I) Ether and secondary amine synthesis

The corresponding alcohol or amine (1.0 eq), alkyl bromide (0.5 – 1.4 eq) and K_2CO_3 (1.2 eq) were suspended in DMF (4 mL/mmol) and stirred under argon atmosphere for 16 hours. EtOAc (30 mL) was

added and the organic layer was washed three times with water (30 mL). The organic layer was dried over Na_2SO_4 and the solvent evaporated. Column chromatography on silica afforded the pure product.

(J) Rosenmund-von Braun reaction

Under argon atmosphere, the bromoarene (1.0 eq) and copper(I) cyanide (1.0 eq) were suspended in DMF (0.5 mL/mmol) and heated to 150 °C for one hour. Subsequently, toluene (2.7 mL/mmol) was added and the mixture was heated to reflux for 60 minutes under stirring. After cooling to room temperature, water (50 mL) was added and the mixture was extracted once with EtOAc (50 mL). The organic layer was dried over Na_2SO_4, and the solvent was evaporated under vacuum. Purification by column chromatography afforded the pure product.

(K) Acidic nitrile hydrolysis

55% H_2SO_4 (5 mL) was added to the nitrile (1.0 eq) and the suspension was heated to reflux for 15 hours. After cooling to ambient temperature, the mixture was extracted three times with EtOAc. The combined organic layers were then extracted three times with 1 M NaOH (10 mL). The product was obtained after acidification of the combined aqueous layers with 10% HCl and extraction with EtOAc (3x). The combined organic layers were dried over Na_2SO_4, and the solvent was evaporated under reduced pressure.

(L) Nitration

To a solution of the aromatic compound (1.0 eq) in acetic anhydride or acetic acid (c = 0.5 M) was dropped an ice-cold mixture of concentrated nitric acid (10 eq) and glacial acetic acid (6 eq) with catalytic amounts of concentrated H_2SO_4 at 0 °C. The reaction was monitored by TLC. After complete conversion, the mixture was poured into an ice-bath and then extracted three times with EtOAc. The combined organic layers were dried over Na_2SO_4, the solvent was removed under reduced pressure, and the pure product was obtained after column chromatography on silica.

(M) Oxidation of toluenes to benzoic acids

The respective toluene (1.0 eq) was added to a 1:1 (v/v) mixture of pyridine and water (c = 0.2 M) and heated to 100 °C. A 0.5 M aqueous solution of $KMnO_4$ (5.0 eq) was slowly added to the solution and the mixture was stirred for additional four hours. The resulting suspension was filtered, acidified with 10% HCl, and extracted three times with EtOAc. The combined organic layers were dried over Na_2SO_4, the solvent was removed under reduced pressure. The crude product was purified by column chromatography on silica.

(N) Demethylation

The respective methoxyarene (1.0 eq) was dissolved in CH_2Cl_2 (c = 0.03 M) at 0 °C under argon atmosphere. After careful addition of a 1 M solution of BBr_3 (10 eq), the solution was allowed to warm to ambient temperature and stirred overnight. The reaction was terminated by addition of water followed by 5% HCl. The mixture was extracted three times with EtOAc, the combined organic layers were dried over Na_2SO_4, and the solvent was evaporated under reduced pressure. Column chromatography on silica provided the pure product.

(O) 2H-Chromene synthesis

Chromene-3-carbonitriles were synthesized from the corresponding salicylaldehydes (1.0 eq), which were heated to reflux in acrylonitrile (c = 1 M) in presence of 1,4-diazabicyclo[2.2.0]octane (0.2 eq) for 15 hours under argon atmosphere. After cooling to room temperature, EtOAc and 1 M NaOH (1:1) were added. Phases were separated, the organic layer was dried over Na_2SO_4 and the solvent was evaporated under reduced pressure. Column chromatography provided the pure product.

(P) Alkaline nitrile hydrolysis

Chromene-3-carbonitriles (1.0 eq) were hydrolyzed to the respective carboxylic acids in a 0.2 M suspension of 10% NaOH under reflux for six hours. The obtained solution was then acidified with 10% HCl. If the product precipitated, it was filtered, washed with water and dried under vacuum. In the other case, the mixture was extracted three times with EtOAc, the combined organic layers were dried over Na_2SO_4 and the solvent was evaporated under reduced pressure.

(Q) Oxidation of toluenes to benzaldehydes

To a solution of the toluene derivative (1.0 eq, c = 0.6 M) and $CuSO_4$ (3.0 eq) in acetonitrile/water (1:1), ammonium persulfate (3.0 eq) was added. The mixture was heated to reflux for 30 minutes under vigorous stirring. The mixture was then extracted three times with CH_2Cl_2, the combined organic layers were dried over Na_2SO_4, and the solvent was evaporated under reduced pressure. Further purification by column chromatography on silica provided the product.

(R) Demethylation II

The respective methoxyarene (1.0 eq) was dissolved in CH_2Cl_2 (c = 0.03 M) under argon atmosphere and cooled to 0 °C. After careful addition of $AlCl_3$ (3.0 eq), the solution was allowed to warm to ambient temperature and stirred overnight. The reaction was terminated by addition of water followed by 5% HCl. The mixture was extracted three times with EtOAc, the combined organic layers were dried over Na_2SO_4, and the solvent was evaporated under reduced pressure. Column chromatography on silica provided the pure product.

(S) 4-Chloro-2H-chromene-3-carbaldehyde synthesis

The chromenone (1.0 eq) was dissolved in DMF (2.5 eq) under argon atmosphere and $POCl_3$ (2.0 eq) was added. The mixture was heated to 60 °C for 6 hours. The reaction was quenched with ice-cold water and the mixture was extracted three times with EtOAc. The combined organic layers were dried over Na_2SO_4, and the solvent was evaporated under reduced pressure. The product was used without further purification.

(T) Oxidation of aldehydes to carboxylic acids

Fresh Ag_2O was prepared by adding a solution of $AgNO_3$ (2.2 eq) in 10 mL water dropwisely to a solution of NaOH (4.0 eq) in 10 mL water and 20 mL EtOH. Subsequently, a solution of the respective aldehyde (1.0 eq) in 5 mL EtOH was added and the resulting mixture was stirred for 75 minutes at 85 °C. After cooling to room temperature, the resulting suspension was filtered, and the residue rinsed three times with a 1:1 (v/v) mixture of EtOH and water. The filtrate's EtOH content was evaporated under reduced pressure and the residue was acidified with 5% HCl, and extracted three times with EtOAc. The

combined organic layers were dried over Na_2SO_4, and the solvent was evaporated under reduced pressure. The product was purified by column chromatography on silica.

(U) Suzuki reaction

The iodoarene (1.0 eq) and Na_2CO_3 (3.0 eq) were dissolved in a 4:1 mixture of 1,4-dioxane and water (c = 0.1 M) under argon atmosphere and degassed. A catalytic amount of $Pd(PPh_3)_4$ (0.05 eq) and the corresponding boronic acid (1.2 eq) were added. The mixture was heated to reflux for 2 hours. After cooling to room temperature, the mixture was acidified by addition of 5% HCl, and extracted three times with EtOAc. The combined organic layers were dried over Na_2SO_4, and the solvent was evaporated under reduced pressure. The product was purified by column chromatography on silica.

(V) Tosylation

The respective amine (2.0 eq) was dissolved in water (2 mL) at 0 °C. Then, a solution of K_2CO_3 (2.0 eq) in water (3 mL) was added dropwisely and the mixture was stirred for 15 minutes after complete addition. THF (10 mL), MeOH (3 mL), and *p*-toluenesulfonyl chloride (1.0 eq) were then added (the latter in portions). The mixture was next allowed to warm to room temperature and stirred for another three hours. Water (30 mL) was added and the mixture was extracted twice with EtOAc (30 mL). The combined organic layers were dried over Na_2SO_4, and the solvent was evaporated under reduced pressure. The product was used without further purification.

(W) Heck reaction

The respective iodoarene (1.0 eq), acrolein diethyl acetal (3.0 eq), $Pd(OAc)_2$ (0.03 eq), K_2CO_3 (2.5 eq), tetrabutylammonium acetate (2.0 eq) and KCl (1.0 eq) were suspended in DMF (2 mL) and the mixture was irradiated in a microwave reactor to 90 °C for two hours. 5% HCl (3 mL) and THF (10 mL) were then added and the mixture was stirred for one hour at room temperature. EtOAc (40 mL) was added, phases were separated, the organic layer was washed three times with water (3x 20 mL), and dried over Na_2SO_4. The solvent was evaporated under reduced pressure and the product was purified by column chromatography on silica.

(X) Isoxazole synthesis

The respective acryl aldehyde derivative (1.0 eq), *N*-hydroxyl-*p*-toluenesulfonamide (4.0 eq) and K_2CO_3 (6.0 eq) were dissolved in a mixture of MeOH (12 mL/mmol) and water (8 mL/mmol), and stirred at room temperature until TLC indicated complete consumption of starting material. The solution was then heated to reflux for 21 hours. EtOAc (30 mL) was added, phases were separated, and the organic layer was washed with diluted HCl twice and water (20 mL each) once. The organic layer was dried over Na_2SO_4 and the solvent was removed under reduced pressure. The crude product was purified by column chromatography on silica.

(Y) Bromination

A solution of bromine (1.0 eq) in glacial acetic acid (9 mL/mmol) was added dropwisely to a solution of the ketone (1.0 eq) in glacial acetic acid (5 mL/mmol) and the mixture was stirred for 17 hours. Water (20 mL) and 5% sodium thiosulfate solution (10 mL) were added and the mixture was extracted three times with EtOAc. The combined organic layers were dried over Na_2SO_4 and the solvent was removed under reduced pressure. The product was used without further purification.

(Z) Oxazole synthesis

The bromoacetophenone (1.0 eq) and ammonium formate (3.0 eq) were dissolved in formic acid (5 mL) and DMF (2 mL), and the mixture was heated to reflux for 5 hours. After cooling to ambient temperature, the solution was neutralized with $NaHCO_3$ solution and EtOAc was added (50 mL). Phases were separated and the organic layer was washed twice with water. The organic layer was dried over Na_2SO_4 and the solvent was evaporated. The product was purified by column chromatography on silica.

List of Compounds

List of Compounds. n.i., not isolated.

ID	Structure	Procedure	Starting material A		Starting material B		Yield [%]
**5**	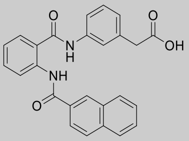	previously published[5]					
**6**	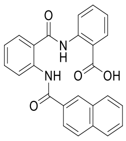	C	**47p**	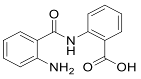	**49a**	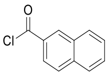	35
**7**	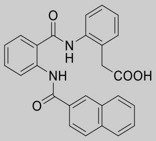	H	**50a**	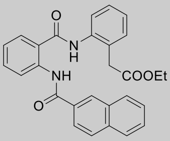			92
**8**	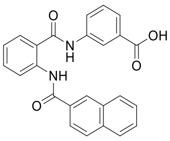	previously published[5]					
**9**	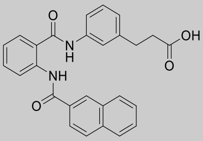	C	**47q**	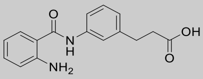	**49a**	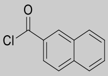	86
**10**	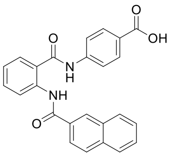	previously published[5]					
**11**	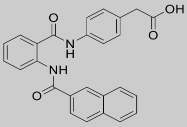	previously published[5]					
**12**	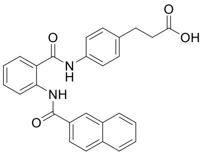	H	**50b**	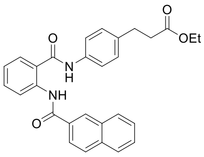			92
**13**	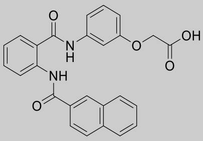	H	**50ak**	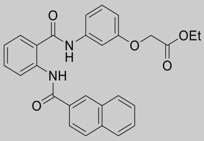			98

ID	Structure	Procedure	Starting material A	Starting material B	Yield [%]
**14**	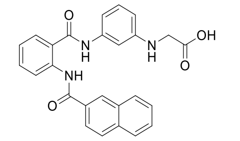	H	**50al** 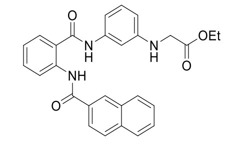		99
**15**	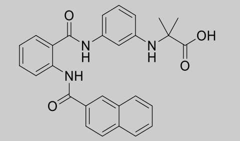	H	**50am** 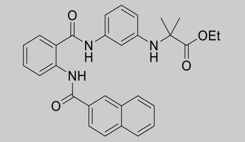		99
**16**	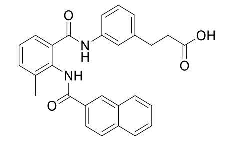	H	**50c** 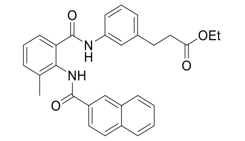		65
**17**	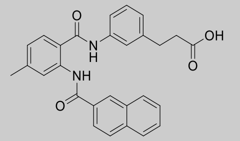	H	**50d** 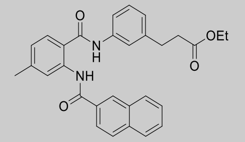		84
**18**	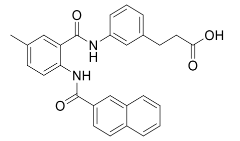	H	**50e** 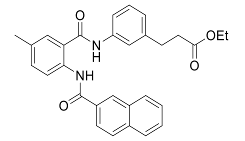		90
**19**	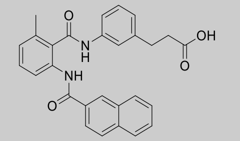	H	**50f** 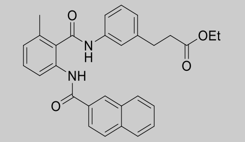		80
**20**	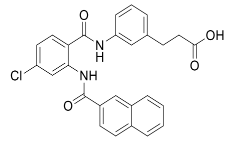	H	**50g** 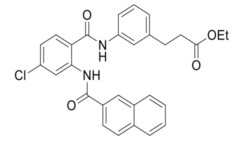		82
**21**	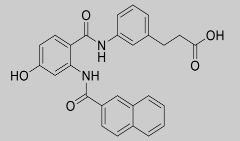	N	**22** 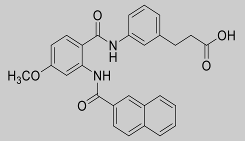		65
**22**	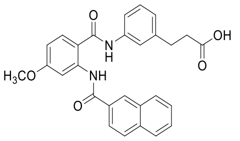	H	**50h** 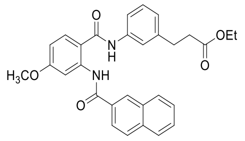		86
**23**	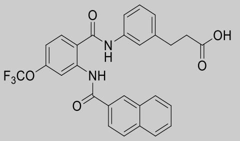	H	**50i** 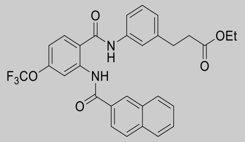		56
**24**	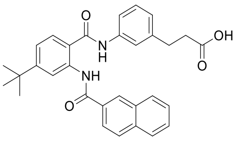	H	**50j** 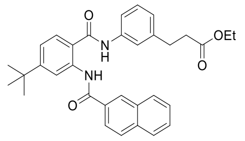		73
**25**	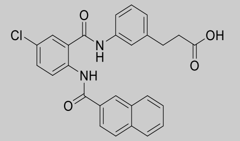	H	**50k** 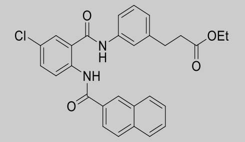		88

ID	Structure	Procedure		Starting material A	Starting material B	Yield [%]
**26**	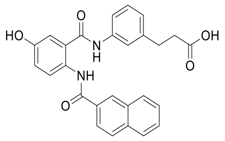	N	**50l**	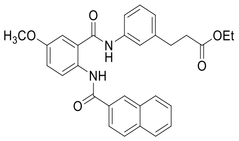		22
**27**	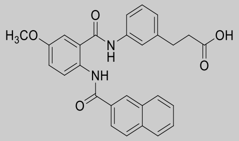	H	**50l**	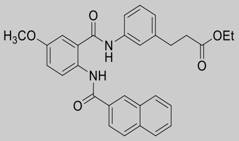		91
**28**	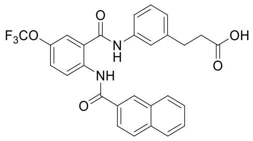	H	**50m**	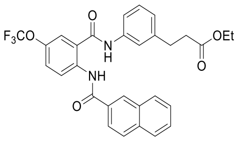		97
**29**	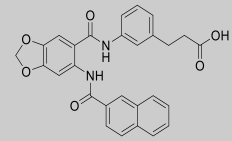	H	**50n**	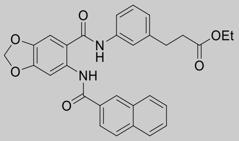		87
**S1**	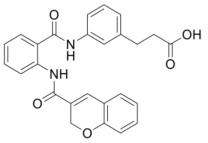	H	**50o**	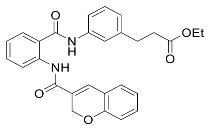		99
**S2**	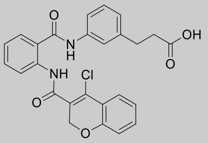	H	**50p**	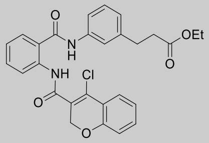		54
**S3**	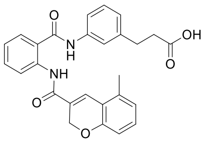	H	**50q**	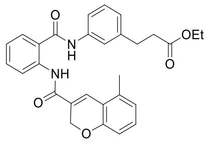		91
**S4**	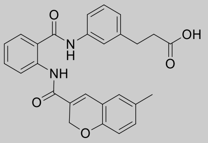	H	**50r**	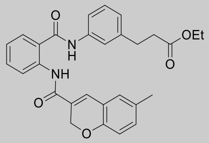		96
**S5**	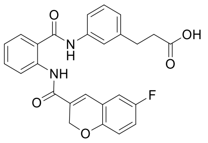	H	**50s**	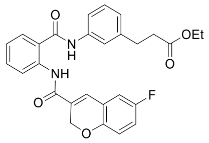		62
**S6**	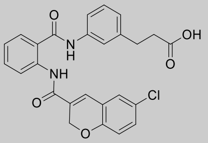	H	**50t**	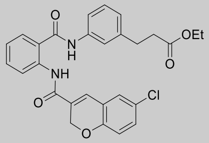		72
**S7**	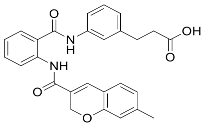	H	**50u**	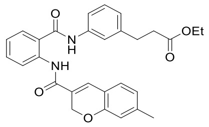		99
**S8**	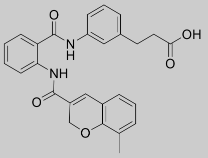	H	**50v**	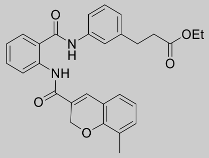		88

ID	Structure	Procedure	Starting material A		Starting material B	Yield [%]
**S9**	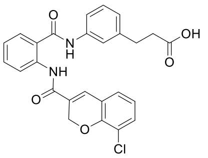	H	**50w**	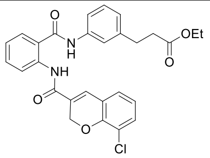		97
**S10**	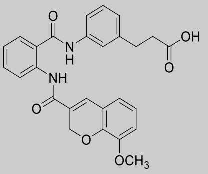	H	**50x**	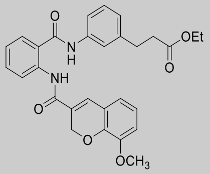		97
**S11**	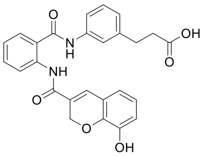	H	**50y**	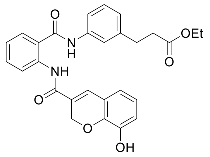		85
**30**	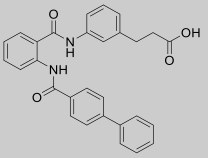	H	**50z**	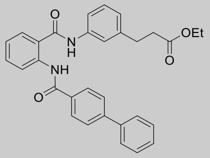		97
**31**	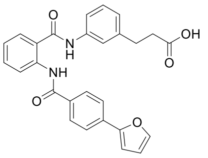	H	**50aa**	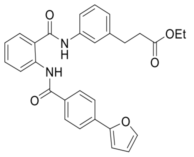		91
**32**	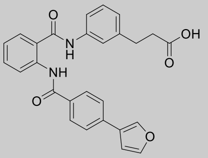	H	**50ab**	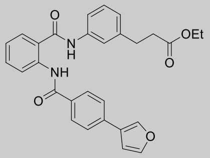		99
**33**	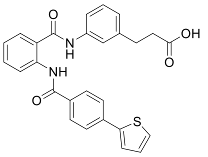	H	**50ac**	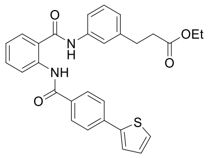		39
**34**	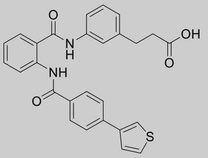	H	**50ad**	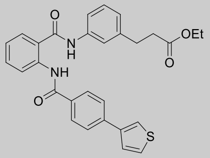		99
**35**	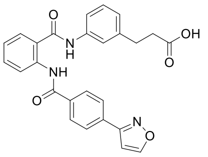	H	**50ae**	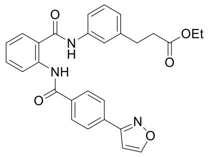		95
**36**	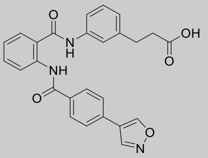	H	**50af**	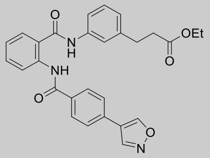		-
**37**	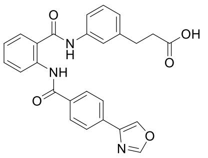	H	**50ag**	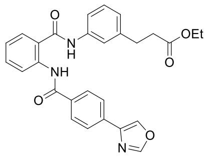		65

ID	Structure	Procedure	Starting material A	Starting material B	Yield [%]
**38**	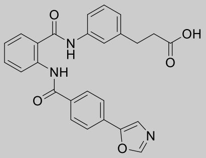	H	**50ah** 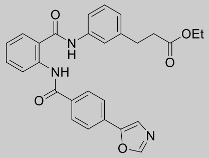		20
**39**	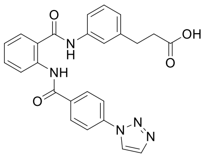	H	**50ai** 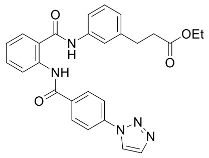		93
**40**	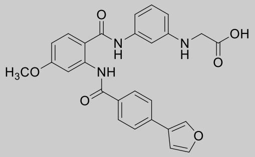	H	**50an** 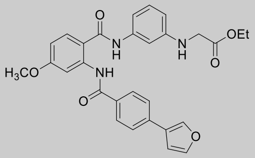		36
**41**	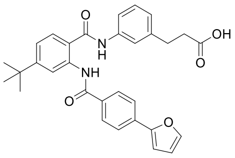	H	**50aj** 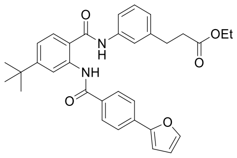		99
**42a**	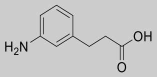	purchased			
**42b**	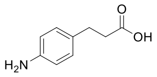	purchased			
**42c**	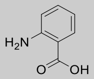	purchased			
**43a**	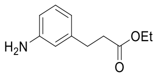	A	**42a** 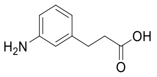		93
**43b**	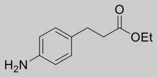	A	**42b** 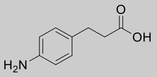		94
**43c**	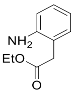	A	**52** 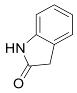		39
**43d**	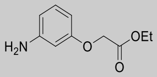	D	**55** 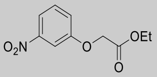		86
**43e**	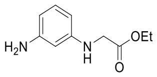	I	**56** 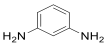	**54a** 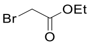	75
**43f**	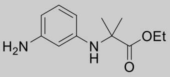	I	**56** 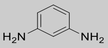	**54b** 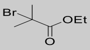	50
**44a**	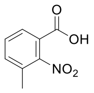	purchased			
**44b**	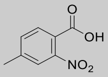	purchased			
**44c**	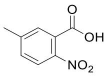	purchased			
**44d**	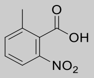	purchased			
**44e**	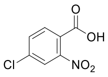	purchased			

ID	Structure	Procedure	Starting material A		Starting material B		Yield [%]
**44f**	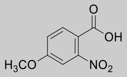	purchased					
**44g**	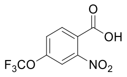	K	**58**	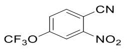			45
**44h**	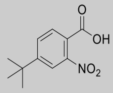	M	**60**	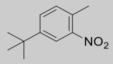			51
**44i**	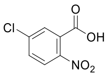	purchased					
**44j**	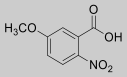	purchased					
**44k**	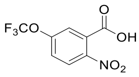	purchased					
**44l**	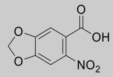	L	**61**	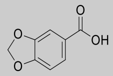			76
**44m**	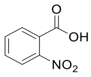	purchased					
**45**	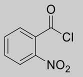	(C)	**44m**	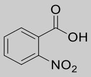			n.i.
**46a**	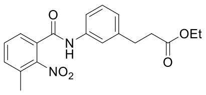	B	**44a**	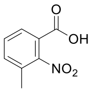	**43a**	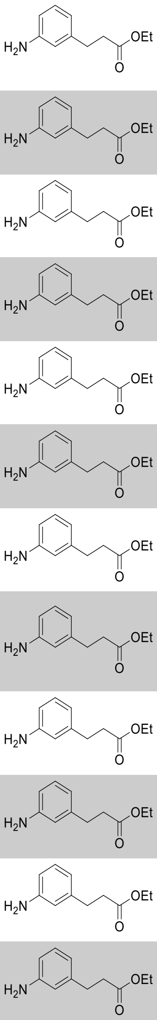	53
**46b**	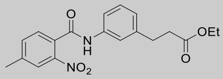	B	**44b**	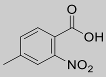	**43a**		66
**46c**	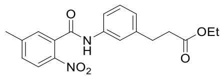	B	**44c**	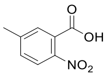	**43a**		58
**46d**	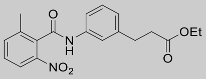	B	**44d**	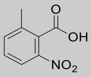	**43a**		17
**46e**	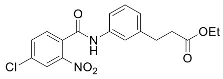	B	**44e**	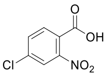	**43a**		62
**46f**	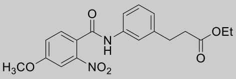	B	**44f**	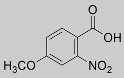	**43a**		82
**46g**	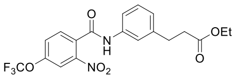	B	**44g**	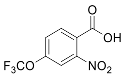	**43a**		61
**46h**	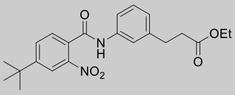	B	**44h**	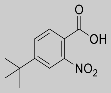	**43a**		80
**46i**	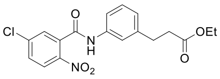	B	**44i**	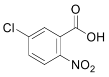	**43a**		45
**46j**	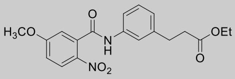	B	**44j**	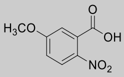	**43a**		40
**46k**	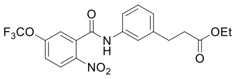	B	**44k**	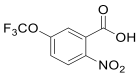	**43a**		68
**46l**	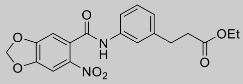	B	**44l**	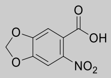	**43a**		59

ID	Structure	Procedure	Starting material A		Starting material B		Yield [%]
**46m**	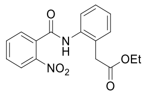	C	**45**	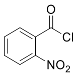	**43c**	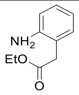	77
**46n**	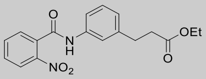	C	**45**	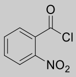	**43a**	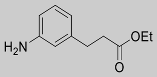	99
**46o**	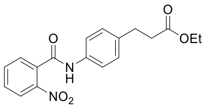	C	**45**	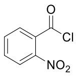	**43b**	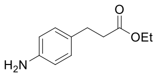	94
**46p**	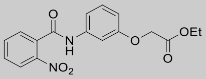	B	**44m**	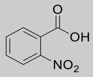	**43d**	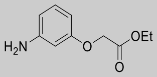	66
**46q**	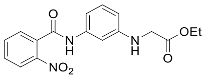	B	**44m**	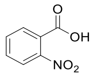	**43e**	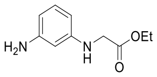	93
**46r**	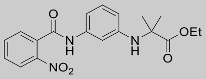	B	**44m**	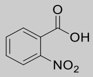	**43f**	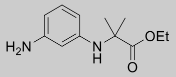	54
**46s**	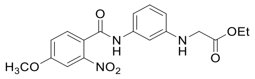	B	**44f**	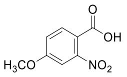	**43e**	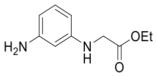	78
**47a**	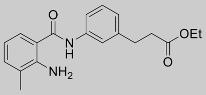	D	**46a**	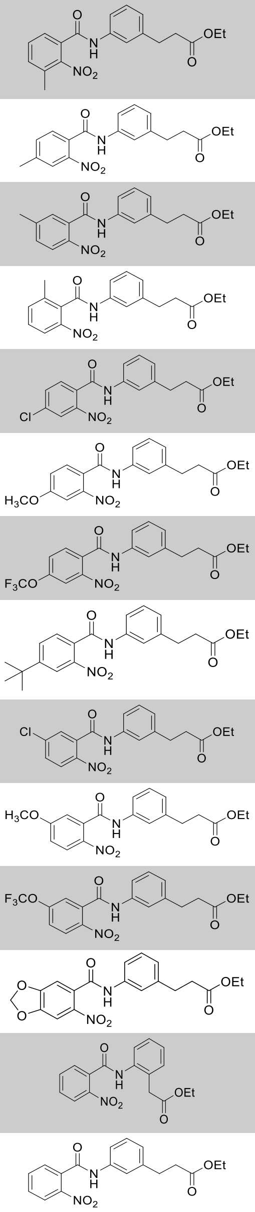			83
**47b**	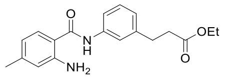	D	**46b**				97
**47c**	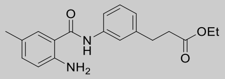	D	**46c**				95
**47d**	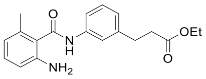	D	**46d**				76
**47e**	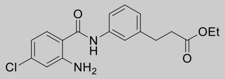	E	**46e**				92
**47f**	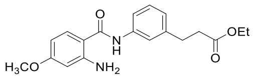	D	**46f**				98
**47g**	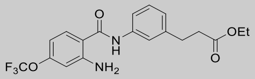	D	**46g**				90
**47h**	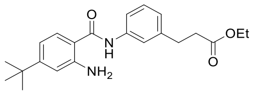	D	**46h**				85
**47i**	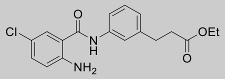	E	**46i**				61
**47j**	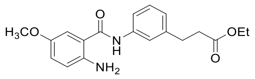	D	**46j**				47
**47k**	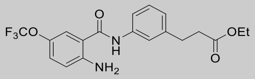	D	**46k**				97
**47l**	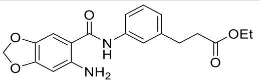	D	**46l**				75
**47m**	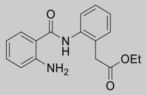	D	**46m**				86
**47n**	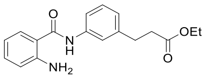	D	**46n**				82

ID	Structure	Procedure	Starting material A		Starting material B		Yield [%]
**47o**	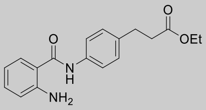	F	**46o**	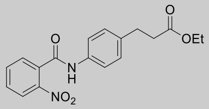			33
**47p**	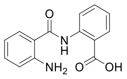	G	**51**	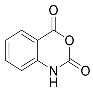	**42c**	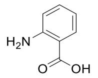	74
**47q**	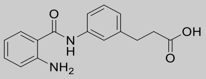	G	**51**	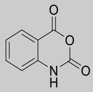	**42a**	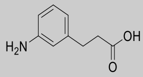	98
**47r**	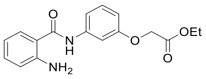	D	**46p**	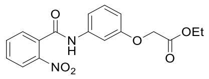			78
**47s**	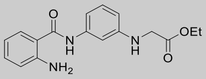	D	**46q**	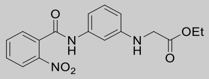			80
**47t**	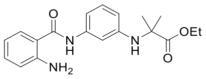	D	**46r**	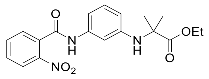			64
**47u**	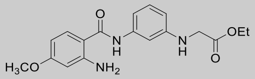	D	**46s**	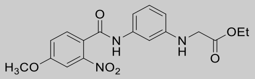			83
**48a**	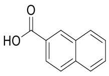	purchased					
**48b**	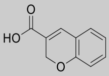	P	**73a**	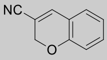			52
**48c**	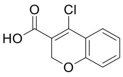	T	**77**	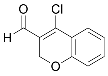			45
**48d**	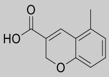	P	**73b**	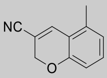			84
**48e**	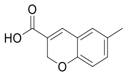	P	**73c**	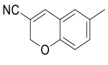			40
**48f**	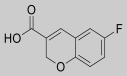	P	**73d**	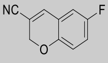			85
**48g**	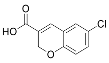	P	**73e**	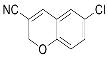			70
**48h**	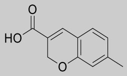	P	**73f**	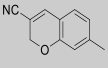			93
**48i**	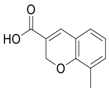	P	**73g**	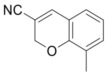			79
**48j**	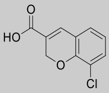	P	**73h**	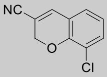			88
**48k**	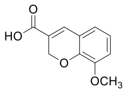	P	**73i**	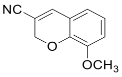			92
**48l**	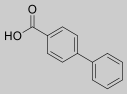	purchased					
**48m**	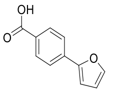	U	**62**	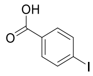	**63a**		81

ID	Structure	Procedure	Starting material A	Starting material B	Yield [%]
**48n**	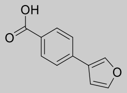	U	**62** 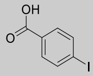	**63b** 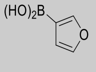	86
**48o**	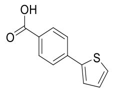	purchased			
**48p**	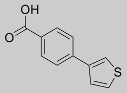	U	**62** 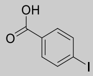	**63c** 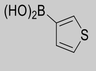	90
**48q**	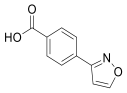	X	**67** 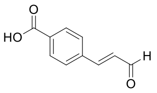	**65** 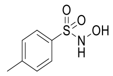	20
**48r**	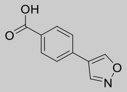	U	**62** 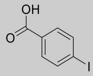	**63d** 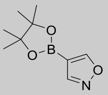	99
**48s**	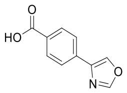	H	**70** 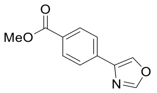		99
**48t**	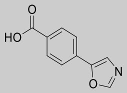	purchased			
**48u**	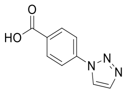	purchased			
**49a**	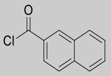	purchased			
**49b**	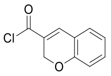	(C)	**48b** 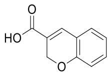		n.i.
**49c**	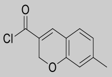	(C)	**48h** 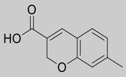		n.i.
**49d**	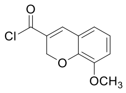	(C)	**48k** 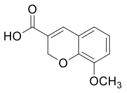		n.i.
**50a**	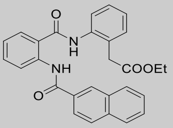	C	**47m** 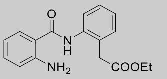	**49a** 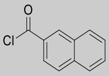	73
**50b**	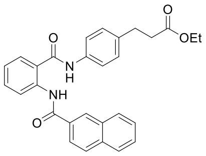	C	**47o** 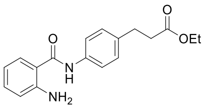	**49a** 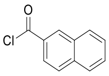	61
**50c**	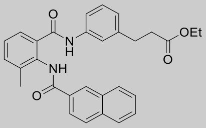	B	**47a** 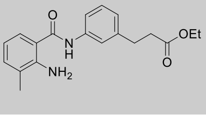	**48a** 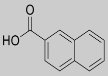	41
**50d**	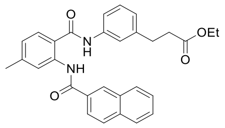	B	**47b** 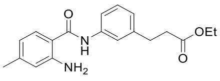	**48a** 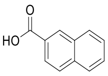	84
**50e**	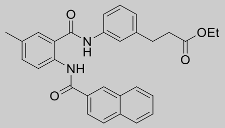	B	**47c** 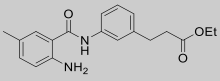	**48a** 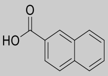	64

ID	Structure	Procedure	Starting material A		Starting material B		Yield [%]
**50f**	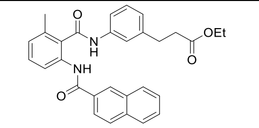	B	**47d**	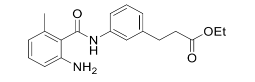	**48a**	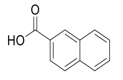	42
**50g**	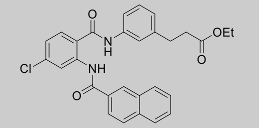	B	**47e**	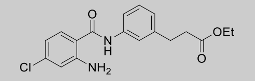	**48a**	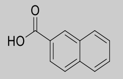	56
**50h**	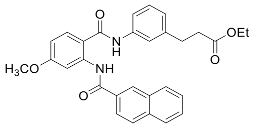	B	**47f**	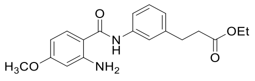	**48a**	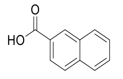	87
**50i**	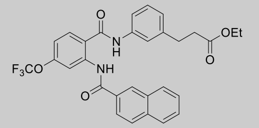	B	**47g**	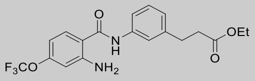	**48a**	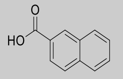	20
**50j**	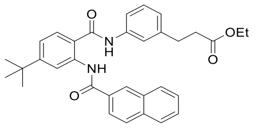	B	**47h**	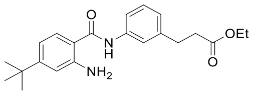	**48a**	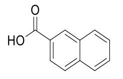	97
**50k**	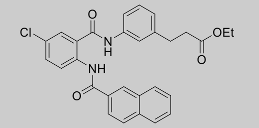	B	**47i**	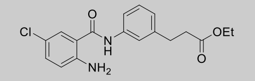	**48a**	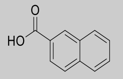	60
**50l**	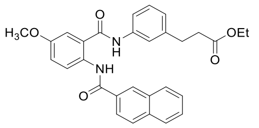	B	**47j**	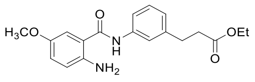	**48a**	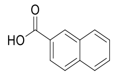	92
**50m**	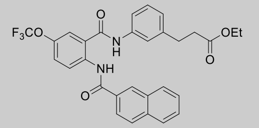	B	**47k**	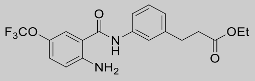	**48a**	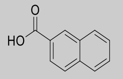	64
**50n**	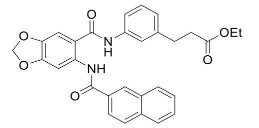	B	**47l**	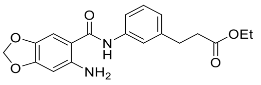	**48a**	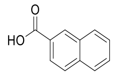	84
**50o**	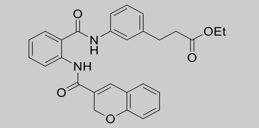	C	**47n**	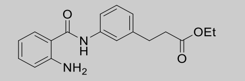	**49b**	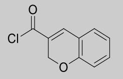	62
**50p**	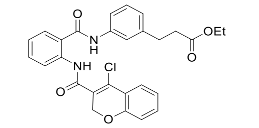	B	**47n**	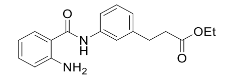	**48c**	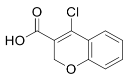	38
**50q**	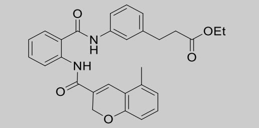	B	**47n**	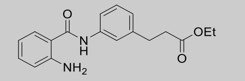	**48d**	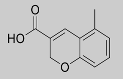	88
**50r**	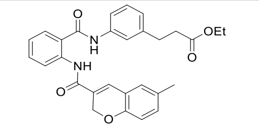	B	**47n**	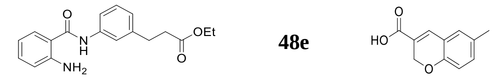	**48e**		94
**50s**	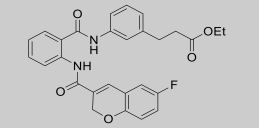	B	**47n**	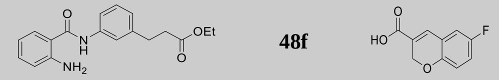	**48f**		90

ID	Structure	Procedure	Starting material A		Starting material B		Yield [%]
**50t**	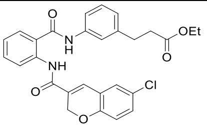	B	**47n**	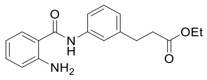	**48g**	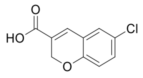	84
**50u**	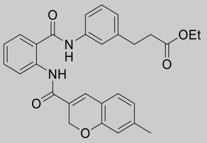	C	**47n**	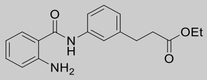	**49c**	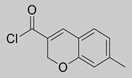	52
**50v**	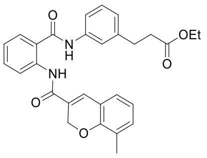	B	**47n**	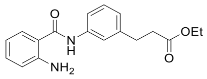	**48i**	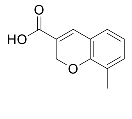	99
**50w**	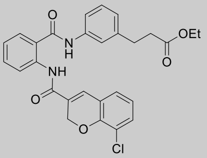	B	**47n**	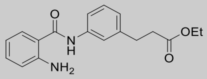	**48j**	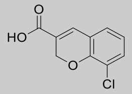	89
**50x**	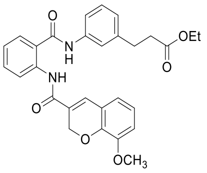	C	**47n**	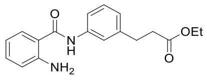	**49d**	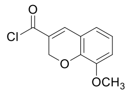	65
**50y**	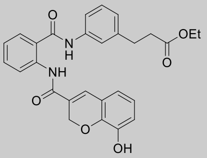	N	**50x**	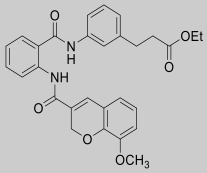			19
**50z**	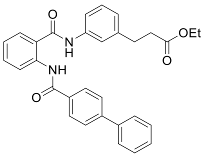	B	**47n**	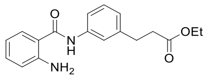	**48l**	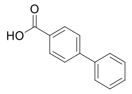	49
**50aa**	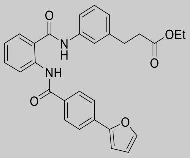	B	**47n**	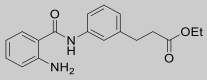	**48m**	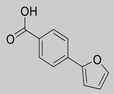	48
**50ab**	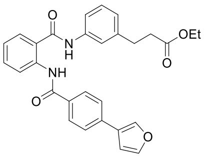	B	**47n**	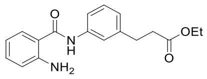	**48n**	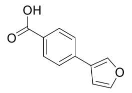	65
**50ac**	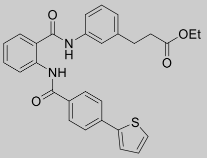	B	**47n**	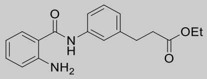	**48o**	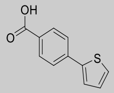	90
**50ad**	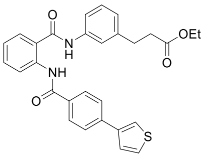	B	**47n**	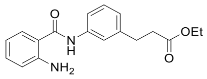	**48p**	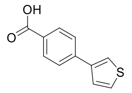	44

ID	Structure	Procedure	Starting material A		Starting material B		Yield [%]
**50ae**	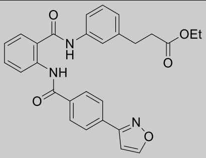	B	**47n**	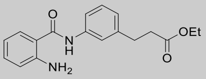	**48q**	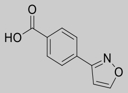	41
**50af**	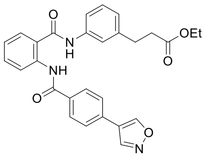	B	**47n**	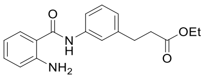	**48r**	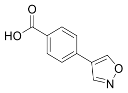	50
**50ag**	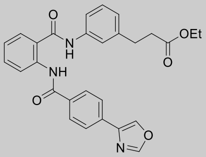	B	**47n**	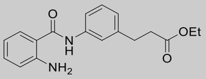	**48s**	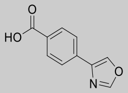	55
**50ah**	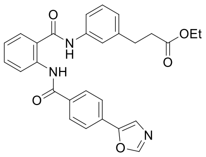	B	**47n**	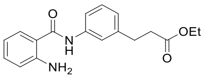	**48t**	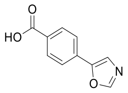	77
**50ai**	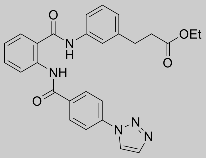	B	**47n**	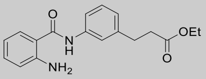	**48u**	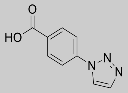	30
**50aj**	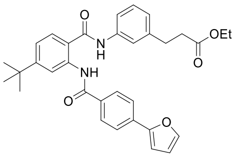	B	**47h**	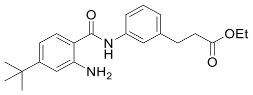	**48m**	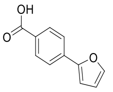	85
**50ak**	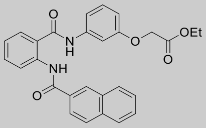	B	**47r**	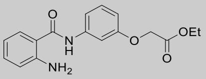	**48a**	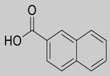	65
**50al**	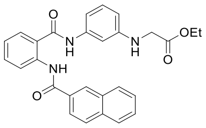	C	**47s**	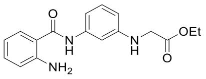	**49a**	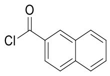	42
**50am**	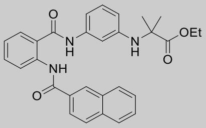	C	**47t**	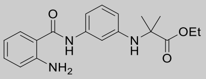	**49a**	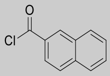	45
**50an**	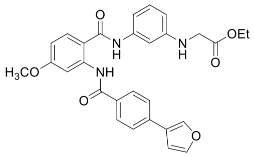	B	**47u**	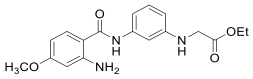	**48n**	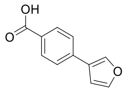	89
**51**	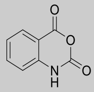	purchased					
**52**	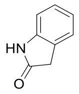	purchased					
**53**	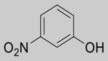	purchased					
**54a**	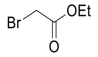	purchased					

ID	Structure	Procedure	Starting material A		Starting material B		Yield [%]
**54b**	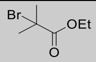	purchased					
**55**	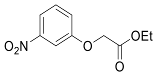	I	**53**	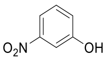	**54a**	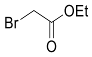	98
**56**	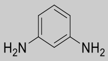	purchased					
**57**	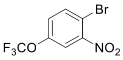	purchased					
**58**	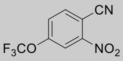	J	**57**	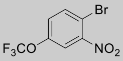			92
**59**	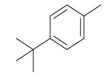	purchased					
**60**	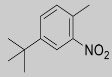	L	**59**	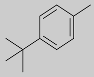			63
**61**	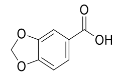	purchased					
**62**	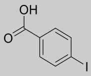	purchased					
**63a**	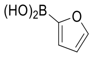	purchased					
**63b**	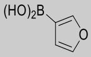	purchased					
**63c**	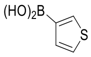	purchased					
**63d**	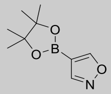	purchased					
**64**	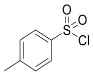	purchased					
**65**	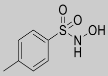	V	**64**	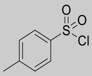			42
**66**	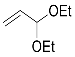	purchased					
**67**	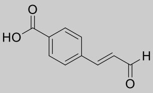	W	**62**	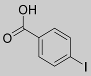	**66**	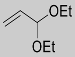	24
**68**	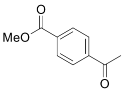	purchased					
**69**	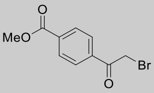	Y	**68**	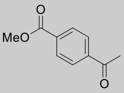			90
**70**	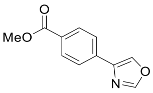	Z	**69**	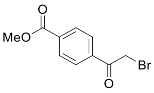			27
**71**		purchased					
**72a**	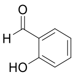	purchased					
**72b**	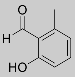	R	**75**	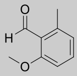			69
**72c**	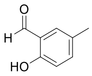	purchased					

ID	Structure	Procedure	Starting material A		Starting material B		Yield [%]
**72d**	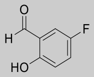	purchased					
**72e**	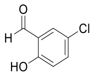	purchased					
**72f**	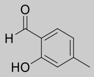	purchased					
**72g**	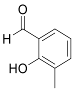	purchased					
**72h**	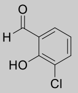	purchased					
**72i**	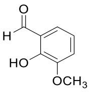	purchased					
**73a**	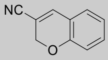	O	**71**		**72a**	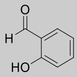	n.i.
**73b**	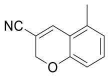	O	**71**		**72b**	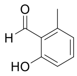	42
**73c**	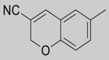	O	**71**		**72c**	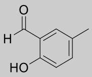	72
**73d**	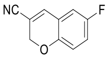	O	**71**		**72d**	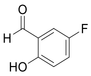	76
**73e**	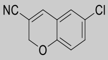	O	**71**		**72e**	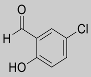	61
**73f**	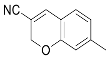	O	**71**		**72f**	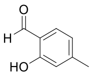	73
**73g**	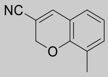	O	**71**		**72g**	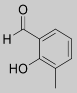	83
**73h**	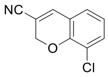	O	**71**		**72h**	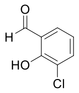	35
**73i**	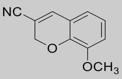	O	**71**		**72i**	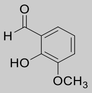	43
**74**	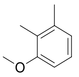	purchased					
**75**	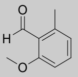	Q	**74**	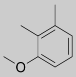			38
**76**	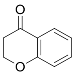	purchased					
**77**	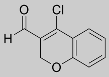	S	**76**	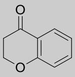			97

Analytical Characterization Data of **6**, **7**, **9**, and **12-40** and their precursors

2-(2-[2-Naphthamido]benzamido)benzoic acid (6): Preparation according to general procedure C using **47p** and **49a**. Yield: 35%. White solid. ^{1}H-NMR (400 MHz, DMSO-*d*$_6$): δ = 7.22 (dd, ^3J = 7.6, 7.6 Hz, 1H), 7.35 (dd, ^3J = 7.6, 7.6 Hz, 1H), 7.58 - 7.72 (m, 4H), 7.91 - 8.05 (m, 4H), 8.08 (d, ^3J = 8.2 Hz, 2H), 8.44 (d, ^3J = 7.9 Hz, 1H), 8.54 (s, 1H), 8.59 (d, ^3J = 8.0 Hz, 1H), 11.77 (s, 1H), 12.11 (s, 1H) ppm. ^{13}C-NMR (101 MHz, DMSO-*d*$_6$): δ = 117.8, 120.8, 122.3, 123.5, 123.6, 123.8, 124.0, 127.0, 127.7, 127.9, 128.0, 128.1, 128.5, 129.0, 131.2, 131.9, 132.2, 132.6, 134.1, 134.4, 138.6, 140.4, 165.0, 166.9, 169.7 ppm. ESI-MS: *m/z* = 409.09 ([M-H]$^-$). HRMS (MALDI): *m/z* calculated 433.11588 for $C_{25}H_{18}N_2O_4Na$, found 433.11561 ([M+Na]$^+$).

2-(2-[2-Naphthamido]benzamido)phenylacetic acid (7): Preparation according to general procedure H using **50a**. Yield: 92%. Beige solid. ^{1}H-NMR (400 MHz, DMSO-*d*$_6$): δ = 3.70 (s, 2H), 7.21 - 7.40 (m, 4H), 7.47 (d, ^3J = 7.3 Hz, 1H), 7.56 - 7.73 (m, 3H), 7.91 - 8.06 (m, 3H), 8.08 (d, ^3J = 8.6 Hz, 2H), 8.54 (s, 1H), 8.65 (d, ^3J = 8.3 Hz, 1H), 10.54 (s, 1H), 12.23 (s, 1H) ppm. ^{13}C-NMR (101 MHz, DMSO-*d*$_6$): δ = 37.9, 120.9, 121.4, 123.1, 123.2, 126.3, 126.9, 127.0, 127.2, 127.6, 127.9, 128.1, 128.6, 128.8, 129.1, 131.0, 131.4, 131.9, 132.2, 132.4, 134.4, 136.0, 139.4, 164.6, 167.6, 172.6 ppm. ESI-MS: *m/z* = 423.15 ([M-H]$^-$). HRMS (MALDI): *m/z* calculated 447.13153 for $C_{26}H_{20}N_2O_4Na$, found 447.13033 ([M+Na]$^+$).

3-(3-[2-{2-Naphthamido}benzamido]phenyl)propanoic acid (9): Preparation according to general procedure C using **47q** and **49a**. Yield: 86%. White solid. ^{1}H-NMR (400 MHz, DMSO-*d*$_6$): δ = 2.54 (t, ^3J = 7.6 Hz, 2H), 2.83 (t, ^3J = 7.6 Hz, 2H), 7.01 (d, ^3J = 7.6 Hz, 1H), 7.24 - 7.35 (m, 2H), 7.56 - 7.70 (m, 5H), 7.92 - 8.04 (m, 3H), 8.05 - 8.12 (m, 2H), 8.49 (d, ^3J = 8.2 Hz, 1H), 8.55 (s, 1H), 10.51 (s, 1H), 11.80 (s, 1H) ppm. ^{13}C-NMR (101 MHz, DMSO-*d*$_6$): δ = 30.4, 35.2, 118.9, 120.9, 121.6, 123.3, 123.4, 123.5, 124.2, 127.1, 127.7, 127.9, 128.1, 128.6, 129.0, 129.1, 131.9, 132.19, 132.22, 134.4, 138.5, 138.6, 141.4, 164.8, 167.4, 173.7 ppm. ESI-MS: *m/z* = 437.13 ([M-H]$^-$). HRMS (MALDI): *m/z* calculated 461.14718 for $C_{27}H_{22}N_2O_4Na$, found 461.14693 ([M+Na]$^+$).

3-(4-[2-{2-Naphthamido}benzamido]phenyl)propanoic acid (12): Preparation according to general procedure H using **50b**. Yield: 92%. Yellow solid. ^{1}H-NMR (400 MHz, DMSO-*d*$_6$): δ = 2.54 (t, ^3J = 7.7 Hz, 2H), 2.81 (t, ^3J = 7.6 Hz, 2H), 7.23 (d, ^3J = 8.5 Hz, 2H), 7.31 (dd, ^3J = 7.6, 7.6 Hz, 1H), 7.58-7.70 (m, 5H), 7.91-7.99 (m, 2H), 8.01 (d, ^3J = 7.6 Hz, 1H), 8.06 - 8.13 (m, 2H), 8.52 (d, ^3J = 8.3 Hz, 1H), 8.54 (s, 1H), 10.51 (s, 1H), 11.86 (s, 1H), 12.13 (br s, 1H) ppm. ^{13}C-NMR (75 MHz, DMSO-*d*$_6$): δ = 29.9, 35.3, 121.1, 121.5, 123.1, 123.4, 127.1, 127.7, 127.9, 128.1, 128.4, 128.6, 129.0, 129.1, 131.9, 132.18, 132.21, 134.4, 136.5, 136.8, 138.7, 164.7, 167.3, 173.7 ppm. ESI-MS: *m/z* = 437.19 ([M-H]$^-$). HRMS (MALDI): *m/z* calculated 461.14718 for $C_{27}H_{22}N_2O_4Na$, found 461.14699 ([M+Na]$^+$).

3-(2-[2-Naphthamido]benzamido)phenoxyacetic acid (13): Preparation according to general procedure H using **50ak**. Yield: 98%. Pale yellow solid. ^{1}H-NMR (500 MHz, DMSO-*d*$_6$): δ = 4.67 (s, 2H), 6.70 (dd, ^3J = 7.9 Hz, ^4J = 2.0 Hz, 1H), 7.27 (dd, ^3J = 8.4, 8.4 Hz, 1H), 7.32 (dd, ^3J = 7.6, 7.6 Hz, 1H), 7.37 - 7.43 (m, 2H), 7.57 - 7.70 (m, 3H), 7.92 (d, ^3J = 7.8 Hz, 1H), 7.98 (dd, ^3J = 8.6 Hz, ^4J = 1.7 Hz, 1H), 8.01 (d, ^3J = 7.9 Hz, 1H), 8.06 - 8.14 (m, 2H), 8.47 (d, ^3J = 8.2 Hz, 1H), 8.55 (s, 1H), 10.55 (s, 1H), 11.71 (s, 1H), 13.01 (br s, 1H) ppm. ^{13}C-NMR (126 MHz, DMSO-*d*$_6$): δ = 64.5, 107.3, 109.9, 113.6, 121.7, 123.4, 123.5, 123.6, 127.1, 127.7, 127.9, 128.1, 128.6, 129.0, 129.1, 129.5, 131.9, 132.20, 132.22, 134.4, 138.5, 139.8, 157.9, 164.8, 167.4, 170.1 ppm. ESI-MS: *m/z* = 438.93 ([M-H]$^-$). HRMS (MALDI): *m/z* calculated 463.12644 for $C_{26}H_{20}N_2O_5Na$, found 463.12548 ([M+Na]$^+$).

(3-[2-{2-Naphthamido}benzamido]phenyl)glycine (14): Preparation according to general procedure H using **50al**. Yield: 99%. Brown solid. ^{1}H-NMR (500 MHz, DMSO-*d*$_6$): δ = 3.79 (s, 2H), 6.35 (dd, ^3J = 8.0 Hz, ^4J = 1.4 Hz, 1H), 6.99 (d, ^3J = 7.9 Hz, 1H), 7.02 (s, 1H), 7.06 (dd, ^3J = 8.0, 8.0 Hz, 1H),

7.30 (ddd, ^3J = 7.6, 7.6 Hz, ^4J = 0.9 Hz, 1H), 7.59 - 7.70 (m, 3H), 7.92 (d, ^3J = 7.8 Hz, 1H), 7.98 (dd, ^3J = 8.6 Hz, ^4J = 1.7 Hz, 1H), 8.02 (d, ^3J = 7.9 Hz, 1H), 8.11 (d, ^3J = 8.3 Hz, 2H), 8.53 (d, ^3J = 8.2 Hz, 1H), 8.55 (s, 1H), 10.36 (s, 1H), 11.89 (s, 1H), 12.38 (br s, 1H) ppm. ^{13}C-NMR (126 MHz, DMSO-d_6): δ = 44.6, 104.9, 108.5, 109.4, 121.4, 123.2, 123.3, 123.4, 127.1, 127.7, 127.9, 128.1, 128.7, 128.9, 129.0, 129.2, 131.9, 132.1, 132.2, 134.4, 138.7, 139.2, 148.6, 164.7, 167.3, 172.6 ppm. ESI-MS: *m/z* = 438.21 ([M-H]$^-$). HRMS (MALDI): *m/z* calculated 462.14243 for $C_{26}H_{21}N_3O_4Na$, found 462.14158 ([M+Na]$^+$).

2-([3-{2-(2-Naphthamido)benzamido}phenyl]amino)-2-methylpropanoic acid (15): Preparation according to general procedure H using **50am**. Yield: 99%. Yellow solid. ^{1}H-NMR (500 MHz, DMSO-d_6): δ = 1.47 (s, 6H), 6.31 (dd, ^3J = 8.1 Hz, ^4J = 1.6 Hz, 1H), 6.90 (d, ^3J = 7.9 Hz, 1H), 7.03 (dd, ^3J = 8.0, 8.0 Hz, 1H), 7.07 (s, 1H), 7.30 (ddd, ^3J = 7.8, 7.8 Hz, ^4J = 1.0 Hz, 1H), 7.60 - 7.69 (m, 3H), 7.92 (dd, ^3J = 7.8 Hz, ^4J = 1.1 Hz, 1H), 7.99 (dd, ^3J = 8.6 Hz, ^4J = 1.8 Hz, 1H), 8.02 (d, ^3J = 7.9 Hz, 1H), 8.07 - 8.15 (m, 2H), 8.53 - 8.59 (m, 2H), 10.39 (s, 1H), 11.98 (s, 1H) ppm. ^{13}C-NMR (126 MHz, DMSO-d_6): δ = 25.9, 56.1, 106.5, 109.2, 110.1, 121.3, 122.9, 123.3, 123.4, 127.1, 127.7, 127.9, 128.2, 128.6, 128.7, 129.0, 129.2, 131.8, 132.2, 132.3, 134.5, 138.8, 138.9, 147.1, 164.6, 167.3, 177.4 ppm. ESI-MS: *m/z* = 466.24 ([M-H]$^-$). HRMS (MALDI): *m/z* calculated 490.17373 for $C_{28}H_{25}N_3O_4Na$, found 490.17242 ([M+Na]$^+$).

3-(3-[2-{2-Naphthamido}-3-methylbenzamido]phenyl)propanoic acid (16): Preparation according to general procedure H using **50c**. Yield: 65%. White solid. ^{1}H-NMR (500 MHz, DMSO-d_6): δ = 2.32 (s, 3H), 2.45 (t, ^3J = 7.7 Hz, 2H), 2.73 (t, ^3J = 7.6 Hz, 2H), 6.89 (d, ^3J = 7.6, Hz, 1H), 7.16 (dd, ^3J = 8.2, 8.2 Hz, 1H), 7.36 (dd, ^3J = 7.6, 7.6 Hz, 1H), 7.45 - 7.56 (m, 4H), 7.57 - 7.67 (m, 2H), 7.90 - 8.09 (m, 4H), 8.56 (s, 1H), 10.18 (s, 1H), 10.21 (s, 1H), 12.18 (br s, 1H) ppm. ^{13}C-NMR (126 MHz, DMSO-d_6): δ = 18.2, 30.5, 35.3, 117.5, 119.5, 123.3, 124.3, 126.1, 126.5, 126.9, 127.7, 127.8, 128.0, 128.5, 128.9, 131.7, 132.1, 134.2, 134.3, 134.9, 136.3, 139.3, 141.3, 165.7, 166.2, 173.7 ppm. ESI-MS: *m/z* = 451.23 ([M-H]$^-$). HRMS (MALDI): *m/z* calculated 475.16283 for $C_{28}H_{24}N_2O_4Na$, found 475.16336 ([M+Na]$^+$).

3-(3-[2-{2-Naphthamido}-4-methylbenzamido]phenyl)propanoic acid (17): Preparation according to general procedure H using **50d**. Yield: 84%. Beige solid. ^{1}H-NMR (500 MHz, DMSO-d_6): δ = 2.48 (s, 3H), 2.59 (t, ^3J = 7.6 Hz, 2H), 2.88 (t, ^3J = 7.6 Hz, 2H), 7.06 (d, ^3J = 7.7, Hz, 1H), 7.17 (dd, ^3J = 8.0 Hz, ^4J = 0.9 Hz, 1H), 7.32 (dd, ^3J = 7.8, 7.8 Hz, 1H), 7.61 - 7.66 (m, 2H), 7.67 - 7.73 (m, 2H), 7.93 (d, ^3J = 8.0 Hz, 1H), 8.02 (dd, ^3J = 8.6 Hz, ^4J = 1.8 Hz, 1H), 8.06 (d, ^3J = 7.9 Hz, 1H), 8.12 - 8.18 (m, 2H), 8.47 (d, ^4J = 0.6 Hz, 1H), 8.58 (d, ^4J = 1.0 Hz, 1H), 10.50 (s, 1H), 12.08 (s, 1H) ppm. ^{13}C-NMR (126 MHz, DMSO-d_6): δ = 21.5, 30.5, 35.3, 119.0, 119.7, 121.1, 121.6, 123.4, 123.9, 124.2, 127.1, 127.7, 127.8, 128.1, 128.6, 128.7, 129.0, 129.1, 132.0, 132.2, 134.4, 138.5, 139.0, 141.5, 142.6, 164.6, 167.5, 173.8 ppm. ESI-MS: *m/z* = 451.24 ([M-H]$^-$). HRMS (MALDI): *m/z* calculated 475.16283 for $C_{28}H_{24}N_2O_4Na$, found 475.16293 ([M+Na]$^+$).

3-(3-[2-{2-Naphthamido}-5-methylbenzamido]phenyl)propanoic acid (18): Preparation according to general procedure H using **50e**. Yield: 90%. White solid. ^{1}H-NMR (500 MHz, DMSO-d_6): δ = 2.45 (s, 3H), 2.50 - 2.62 (m, 2H), 2.87 (t, ^3J = 7.6 Hz, 2H), 7.05 (d, ^3J = 7.6, Hz, 1H), 7.32 (dd, ^3J = 7.8, 7.8 Hz, 1H), 7.50 (d, ^3J = 8.4 Hz, 1H), 7.62 (s, 1H), 7.64 - 7.73 (m, 3H), 7.81 (s, 1H), 8.01 (d, ^3J = 8.6 Hz, 1H), 8.06 (d, ^3J = 7.9 Hz, 1H), 8.11 - 8.17 (m, 2H), 8.40 (d, ^3J = 8.4 Hz, 1H), 8.58 (s, 1H), 10.53 (s, 1H), 11.72 (s, 1H) ppm. ^{13}C-NMR (126 MHz, DMSO-d_6): δ = 20.5, 30.5, 35.4, 118.8, 120.8, 121.7, 123.4, 123.5, 124.1, 127.1, 127.7, 127.8, 128.1, 128.6, 129.1, 129.3, 132.0, 132.2, 132.58, 132.62, 134.4, 136.1, 138.6, 141.5, 164.6, 167.4, 173.8 ppm. ESI-MS: *m/z* = 451.24 ([M-H]$^-$). HRMS (MALDI): *m/z* calculated 475.16283 for $C_{28}H_{24}N_2O_4Na$, found 475.16258 ([M+Na]$^+$).

3-(3-[2-{2-Naphthamido}-6-methylbenzamido]phenyl)propanoic acid (19): Preparation according to general procedure H using **50f**. Yield: 80%. White solid. ^{1}H-NMR (500 MHz, DMSO-d_6): δ = 2.40 (s, 3H), 2.45 - 2.53 (m, 2H), 2.77 (t, ^3J = 7.7 Hz, 2H), 6.94 (d, ^3J = 7.1, Hz, 1H), 7.16 - 7.26 (m, 2H), 7.42 (dd, ^3J = 7.8, 7.8 Hz, 1H), 7.51 - 7.67 (m, 5H), 7.85 - 7.93 (m, 2H), 7.97 (d, ^3J = 8.1 Hz, 1H), 8.00 (d, ^3J = 8.6 Hz, 1H), 8.41 (s, 1H), 10.10 (s, 1H), 10.30 - 10.35 (m, 1H), 12.20 (br s, 1H) ppm. ^{13}C-NMR

(126 MHz, DMSO-d_6): δ = 20.1, 30.9, 35.7, 118.1, 120.1, 123.9, 124.0, 124.6, 127.4, 128.0, 128.1, 128.3, 128.4, 128.6, 129.1, 129.2, 129.6, 132.3, 132.5, 133.7, 134.7, 135.4, 136.0, 139.4, 141.9, 166.2, 166.3, 174.1 ppm. ESI-MS: *m/z* = 475.23 ([M+Na]$^+$). HRMS (MALDI): *m/z* calculated 475.16283 for $C_{28}H_{24}N_2O_4Na$, found 475.16183 ([M+Na]$^+$).

3-(3-[2-{2-Naphthamido}-4-chlorobenzamido]phenyl)propanoic acid (20): Preparation according to general procedure H using **50g**. Yield: 82%. Beige solid. ^{1}H-NMR (500 MHz, DMSO-d_6): δ = 2.55 (t, ^3J = 7.6 Hz, 2H), 2.83 (t, ^3J = 7.6 Hz, 2H), 7.03 (d, ^3J = 7.7 Hz, 1H), 7.29 (dd, ^3J = 7.8, 7.8 Hz, 1H), 7.40 (dd, ^3J = 8.5 Hz, ^4J = 2.2 Hz, 1H), 7.54 - 7.61 (m, 2H), 7.63 (ddd, ^3J = 7.4, 7.4 Hz, ^4J = 1.2 Hz, 1H), 7.67 (ddd, ^3J = 7.4, 7.4 Hz, ^4J = 1.3 Hz, 1H), 7.96 (dd, ^3J = 8.6 Hz, ^4J = 1.8 Hz, 1H), 7.99 (d, ^3J = 8.5 Hz, 1H), 8.02 (d, ^3J = 7.9 Hz, 1H), 8.10 (dd, ^3J = 8.5, 8.5 Hz, 2H), 8.54 (s, 1H), 8.63 (d, ^4J = 2.1 Hz, 1H), 10.58 (s, 1H), 12.00 (s, 1H), 12.15 (br s, 1H) ppm. ^{13}C-NMR (126 MHz, DMSO-d_6): δ = 30.4, 35.1, 119.0, 120.7, 121.0, 121.4, 123.0, 123.4, 124.4, 127.2, 127.7, 128.1, 128.3, 128.66, 128.73, 129.1, 130.8, 131.5, 132.2, 134.5, 136.6, 138.3, 140.1, 141.4, 165.0, 166.5, 173.7 ppm. ESI-MS: *m/z* = 471.01 ([M-H]$^-$). HRMS (MALDI): *m/z* calculated 495.10821 for $C_{27}H_{21}ClN_2O_4Na$, found 495.10725 ([M+Na]$^+$).

3-(3-[2-{2-Naphthamido}-4-hydroxybenzamido]phenyl)propanoic acid (21): Preparation according to general procedure N using **22**. Yield: 65%. White solid. ^{1}H-NMR (500 MHz, DMSO-d_6): δ = 2.54 (t, ^3J = 7.6 Hz, 2H), 2.83 (t, ^3J = 7.6 Hz, 2H), 6.66 (dd, ^3J = 8.7 Hz, ^4J = 2.4 Hz, 1H), 7.00 (d, ^3J = 7.6 Hz, 1H), 7.27 (dd, ^3J = 7.8, 7.8 Hz, 1H), 7.55 (s, 1H), 7.57 (d, ^3J = 8.1 Hz, 1H), 7.60 - 7.70 (m, 2H), 7.91 (d, ^3J = 8.7 Hz, 1H), 7.98 (dd, ^3J = 8.6 Hz, ^4J = 1.6 Hz, 1H), 8.02 (d, ^3J = 8.0 Hz, 1H), 8.11 (dd, ^3J = 8.3, 8.3 Hz, 2H), 8.26 (d, ^4J = 2.4 Hz, 1H), 8.54 (s, 1H), 10.28 (s, 1H), 11.42 (br s, 2H), 12.57 (s, 1H) ppm. ^{13}C-NMR (126 MHz, DMSO-d_6): δ = 30.4, 35.4, 107.1, 110.1, 111.7, 119.2, 121.2, 123.3, 124.1, 127.1, 127.7, 127.8, 128.1, 128.6, 128.7, 129.1, 130.8, 132.0, 132.3, 134.4, 138.5, 141.5, 141.7, 161.3, 164.6, 167.8, 173.8 ppm. ESI-MS: *m/z* = 453.08 ([M-H]$^-$). HRMS (MALDI): *m/z* calculated 477.14209 for $C_{27}H_{22}N_2O_5Na$, found 477.14099 ([M+Na]$^+$).

3-(3-[2-{2-Naphthamido}-4-methoxybenzamido]phenyl)propanoic acid (22): Preparation according to general procedure H using **50h**. Yield: 86%. White solid. ^{1}H-NMR (500 MHz, DMSO-d_6): δ = 2.56 (t, ^3J = 7.6 Hz, 2H), 2.84 (t, ^3J = 7.6 Hz, 2H), 3.89 (s, 3H), 6.87 (dd, ^3J = 8.8 Hz, ^4J = 2.6 Hz, 1H), 7.02 (d, ^3J = 7.6 Hz, 1H), 7.29 (dd, ^3J = 7.8, 7.8 Hz, 1H), 7.52 - 7.72 (m, 4H), 7.94 - 8.06 (m, 3H), 8.11 (dd, ^3J = 6.8, 6.8 Hz, 2H), 8.36 (d, ^4J = 2.5 Hz, 1H), 8.55 (s, 1H), 10.38 (s, 1H), 12.17 (br s, 1H), 12.51 (s, 1H) ppm. ^{13}C-NMR (126 MHz, DMSO-d_6): δ = 30.4, 35.1, 55.5, 105.9, 108.5, 113.6, 119.3, 121.3, 123.3, 124.2, 127.1, 127.7, 127.9, 128.2, 128.6, 128.8, 129.2, 130.7, 131.9, 132.2, 134.5, 138.5, 141.4, 141.5, 162.3, 164.8, 167.5, 173.7 ppm. ESI-MS: *m/z* = 467.25 ([M-H]$^-$). HRMS (MALDI): *m/z* calculated 491.15774 for $C_{28}H_{24}N_2O_5Na$, found 491.15677 ([M+Na]$^+$).

3-(3-[2-{2-Naphthamido}-4-{trifluoromethoxy}benzamido]phenyl)propanoic acid (23): Preparation according to general procedure H using **50i**. Yield: 56%. White solid. ^{1}H-NMR (500 MHz, DMSO-d_6): δ = 2.54 (t, ^3J = 7.6 Hz, 2H), 2.83 (t, ^3J = 7.5 Hz, 2H), 7.03 (d, ^3J = 7.6 Hz, 1H), 7.24 - 7.37 (m, 2H), 7.53 - 7.72 (m, 4H), 7.96 (dd, ^3J = 8.6 Hz, ^4J = 1.3 Hz, 1H), 8.02 (d, ^3J = 8.0 Hz, 1H), 8.05 - 8.16 (m, 3H), 8.52 - 8.61 (m, 2H), 10.62 (s, 1H), 12.01 (br s, 2H) ppm. ^{13}C-NMR (126 MHz, DMSO-d_6): δ = 30.4, 35.2, 112.8, 115.0, 119.0, 120.1, 121.0, 121.7, 123.4, 124.4, 127.2, 127.8, 128.1, 128.3, 128.67, 128.72, 129.1, 131.3, 131.4, 132.2, 134.6, 138.4, 140.5, 141.5, 150.4, 165.1, 166.3, 173.7 ppm. ESI-MS: *m/z* = 520.99 ([M-H]$^-$). HRMS (MALDI): *m/z* calculated 545.12948 for $C_{28}H_{21}F_3N_2O_5Na$, found 545.12780 ([M+Na]$^+$).

3-(3-[2-{2-Naphthamido}-4-{tert-butyl}benzamido]phenyl)propanoic acid (24): Preparation according to general procedure H using **50j**. Yield: 73%. White solid. ^{1}H-NMR (500 MHz, DMSO-d_6): δ = 1.36 (s, 9H), 2.54 (t, ^3J = 7.6 Hz, 2H), 2.82 (t, ^3J = 7.6 Hz, 2H), 7.00 (d, ^3J = 7.6, Hz, 1H), 7.27 (dd, ^3J = 8.6, 7.8 Hz, 1H), 7.34 (dd, ^3J = 8.3 Hz, ^4J = 1.9 Hz, 1H), 7.55 - 7.61 (m, 2H), 7.61 - 7.69 (m, 2H), 7.89 (d, ^3J = 8.3 Hz, 1H), 7.98 (dd, ^3J = 8.6 Hz, ^4J = 1.7 Hz, 1H), 8.02 (d, ^3J = 7.7 Hz, 1H), 8.09 (dd, ^3J = 8.2, 8.2 Hz, 2H), 8.55 (s, 1H), 8.62 (d, ^4J = 1.8 Hz, 1H), 10.44 (s, 1H), 11.89 (s, 1H), 12.17 (br s, 1H) ppm.

^{13}C-NMR (126 MHz, DMSO-d_6): δ = 30.4, 30.9, 34.9, 35.2, 118.3, 118.8, 120.4, 120.5, 120.8, 123.5, 124.1, 127.1, 127.7, 127.8, 128.1, 128.6, 128.8, 129.1, 132.0, 132.2, 134.4, 138.6, 138.7, 141.4, 155.3, 164.8, 167.3, 173.7 ppm. ESI-MS: *m/z* = 493.14 ([M-H]$^-$). HRMS (MALDI): *m/z* calculated 517.20978 for $C_{31}H_{30}N_2O_4Na$, found 517.20837 ([M+Na]$^+$).

3-(3-[2-{2-Naphthamido}-5-chlorobenzamido]phenyl)propanoic acid (25): Preparation according to general procedure H using **50k**. Yield: 88%. White solid. ^{1}H-NMR (500 MHz, DMSO-d_6): δ = 2.54 (t, ^3J = 7.6 Hz, 2H), 2.82 (t, ^3J = 7.6 Hz, 2H), 7.02 (d, ^3J = 7.6, Hz, 1H), 7.28 (dd, ^3J = 7.8, 7.8 Hz, 1H), 7.57 (s, 1H), 7.58 - 7.68 (m, 3H), 7.71 (dd, ^3J = 8.9 Hz, ^4J = 2.4 Hz, 1H), 7.92 - 8.00 (m, 2H), 8.01 (d, ^3J = 8.0 Hz, 1H), 8.06 - 8.12 (m, 2H), 8.45 (d, ^3J = 8.9 Hz, 1H), 8.54 (s, 1H), 10.60 (s, 1H), 11.68 (s, 1H), 12.16 (br s, 1H) ppm. ^{13}C-NMR (126 MHz, DMSO-d_6): δ = 30.4, 35.1, 118.9, 120.9, 123.5, 123.6, 124.4, 125.4, 127.1, 127.3, 127.8, 128.1, 128.2, 128.66, 128.68, 129.1, 131.6, 131.8, 132.2, 134.5, 137.4, 138.4, 141.4, 164.9, 165.9, 173.7 ppm. ESI-MS: *m/z* = 471.22 ([M-H]$^-$). HRMS (MALDI): *m/z* calculated 495.10821 for $C_{27}H_{21}ClN_2O_4Na$, found 495.10712 ([M+Na]$^+$).

3-(3-[2-{2-Naphthamido}-5-hydroxybenzamido]phenyl)propanoic acid (26): Preparation according to general procedure N using **50l**. Yield: 22%. Pale yellow solid. ^{1}H-NMR (500 MHz, DMSO-d_6): δ = 2.43 (t, ^3J = 7.7 Hz, 2H), 2.70 (t, ^3J = 7.6 Hz, 2H), 6.87 (d, ^3J = 7.7, Hz, 1H), 6.93 (dd, ^3J = 8.8 Hz, ^4J = 2.8 Hz, 1H), 7.11 - 7.17 (m, 2H), 7.44 - 7.56 (m, 4H), 7.84 (d, ^3J = 8.5 Hz, ^4J = 1.7 Hz, 1H), 7.89 (d, ^3J = 7.8 Hz, 1H), 7.95 (d, ^3J = 8.5 Hz, 2H), 7.99 (d, ^3J = 8.8 Hz, 1H), 8.40 (s, 1H), 9.66 (br s, 1H), 10.32 (s, 1H), 11.04 (s, 1H), 12.03 (br s, 1H) ppm. ^{13}C-NMR (126 MHz, DMSO-d_6): δ = 30.4, 35.1, 115.3, 118.4, 118.5, 120.5, 123.6, 124.0, 124.3, 126.7, 127.0, 127.69, 127.70, 127.9, 128.4, 128.6, 129.0, 129.5, 132.1, 132.2, 134.3, 138.8, 141.4, 153.6, 164.6, 167.0, 173.7 ppm. ESI-MS: *m/z* = 477.15 ([M+Na]$^+$). HRMS (MALDI): *m/z* calculated 477.14209 for $C_{27}H_{22}N_2O_5Na$, found 477.14140 ([M+Na]$^+$).

3-(3-[2-{2-Naphthamido}-5-methoxybenzamido]phenyl)propanoic acid (27): Preparation according to general procedure H using **50l**. Yield: 91%. Beige solid. ^{1}H-NMR (500 MHz, DMSO-d_6): δ = 2.53 (t, ^3J = 7.5 Hz, 2H), 2.82 (t, ^3J = 7.6 Hz, 2H), 3.87 (s, 3H), 7.00 (d, ^3J = 7.6, Hz, 1H), 7.23 (dd, ^3J = 9.0 Hz, ^4J = 2.9 Hz, 1H), 7.27 (dd, ^3J = 7.8, 7.8 Hz, 1H), 7.43 (d, ^4J = 2.4 Hz, 1H), 7.54 - 7.68 (m, 4H), 7.95 (d, ^3J = 8.6 Hz, 1H), 8.00 (d, ^3J = 7.9 Hz, 1H), 8.04 - 8.10 (m, 2H), 8.26 (d, ^3J = 9.0 Hz, 1H), 8.52 (s, 1H), 10.46 (s, 1H), 11.34 (s, 1H), 12.14 (br s, 1H) ppm. ^{13}C-NMR (126 MHz, DMSO-d_6): δ = 30.4, 35.1, 55.6, 113.9, 117.4, 118.8, 120.8, 123.6, 124.0, 124.1, 125.9, 127.0, 127.7, 127.8, 128.0, 128.5, 128.6, 129.0, 131.2, 132.0, 132.2, 134.3, 138.6, 141.4, 155.1, 164.6, 166.8, 173.7 ppm. ESI-MS: *m/z* = 467.24 ([M-H]$^-$). HRMS (MALDI): *m/z* calculated 491.15774 for $C_{28}H_{24}N_2O_5Na$, found 491.15664 ([M+Na]$^+$).

3-(3-[2-{2-Naphthamido}-5-{trifluoromethoxy}benzamido]phenyl)propanoic acid (28): Preparation according to general procedure H using **50m**. Yield: 97%. White solid. ^{1}H-NMR (500 MHz, Acetone-d_6): δ = 2.66 (t, ^3J = 7.6 Hz, 2H), 2.95 (t, ^3J = 7.6 Hz, 2H), 7.12 (d, ^3J = 7.8 Hz, 1H), 7.33 (dd, ^3J = 7.8, 7.8 Hz, 1H), 7.61 - 7.67 (m, 3H), 7.67 (s, 1H), 7.71 (d, ^3J = 8.1 Hz, 1H), 7.99 - 8.04 (m, 2H), 8.06 - 8.13 (m, 3H), 8.61 (s, 1H), 8.98 (d, ^3J = 9.2 Hz, 1H), 9.99 (s, 1H), 10.63 (br s, 1H), 12.22 (s, 1H) ppm. ^{13}C-NMR (126 MHz, Acetone-d_6): δ = 31.5, 35.7, 120.1, 121.5, 122.1, 122.3, 123.4, 123.6, 124.4, 125.8, 126.0, 127.9, 128.7, 128.97, 128.99, 129.6, 129.7, 130.1, 132.9, 133.7, 136.0, 139.2, 140.2, 142.8, 144.3, 165.7, 167.4, 173.8 ppm. ESI-MS: *m/z* = 521.04 ([M-H]$^-$). HRMS (MALDI): *m/z* calculated 545.12948 for $C_{28}H_{21}F_3N_2O_5Na$, found 545.12842 ([M+Na]$^+$).

3-(3-[6-{2-Naphthamido}benzo[1,3]dioxole-5-carboxamido]phenyl)propanoic acid (29): Preparation according to general procedure H using **50n**. Yield: 87%. White solid. ^{1}H-NMR (500 MHz, DMSO-d_6): δ = 2.55 (t, ^3J = 7.6 Hz, 2H), 2.83 (t, ^3J = 7.5 Hz, 2H), 6.18 (s, 2H), 7.01 (d, ^3J = 7.5, Hz, 1H), 7.28 (dd, ^3J = 7.8, 7.8 Hz, 1H), 7.52 - 7.69 (m, 5H), 7.96 (d, ^3J = 8.5 Hz, 1H), 8.01 (d, ^3J = 7.9 Hz, 1H), 8.06 - 8.12 (m, 2H), 8.20 (s, 1H), 8.53 (s, 1H), 10.29 (s, 1H), 12.15 (br s, 1H), 12.29 (s, 1H) ppm. ^{13}C-NMR (126 MHz, DMSO-d_6): δ = 30.4, 35.1, 102.2, 102.2, 108.1, 114.7, 119.1, 121.2, 123.3, 124.2, 127.1, 127.7, 127.8, 128.1, 128.6, 128.7, 129.1, 131.8, 132.2, 134.4, 135.7, 138.4, 141.4, 142.8, 150.1, 164.5,

167.0, 173.7 ppm. ESI-MS: *m/z* = 481.06 ([M-H]$^-$). HRMS (MALDI): *m/z* calculated 505.13701 for $C_{28}H_{22}N_2O_6Na$, found 505.13669 ([M+Na]$^+$).

3-(3-[2-{2H-Chromene-3-carboxamido}benzamido]phenyl)propanoic acid (S1): Preparation according to general procedure H using **50o**. Yield: 99%. Yellow solid. ^{1}H-NMR (400 MHz, DMSO-d_6): δ = 2.55 (t, ^3J = 7.6 Hz, 2H), 2.84 (t, ^3J = 7.5 Hz, 2H), 5.00 (s, 2H), 6.87 (d, ^3J = 8.0 Hz, 1H), 6.92 - 7.08 (m, 2H), 7.20 - 7.32 (m, 4H), 7.35 (s, 1H), 7.52 - 7.67 (m, 3H), 7.90 (d, ^3J = 7.2 Hz, 1H), 8.31 (d, ^3J = 8.1 Hz, 1H), 10.46 (s, 1H), 11.34 (s, 1H) ppm. ^{13}C-NMR (126 MHz, DMSO-d_6): δ = 30.4, 35.1, 64.2, 115.8, 118.9, 120.91, 120.93, 121.6, 122.0, 123.1, 123.3, 124.2, 127.1, 127.9, 128.6, 128.9, 129.0, 131.6, 132.1, 138.2, 138.6, 141.4, 154.3, 162.7, 167.2, 173.7 ppm. ESI-MS: *m/z* = 441.18 ([M-H]$^-$). HRMS (MALDI): *m/z* calculated 441.14450 for $C_{26}H_{21}N_2O_5$, found 441.14408 ([M-H]$^+$).

3-(3-[2-{4-Chloro-2H-chromene-3-carboxamido}benzamido]phenyl)propanoic acid (S2): Preparation according to general procedure H using **50p**. Yield: 54%. Yellow solid. ^{1}H-NMR (500 MHz, DMSO-d_6): δ = 2.53 (t, ^3J = 7.7 Hz, 2H), 2.81 (t, ^3J = 7.6 Hz, 2H), 5.01 (s, 2H), 6.96 (dd, ^3J = 8.1 Hz, ^4J = 0.6 Hz, 1H), 7.00 (d, ^3J = 7.6 Hz, 1H), 7.10 (ddd, ^3J = 7.6, 7.6 Hz, ^4J = 0.9 Hz, 1H), 7.26 (dd, ^3J = 7.8, 7.8 Hz, 1H), 7.31 (dd, ^3J = 7.6, 7.6 Hz, 1H), 7.37 (ddd, ^3J = 8.0, 8.0 Hz, ^4J = 1.5 Hz, 1H), 7.52 - 7.63 (m, 4H), 7.84 (d, ^3J = 7.1 Hz, 1H), 8.27 (d, ^3J = 8.1 Hz, 1H), 10.47 (s, 1H), 11.13 (s, 1H), 12.10 (br s, 1H) ppm. ^{13}C-NMR (126 MHz, DMSO-d_6): δ = 30.4, 35.1, 66.6, 116.1, 118.6, 120.5, 120.6, 122.0, 122.4, 124.0, 124.1, 125.8, 125.9, 128.6, 128.9, 131.9, 132.2, 136.9, 138.6, 141.4, 154.5, 161.9, 166.7, 173.7 ppm. ESI-MS: *m/z* = 475.00 ([M-H]$^-$). HRMS (MALDI): *m/z* calculated 499.10312 for $C_{26}H_{21}ClN_2O_5Na$ found 499.10171 ([M+Na]$^+$).

3-(3-[2-{5-Methyl-2H-chromene-3-carboxamido}benzamido]phenyl)propanoic acid (S3): Preparation according to general procedure H using **50q**. Yield: 91%. Yellow solid. ^{1}H-NMR (500 MHz, DMSO-d_6): δ = 2.37 (s, 3H), 2.53 (t, ^3J = 7.7 Hz, 2H), 2.82 (t, ^3J = 7.6 Hz, 2H), 4.93 (d, ^4J = 0.9 Hz, 2H), 6.73 (d, ^3J = 8.1 Hz, 1H), 6.83 (d, ^3J = 7.5 Hz, 1H), 7.01 (d, ^3J = 7.6 Hz, 1H), 7.16 (dd, ^3J = 7.8, 7.8 Hz, 1H), 7.24 - 7.30 (m, 2H), 7.55 (s, 1H), 7.57 - 7.63 (m, 2H), 7.69 (s, 1H), 7.91 (dd, ^3J = 7.8 Hz, ^4J = 1.0 Hz, 1H), 8.31 (d, ^3J = 8.0 Hz, 1H), 10.47 (s, 1H), 11.49 (s, 1H), 12.15 (br s, 1H) ppm. ^{13}C-NMR (126 MHz, DMSO-d_6): 17.9, 30.5, 35.2, 63.4, 113.7, 118.6, 119.7, 120.7, 121.4, 123.27, 123.32, 123.4, 124.1, 125.2, 126.8, 128.6, 128.9, 131.0, 132.1, 136.4, 138.2, 138.7, 141.4, 154.5, 162.8, 167.2, 173.7 ppm. ESI-MS: *m/z* = 455.16 ([M-H]$^-$). HRMS (MALDI): *m/z* calculated 479.15774 for $C_{27}H_{24}N_2O_5Na$, found 479.15775 ([M+Na]$^+$).

3-(3-[2-{6-Methyl-2H-chromene-3-carboxamido}benzamido]phenyl)propanoic acid (S4): Preparation according to general procedure H using **50r**. Yield: 96%. Yellow solid. ^{1}H-NMR (500 MHz, DMSO-d_6): δ = 2.23 (s, 3H), 2.55 (t, ^3J = 7.6 Hz, 2H), 2.83 (t, ^3J = 7.6 Hz, 2H), 4.95 (d, ^4J = 1.0 Hz, 2H), 6.77 (d, ^3J = 8.1 Hz, 1H), 7.02 (d, ^3J = 7.6 Hz, 1H), 7.07 (dd, ^3J = 8.2 Hz, ^4J = 1.7 Hz, 1H), 7.10 (s, 1H), 7.21 - 7.33 (m, 3H), 7.52 - 7.64 (m, 3H), 7.90 (dd, ^3J = 7.8 Hz, ^4J = 1.0 Hz, 1H), 8.31 (d, ^3J = 8.2 Hz, 1H), 10.46 (s, 1H), 11.32 (s, 1H), 12.13 (br s, 1H) ppm. ^{13}C-NMR (126 MHz, DMSO-d_6): 20.0, 30.4, 35.1, 64.1, 115.5, 119.0, 120.7, 120.9, 121.5, 123.1, 123.3, 124.2, 127.2, 128.1, 128.6, 128.98, 129.01, 130.8, 132.0, 132.1, 138.2, 138.5, 141.4, 152.1, 162.7, 167.2, 173.7 ppm. ESI-MS: *m/z* = 455.10 ([M-H]$^-$). ESI-MS: *m/z* = 455.21 ([M-H]$^-$). HRMS (MALDI): *m/z* calculated 479.15774 for $C_{27}H_{24}N_2O_5Na$, found 479.15855 ([M+Na]$^+$).

3-(3-[2-{6-Fluoro-2H-chromene-3-carboxamido}benzamido]phenyl)propanoic acid (S5): Preparation according to general procedure H using **50s**. Yield: 62%. Yellow solid. ^{1}H-NMR (500 MHz, DMSO-d_6): δ = 2.54 (t, ^3J = 7.6 Hz, 2H), 2.83 (t, ^3J = 7.6 Hz, 2H), 4.99 (d, ^4J = 1.1 Hz, 2H), 6.90 (dd, ^3J = 8.9 Hz, ^4J = 4.6 Hz, 1H), 7.01 (d, ^3J = 7.7 Hz, 1H), 7.10 (ddd, ^3J = 8.7, 8.7 Hz, ^4J = 3.1 Hz, 1H), 7.24 (dd, ^3J = 8.6 Hz, ^4J = 3.1 Hz, 1H), 7.26 - 7.31 (m, 2H), 7.33 (s, 1H), 7.53 - 7.63 (m, 3H), 7.89 (dd, ^3J = 7.8 Hz, ^4J = 0.9 Hz, 1H), 8.27 (d, ^3J = 8.1 Hz, 1H), 10.46 (s, 1H), 11.30 (s, 1H), 12.09 (br s, 1H) ppm. ^{13}C-NMR (126 MHz, DMSO-d_6): δ = 30.4, 35.1, 64.4, 114.7, 117.0, 117.6, 118.9, 120.9, 121.7, 122.2, 123.4, 123.5, 124.2, 124.9, 127.2, 128.7, 129.0, 132.1, 138.0, 138.5, 141.4, 150.4, 156.9, 162.5,

167.1, 173.7 ppm. ESI-MS: *m/z* = 459.08 ([M-H]$^-$). HRMS (MALDI): *m/z* calculated 483.13267 for $C_{26}H_{21}FN_2O_5Na$ found 483.13223 ([M+Na]$^+$).

3-(3-[2-{6-Chloro-2H-chromene-3-carboxamido}benzamido]phenyl)propanoic acid (S6): Preparation according to general procedure H using **50t**. Yield: 72%. Pale yellow solid. ^{1}H-NMR (500 MHz, DMSO-*d*$_6$): δ = 2.54 (t, ^3J = 7.6 Hz, 2H), 2.83 (t, ^3J = 7.5 Hz, 2H), 5.02 (d, ^4J = 1.1 Hz, 2H), 6.90 (d, ^3J = 8.6 Hz, 1H), 7.01 (d, ^3J = 7.6 Hz, 1H), 7.25 - 7.31 (m, 3H), 7.33 (s, 1H), 7.43 (dd, ^3J = 2.7 Hz, 1H), 7.51 - 7.65 (m, 3H), 7.89 (d, ^3J = 7.8 Hz, 1H), 8.27 (d, ^3J = 8.2 Hz, 1H), 10.47 (s, 1H), 11.28 (s, 1H), 12.13 (br s, 1H) ppm. ^{13}C-NMR (126 MHz, DMSO-*d*$_6$): δ = 30.4, 35.1, 64.5, 117.5, 118.9, 120.8, 121.7, 122.6, 123.4, 123.5, 124.2, 125.4, 126.8, 128.0, 128.5, 128.6, 129.0, 130.8, 132.1, 138.0, 138.5, 141.4, 152.9, 162.4, 167.1, 173.7 ppm. ESI-MS: *m/z* = 475.05 ([M-H]$^-$). HRMS (MALDI): *m/z* calculated 499.10312 for $C_{26}H_{21}ClN_2O_5Na$ found 499.10239 ([M+Na]$^+$).

3-(3-[2-{7-Methyl-2H-chromene-3-carboxamido}benzamido]phenyl)propanoic acid (S7): Preparation according to general procedure H using **50u**. Yield: 99%. Yellow solid. ^{1}H-NMR (500 MHz, DMSO-*d*$_6$): δ = 2.27 (s, 3H), 2.54 (t, ^3J = 7.6 Hz, 2H), 2.83 (t, ^3J = 7.6 Hz, 2H), 4.96 (d, ^4J = 0.9 Hz, 2H), 6.71 (s, 1H), 6.80 (dd, ^3J = 7.6 Hz, ^4J = 0.6 Hz, 1H), 7.01 (d, ^3J = 7.7 Hz, 1H), 7.19 (d, ^3J = 7.7 Hz, 1H), 7.23 - 7.31 (m, 2H), 7.32 (s, 1H), 7.53 - 7.63 (m, 3H), 7.89 (dd, ^3J = 7.8 Hz, ^4J = 1.0 Hz, 1H), 8.30 (d, ^3J = 8.3 Hz, 1H), 10.45 (s, 1H), 11.29 (s, 1H), 12.18 (br s, 1H) ppm. ^{13}C-NMR (126 MHz, DMSO-*d*$_6$): δ = 21.2, 30.4, 35.2, 64.2, 116.2, 118.3, 118.9, 120.9, 121.5, 122.7, 123.1, 123.3, 124.2, 125.9, 128.1, 128.6, 128.7, 129.0, 132.1, 138.3, 138.5, 141.4, 141.9, 154.2, 162.7, 167.2, 173.7 ppm. ESI-MS: *m/z* = 455.21 ([M-H]$^-$). HRMS (MALDI): *m/z* calculated 455.16015 for $C_{27}H_{23}N_2O_5$, found 455.15945 ([M-H]$^+$).

3-(3-[2-{8-Methyl-2H-chromene-3-carboxamido}benzamido]phenyl)propanoic acid (S8): Preparation according to general procedure H using **50v**. Yield: 88%. Yellow solid. ^{1}H-NMR (500 MHz, DMSO-*d*$_6$): δ = 2.14 (s, 3H), 2.54 (t, ^3J = 7.6 Hz, 2H), 2.83 (t, ^3J = 7.6 Hz, 2H), 5.01 (d, ^4J = 0.9 Hz, 2H), 6.87 (dd, ^3J = 7.5, 7.5 Hz, 1H), 7.02 (d, ^3J = 7.6 Hz, 1H), 7.11 - 7.18 (m, 2H), 7.23 - 7.32 (m, 2H), 7.33 (s, 1H), 7.53 - 7.65 (m, 3H), 7.90 (dd, ^3J = 7.8 Hz, ^4J = 0.9 Hz, 1H), 8.31 (d, ^3J = 8.2 Hz, 1H), 10.46 (s, 1H), 11.31 (s, 1H) ppm. ^{13}C-NMR (126 MHz, DMSO-*d*$_6$): 15.3, 30.4, 35.1, 64.2, 118.9, 120.4, 120.9, 121.3, 121.5, 123.1, 123.3, 124.2, 124.6, 126.6, 126.7, 128.4, 128.6, 129.0, 132.1, 132.9, 138.2, 138.5, 141.4, 152.3, 162.7, 167.2, 173.7 ppm. ESI-MS: *m/z* = 455.11 ([M-H]$^-$). HRMS (MALDI): *m/z* calculated 479.15774 for $C_{27}H_{24}N_2O_5Na$, found 479.15903 ([M+Na]$^+$).

3-(3-[2-{8-Chloro-2H-chromene-3-carboxamido}benzamido]phenyl)propanoic acid (S9): Preparation according to general procedure H using **50w**. Yield: 97%. White solid. ^{1}H-NMR (500 MHz, DMSO-*d*$_6$): δ = 2.54 (t, ^3J = 7.6 Hz, 2H), 2.83 (t, ^3J = 7.6 Hz, 2H), 5.12 (d, ^4J = 1.2 Hz, 2H), 6.98 (dd, ^3J = 7.8, 7.8 Hz, 1H), 7.01 (d, ^3J = 7.7 Hz, 1H), 7.25 - 7.32 (m, 3H), 7.37 (s, 1H), 7.39 (dd, ^3J = 8.0 Hz, ^4J = 1.4 Hz, 1H), 7.52 - 7.64 (m, 3H), 7.90 (d, ^3J = 7.8 Hz, 1H), 8.28 (d, ^3J = 8.1 Hz, 1H), 10.47 (s, 1H), 11.32 (s, 1H), 12.10 (br s, 1H) ppm. ^{13}C-NMR (126 MHz, DMSO-*d*$_6$): 30.4, 35.1, 65.0, 118.9, 119.8, 120.9, 121.7, 122.52, 122.54, 123.4, 123.5, 124.2, 127.2, 127.7, 128.0, 128.6, 129.0, 131.6, 132.1, 138.0, 138.5, 141.4, 149.7, 162.4, 167.1, 173.7 ppm. ESI-MS: *m/z* = 475.06 ([M-H]$^-$). HRMS (MALDI): *m/z* calculated 499.10312 for $C_{26}H_{21}ClN_2O_5Na$ found 499.10223 ([M+Na]$^+$).

3-(3-[2-{8-Methoxy-2H-chromene-3-carboxamido}benzamido]phenyl)propanoic acid (S10): Preparation according to general procedure H using **50x**. Yield: 97%. Yellow solid. ^{1}H-NMR (500 MHz, DMSO-*d*$_6$): δ = 2.54 (t, ^3J = 7.6 Hz, 2H), 2.83 (t, ^3J = 7.6 Hz, 2H), 3.77 (s, 3H), 4.96 (s, 2H), 6.87 - 6.95 (m, 2H), 6.99 - 7.04 (m, 2H), 7.23 - 7.30 (m, 2H), 7.33 (s, 1H), 7.53 - 7.63 (m, 3H), 7.89 (d, ^3J = 7.3 Hz, 1H), 8.29 (d, ^3J = 8.2 Hz, 1H), 10.45 (s, 1H), 11.31 (s, 1H), 12.11 (br s, 1H) ppm. ^{13}C-NMR (126 MHz, DMSO-*d*$_6$): δ = 30.4, 35.1, 55.7, 64.1, 114.9, 118.9, 120.6, 120.9, 121.6, 121.7, 123.2, 123.4, 124.2, 127.2, 128.1, 128.6, 129.0, 132.1, 138.2, 138.6, 141.4, 143.2, 147.6, 162.7, 167.2, 173.7 ppm. ESI-MS: *m/z* = 471.21 ([M-H]$^-$). HRMS (MALDI): *m/z* calculated 471.15506 for $C_{27}H_{23}N_2O_6$, found 471.15487 ([M-H]$^+$).

3-(3-[2-{8-Hydroxy-2H-chromene-3-carboxamido}benzamido]phenyl)propanoic acid (S11): Preparation according to general procedure H using **50y**. Yield: 85%. Yellow solid. ^{1}H-NMR (500 MHz, DMSO-d_6): δ = 2.53 (t, ^3J = 7.7 Hz, 2H), 2.82 (t, ^3J = 7.5 Hz, 2H), 4.96 (s, 2H), 6.72 - 6.87 (m, 3H), 7.01 (d, ^3J = 7.5 Hz, 1H), 7.21 - 7.30 (m, 2H), 7.32 (s, 1H), 7.48 - 7.65 (m, 3H), 7.90 (d, ^3J = 7.7 Hz, 1H), 8.31 (d, ^3J = 8.2 Hz, 1H), 10.48 (s, 1H), 11.34 (s, 1H) ppm. ^{13}C-NMR (126 MHz, DMSO-d_6): δ = 30.6, 35.5, 64.1, 118.78, 118.84, 119.2, 120.9, 121.5, 121.78, 121.82, 123.1, 123.3, 124.2, 127.0, 128.5, 128.6, 129.0, 132.1, 138.3, 138.5, 141.6, 142.0, 145.3, 162.8, 167.2, 173.9 ppm. ESI-MS: *m/z* = 457.16 ([M-H]$^-$). HRMS (MALDI): *m/z* calculated 481.13701 for $C_{26}H_{22}N_2O_6Na$, found 481.13635 ([M+Na]$^+$).

3-(3-[2-{(1,1'-Biphenyl)-4-carboxamido}benzamido]phenyl)propanoic acid (30): Preparation according to general procedure H using **50z**. Yield: 97%. Off-white solid. ^{1}H-NMR (500 MHz, DMSO-d_6): δ = 2.54 (t, ^3J = 7.6 Hz, 2H), 2.83 (t, ^3J = 7.5 Hz, 2H), 7.02 (d, ^3J = 7.5 Hz, 1H), 7.25 - 7.34 (m, 2H), 7.42 (dd, ^3J = 7.3, 7.3 Hz, 1H), 7.50 (dd, ^3J = 7.6, 7.6 Hz, 2H), 7.55 (s, 1H), 7.57 - 7.67 (m, 2H), 7.72 - 7.79 (m, 2H), 7.88 (d, ^3J = 8.2 Hz, 2H), 7.95 (d, ^3J = 7.8 Hz, 1H), 8.01 (d, ^3J = 8.3 Hz, 2H), 8.53 (d, ^3J = 8.3 Hz, 1H), 10.53 (s, 1H), 11.80 (s, 1H) ppm. ^{13}C-NMR (126 MHz, DMSO-d_6): δ = 30.5, 35.3, 119.1, 121.1, 121.3, 122.8, 123.3, 124.3, 127.0, 127.2, 127.8, 128.3, 128.6, 129.07, 129.10, 132.3, 133.2, 138.5, 138.8, 139.0, 141.5, 143.6, 164.3, 167.5, 173.8 ppm. ESI-MS: *m/z* = 463.15 ([M-H]$^-$). HRMS (MALDI): *m/z* calculated 487.16283 for $C_{29}H_{24}N_2O_4Na$, found 487.16123 ([M+Na]$^+$).

3-(3-[2-{4-(2-Furyl)benzamido}benzamido]phenyl)propanoic acid (31): Preparation according to general procedure H using **50aa**. Yield: 91%. Brown solid. ^{1}H-NMR (500 MHz, DMSO-d_6): δ = 2.55 (t, ^3J = 7.6 Hz, 2H), 2.83 (t, ^3J = 7.5 Hz, 2H), 6.66 (dd, ^3J = 3.3, 1.8 Hz, 1H), 7.02 (d, ^3J = 7.6 Hz, 1H), 7.14 (d, ^3J = 3.3 Hz, 1H), 7.25 - 7.34 (m, 2H), 7.55 (s, 1H), 7.59 (d, ^3J = 8.3 Hz, 1H), 7.62 (ddd, ^3J = 7.9, 7.9 Hz, ^4J = 1.2 Hz, 1H), 7.84 (dd, ^3J = 1.2 Hz, 1H), 7.89 (d, ^3J = 8.4 Hz, 2H), 7.93 (d, ^3J = 7.2 Hz, 1H), 7.97 (d, ^3J = 8.5 Hz, 2H), 8.48 (d, ^3J = 8.2 Hz, 1H), 10.51 (s, 1H), 11.73 (s, 1H), 12.15 (br s, 1H) ppm. ^{13}C-NMR (126 MHz, DMSO-d_6): δ = 30.4, 35.1, 108.1, 112.5, 119.0, 121.0, 121.3, 122.9, 123.3, 123.6, 124.2, 127.8, 128.6, 129.0, 132.3, 132.8, 133.4, 138.5, 138.7, 141.4, 144.0, 152.1, 164.1, 167.4, 173.7 ppm. ESI-MS: *m/z* = 453.16 ([M-H]$^-$). HRMS (MALDI): *m/z* calculated 477.14209 for $C_{27}H_{22}N_2O_5Na$, found 477.14108 ([M+Na]$^+$).

3-(3-[2-{4-(3-Furyl)benzamido}benzamido]phenyl)propanoic acid (32): Preparation according to general procedure H using **50ab**. Yield: 99%. Beige solid. ^{1}H-NMR (500 MHz, DMSO-d_6): δ = 2.55 (t, ^3J = 7.6 Hz, 2H), 2.84 (t, ^3J = 7.6 Hz, 2H), 7.03 (d, ^3J = 7.6 Hz, 1H), 7.06 (d, ^4J = 0.9 Hz, 1H), 7.29 (dd, ^3J = 7.8, 7.8 Hz, 2H), 7.55 (s, 1H), 7.58 - 7.66 (m, 2H), 7.79 (dd, ^4J = 1.6, 1.6 Hz, 1H), 7.83 (d, ^3J = 8.3 Hz, 2H), 7.94 (d, ^3J = 8.4 Hz, 3H), 8.33 (s, 1H), 8.51 (d, ^3J = 8.2 Hz, 1H), 10.51 (s, 1H), 11.76 (s, 1H), 12.15 (br s, 1H) ppm. ^{13}C-NMR (126 MHz, DMSO-d_6): δ = 30.4, 35.1, 108.7, 119.0, 121.0, 121.2, 122.7, 123.2, 124.3, 125.0, 125.8, 127.7, 128.6, 129.0, 132.3, 132.6, 135.7, 138.5, 138.8, 140.6, 141.4, 144.7, 164.2, 167.5, 173.7 ppm. ESI-MS: *m/z* = 453.08 ([M-H]$^-$). HRMS (MALDI): *m/z* calculated 477.14209 for $C_{27}H_{22}N_2O_5Na$, found 477.14124 ([M+Na]$^+$).

3-(3-[2-{4-(2-Thienyl)benzamido}benzamido]phenyl)propanoic acid (33): Preparation according to general procedure H using **50ac**. Yield: 39%. Yellow solid. ^{1}H-NMR (500 MHz, DMSO-d_6): δ = 2.55 (t, ^3J = 7.6 Hz, 2H), 2.84 (t, ^3J = 7.5 Hz, 2H), 7.03 (d, ^3J = 7.6 Hz, 1H), 7.19 (dd, ^3J = 4.9, 3.8 Hz, 1H), 7.26 - 7.32 (m, 2H), 7.55 (s, 1H), 7.58 - 7.66 (m, 3H), 7.68 (d, ^3J = 3.5 Hz, 1H), 7.87 (d, ^3J = 8.4 Hz, 2H), 7.91 - 7.99 (m, 3H), 8.50 (d, ^3J = 8.2 Hz, 1H), 10.51 (s, 1H), 11.76 (s, 1H), 12.15 (br s, 1H) ppm. ^{13}C-NMR (126 MHz, DMSO-d_6): δ = 30.4, 35.1, 119.1, 121.1, 121.3, 122.8, 123.3, 124.3, 125.3, 125.6, 127.2, 128.0, 128.6, 128.8, 129.0, 132.3, 133.0, 137.1, 138.5, 138.7, 141.4, 142.1, 164.0, 167.4, 173.7 ppm. ESI-MS: *m/z* = 469.06 ([M-H]$^-$). HRMS (MALDI): *m/z* calculated 493.11925 for $C_{27}H_{22}N_2O_4SNa$, found 493.11924 ([M+Na]$^+$).

3-(3-[2-{4-(3-Thienyl)benzamido}benzamido]phenyl)propanoic acid (34): Preparation according to general procedure H using **50ad**. Yield: 99%. Pale yellow solid. ^{1}H-NMR (500 MHz, DMSO-d_6):

δ = 2.54 (t, ^3J = 7.6 Hz, 2H), 2.83 (t, ^3J = 7.6 Hz, 2H), 7.02 (d, ^3J = 7.6 Hz, 1H), 7.26 - 7.33 (m, 2H), 7.54 (s, 1H), 7.57 - 7.65 (m, 2H), 7.65 - 7.71 (m, 2H), 7.91 - 7.99 (m, 5H), 8.06 (dd, ^3J = 2.8 Hz, ^4J = 1.3 Hz, 1H), 8.51 (d, ^3J = 8.3 Hz, 1H), 10.52 (s, 1H), 11.77 (s, 1H), 11.89 (br s, 1H) ppm. ^{13}C-NMR (126 MHz, DMSO-d_6): δ = 30.4, 35.2, 119.1, 121.1, 121.2, 122.7, 122.8, 123.2, 124.3, 126.2, 126.4, 127.5, 127.7, 128.6, 129.1, 132.3, 132.7, 138.43, 138.44, 138.8, 140.3, 141.5, 164.2, 167.5, 173.8 ppm. ESI-MS: *m/z* = 469.04 ([M-H]$^-$). HRMS (MALDI): *m/z* calculated 493.11925 for $C_{27}H_{22}N_2O_4SNa$, found 493.11832 ([M+Na]$^+$).

3-(3-[2-{4-(Isoxazol-3-yl)benzamido}benzamido]phenyl)propanoic acid (35): Preparation according to general procedure H using **50ae**. Yield: 95%. Yellow solid. ^{1}H-NMR (500 MHz, DMSO-d_6): δ = 2.55 (t, ^3J = 7.6 Hz, 2H), 2.83 (t, ^3J = 7.6 Hz, 2H), 7.02 (d, ^3J = 7.6 Hz, 1H), 7.25 (d, ^4J = 1.7 Hz, 1H), 7.26 - 7.35 (m, 2H), 7.55 (s, 1H), 7.57 - 7.67 (m, 2H), 7.94 (d, ^3J = 7.8 Hz, 1H), 8.05 (d, ^3J = 8.5 Hz, 2H), 8.10 (d, ^3J = 8.5 Hz, 2H), 8.47 (d, ^3J = 8.2 Hz, 1H), 9.07 (d, ^4J = 1.7 Hz, 1H), 10.51 (s, 1H), 11.78 (s, 1H), 12.16 (br s, 1H) ppm. ^{13}C-NMR (126 MHz, DMSO-d_6): δ = 30.4, 35.1, 103.2, 119.0, 121.0, 121.5, 123.2, 123.5, 124.3, 127.2, 127.9, 128.6, 129.1, 131.6, 132.3, 135.8, 138.5, 138.5, 141.4, 160.3, 161.2, 164.0, 167.4, 173.7 ppm. ESI-MS: *m/z* = 454.20 ([M-H]$^-$). HRMS (MALDI): *m/z* calculated 478.13734 for $C_{26}H_{21}N_3O_5Na$, found 478.13610 ([M+Na]$^+$).

3-(3-[2-{4-(Isoxazol-4-yl)benzamido}benzamido]phenyl)propanoic acid (36): Preparation according to general procedure H using **50af**. Yellow solid. ESI-MS: *m/z* = 454.17 ([M-H]$^-$). HRMS (MALDI): *m/z* calculated 478.13734 for $C_{26}H_{21}N_3O_5Na$, found 478.13706 ([M+Na]$^+$). NMR spectra indicated decomposition in DMSO solution.

3-(3-[2-{4-(Oxazol-4-yl)benzamido}benzamido]phenyl)propanoic acid (37): Preparation according to general procedure H using **50ag**. Yield: 68%. White solid. ^{1}H-NMR (500 MHz, DMSO-d_6): δ = 2.54 (t, ^3J = 7.6 Hz, 2H), 2.83 (t, ^3J = 7.6 Hz, 2H), 7.02 (d, ^3J = 7.7 Hz, 1H), 7.24 - 7.34 (m, 2H), 7.55 (s, 1H), 7.57 - 7.66 (m, 2H), 7.93 (dd, ^3J = 7.9 Hz, ^4J = 1.0 Hz, 1H), 7.99 (s, 4H), 8.47 (d, ^3J = 8.2 Hz, 1H), 8.53 (d, ^4J = 0.7 Hz, 1H), 8.77 (d, ^4J = 0.7 Hz, 1H), 10.50 (s, 1H), 11.72 (s, 1H), 12.07 (br s, 1H) ppm. ^{13}C-NMR (126 MHz, DMSO-d_6): δ = 30.4, 35.1, 119.0, 121.0, 121.4, 123.0, 123.4, 124.2, 125.5, 127.8, 128.6, 129.0, 132.3, 133.7, 134.2, 136.5, 138.4, 138.5, 138.6, 141.4, 153.0, 164.2, 167.4, 173.7 ppm. ESI-MS: *m/z* = 454.24 ([M-H]$^-$). HRMS (MALDI): *m/z* calculated 478.13734 for $C_{26}H_{21}N_3O_5Na$, found 478.13672 ([M+Na]$^+$).

3-(3-[2-{4-(Oxazol-5-yl)benzamido}benzamido]phenyl)propanoic acid (38): Preparation according to general procedure H using **50ah**. Yield: 20%. White solid. ^{1}H-NMR (500 MHz, DMSO-d_6): δ = 2.54 (t, ^3J = 7.6 Hz, 2H), 2.83 (t, ^3J = 7.5 Hz, 2H), 7.02 (d, ^3J = 7.5 Hz, 1H), 7.26 - 7.34 (m, 2H), 7.55 (s, 1H), 7.59 (d, ^3J = 8.3 Hz, 1H), 7.63 (dd, ^3J = 7.9, 7.9 Hz, 1H), 7.86 (s, 1H), 7.93 (d, ^3J = 8.3 Hz, 3H), 8.02 (d, ^3J = 8.4 Hz, 2H), 8.46 (d, ^3J = 8.2 Hz, 1H), 8.54 (s, 1H), 10.51 (s, 1H), 11.74 (s, 1H), 12.21 (br s, 1H) ppm. ^{13}C-NMR (126 MHz, DMSO-d_6): δ = 30.4, 35.1, 119.0, 121.0, 121.5, 123.1, 123.4, 123.9, 124.2, 124.4, 128.0, 128.6, 129.0, 130.5, 132.2, 134.0, 138.5, 138.5, 141.4, 149.7, 152.6, 163.9, 167.4, 173.7 ppm. ESI-MS: *m/z* = 454.16 ([M-H]$^-$). HRMS (MALDI): *m/z* calculated 478.13734 for $C_{26}H_{21}N_3O_5Na$, found 478.13721 ([M+Na]$^+$).

3-(3-[2-{4-(1H-1,2,3-Triazol-1-yl)benzamido}benzamido]phenyl)propanoic acid (39): Preparation according to general procedure H using **50ai**. Yield: 93%. White solid. ^{1}H-NMR (500 MHz, DMSO-d_6): δ = 2.54 (t, ^3J = 7.6 Hz, 2H), 2.83 (t, ^3J = 7.6 Hz, 2H), 7.02 (d, ^3J = 7.6 Hz, 1H), 7.28 (t, ^3J = 7.8 Hz, 1H), 7.32 (ddd, ^3J = 7.6, 7.6 Hz, ^4J = 1.0 Hz, 1H), 7.55 (s, 1H), 7.59 (d, ^3J = 8.2 Hz, 1H), 7.61 - 7.67 (m, 1H), 7.94 (dd, ^3J = 7.7 Hz, ^4J = 1.0 Hz, 1H), 8.03 (d, ^4J = 1.1 Hz, 1H), 8.11 - 8.17 (m, 4H), 8.44 (d, ^3J = 8.2 Hz, 1H), 8.94 (d, ^4J = 1.1 Hz, 1H), 10.51 (s, 1H), 11.75 (s, 1H), 12.14 (br s, 1H) ppm. ^{13}C-NMR (126 MHz, DMSO-d_6) δ = 30.4, 35.1, 119.0, 120.2, 121.0, 121.6, 123.4, 123.4, 123.6, 124.2, 128.6, 128.9, 129.1, 132.2, 134.2, 134.8, 138.4, 138.5, 139.0, 141.4, 163.6, 167.3, 173.7 ppm. ESI-MS: *m/z* = 454.17 ([M-H]$^-$). HRMS (MALDI): *m/z* calculated 478.14858 for $C_{25}H_{21}N_5O_4Na$, found 478.14793 ([M+Na]$^+$).

(3-[2-{4-(3-Furyl)benzamido}-4-methoxybenzamido]phenyl)glycine (40): Preparation according to general procedure H using **50an** and purified by HPLC. Yield: 36%. Pale yellow solid. ^{1}H-NMR (500 MHz, DMSO-d_6): δ = 3.79 (s, 2H), 3.87 (s, 3H), 6.36 (dd, ^3J = 8.0 Hz, ^4J = 1.5 Hz, 1H), 6.83 (dd, ^3J = 8.8 Hz, ^4J = 2.6 Hz, 1H), 6.91 - 6.98 (m, 2H), 7.03 - 7.12 (m, 2H), 7.79 (dd, ^4J = 1.5, 1.5 Hz, 1H), 7.85 (d, ^3J = 8.4 Hz, 2H), 7.91 - 8.02 (m, 3H), 8.33 (s, 1H), 8.37 (d, ^4J = 2.6 Hz, 1H), 10.20 (s, 1H), 12.53 (s, 1H) ppm. ^{13}C-NMR (126 MHz, DMSO-d_6): δ = 44.7, 55.5, 105.4, 105.5, 108.37, 108.40, 108.7, 109.9, 113.2, 125.0, 125.9, 127.6, 128.9, 130.7, 132.5, 135.7, 139.0, 140.6, 141.6, 144.7, 148.6, 162.3, 164.2, 167.5, 172.6 ppm. ESI-MS: *m/z* = 484.01 ([M-H]$^-$). HRMS (MALDI): *m/z* calculated 508.14791 for $C_{27}H_{23}N_3O_6Na$, found 508.14649 ([M+Na]$^+$).

3-(3-[4-{tert-Butyl}-2-{4-(2-furyl)}benzamido]phenyl)propanoic acid (41): Preparation according to general procedure H using **50aj**. Yield: 99%. Beige solid. ^{1}H-NMR (500 MHz, DMSO-d_6): δ = 1.35 (s, 9H), 2.55 (t, ^3J = 7.6 Hz, 2H), 2.83 (t, ^3J = 7.6 Hz, 2H), 6.65 (dd, ^3J = 3.4 Hz, ^4J = 1.8 Hz, 1H), 7.02 (d, ^3J = 7.6 Hz, 1H), 7.13 (dd ^3J = 3.3 Hz, 1H), 7.28 (dd, ^3J = 7.8, 7.8 Hz, 1H), 7.32 (dd, ^3J = 8.3 Hz, ^4J = 1.9 Hz, 1H), 7.55 (s, 1H), 7.58 (d, ^3J = 8.8 Hz, 1H), 7.83 (d, ^4J = 1.5 Hz, 1H), 7.85 - 7.92 (m, 3H), 7.98 (d, ^3J = 8.5 Hz, 2H), 8.64 (d, ^4J = 1.9 Hz, 1H), 10.42 (s, 1H), 11.84 (s, 1H), 12.12 (br s, 1H) ppm. ^{13}C-NMR (126 MHz, DMSO-d_6): δ = 30.4, 30.8, 34.9, 35.1, 108.0, 112.4, 118.0, 119.0, 119.8, 120.3, 120.9, 123.6, 124.1, 127.8, 128.6, 128.8, 132.9, 133.3, 138.5, 138.8, 141.4, 144.0, 152.1, 155.4, 164.1, 167.4, 173.6 ppm. ESI-MS: *m/z* = 509.06 ([M-H]$^-$). HRMS (MALDI): *m/z* calculated 533.20469 for $C_{31}H_{30}N_2O_5Na$, found 533.20371 ([M+Na]$^+$).

Ethyl 3-(3-aminophenyl)propanoate (43a): Preparation according to general procedure A using **42a**. Yield: 93%. Brown oil. ^{1}H-NMR (400 MHz, $CDCl_3$): δ = 1.24 (t, ^3J = 7.1 Hz, 3H), 2.59 (t, ^3J = 7.9 Hz, 2H), 2.86 (t, ^3J = 7.9 Hz, 2H), 3.62 (br s, 2H), 4.13 (q, ^3J = 7.1 Hz, 2H), 6.50 - 6.56 (m, 2H), 6.60 (d, ^3J = 7.8 Hz, 1H), 7.07 (dd, ^3J = 8.1, 8.1 Hz, 1H) ppm. ^{13}C-NMR (75 MHz, $CDCl_3$): δ = 14.4, 31.1, 36.0, 65.0, 113.2, 115.2, 118.7, 129.5, 142.0, 146.6, 173.2 ppm.

Ethyl 3-(4-aminophenyl)propanoate (43b): Preparation according to general procedure A using **42b**. Yield: 94%. Brown liquid. ^{1}H-NMR (400 MHz, $CDCl_3$): δ = 1.23 (t, ^3J = 7.1 Hz, 3H), 2.56 (t, ^3J = 7.8 Hz, 2H), 2.84 (t, ^3J = 7.8 Hz, 2H), 3.55 (s, 2H), 4.12 (q, ^3J = 7.1 Hz, 2H), 6.62 (d, ^3J = 8.4 Hz, 2H), 6.99 (d, ^3J = 8.4 Hz, 2H) ppm. ^{13}C-NMR (101 MHz, $CDCl_3$): δ = 14.4, 30.3, 36.5, 60.4, 115.4, 129.3, 130.8, 144.7, 173.3 ppm.

Ethyl 2-aminophenylacetate (43c): Preparation according to general procedure A using **52**. Yield: 39%. Brown liquid. ^{1}H-NMR (400 MHz, $CDCl_3$): δ = 1.26 (t, ^3J = 7.1 Hz, 3H), 3.56 (s, 2H), 4.09 (br s, 2H), 4.15 (q, ^3J = 7.1 Hz, 2H), 6.69-6.73 (m, 1H), 6.75 (ddd, ^3J = 7.5, 7.5 Hz, ^4J = 1.1 Hz, 1H), 7.05-7.14 (m, 2H) ppm. ^{13}C-NMR (101 MHz, $CDCl_3$): δ = 14.2, 38.7, 61.2, 116.6, 119.0, 119.7, 128.6, 131.2, 145.6, 172.0 ppm.

Ethyl 3-aminophenoxyacetate (43d): Preparation according to general procedure D using **55**. Yield: 86%. Brown oil. ^{1}H-NMR (300 MHz, $CDCl_3$): δ = 1.29 (t, ^3J = 7.1 Hz, 3H), 3.24 (br s, 2H), 4.26 (q, ^3J = 7.1 Hz, 2H), 4.57 (s, 2H), 6.29 - 6.39 (m, 3H), 7.02 - 7.10 (m, 1H) ppm. ^{13}C-NMR (75 MHz, $CDCl_3$): δ = 14.3, 61.5, 65.6, 102.5, 104.8, 109.4, 130.4, 147.4, 159.2, 169.2 ppm.

Ethyl 3-aminophenylglycinate (43e): Preparation according to general procedure I using **56** and **54a**. Yield: 75%. Brown oil. ^{1}H-NMR (300 MHz, $CDCl_3$): δ = 1.30 (t, ^3J = 7.1 Hz, 3H), 3.73 (br s, 3H), 3.86 (s, 2H), 4.24 (q, ^3J = 7.1 Hz, 2H), 5.94 (dd, ^4J = 2.2, 2.2 Hz, 1H), 6.05 (ddd, ^3J = 8.0 Hz, ^4J = 2.2, 0.7 Hz, 1H), 6.11 (ddd, ^3J = 7.9 Hz, ^4J = 2.1, 0.7 Hz, 1H), 6.97 (dd, ^3J = 7.9, 7.9 Hz, 1H) ppm. ^{13}C-NMR (75 MHz, $CDCl_3$): δ = 14.3, 45.9, 61.3, 99.9, 104.0, 105.8, 130.2, 147.6, 148.3, 171.3 ppm.

Ethyl 2-(3-aminophenyl)-2-methylpropanoate (43f): Preparation according to general procedure I using **56** and **54b**. Yield: 50%. Brown oil. ^{1}H-NMR (300 MHz, $CDCl_3$): δ = 1.19 (t, ^3J = 7.1 Hz, 3H), 1.54 (s, 6H), 3.98 (br s, 3H), 4.17 (q, ^3J = 7.1 Hz, 2H), 5.99 (dd, ^4J = 2.2, 2.2 Hz, 1H), 6.05 (ddd, ^3J = 8.0 Hz, ^4J = 2.2, 0.7 Hz, 1H), 6.15 (ddd, ^3J = 7.9 Hz, ^4J = 2.1, 0.7 Hz, 1H), 6.93 (dd, ^3J = 7.9,

7.9 Hz, 1H) ppm. ^{13}C-NMR (75 MHz, $CDCl_3$): δ = 14.3, 26.4, 57.7, 61.4, 102.9, 106.6, 107.1, 130.0, 146.4, 146.7, 176.3 ppm.

2-Nitro-4-(trifluoromethoxy)benzoic acid (44g): Preparation according to general procedure K using **58**. Yield: 45%. Off-white solid. ^{1}H-NMR (300 MHz, DMSO-d_6) δ = 7.74 (m, 1H), 7.96 (d, ^{3}J = 8.5 Hz, 1H), 8.02 (d, ^{4}J = 1.7 Hz, 1H). ^{13}C-NMR (75 MHz, DMSO-d_6) δ = 116.4, 119.8, 124.5, 128.1, 132.3, 149.0, 149.5, 164.8 ppm.

4-(tert-Butyl)-2-nitrobenzoic acid (44h): Preparation according to general procedure M using **60**. Yield: 51%. Pale yellow solid. ^{1}H-NMR (300 MHz, $CDCl_3$): δ = 1.32 (s, 9H), 7.79 - 7.82 (m, 2H), 7.90 (dd, ^{4}J = 1.3, 0.9 Hz, 1H), 13.71 (br s, 1H) ppm. ^{13}C-NMR (75 MHz, $CDCl_3$): δ = 30.5, 35.2, 120.4, 123.8, 129.5, 130.0, 149.1, 156.3, 165.5 ppm.

6-Nitrobenzo[1,3]dioxole-5-carboxylic acid (44l): Preparation according to general procedure L using **61**. Yield: 76%. Yellow solid. ^{1}H-NMR (300 MHz, DMSO-d_6): δ = 6.28 (s, 2H), 7.30 (s, 1H), 7.62 (s, 1H), 13.63 (br s, 1H) ppm. ^{13}C-NMR (75 MHz, DMSO-d_6): δ = 103.9, 104.6, 108.2, 123.5, 143.1, 149.5, 150.8, 165.7 ppm.

Ethyl 3-(3-[3-methyl-2-nitrobenzamido]phenyl)propanoate (46a): Preparation according to general procedure B using **44a** and **43a**. Yield: 53%. Brown solid. ^{1}H-NMR (300 MHz, $CDCl_3$): δ = 1.22 (t, ^{3}J = 7.1 Hz, 3H), 2.37 (s, 3H), 2.58 (t, ^{3}J = 7.8 Hz, 2H), 2.89 (t, ^{3}J = 7.8 Hz, 2H), 4.10 (q, ^{3}J = 7.1 Hz, 2H), 6.97 (d, ^{3}J = 7.6 Hz, 1H), 7.22 (dd, ^{3}J = 7.8, 7.8 Hz, 1H), 7.30 - 7.58 (m, 5H), 7.99 (s, 1H) ppm. ^{13}C-NMR (75 MHz, $CDCl_3$): δ = 14.3, 17.8, 30.9, 35.8, 60.6, 118.3, 120.3, 125.1, 125.7, 129.3, 130.4, 130.7, 131.6, 134.1, 137.6, 141.8, 149.4, 163.4, 173.0 ppm.

Ethyl 3-(3-[4-methyl-2-nitrobenzamido]phenyl)propanoate (46b): Preparation according to general procedure B using **44b** and **43a**. Yield: 66%. Yellow oil. ^{1}H-NMR (300 MHz, $CDCl_3$): δ = 1.23 (t, ^{3}J = 7.1 Hz, 3H), 2.48 (s, 3H), 2.61 (t, ^{3}J = 7.8 Hz, 2H), 2.93 (t, ^{3}J = 7.7 Hz, 2H), 4.11 (q, ^{3}J = 7.1 Hz, 2H), 7.00 (d, ^{3}J = 7.5 Hz, 1H), 7.26 (dd, ^{3}J = 7.6, 7.6 Hz, 1H), 7.36 – 7.53 (m, 4H), 7.65 (s, 1H), 7.86 (s, 1H) ppm. ^{13}C-NMR (75 MHz, $CDCl_3$): δ = 14.3, 21.3, 31.0, 35.9, 60.6, 118.4, 120.4, 125.1, 128.6, 129.4, 130.3, 134.4, 137.7, 141.9, 146.6, 164.6, 173.0 ppm.

Ethyl 3-(3-[5-methyl-2-nitrobenzamido]phenyl)propanoate (46c): Preparation according to general procedure B with dicyclohexylcarbodiimide instead of EDC using **44c** and **43a**. Yield: 58%. Yellow oil. ^{1}H-NMR (300 MHz, $CDCl_3$): δ = 1.24 (t, ^{3}J = 7.1 Hz, 3H), 2.49 (s, 3H), 2.64 (t, ^{3}J = 7.7 Hz, 2H), 2.96 (t, ^{3}J = 7.7 Hz, 2H), 4.13 (q, ^{3}J = 7.1 Hz, 2H), 7.03 (d, ^{3}J = 7.5 Hz, 1H), 7.29 (dd, ^{3}J = 7.8, 7.6 Hz, 1H), 7.35 – 7.52 (m, 5H), 8.04 (d, ^{3}J = 8.0 Hz, 1H) ppm. ^{13}C-NMR (75 MHz, $CDCl_3$): δ = 14.4, 21.6, 31.0, 35.9, 60.6, 118.5, 120.5, 125.0, 125.3, 129.3, 129.4, 131.3, 133.2, 137.6, 142.0, 144.1, 145.8, 164.7, 173.0 ppm.

Ethyl 3-(3-[2-methyl-6-nitrobenzamido]phenyl)propanoate (46d): Preparation according to general procedure B using **44d** and **43a**. Yield: 17%. Brown oil. ^{1}H-NMR (300 MHz, $CDCl_3$): δ = 1.22 (t, ^{3}J = 7.1 Hz, 3H), 2.45 (s, 3H), 2.59 (t, ^{3}J = 7.8 Hz, 2H), 2.91 (t, ^{3}J = 7.8 Hz, 2H), 4.09 (q, ^{3}J = 7.1 Hz, 2H), 7.00 (d, ^{3}J = 7.7 Hz, 1H), 7.25 (dd, ^{3}J = 7.8, 7.8 Hz, 1H), 7.32 - 7.47 (m, 3H), 7.52 (d, ^{3}J = 7.3 Hz, 1H), 7.86 (s, 1H), 7.94 (d, ^{3}J = 8.1 Hz, 1H) ppm. ^{13}C-NMR (75 MHz, $CDCl_3$): δ = 14.3, 19.1, 31.0, 35.9, 60.6, 118.7, 120.6, 122.1, 125.1, 129.3, 129.7, 132.4, 136.3, 137.7, 138.0, 141.9, 145.8, 164.7, 173.1 ppm.

Ethyl 3-(3-[4-chloro-2-nitrobenzamido]phenyl)propanoate (46e): Preparation according to general procedure B using **44e** and **43a**. Yield: 62%. Brown oil. ^{1}H-NMR (300 MHz, $CDCl_3$): δ = 1.23 (t, ^{3}J = 7.1 Hz, 3H), 2.59 (t, ^{3}J = 7.7 Hz, 2H), 2.91 (t, ^{3}J = 7.7 Hz, 2H), 4.10 (q, ^{3}J = 7.1 Hz, 2H), 7.01 (d, ^{3}J = 7.5 Hz, 1H), 7.24 (dd, ^{3}J = 7.7, 7.7 Hz, 1H), 7.31 - 7.43 (m, 2H), 7.53 (d, ^{3}J = 8.2 Hz, 1H), 7.64 (dd, ^{3}J = 8.2 Hz, ^{4}J = 1.9 Hz, 1H), 7.91 (s, 1H), 8.01 (d, ^{4}J = 1.9 Hz, 1H) ppm. ^{13}C-NMR (75 MHz, $CDCl_3$): δ = 14.3, 30.9, 35.8, 60.7, 118.6, 120.5, 125.0, 125.4, 129.4, 129.9, 131.2, 133.9, 136.8, 137.4, 141.9, 147.0, 163.5, 173.0 ppm.

Ethyl 3-(3-[4-methoxy-2-nitrobenzamido]phenyl)propanoate (46f): Preparation according to general procedure B using **44f** and **43a**. Yield: 82%. Beige solid. ^{1}H-NMR (300 MHz, $CDCl_3$): δ = 1.24 (t, ^3J = 7.1 Hz, 3H), 2.61 (t, ^3J = 7.6 Hz, 2H), 2.93 (t, ^3J = 7.7 Hz, 2H), 3.91 (s, 3H), 4.12 (q, ^3J = 7.1 Hz, 2H), 7.00 (d, ^3J = 7.4 Hz, 1H), 7.17 (dd, ^3J = 8.5 Hz, ^4J = 2.4 Hz, 1H), 7.23 - 7.31 (m, 1H), 7.47 (m, 4H), 7.63 (s, 1H) ppm. ^{13}C-NMR (75 MHz, $CDCl_3$): δ = 14.3, 31.0, 35.9, 56.3, 60.6, 109.9, 118.4, 119.4, 120.3, 125.1, 129.4, 129.9, 130.0, 137.8, 141.9, 148.0, 161.1, 164.3, 173.0 ppm.

Ethyl 3-(3-[2-nitro-4-{trifluoromethoxy}benzamido]phenyl)propanoate (46g): Preparation according to general procedure B using **44g** and **43a**. Yield: 61%. Yellow oil. ^{1}H-NMR (400 MHz, $CDCl_3$): δ = 1.22 (t, ^3J = 7.1 Hz, 3H), 2.60 (t, ^3J = 7.7 Hz, 2H), 2.92 (t, ^3J = 7.7 Hz, 2H), 4.10 (q, ^3J = 7.1 Hz, 2H), 7.01 (d, ^3J = 7.6 Hz, 1H), 7.22 - 7.30 (m, 1H), 7.38 (d, ^3J = 8.2 Hz, 1H), 7.41 (s, 1H), 7.54 (d, ^3J = 8.3 Hz, 1H), 7.67 (d, ^3J = 8.4 Hz, 1H), 7.91 (m, 2H) ppm. ^{13}C-NMR (75 MHz, $CDCl_3$): δ = 14.3, 31.0, 35.8, 60.7, 117.4, 118.6, 120.3, 120.5, 125.5, 125.9, 129.4, 130.5, 131.3, 137.4, 142.0, 147.3, 150.0, 163.3, 173.0 ppm.

Ethyl 3-(3-[4-{tert-butyl}-2-nitrobenzamido]phenyl)propanoate (46h): Preparation according to general procedure B using **44h** and **43a**. Yield: 80%. Brown solid. ^{1}H-NMR (300 MHz, $CDCl_3$): δ = 1.23 (t, ^3J = 7.1 Hz, 3H), 1.37 (s, 9H), 2.61 (t, ^3J = 7.7 Hz, 2H), 2.93 (t, ^3J = 7.7 Hz, 2H), 4.11 (q, ^3J = 7.1 Hz, 2H), 7.00 (d, ^3J = 7.6 Hz, 1H), 7.26 (dd, ^3J = 7.8, 7.8 Hz, 1H), 7.37 - 7.49 (m, 2H), 7.54 (d, ^3J = 8.0 Hz, 1H), 7.70 (dd, ^3J = 8.0 Hz, ^4J = 1.6 Hz, 1H), 7.74 (s, 1H), 8.07 (d, ^4J = 1.5 Hz, 1H) ppm. ^{13}C-NMR (75 MHz, $CDCl_3$): δ = 14.3, 31.0, 31.1, 35.4, 35.9, 60.7, 118.4, 120.4, 121.8, 125.1, 128.5, 129.4, 130.2, 131.0, 137.8, 141.9, 146.6, 155.3, 164.7, 173.1 ppm.

Ethyl 3-(3-[5-chloro-2-nitrobenzamido]phenyl)propanoate (46i): Preparation according to general procedure B using **44i** and **43a**. Yield: 45%. Brown oil. ^{1}H-NMR (300 MHz, $CDCl_3$): δ = 1.20 (t, ^3J = 7.1 Hz, 3H), 2.52 (t, ^3J = 7.8 Hz, 2H), 2.82 (t, ^3J = 7.8 Hz, 2H), 4.05 (q, ^3J = 7.1 Hz, 2H), 6.94 (d, ^3J = 7.6 Hz, 1H), 7.17 (dd, ^3J = 7.7, 7.7 Hz, 1H), 7.24 - 7.33 (m, 2H), 7.40 - 7.47 (m, 2H), 7.90 (d, ^3J = 8.5 Hz, 1H), 8.58 (s, 1H) ppm. ^{13}C-NMR (75 MHz, $CDCl_3$): δ = 14.2, 30.8, 35.7, 60.6, 118.6, 120.5, 125.2, 126.0, 128.9, 129.2, 130.5, 134.2, 137.4, 140.4, 141.6, 144.3, 163.4, 173.1 ppm.

Ethyl 3-(3-[5-methoxy-2-nitrobenzamido]phenyl)propanoate (46j): Preparation according to general procedure B using **44j** and **43a**. Yield: 40%. Yellow oil. ^{1}H-NMR (300 MHz, $CDCl_3$): δ = 1.20 (t, ^3J = 7.1 Hz, 3H), 2.52 (t, ^3J = 7.8 Hz, 2H), 2.82 (t, ^3J = 7.8 Hz, 2H), 3.81 (s, 3H), 4.06 (q, ^3J = 7.1 Hz, 2H), 6.82 - 6.90 (m, 2H), 6.92 (d, ^3J = 7.6 Hz, 1H), 7.16 (dd, ^3J = 7.8, 7.8 Hz, 1H), 7.27 - 7.37 (m, 2H), 7.92 (d, ^3J = 9.1 Hz, 1H), 8.47 (s, 1H) ppm. ^{13}C-NMR (75 MHz, $CDCl_3$): δ = 14.2, 30.8, 35.7, 56.3, 60.6, 113.6, 115.2, 118.5, 120.3, 124.8, 127.0, 129.1, 135.3, 137.8, 138.4, 141.5, 163.8, 164.9, 173.0 ppm.

Ethyl 3-(3-[2-nitro-5-{trifluoromethoxy}benzamido]phenyl)propanoate (46k): Preparation according to general procedure B using **44k** and **43a**. Yield: 68%. Yellow solid. ^{1}H-NMR (300 MHz, $CDCl_3$): δ = 1.23 (t, ^3J = 7.1 Hz, 3H), 2.61 (t, ^3J = 7.7 Hz, 2H), 2.93 (t, ^3J = 7.7 Hz, 2H), 4.10 (q, ^3J = 7.1 Hz, 2H), 7.03 (d, ^3J = 7.6 Hz, 1H), 7.27 (dd, ^3J = 7.7, 7.7 Hz, 1H), 7.36 - 7.47 (m, 4H), 7.78 (s, 1H), 8.18 (d, ^3J = 8.4 Hz, 1H) ppm. ^{13}C-NMR (75 MHz, $CDCl_3$): δ = 14.3, 31.0, 35.8, 60.7, 118.6, 120.2, 120.6, 125.6, 127.2, 129.5, 135.3, 137.3, 142.0, 143.8, 152.7, 162.9, 173.0 ppm.

Ethyl 3-(3-[6-nitrobenzo[1,3]dioxole-5-carboxamido]phenyl)propanoate (46l): Preparation according to general procedure B using **44l** and **43a**. Yield: 59%. Brown solid. ^{1}H-NMR (300 MHz, $CDCl_3$): δ = 1.23 (t, ^3J = 7.1 Hz, 3H), 2.61 (t, ^3J = 7.8 Hz, 2H), 2.93 (t, ^3J = 7.7 Hz, 2H), 4.12 (q, ^3J = 7.1 Hz, 2H), 6.17 (s, 2H), 6.94 (s, 1H), 7.00 (d, ^3J = 7.5 Hz, 1H), 7.26 (dd, ^3J = 7.7, 7.7 Hz, 1H), 7.34 - 7.46 (m, 2H), 7.54 (s, 1H), 7.63 (s, 1H) ppm. ^{13}C-NMR (75 MHz, $CDCl_3$): δ = 14.3, 31.0, 35.9, 60.6, 103.8, 105.4, 107.8, 118.5, 120.5, 125.2, 129.4, 137.6, 140.7, 142.0, 149.0, 152.4, 164.2, 173.0 ppm.

Ethyl 2-(2-nitrobenzamido)phenylacetate (46m): Preparation according to general procedure C using **45** and **43c**. Yield: 77%. Beige solid. ^{1}H-NMR (400 MHz, $CDCl_3$): δ = 1.21 (t, ^3J = 7.1 Hz, 3H),

3.71 (s, 2H), 4.10 (q, ^3J = 7.1 Hz, 2H), 7.19 (t, ^3J = 7.2 Hz, 1H), 7.23 - 7.28 (m, 1H), 7.39 (dd, ^3J = 7.7, 7.7 Hz, 1H), 7.58 - 7.67 (m, 1H), 7.74 (d, ^3J = 3.7 Hz, 2H), 7.99 (d, ^3J = 8.0 Hz, 1H), 8.11 (d, ^3J = 8.1 Hz, 1H), 9.28 (s, 1H) ppm. ^{13}C-NMR (101 MHz, $CDCl_3$): δ = 14.1, 39.0, 61.9, 124.8, 125.5, 126.2, 126.7, 128.7, 128.8, 130.8, 131.1, 133.2, 134.0, 136.2, 146.8, 164.9, 173.2 ppm.

Ethyl 3-(3-[2-nitrobenzamido]phenyl)propanoate (46n): Preparation according to general procedure C using **45** and **43a**. Yield: 99% yield. Brown oil. ^{1}H-NMR (400 MHz, $CDCl_3$): δ = 1.23 (t, ^3J = 7.0 Hz, 3H), 2.61 (t, ^3J = 7.8 Hz, 2H), 2.93 (t, ^3J = 7.7 Hz, 2H), 4.11 (q, ^3J = 7.1 Hz, 2H), 7.01 (t, ^3J = 7.6 Hz, 1H), 7.27 (dd, ^3J = 7.8, 7.8 Hz, 1H), 7.41 (d, ^3J = 8.1 Hz, 1H), 7.45 (br s, 1H), 7.56 - 7.65 (m, 2H), 7.66 - 7.76 (m, 2H), 8.08 (d, ^3J = 8.1 Hz, 1H) ppm. ^{13}C-NMR (75 MHz, $CDCl_3$): δ = 14.3, 31.0, 35.9, 60.7, 118.5, 120.4, 124.8, 125.2, 128.8, 129.4, 130.9, 133.0, 134.0, 137.7, 141.9, 146.4, 164.5, 173.0 ppm.

Ethyl 3-(4-[2-nitrobenzamido]phenyl)propanoate (46o): Preparation according to general procedure C using **45** and **43b**. Yield: 94%. Brown solid. ^{1}H-NMR (400 MHz, $CDCl_3$): δ = 1.14 (t, ^3J = 7.1 Hz, 3H), 2.50 (t, ^3J = 7.7 Hz, 2H), 2.83 (t, ^3J = 7.7 Hz, 2H), 4.02 (q, ^3J = 7.1 Hz, 2H), 7.09 (d, ^3J = 8.3 Hz, 2H), 7.38 (d, ^3J = 8.3 Hz, 2H), 7.45-7.55 (m, 2H), 7.55-7.64 (m, 2H), 7.97 (d, ^3J = 8.1 Hz, 1H) ppm. ^{13}C-NMR (101 MHz, $CDCl_3$): δ = 14.3, 30.5, 36.0, 60.6, 120.8, 124.8, 128.8, 129.1, 130.9, 133.0, 134.0, 135.7, 137.7, 146.5, 164.5, 173.0 ppm.

Ethyl 3-(2-nitrobenzamido)phenoxyacetate (46p): Preparation according to general procedure B using **44m** and **43d**. Yield: 66%. Yellow oil. ^{1}H-NMR (300 MHz, $CDCl_3$): δ = 1.28 (t, ^3J = 7.1 Hz, 3H), 4.24 (q, ^3J = 7.1 Hz, 2H), 4.59 (s, 2H), 6.70 (dd, ^3J = 8.2 Hz, ^4J = 1.8 Hz, 1H), 7.10 (d, ^3J = 8.1 Hz, 1H), 7.22 (dd, ^3J = 8.2, 8.2 Hz, 1H), 7.31 (s, 1H), 7.54 - 7.63 (m, 2H), 7.67 (d, ^3J = 6.9 Hz, 1H), 7.97 (s, 1H), 8.06 (d, ^3J = 8.0 Hz, 1H) ppm. ^{13}C-NMR (75 MHz, $CDCl_3$): δ = 14.3, 61.6, 65.6, 107.1, 111.5, 113.7, 124.7, 128.8, 130.1, 130.9, 132.9, 134.0, 138.9, 146.4, 158.4, 164.6, 169.0 ppm.

Ethyl (3-[2-nitrobenzamido]phenyl)glycinate (46q): Preparation according to general procedure B using **44m** and **43e**. Yield: 93%. Yellow oil. ^{1}H-NMR (500 MHz, $CDCl_3$): δ = 1.30 (t, ^3J = 7.1 Hz, 3H), 3.93 (s, 2H), 4.24 (q, ^3J = 7.1 Hz, 2H), 6.47 (d, ^3J = 7.7 Hz, 1H), 6.83 (d, ^3J = 7.7 Hz, 1H), 7.12 - 7.21 (m, 2H), 7.57 (br s, 1H), 7.59 - 7.66 (m, 2H), 7.72 (d, ^3J = 7.4 Hz, 1H), 8.11 (d, ^3J = 8.2 Hz, 1H), ppm. ^{13}C-NMR (126 MHz, $CDCl_3$): δ = 14.3, 46.2, 61.7, 105.6, 110.3, 110.6, 124.9, 128.7, 130.1, 130.9, 133.1, 134.0, 138.6, 146.5, 147.4, 164.4, 170.8 ppm.

Ethyl 2-methyl-2-([3-{2-nitrobenzamido}phenyl]amino)propanoate (46r): Preparation according to general procedure B using **44m** and **43f**. Yield: 54%. Brown oil. ^{1}H-NMR (300 MHz, $CDCl_3$): δ = 1.18 (t, ^3J = 7.1 Hz, 3H), 1.51 (s, 6H), 4.14 (q, ^3J = 7.1 Hz, 2H), 6.30 (dd, ^3J = 8.1 Hz, ^4J = 1.6 Hz, 1H), 6.74 (d, ^3J = 7.7 Hz, 1H), 6.95 - 7.11 (m, 2H), 7.50 (dd, ^3J = 7.0, 7.0 Hz, 2H), 7.60 (dd, ^3J = 7.1, 7.1 Hz, 1H), 7.92 - 8.03 (m, 1H), 8.07 (s, 1H) ppm. ^{13}C-NMR (75 MHz, $CDCl_3$): δ = 14.2, 26.2, 57.6, 61.5, 107.7, 110.3, 111.5, 124.6, 128.7, 129.5, 130.6, 133.0, 133.9, 138.4, 146.2, 146.3, 164.5, 176.1 ppm.

Ethyl (3-[4-methoxy-2-nitrobenzamido]phenyl)glycinate (46s): Preparation according to general procedure B using **44f** and **43e**. Yield: 78%. Yellow solid. ^{1}H-NMR (300 MHz, DMSO-d_6): δ = 1.20 (t, ^3J = 7.1 Hz, 3H), 3.85 (d, ^3J = 6.4 Hz, 2H), 3.91 (s, 3H), 4.12 (q, ^3J = 7.1 Hz, 2H), 6.11 (t, ^3J = 6.4 Hz, 1H), 6.30 (dd, ^3J = 8.0 Hz, ^4J = 1.4 Hz, 1H), 6.87 (d, ^3J = 8.0 Hz, 1H), 6.98 (s, 1H), 7.03 (dd, ^3J = 8.0, 8.0 Hz, 1H), 7.37 (dd, ^3J = 8.5 Hz, ^4J = 2.5 Hz, 1H), 7.60 (d, ^4J = 2.5 Hz, 1H), 7.68 (d, ^3J = 8.5 Hz, 1H), 10.32 (s, 1H) ppm. ^{13}C-NMR (75 MHz, DMSO-d_6): δ = 14.1, 44.7, 56.3, 60.3, 103.7, 107.8, 108.2, 109.4, 118.7, 124.9, 129.0, 130.6, 139.7, 148.4, 148.5, 160.3, 163.5, 171.2 ppm.

Ethyl 3-(3-[2-amino-3-methylbenzamido]phenyl)propanoate (47a): Preparation according to general procedure D using **46a**. Yield: 83%. Yellow oil. ^{1}H-NMR (300 MHz, $CDCl_3$): δ = 1.24 (t, ^3J = 7.1 Hz, 3H), 2.20 (s, 3H), 2.64 (t, ^3J = 7.7 Hz, 2H), 2.96 (t, ^3J = 7.8 Hz, 2H), 4.13 (q, ^3J = 7.1 Hz, 2H), 5.57 (br s, 2H), 6.66 (dd, ^3J = 7.6, 7.6 Hz, 1H), 6.99 (d, ^3J = 7.6 Hz, 1H), 7.18 (d, ^3J = 7.3, Hz, 1H), 7.28 (dd,

^3J = 7.8, 7.8 Hz, 1H), 7.36 (dd, ^3J = 7.9 Hz, ^4J = 1.3 Hz, 1H), 7.39 - 7.44 (m, 1H), 7.45 (dd, ^4J = 1.7, 1.7 Hz, 1H), 7.75 (s, 1H) ppm. ^{13}C-NMR (75 MHz, $CDCl_3$): δ = 14.4, 17.7, 31.1, 36.0, 60.6, 116.0, 116.5, 118.6, 120.5, 124.3, 124.6, 125.1, 129.3, 133.8, 138.2, 141.8, 147.2, 168.2, 173.0 ppm.

Ethyl 3-(3-[2-amino-4-methylbenzamido]phenyl)propanoate (47b): Preparation according to general procedure D using **46b**. Yield: 97%. Brown solid. ^{1}H-NMR (300 MHz, $CDCl_3$): δ = 1.24 (t, ^3J = 7.1 Hz, 3H), 2.28 (s, 3H), 2.63 (t, ^3J = 7.7 Hz, 2H), 2.95 (t, ^3J = 7.8 Hz, 2H), 4.13 (q, ^3J = 7.1 Hz, 2H), 4.98 (br s, 2H), 6.50 - 6.57 (m, 2H), 6.98 (ddd, ^3J = 7.5 Hz, ^4J = 1.6, 1.1 Hz, 1H), 7.27 (dd, ^3J = 7.8, 7.8 Hz, 1H), 7.35 (d, ^3J = 8.6 Hz, 1H), 7.38 - 7.43 (m, 1H), 7.44 (dd, ^4J = 1.7, 1.7 Hz, 1H), 7.73 (s, 1H) ppm. ^{13}C-NMR (75 MHz, $CDCl_3$): δ = 14.4, 21.6, 31.1, 36.0, 60.6, 113.8, 118.1, 118.4, 118.5, 120.5, 124.4, 127.2, 129.3, 138.3, 141.8, 143.6, 149.1, 167.6, 173.0 ppm.

Ethyl 3-(3-[2-amino-5-methylbenzamido]phenyl)propanoate (47c): Preparation according to general procedure D using **46c**. Yield: 95%. Brown solid. ^{1}H-NMR (300 MHz, $CDCl_3$): δ = 1.24 (t, ^3J = 7.1 Hz, 3H), 2.28 (s, 3H), 2.64 (t, ^3J = 7.8 Hz, 2H), 2.96 (t, ^3J = 7.8 Hz, 2H), 4.13 (q, ^3J = 7.1 Hz, 2H), 5.14 (br s, 2H), 6.66 (d, ^3J = 8.3 Hz, 1H), 6.98 (d, ^3J = 7.8 Hz, 1H), 7.08 (dd, ^3J = 8.0 Hz, ^4J = 1.7 Hz, 1H), 7.24 - 7.31 (m, 2H), 7.41 - 7.47 (m, 2H), 7.81 (s, 1H) ppm. ^{13}C-NMR (75 MHz, $CDCl_3$): δ = 14.4, 20.5, 31.1, 36.0, 60.6, 116.8, 118.1, 118.5, 120.5, 124.5, 126.5, 127.4, 129.3, 133.8, 138.2, 141.8, 146.4, 167.6, 173.0 ppm.

Ethyl 3-(3-[2-amino-6-methylbenzamido]phenyl)propanoate (47d): Preparation according to general procedure D using **46d**. Yield: 76%. Brown oil. ^{1}H-NMR (300 MHz, $CDCl_3$): δ = 1.23 (t, ^3J = 7.1 Hz, 3H), 2.36 (s, 3H), 2.59 (t, ^3J = 7.8 Hz, 2H), 2.91 (t, ^3J = 7.8 Hz, 2H), 3.96 (br s, 2H), 4.10 (q, ^3J = 7.1 Hz, 2H), 6.52 (d, ^3J = 8.0 Hz, 1H), 6.58 (d, ^3J = 7.5 Hz, 1H), 6.97 (d, ^3J = 7.5, Hz, 1H), 7.04 (dd, ^3J = 7.8, 7.8 Hz, 1H), 7.24 (dd, ^3J = 7.8, 7.8 Hz, 1H), 7.39 - 7.48 (m, 2H), 7.88 (s, 1H) ppm. ^{13}C-NMR (75 MHz, $CDCl_3$): δ = 14.3, 20.1, 31.0, 35.8, 60.5, 114.0, 118.0, 119.9, 120.5, 123.1, 124.6, 129.2, 130.3, 135.5, 138.1, 141.7, 144.7, 167.7, 173.0 ppm.

Ethyl 3-(3-[2-amino-4-chlorobenzamido]phenyl)propanoate (47e): Preparation according to general procedure E using **46e**. Yield: 92%. Brown solid. ^{1}H-NMR (300 MHz, $CDCl_3$): δ = 1.23 (t, ^3J = 7.1 Hz, 3H), 2.61 (t, ^3J = 7.7 Hz, 2H), 2.93 (t, ^3J = 7.8 Hz, 2H), 4.12 (q, ^3J = 7.1 Hz, 2H), 5.34 (br s, 2H), 6.64 (dd, ^3J = 8.4 Hz, ^4J = 2.0 Hz, 1H), 6.70 (d, ^4J = 2.0 Hz, 1H), 6.98 (d, ^3J = 7.4 Hz, 1H), 7.25 (dd, ^3J = 7.9, 7.9 Hz, 1H), 7.33 - 7.45 (m, 3H), 7.82 (s, 1H) ppm. ^{13}C-NMR (75 MHz, $CDCl_3$): δ = 14.3, 31.0, 35.9, 60.6, 114.8, 117.0, 117.1, 118.7, 120.7, 124.7, 128.6, 129.3, 137.9, 138.6, 141.8, 149.9, 167.0, 173.0 ppm.

Ethyl 3-(3-[2-amino-4-methoxybenzamido]phenyl)propanoate (47f): Preparation according to general procedure D using **46f**. Yield: 98%. Pale yellow solid. ^{1}H-NMR (300 MHz, $CDCl_3$): δ = 1.24 (t, ^3J = 7.1 Hz, 3H), 2.63 (t, ^3J = 7.8 Hz, 2H), 2.95 (t, ^3J = 7.8 Hz, 2H), 3.80 (s, 3H), 4.13 (q, ^3J = 7.1 Hz, 2H), 5.80 (br s, 2H), 6.19 (d, ^4J = 2.4 Hz, 1H), 6.28 (dd, ^3J = 8.8 Hz, ^4J = 2.5 Hz, 1H), 6.97 (d, ^3J = 7.5 Hz, 1H), 7.26 (dd, ^3J = 7.8, 7.8 Hz, 1H), 7.35 - 7.45 (m, 3H), 7.65 (s, 1H) ppm. ^{13}C-NMR (75 MHz, $CDCl_3$): δ = 14.4, 31.1, 36.0, 55.4, 60.6, 101.1, 104.6, 109.4, 118.6, 120.5, 124.3, 129.0, 129.2, 138.3, 141.8, 151.3, 163.4, 167.4, 173.0 ppm.

Ethyl 3-(3-[2-amino-4-{trifluoromethoxy}benzamido]phenyl)propanoate (47g): Preparation according to general procedure D using **46g**. Yield: 90%. Yellow solid. ^{1}H-NMR (400 MHz, $CDCl_3$): δ = 1.17 (t, ^3J = 7.1 Hz, 3H), 2.56 (t, ^3J = 7.7 Hz, 2H), 2.88 (t, ^3J = 7.7 Hz, 2H), 4.06 (q, ^3J = 7.1 Hz, 2H), 4.84 (s, 2H), 6.46 - 6.54 (m, 2H), 6.93 (d, ^3J = 7.5 Hz, 1H), 7.16 - 7.24 (m, 1H), 7.32 (d, ^3J = 8.1 Hz, 1H), 7.36 (s, 1H), 7.42 (d, ^3J = 9.1 Hz, 1H), 7.69 (br s, 1H) ppm. ^{13}C-NMR (75 MHz, $CDCl_3$): δ = 14.3, 31.0, 35.9, 60.6, 108.8, 109.0, 116.9, 118.7, 120.7, 124.9, 129.2, 129.3, 137.8, 141.9, 150.2, 152.5, 166.7, 173.0 ppm.

Ethyl 3-(3-[2-amino-4-{tert-butyl}benzamido]phenyl)propanoate (47h): Preparation according to general procedure D using **46h**. Yield: 85%. Brown solid. ^{1}H-NMR (500 MHz, $CDCl_3$): δ = 1.24 (t, ^3J = 7.2 Hz, 3H), 1.30 (s, 9H), 2.63 (t, ^3J = 7.8 Hz, 2H), 2.95 (t, ^3J = 7.8 Hz, 2H), 4.13 (q, ^3J = 7.1 Hz,

2H), 5.40 (br s, 2H), 6.75 (d, ^4J = 1.6 Hz, 1H), 6.77 (dd, ^3J = 8.3 Hz, ^4J = 1.8 Hz, 1H), 6.98 (d, ^3J = 7.6 Hz, 1H), 7.27 (dd, ^3J = 7.8, 7.8 Hz, 1H), 7.38 - 7.43 (m, 2H), 7.45 (s, 1H), 7.78 (s, 1H) ppm. ^{13}C-NMR (126 MHz, $CDCl_3$): δ = 14.3, 31.1, 31.1, 34.9, 36.0, 60.6, 113.9, 114.9, 115.1, 118.5, 120.5, 124.5, 127.1, 129.3, 138.2, 141.8, 148.5, 156.7, 167.5, 173.0 ppm.

Ethyl 3-(3-[2-amino-5-chlorobenzamido]phenyl)propanoate (47i): Preparation according to general procedure E using **46i**. Yield: 61%. White solid. ^{1}H-NMR (300 MHz, $CDCl_3$): δ = 1.23 (t, ^3J = 7.1 Hz, 3H), 2.60 (t, ^3J = 7.7 Hz, 2H), 2.91 (t, ^3J = 77.8 Hz, 2H), 4.11 (q, ^3J = 7.1 Hz, 2H), 5.36 (br s, 2H), 6.62 (d, ^3J = 8.7 Hz, 1H), 6.96 (d, ^3J = 7.4 Hz, 1H), 7.15 (dd, ^3J = 8.7 Hz, ^4J = 2.4 Hz, 1H), 7.20 - 7.27 (m, 1H), 7.37 - 7.41 (m, 2H), 7.42 (d, ^4J = 2.4 Hz, 1H), 7.99 (s, 1H) ppm. ^{13}C-NMR (75 MHz, $CDCl_3$): δ = 14.3, 31.0, 35.8, 60.6, 117.4, 118.8, 118.9, 120.7, 121.3, 124.7, 127.1, 129.2, 132.5, 137.9, 141.7, 147.3, 166.6, 173.1 ppm.

Ethyl 3-(3-[2-amino-5-methoxybenzamido]phenyl)propanoate (47j): Preparation according to general procedure D using **46j**. Yield: 47%. Yellow oil. ^{1}H-NMR (300 MHz, $CDCl_3$): δ = 1.23 (t, ^3J = 7.1 Hz, 3H), 2.61 (t, ^3J = 7.7 Hz, 2H), 2.93 (t, ^3J = 7.8 Hz, 2H), 3.76 (s, 3H), 4.12 (q, ^3J = 7.1 Hz, 2H), 4.58 (br s, 2H), 6.69 (d, ^3J = 8.8 Hz, 1H), 6.89 (dd, ^3J = 8.8 Hz, ^4J = 2.9 Hz, 1H), 6.96 (d, ^3J = 7.6 Hz, 1H), 7.05 (d, ^4J = 2.9 Hz, 1H), 7.25 (dd, ^3J = 7.7, 7.7 Hz, 1H), 7.39 - 7.45 (m, 2H), 8.19 (s, 1H) ppm. ^{13}C-NMR (75 MHz, $CDCl_3$): δ = 14.3, 31.0, 35.9, 56.1, 60.6, 112.4, 118.4, 118.5, 119.5, 119.8, 120.5, 124.5, 129.2, 138.2, 141.7, 141.9, 151.8, 167.2, 173.0 ppm.

Ethyl 3-(3-[2-amino-5-{trifluoromethoxy}benzamido]phenyl)propanoate (47k): Preparation according to general procedure D using **46k**. Yield: 97%. Brown solid. ^{1}H-NMR (500 MHz, $CDCl_3$): δ = 1.24 (t, ^3J = 7.1 Hz, 3H), 2.63 (t, ^3J = 7.8 Hz, 2H), 2.94 (t, ^3J = 7.8 Hz, 2H), 4.13 (q, ^3J = 7.1 Hz, 2H), 5.58 (br s, 2H), 6.74 (d, ^3J = 8.9 Hz, 1H), 7.00 (d, ^3J = 7.6 Hz, 1H), 7.14 (dd, ^3J = 8.9 Hz, ^4J = 1.7 Hz, 1H), 7.27 (dd, ^3J = 7.8, 7.8 Hz, 1H), 7.33 (d, ^4J = 2.4 Hz, 1H), 7.39 - 7.44 (m, 2H), 7.82 (s, 1H) ppm. ^{13}C-NMR (126 MHz, $CDCl_3$): δ = 14.3, 31.0, 35.9, 60.7, 116.9, 118.77, 118.83, 120.5, 120.75, 120.77, 125.0, 126.3, 129.3, 137.7, 139.8, 141.9, 147.0, 166.4, 173.0 ppm.

Ethyl 3-(3-[6-aminobenzo[1,3]dioxole-5-carboxamido]phenyl)propanoate (47l): Preparation according to general procedure D using **46l**. Yield: 75%. Brown oil. ^{1}H-NMR (300 MHz, $CDCl_3$): δ = 1.24 (t, ^3J = 7.1 Hz, 3H), 2.63 (t, ^3J = 7.8 Hz, 2H), 2.95 (t, ^3J = 7.8 Hz, 2H), 4.13 (q, ^3J = 7.1 Hz, 2H), 5.53 (br s, 2H), 5.91 (s, 2H), 6.23 (s, 1H), 6.91 (s, 1H), 6.96 (d, ^3J = 7.7 Hz, 1H), 7.26 (dd, ^3J = 7.8, 7.8 Hz, 1H), 7.34 - 7.39 (m, 1H), 7.40 - 7.43 (m, 1H), 7.62 (s, 1H) ppm. ^{13}C-NMR (75 MHz, $CDCl_3$): δ = 14.4, 31.1, 36.0, 60.6, 98.5, 101.4, 105.7, 107.6, 118.6, 120.5, 124.4, 129.2, 138.3, 139.8, 141.8, 146.9, 151.7, 167.3, 173.0 ppm.

Ethyl 2-(2-aminobenzamido)phenylacetate (47m): Preparation according to general procedure D using **46m**. Yield: 86%. Beige solid. ^{1}H-NMR (400 MHz, $CDCl_3$): δ = 1.28 (t, ^3J = 7.1 Hz, 3H), 3.67 (s, 2H), 4.19 (q, ^3J = 7.1 Hz, 2H), 5.74 (br s, 2H), 6.66 - 6.78 (m, 2H), 7.15 (ddd, ^3J = 7.5, 7.5 Hz, ^4J = 1.1 Hz, 1H), 7.22 - 7.30 (m, 2H), 7.36 (ddd, ^3J = 7.7, 7.7 Hz, ^4J = 1.4 Hz, 1H), 7.67 (dd, ^3J = 7.9 Hz, ^4J = 1.2 Hz, 1H), 7.90 (d, ^3J = 8.0 Hz, 1H), 9.45 (br s, 1H) ppm. ^{13}C-NMR (101 MHz, $CDCl_3$): δ = 14.2, 39.2, 61.9, 115.4, 116.8, 117.6, 125.4, 125.5, 126.6, 127.7, 128.4, 131.0, 132.9, 137.0, 149.9, 168.0, 173.0 ppm.

Ethyl 3-(3-[2-aminobenzamido]phenyl)propanoate (47n): Preparation according to general procedure D using **46n**. Yield: 82%. Beige solid. ^{1}H-NMR (400 MHz, $CDCl_3$): δ = 1.24 (t, ^3J = 7.1 Hz, 3H), 2.63 (t, ^3J = 7.8 Hz, 2H), 2.95 (t, ^3J = 7.8 Hz, 2H), 4.13 (q, ^3J = 7.1 Hz, 2H), 5.51 (br s, 2H), 6.64 - 6.77 (m, 2H), 6.98 (d, ^3J = 7.6 Hz, 1H), 7.20 - 7.31 (m, 2H), 7.41 (d, ^3J = 8.1 Hz, 1H), 7.43 - 7.49 (m, 2H), 7.79 (br s, 1H) ppm. ^{13}C-NMR (75 MHz, $CDCl_3$): δ = 14.3, 31.0, 35.9, 60.6, 116.3, 116.9, 117.6, 118.6, 120.5, 124.6, 127.3, 129.3, 132.9, 138.1, 141.8, 149.1, 167.7, 173.0 ppm.

Ethyl 3-(4-[2-aminobenzamido]phenyl)propanoate (47o): Preparation according to general procedure F using **46o**. Yield: 33%. Brown oil. ^{1}H-NMR (250 MHz, $CDCl_3$): δ = 1.12 (t, ^3J = 7.1 Hz, 3H), 2.47

(t, ^3J = 7.7 Hz, 2H), 2.78 (t, ^3J = 7.7 Hz, 2H), 4.00 (q, ^3J = 7.1 Hz, 2H), 5.20 (br s, 2H), 6.43 - 6.65 (m, 2H), 6.87-7.20 (m, 3H), 7.34 (d, ^3J = 8.3 Hz, 3H), 7.97 (s, 1H) ppm. ^{13}C-NMR (75 MHz, $CDCl_3$): δ = 14.2, 30.3, 35.9, 60.5, 111.9, 116.7, 117.4, 121.0, 127.5, 128.7, 132.6, 136.1, 136.7, 148.8, 167.8, 173.1 ppm.

2-Aminobenzamidoanthranilic acid (47p): Preparation according to general procedure G using **51** and **42c**. Yield: 74%. Beige solid. ^{1}H-NMR (400 MHz, DMSO-d_6): δ = 6.62 (ddd, ^3J = 7.5, 7.5 Hz, ^4J = 1.0 Hz, 1H), 6.80 (dd, ^3J = 8.2 Hz, ^4J = 0.8 Hz, 1H), 7.17 (ddd, ^3J = 7.6, 7.6 Hz, ^4J = 1.0 Hz, 1H), 7.24 (ddd, ^3J = 7.7, 7.7 Hz, ^4J = 1.4 Hz, 1H), 7.57-7.66 (m, 2H), 8.04 (d, ^3J = 7.9 Hz, ^4J = 1.6 Hz, 1H), 8.64 (dd, ^3J = 8.4, 8.4 Hz, 1H), 11.91 (s, 1H) ppm. ^{13}C-NMR (101 MHz, DMSO-d_6): δ = 114.1, 115.2, 116.3, 117.1, 119.9, 122.5, 127.3, 131.3, 132.8, 134.2, 141.4, 150.5, 167.5, 170.0 ppm.

3-(3-[2-Aminobenzamido]phenyl)propanoic acid (47q)*:* Preparation according to general procedure G using **51** and **42a**. Yield: 98%. Beige solid. ^{1}H-NMR (400 MHz, DMSO-d_6): δ = 2.54 (t, ^3J = 7.7 Hz, 2H), 2.81 (t, ^3J = 7.6 Hz, 2H), 6.30 (br s, 2H), 6.58 (ddd, ^3J = 7.5, 7.5 Hz, ^4J = 1.1 Hz, 1H), 6.74 (dd, ^3J = 8.3 Hz, ^4J = 0.9 Hz, 1H), 6.94 (d, ^3J = 7.7, Hz, 1H), 7.12 - 7.32 (m, 2H), 7.48 - 7.65 (m, 3H), 9.92 (s, 1H), 12.10 (br s, 1H) ppm. ^{13}C-NMR (101 MHz, DMSO-d_6): δ = 30.5, 35.2, 114.7, 115.3, 116.4, 118.4, 120.4, 123.3, 128.4, 128.7, 132.1, 139.2, 141.2, 149.7, 167.8, 173.7 ppm.

Ethyl 3-(2-aminobenzamido)phenoxyacetate (47r): Preparation according to general procedure D using **46p**. Yield: 78%. Yellow oil. ^{1}H-NMR (500 MHz, $CDCl_3$): δ = 1.30 (t, ^3J = 7.1 Hz, 3H), 4.28 (q, ^3J = 7.1 Hz, 2H), 4.63 (s, 2H), 6.69 (dd, ^3J = 8.2 Hz, ^4J = 2.5 Hz, 1H), 6.72 - 6.79 (m, 2H), 7.11 (dd, ^3J = 8.0 Hz, ^4J = 1.2 Hz, 1H), 7.21 - 7.29 (m, 2H), 7.36 (dd, ^4J = 2.2, 2.2 Hz, 1H), 7.47 (d, ^3J = 7.8 Hz, ^4J = 1.1 Hz, 1H), 7.90 (s, 1H) ppm. ^{13}C-NMR (126 MHz, $CDCl_3$): δ = 14.3, 61.6, 65.6, 107.2, 110.9, 113.8, 117.0, 117.7, 118.2, 127.4, 130.0, 133.0, 139.3, 148.0, 158.5, 167.5, 169.1 ppm.

Ethyl (3-[2-aminobenzamido]phenyl)glycinate (47s): Preparation according to general procedure D using **46q**. Yield: 80%. Yellow oil. ^{1}H-NMR (300 MHz, $CDCl_3$): δ = 1.26 (t, ^3J = 7.1 Hz, 3H), 3.83 (s, 2H), 4.19 (q, ^3J = 7.1 Hz, 2H), 4.99 (s, 3H), 6.32 (ddd, ^3J = 8.1 Hz, ^4J = 2.2, 0.7 Hz, 1H), 6.57 - 6.69 (m, 2H), 6.82 (dd, ^3J = 8.0 Hz, ^4J = 1.2 Hz, 1H), 6.95 (dd, ^4J = 2.1, 2.1 Hz, 1H), 7.09 (dd, ^3J = 8.0, 8.0 Hz, 1H), 7.18 (dd, ^3J = 8.4, 7.2 Hz, ^4J = 1.5 Hz, 1H), 7.41 (dd, ^3J = 7.9 Hz, ^4J = 1.4 Hz, 1H), 7.99 (s, 1H) ppm. ^{13}C-NMR (75 MHz, $CDCl_3$): δ = 14.1, 45.7, 61.3, 105.4, 108.9, 110.3, 116.6, 116.7, 117.3, 127.4, 129.6, 132.4, 139.0, 147.7, 148.7, 167.6, 171.2 ppm.

Ethyl 2-([3-{2-aminobenzamido}phenyl]amino)-2-methylpropanoate (47t): Preparation according to general procedure D using **46r**. Yield: 64%. Brown solid. ^{1}H-NMR (300 MHz, $CDCl_3$): δ = 1.20 (t, ^3J = 7.1 Hz, 3H), 1.56 (s, 6H), 4.18 (q, ^3J = 7.1 Hz, 2H), 5.01 (br s, 3H), 6.36 (ddd, ^3J = 8.1 Hz, ^4J = 2.3, 0.8 Hz, 1H), 6.64 - 6.73 (m, 2H), 6.82 (ddd, ^3J = 8.0 Hz, ^4J = 1.9, 0.7 Hz, 1H), 7.04 - 7.14 (m, 2H), 7.18 - 7.26 (m, 1H), 7.43 (dd, ^3J = 8.3 Hz, ^4J = 1.4 Hz, 1H), 7.74 (s, 1H) ppm. ^{13}C-NMR (75 MHz, $CDCl_3$): δ = 14.2, 26.2, 57.8, 61.5, 107.9, 110.7, 111.4, 116.8, 116.9, 117.6, 127.3, 129.5, 132.7, 138.8, 146.1, 148.9, 167.6, 176.0 ppm.

Ethyl (3-[2-amino-4-methoxybenzamido]phenyl)glycinate (47u): Preparation according to general procedure D using **46s**. Yield: 83%. Beige solid. ^{1}H-NMR (300 MHz, DMSO-d_6): δ = 1.20 (t, ^3J = 7.1 Hz, 3H), 3.73 (s, 3H), 3.85 (d, ^3J = 6.4 Hz, 2H), 4.12 (q, ^3J = 7.1 Hz, 2H), 5.98 (t, ^3J = 6.4, Hz, 1H), 6.17 (dd, ^3J = 8.8 Hz, ^4J = 2.4 Hz, 1H), 6.22 - 6.33 (m, 2H), 6.53 (s, 2H), 6.91 (d, ^3J = 8.3 Hz, 1H), 6.95 - 7.09 (m, 2H), 7.59 (d, ^3J = 8.9 Hz, 1H), 9.54 (s, 1H) ppm. ^{13}C-NMR (75 MHz, DMSO-d_6): δ = 14.1, 44.8, 54.9, 60.2, 99.4, 102.3, 104.5, 107.3, 108.4, 109.2, 128.7, 130.3, 140.1, 148.3, 152.1, 162.3, 167.4, 171.3 ppm.

2H-Chromene-3-carboxylic acid (48b): Preparation according to general procedures O and P in a sequential one pot manner using **71** and **72a**. Yield: 52%. Yellow solid. ^{1}H-NMR (400 MHz, DMSO-d_6): δ = 4.90 (d, ^4J = 1.4 Hz, 2H), 6.84 (d, ^3J = 8.1 Hz, 1H), 6.95 (ddd, ^3J = 7.5, 7.5 Hz, ^4J = 1.0 Hz, 1H),

7.26 (ddd, ^{3}J = 7.9, 7.9 Hz, ^{4}J = 1.6 Hz, 1H), 7.32 (dd, ^{3}J = 7.5 Hz, ^{4}J = 1.5 Hz, 1H), 7.44 (s, 1H), 12.83 (s, 1H) ppm. ^{13}C-NMR (101 MHz, DMSO-d_6): δ = 64.1, 115.7, 120.8, 121.7, 123.4, 129.1, 131.8, 132.3, 154.4, 165.5 ppm.

4-Chloro-2H-chromene-3-carboxylic acid (48c): Preparation according to general procedure T using **77**. Yield: 45%. Yellow solid. ^{1}H-NMR (500 MHz, DMSO-d_6): δ = 4.97 (s, 2H), 6.96 (dd, ^{3}J = 8.1 Hz, ^{4}J = 0.9 Hz, 1H), 7.10 (ddd, ^{3}J = 7.7, 7.7 Hz, ^{4}J = 1.1 Hz, 1H), 7.38 (ddd, ^{3}J = 8.0, 8.0 Hz, ^{4}J = 1.6 Hz, 1H), 7.63 (dd, ^{3}J = 7.8 Hz, ^{4}J = 1.5 Hz, 1H), 13.39 (br s, 1H) ppm. ^{13}C-NMR (126 MHz, DMSO-d_6): δ = 66.2, 116.1, 120.9, 121.2, 122.3, 126.4, 132.5, 132.6, 155.0, 164.1 ppm.

5-Methyl-2H-chromene-3-carboxylic acid (48d): Preparation according to general procedure P using **73b**. Yield: 84%. Yellow solid. ^{1}H-NMR (400 MHz, DMSO-d_6): δ = 2.33 (s, 3H), 4.84 (d, ^{4}J = 0.7 Hz, 2H), 6.70 (d, ^{3}J = 8.1 Hz, 1H), 6.82 (d, ^{3}J = 7.6 Hz, 1H), 7.15 (dd, ^{3}J = 7.8, 7.8 Hz, 1H), 7.53 (s, 1H), 12.81 (br s, 1H) ppm. ^{13}C-NMR (75 MHz, DMSO-d_6): 18.0, 63.5, 113.7, 119.6, 123.2, 123.3, 129.3, 131.2, 136.7, 154.8, 165.6 ppm.

6-Methyl-2H-chromene-3-carboxylic acid (48e): Preparation according to general procedure P using **73c**. Yield: 40%. Yellow solid. ^{1}H-NMR (400 MHz, DMSO-d_6): δ = 2.21 (s, 3H), 4.85 (d, ^{4}J = 1.4 Hz, 2H), 6.74 (d, ^{3}J = 8.2 Hz, 1H), 7.07 (dd, ^{3}J = 8.2 Hz, ^{4}J = 1.6 Hz, 1H), 7.12 (d, ^{4}J = 1.7 Hz, 1H), 7.39 (s, 1H), 12.73 (br s, 1H) ppm. ^{13}C-NMR (75 MHz, DMSO-d_6): 20.0, 64.1, 115.4, 120.7, 123.5, 129.2, 130.6, 132.2, 132.4, 152.3, 165.5 ppm.

6-Fluoro-2H-chromene-3-carboxylic acid (48f): Preparation according to general procedure P using **73d**. Yield: 85%. Pale yellow solid. ^{1}H-NMR (400 MHz, DMSO-d_6): δ = 4.89 (d, ^{4}J = 1.4 Hz, 2H), 6.86 (dd, ^{3}J = 8.9 Hz, ^{4}J = 4.6 Hz, 1H), 7.08 (ddd, ^{3}J = 8.7, 8.7 Hz, ^{4}J = 3.1 Hz, 1H), 7.23 (dd, ^{3}J = 8.7 Hz, ^{4}J = 3.1 Hz, 1H), 7.42 (s, 1H), 12.92 (br s, 1H) ppm. ^{13}C-NMR (75 MHz, DMSO-d_6): δ = 64.3, 114.9, 116.9, 117.9, 122.1, 125.0, 131.4, 150.6, 156.9, 165.3 ppm.

6-Chloro-2H-chromene-3-carboxylic acid (48g): Preparation according to general procedure P using **73e**. Yield: 70%. Pale yellow solid. ^{1}H-NMR (400 MHz, DMSO-d_6): δ = 4.92 (d, ^{4}J = 1.4 Hz, 2H), 6.86 (d, ^{3}J = 8.7 Hz, 1H), 7.27 (dd, ^{3}J = 8.6 Hz, ^{4}J = 2.6 Hz, 1H), 7.38 - 7.47 (m, 2H), 12.97 (s, 1H) ppm. ^{13}C-NMR (75 MHz, DMSO-d_6): δ = 64.5, 117.5, 122.6, 124.8, 125.3, 128.3, 131.1, 153.2, 165.3 ppm.

7-Methyl-2H-chromene-3-carboxylic acid (48h): Preparation according to general procedure P using **73f**. Yield: 93%. White solid. ^{1}H-NMR (400 MHz, DMSO-d_6): δ = 2.25 (s, 3H), 4.87 (d, ^{4}J = 1.2 Hz, 2H), 6.68 (s, 1H), 6.77 (d, ^{3}J = 7.7 Hz, 1H), 7.19 (d, ^{3}J = 7.7 Hz, 1H), 7.41 (s, 1H), 12.74 (br s, 1H) ppm. ^{13}C-NMR (126 MHz, DMSO-d_6): δ = 21.2, 64.2, 116.2, 118.3, 122.2, 122.6, 129.0, 132.5, 142.3, 154.5, 165.6 ppm.

8-Methyl-2H-chromene-3-carboxylic acid (48i): Preparation according to general procedure P using **73g**. Yield: 79%. Yellow solid. ^{1}H-NMR (400 MHz, DMSO-d_6): δ = 2.12 (s, 3H), 4.92 (d, ^{4}J = 1.4 Hz, 2H), 6.85 (dd, ^{3}J = 7.5, 7.5 Hz, 1H), 7.14 (d, ^{3}J = 7.6 Hz, 2H), 7.42 (dd, ^{4}J = 1.3, 1.3 Hz, 1H), 12.82 (br s, 1H) ppm. ^{13}C-NMR (75 MHz, DMSO-d_6): 15.2, 64.2, 120.4, 121.2, 123.1, 124.6, 126.8, 132.7, 133.2, 152.5, 165.6 ppm.

8-Chloro-2H-chromene-3-carboxylic acid (48j): Preparation according to general procedure P using **73h**. Yield: 88%. Pale yellow solid. ^{1}H-NMR (400 MHz, DMSO-d_6): δ = 5.03 (d, ^{4}J = 1.4 Hz, 2H), 6.96 (dd, ^{3}J = 7.8, 7.8 Hz, 1H), 7.30 (dd, ^{3}J = 7.6 Hz, ^{4}J = 1.4 Hz, 1H), 7.38 (dd, ^{3}J = 8.0 Hz, ^{4}J = 1.5 Hz, 1H), 7.45 (t, ^{4}J = 1.3 Hz, 1H), 12.95 (br s, 1H) ppm. ^{13}C-NMR (75 MHz, DMSO-d_6): 65.0, 119.8, 122.3, 122.4, 124.4, 127.9, 131.4, 131.8, 149.9, 165.2 ppm.

8-Methoxy-2H-chromene-3-carboxylic acid (48k): Preparation according to general procedure P using **73i**. Yield: 92%. Yellow solid. ^{1}H-NMR (400 MHz, DMSO-d_6): δ = 3.76 (s, 3H), 4.87 (d, ^{4}J = 1.4 Hz, 2H), 6.85 - 6.96 (m, 2H), 7.01 (dd, ^{3}J = 7.2 Hz, ^{4}J = 2.4 Hz, 1H), 7.42 (dd, ^{4}J = 1.3, 1.3 Hz, 1H), 12.84

(br s, 1H) ppm. ^{13}C-NMR (126 MHz, DMSO-d_6): δ = 55.7, 64.0, 115.1, 120.8, 121.5, 121.6, 123.5, 132.5, 143.4, 147.6, 165.5 ppm.

4-(2-Furyl)benzoic acid (48m): Preparation according to general procedure U using **62** and **63a**. Yield: 81%. Brown solid. ^{1}H-NMR (300 MHz, CDCl$_3$): δ = 6.65 (dd, ^3J = 3.4, 1.8 Hz, 1H), 7.13 (dd, ^3J = 3.4 Hz, ^4J = 0.6 Hz, 1H), 7.78 - 7.86 (m, 3H), 7.90 (ddd, ^3J = 8.5 Hz, ^4J = 1.8, 1.8 Hz, 2H), 12.92 (br s, 1H) ppm. ^{13}C-NMR (75 MHz, CDCl$_3$): δ = 108.2, 112.4, 123.2, 129.2, 130.0, 134.0, 144.1, 152.1, 166.9 ppm.

4-(3-Furyl)benzoic acid (48n): Preparation according to general procedure U using **62** and **63b**. Yield: 86%. White solid. ^{1}H-NMR (500 MHz, DMSO-d_6): δ = 7.04 (d, ^4J = 0.8 Hz, 1H), 7.74 (d, ^4J = 8.3 Hz, 2H), 7.79 (s, 1H), 7.94 (d, ^3J = 8.3 Hz, 2H), 8.32 (s, 1H), 12.88 (br s, 1H) ppm. ^{13}C-NMR (126 MHz, DMSO-d_6): δ = 108.7, 125.1, 125.5, 129.0, 129.9, 136.4, 140.7, 144.7, 167.1 ppm.

4-(3-Thienyl)benzoic acid (48p): Preparation according to general procedure U using **62** and **63c**. Yield: 90%. Beige solid. ^{1}H-NMR (300 MHz, DMSO-d_6): δ = 7.64 (dd, ^3J = 5.1 Hz, ^4J = 1.4 Hz, 1H), 7.69 (dd, ^3J = 5.0 Hz, ^4J = 2.9 Hz, 1H), 7.85 (ddd, ^3J = 8.7 Hz, ^4J = 1.8, 1.8 Hz, 2H), 7.97 (ddd, ^3J = 8.6 Hz, ^4J = 1.8, 1.8 Hz, 2H), 8.05 (dd, ^4J = 2.9, 1.4 Hz, 1H), 12.91 (br s, 1H) ppm. ^{13}C-NMR (75 MHz, DMSO-d_6): δ = 122.8, 126.0, 126.2, 127.5, 129.1, 130.0, 139.1, 140.3, 167.1 ppm.

4-(Isoxazol-3-yl)benzoic acid (48q): Preparation according to general procedure X using **67** and **65**. Yield: 20%. White solid. ^{1}H-NMR (300 MHz, DMSO-d_6): δ = 7.27 (d, ^4J = 1.8 Hz, 1H), 8.02 - 8.07 (m, 2H), 8.07 - 8.16 (m, 2H), 9.09 (d, ^4J = 1.7 Hz, 1H), 13.16 (br s, 1H) ppm. ^{13}C-NMR (75 MHz, DMSO-d_6): δ = 103.2, 126.9, 130.0, 132.1, 132.3, 160.3, 161.2, 166.8 ppm.

4-(4-Isoxazolyl)benzoic acid (48r): Preparation according to general procedure U using **62** and **63d**. Yield: 99%. Off-white solid. ^{1}H-NMR (300 MHz, CDCl$_3$): δ = 7.80 - 7.88 (m, 2H), 7.94 - 8.04 (m, 2H), 9.25 (s, 1H), 9.58 (s, 1H), 12.94 (br s, 1H) ppm. ^{13}C-NMR (75 MHz, CDCl$_3$): δ = 120.1, 126.2, 129.8, 130.0, 132.9, 148.2, 156.2, 166.9 ppm.

4-(Oxazol-4-yl)benzoic acid (48s): Preparation according to general procedure H using **70**. Yield: 99%. White solid. ^{1}H-NMR (300 MHz, DMSO-d_6): δ = 7.87 - 7.96 (m, 2H), 7.97 - 8.05 (m, 2H), 8.52 (d, ^4J = 0.9 Hz, 1H), 8.78 (d, ^4J = 0.9 Hz, 1H), 12.96 (br s, 1H) ppm. ^{13}C-NMR (75 MHz, DMSO-d_6): δ = 125.2, 129.9, 130.0, 134.9, 136.6, 138.4, 152.9, 167.0 ppm.

Ethyl 2-(2-[2-naphthamido]benzamido)phenylacetate (50a): Preparation according to general procedure C using **47m** and **49a**. Yield: 73%. White solid. ^{1}H-NMR (400 MHz, DMSO-d_6): δ = 0.96 (t, ^3J = 7.1 Hz, 3H), 3.76 (s, 2H), 3.90 (q, ^3J = 7.1 Hz, 2H), 7.22 - 7.43 (m, 5H), 7.55 - 7.74 (m, 3H), 7.95 (dd, ^3J = 8.6 Hz, ^4J = 1.7 Hz, 1H), 8.00 (d, ^3J = 8.0 Hz, 2H), 8.08 (d, ^3J = 8.6 Hz, 2H), 8.53 (s, 1H), 8.64 (d, ^3J = 8.2 Hz, 1H), 10.34 (s, 1H), 12.18 (s, 1H) ppm. ^{13}C-NMR (101 MHz, DMSO-d_6): δ = 13.8, 37.4, 60.2, 120.9, 121.2, 123.1, 123.2, 126.6, 127.0, 127.5, 127.6, 127.7, 127.8, 128.1, 128.6, 128.8, 129.1, 131.1, 131.4, 131.8, 132.2, 132.5, 134.4, 135.9, 139.3, 164.6, 167.7, 170.8 ppm.

Ethyl 3-(4-[2-{2-naphthamido}benzamido]phenyl)propanoate (50b): Preparation according to general procedure C using **47o** and **49a**. Yield: 61%. Pale yellow solid. ^{1}H-NMR (400 MHz, DMSO-d_6): δ = 1.15 (t, ^3J = 7.1 Hz, 3H), 2.60 (t, ^3J = 7.6 Hz, 2H), 2.83 (t, ^3J = 7.5 Hz, 2H), 4.04 (q, ^3J = 7.1 Hz, 2H), 7.22 (d, ^3J = 8.5 Hz, 2H), 7.31 (ddd, ^3J = 7.8, 7.8 Hz, ^4J = 1.0 Hz, 1H), 7.58 - 7.70 (m, 5H), 7.90 - 7.99 (m, 2H), 8.02 (d, ^3J = 7.7 Hz, 1H), 8.05 - 8.14 (m, 2H), 8.50 (d, ^3J = 8.2 Hz, 1H), 8.54 (s, 1H), 10.50 (s, 1H), 11.83 (s, 1H) ppm. ^{13}C-NMR (75 MHz, DMSO-d_6): δ = 14.1, 29.8, 35.2, 59.8, 121.1, 121.5, 123.2, 123.4, 127.1, 127.7, 127.9, 128.1, 128.4, 128.6, 129.0, 129.1, 131.9, 132.18, 132.21, 134.4, 136.5, 136.6, 138.6, 164.7, 167.3, 172.2 ppm.

Ethyl 3-(3-[2-{2-naphthamido}-3-methylbenzamido]phenyl)propanoate (50c): Preparation according to general procedure B using **47a** and **48a**. Yield: 41%. Beige solid. ^{1}H-NMR (300 MHz, DMSO-d_6): δ = 1.10 (t, ^3J = 7.1 Hz, 3H), 2.32 (s, 3H), 2.53 (m, 2H), 2.76 (t, ^3J = 7.5 Hz, 2H), 3.99 (q, ^3J = 7.1 Hz,

2H), 6.88 (d, ^3J = 7.5, Hz, 1H), 7.17 (dd, ^3J = 7.8, 7.8 Hz, 1H), 7.36 (dd, ^3J = 7.5, 7.5 Hz, 1H), 7.43 - 7.56 (m, 4H), 7.56 - 7.70 (m, 2H), 7.94 - 8.06 (m, 4H), 8.56 (s, 1H), 10.13 (s, 1H), 10.16 (s, 1H) ppm. ^{13}C-NMR (75 MHz, DMSO-d_6): δ = 14.0, 18.1, 30.4, 35.0, 59.8, 117.6, 119.5, 123.3, 124.3, 126.1, 126.4, 126.8, 127.6, 127.8, 128.0, 128.5, 128.8, 131.6, 132.08, 132.11, 134.1, 134.3, 134.9, 136.3, 139.3, 140.9, 165.6, 166.2, 172.0 ppm.

Ethyl 3-(3-[2-{2-naphthamido}-4-methylbenzamido]phenyl)propanoate (50d): Preparation according to general procedure B using **47b** and **48a**. Yield: 84%. Beige solid. ^{1}H-NMR (300 MHz, DMSO-d_6): δ = 1.17 (t, ^3J = 7.1 Hz, 3H), 2.48 (s, 3H), 2.65 (t, ^3J = 7.4 Hz, 2H), 2.90 (t, ^3J = 7.4 Hz, 2H), 4.08 (q, ^3J = 7.1 Hz, 2H), 7.05 (d, ^3J = 7.8, Hz, 1H), 7.17 (dd, ^3J = 8.0 Hz, ^4J = 1.1 Hz, 1H), 7.32 (dd, ^3J = 8.7, 7.6 Hz, 1H), 7.59 - 7.66 (m, 2H), 7.66 - 7.75 (m, 2H), 7.93 (d, ^3J = 8.0 Hz, 1H), 8.02 (dd, ^3J = 8.6 Hz, ^4J = 1.8 Hz, 1H), 8.04 - 8.09 (m, 1H), 8.11 - 8.19 (m, 2H), 8.47 (d, ^4J = 0.9 Hz, 1H), 8.58 (d, ^4J = 1.3 Hz, 1H), 10.49 (s, 1H), 12.06 (s, 1H) ppm. ^{13}C-NMR (75 MHz, DMSO-d_6): δ = 14.0, 21.4, 30.3, 35.0, 59.8, 119.1, 119.7, 121.0, 121.6, 123.4, 123.9, 124.2, 127.0, 127.68, 127.73, 128.1, 128.59, 128.61, 128.9, 129.0, 132.0, 132.2, 134.4, 138.5, 139.1, 141.0, 142.5, 164.6, 167.4, 172.1 ppm.

Ethyl 3-(3-[2-{2-naphthamido}-5-methylbenzamido]phenyl)propanoate (50e): Preparation according to general procedure B using **47c** and **48a**. Yield: 64%. White solid. ^{1}H-NMR (300 MHz, DMSO-d_6): δ = 1.12 (t, ^3J = 7.1 Hz, 3H), 2.40 (s, 3H), 2.60 (t, ^3J = 7.4 Hz, 2H), 2.85 (t, ^3J = 7.4 Hz, 2H), 4.03 (q, ^3J = 7.1 Hz, 2H), 7.00 (d, ^3J = 7.8, Hz, 1H), 7.27 (dd, ^3J = 8.7, 7.6 Hz, 1H), 7.45 (dd, ^3J = 8.5 Hz, ^4J = 1.6 Hz, 1H), 7.55 - 7.61 (m, 2H), 7.61 - 7.69 (m, 2H), 7.75 (d, ^4J = 1.7 Hz, 1H), 7.93 - 8.04 (m, 2H), 8.07 (d, ^4J = 0.8 Hz, 1H), 8.10 (s, 1H), 8.35 (d, ^3J = 8.4 Hz, 1H), 8.53 (d, ^4J = 1.3 Hz, 1H), 10.45 (s, 1H), 11.64 (s, 1H) ppm. ^{13}C-NMR (75 MHz, DMSO-d_6): δ = 14.0, 20.5, 30.3, 35.0, 59.8, 118.9, 120.8, 121.8, 123.4, 124.1, 127.0, 127.6, 127.67, 127.74, 128.0, 128.5, 128.6, 129.0, 129.2, 131.9, 132.2, 132.5, 132.6, 134.4, 136.1, 138.6, 141.0, 164.6, 167.3, 172.1 ppm.

Ethyl 3-(3-[2-{2-naphthamido}-6-methylbenzamido]phenyl)propanoate (50f): Preparation according to general procedure B using **47d** and **48a**. Yield: 42%. Beige solid. ^{1}H-NMR (300 MHz, DMSO-d_6): δ = 1.15 (t, ^3J = 7.1 Hz, 3H), 2.45 (s, 3H), 2.53 – 2.62 (m, 2H), 2.84 (t, ^3J = 7.5 Hz, 2H), 4.04 (q, ^3J = 7.1 Hz, 2H), 6.98 (d, ^3J = 7.6, Hz, 1H), 7.22 - 7.31 (m, 2H), 7.46 (dd, ^3J = 7.8, 7.8 Hz, 1H), 7.55 - 7.71 (m, 5H), 7.95 (dd, ^3J = 8.5 Hz, ^4J = 1.7 Hz, 2H), 8.03 (dd, ^3J = 7.8, 7.8 Hz, 2H), 8.47 (d, ^4J = 1.1 Hz, 1H), 10.12 (s, 1H), 10.34 (s, 1H) ppm. ^{13}C-NMR (75 MHz, DMSO-d_6): δ = 14.0, 19.5, 30.4, 35.0, 59.8, 117.8, 119.7, 123.5, 123.6, 124.1, 126.8, 127.6, 127.7, 127.8, 127.9, 128.1, 128.6, 128.8, 129.1, 131.8, 132.0, 133.4, 134.3, 134.9, 135.5, 138.9, 141.0, 165.8, 165.8, 172.0 ppm.

Ethyl 3-(3-[2-{2-naphthamido}-4-chlorobenzamido]phenyl)propanoate (50g): Preparation according to general procedure B using **47e** and **48a**. Yield: 56%. Pale yellow solid. ^{1}H-NMR (300 MHz, DMSO-d_6): δ = 1.12 (t, ^3J = 7.1 Hz, 3H), 2.59 (t, ^3J = 7.6 Hz, 2H), 2.84 (t, ^3J = 7.4 Hz, 2H), 4.02 (q, ^3J = 7.1 Hz, 2H), 7.01 (d, ^3J = 7.5 Hz, 1H), 7.28 (dd, ^3J = 8.4, 7.7 Hz, 1H), 7.38 (d, ^3J = 8.1 Hz, 1H), 7.54 - 7.61 (m, 2H), 7.61 - 7.70 (m, 2H), 7.94 - 8.05 (m, 3H), 8.06 - 8.13 (m, 2H), 8.54 (d, ^4J = 1.3 Hz, 1H), 8.62 (d, ^4J = 2.2 Hz, 1H), 10.58 (s, 1H), 11.98 (s, 1H) ppm. ^{13}C-NMR (75 MHz, DMSO-d_6): δ = 14.0, 30.3, 35.0, 59.8, 119.0, 120.8, 121.0, 121.5, 121.6, 123.5, 124.3, 127.1, 127.7, 128.0, 128.1, 128.57, 128.64, 129.1, 130.7, 132.2, 132.9, 134.5, 136.5, 138.4, 140.98, 141.04, 162.1, 166.4, 172.1 ppm.

Ethyl 3-(3-[2-{2-naphthamido}-4-methoxybenzamido]phenyl)propanoate (50h): Preparation according to general procedure B using **47f** and **48a**. Yield: 87%. White solid. ^{1}H-NMR (500 MHz, DMSO-d_6): δ = 1.12 (t, ^3J = 7.1 Hz, 3H), 2.62 (t, ^3J = 7.5 Hz, 2H), 2.86 (t, ^3J = 7.4 Hz, 2H), 3.89 (s, 3H), 4.03 (q, ^3J = 7.1 Hz, 2H), 6.87 (dd, ^3J = 8.8 Hz, ^4J = 2.5 Hz, 1H), 7.01 (d, ^3J = 7.6 Hz, 1H), 7.28 (dd, ^3J = 8.1, 8.1 Hz, 1H), 7.54 - 7.60 (m, 2H), 7.60 – 7.71 (m, 2H), 7.98 (dd, ^3J = 8.6 Hz, ^4J = 1.6 Hz, 1H), 7.99 - 8.05 (m, 2H), 8.08 - 8.13 (m, 2H), 8.35 (d, ^4J = 2.6 Hz, 1H), 8.55 (s, 1H), 10.39 (s, 1H), 12.50 (s, 1H) ppm. ^{13}C-NMR (126 MHz, DMSO-d_6): δ = 14.1, 30.4, 35.1, 55.5, 59.9, 105.9, 108.5, 113.6, 119.3, 121.3, 123.3, 124.2, 127.1, 127.8, 127.9, 128.2, 128.6, 128.8, 129.2, 130.8, 131.9, 132.3, 134.5, 138.5, 141.0, 141.5, 162.3, 164.8, 167.5, 172.2 ppm.

Ethyl 3-(3-[2-{2-naphthamido}-4-{trifluoromethoxy}benzamido]phenyl)propanoate (50i): Preparation according to general procedure B using **47g** and **48a**. Yield: 20%. Yellow solid. ^{1}H-NMR (500 MHz, DMSO-d_6): δ = 1.12 (t, ^{3}J = 7.1 Hz, 3H), 2.61 (t, ^{3}J = 7.4 Hz, 2H), 2.85 (t, ^{3}J = 7.3 Hz, 2H), 4.03 (q, ^{3}J = 7.0 Hz, 2H), 7.02 (d, ^{3}J = 7.4 Hz, 1H), 7.21 - 7.38 (m, 2H), 7.51 - 7.74 (m, 4H), 7.96 (d, ^{3}J = 8.5 Hz, 1H), 8.02 (d, ^{3}J = 8.0 Hz, 1H), 8.05 - 8.16 (m, 3H), 8.55 (d, ^{3}J = 8.9 Hz, 2H), 10.60 (s, 1H), 11.98 (s, 1H) ppm. ^{13}C-NMR (126 MHz, DMSO-d_6): δ = 14.1, 30.3, 35.0, 59.9, 112.9, 115.0, 119.0, 120.0, 120.9, 121.8, 123.4, 124.4, 127.1, 127.7, 128.1, 128.3, 128.7, 129.1, 131.3, 131.4, 132.2, 134.5, 138.4, 140.5, 141.1, 150.4, 165.1, 166.3, 172.1 ppm.

Ethyl 3-(3-[2-{2-naphthamido}-4-{tert-butyl}benzamido]phenyl)propanoate (50j): Preparation according to general procedure B using **47h** and **48a**. Yield: 97%. White solid. ^{1}H-NMR (300 MHz, DMSO-d_6): δ = 1.12 (t, ^{3}J = 7.1 Hz, 3H), 1.36 (s, 9H), 2.60 (t, ^{3}J = 7.5 Hz, 2H), 2.85 (t, ^{3}J = 7.4 Hz, 2H), 4.03 (q, ^{3}J = 7.1 Hz, 2H), 6.99 (d, ^{3}J = 7.6, Hz, 1H), 7.27 (dd, ^{3}J = 7.7, 7.7 Hz, 1H), 7.34 (dd, ^{3}J = 8.3 Hz, ^{4}J = 2.0 Hz, 1H), 7.54 - 7.70 (m, 4H), 7.88 (d, ^{3}J = 8.4 Hz, 1H), 7.95 - 8.04 (m, 2H), 8.05 - 8.14 (m, 2H), 8.55 (d, ^{4}J = 1.3 Hz, 1H), 8.62 (d, ^{4}J = 1.9 Hz, 1H), 10.42 (s, 1H), 11.87 (s, 1H) ppm. ^{13}C-NMR (75 MHz, DMSO-d_6): δ = 14.0, 30.3, 30.8, 34.9, 35.0, 59.8, 118.3, 118.9, 120.4, 120.8, 123.4, 124.1, 125.1, 127.0, 127.7, 127.8, 128.1, 128.55, 128.59, 128.8, 129.0, 132.0, 132.2, 134.4, 138.6, 138.6, 141.0, 155.2, 164.8, 167.3, 172.1 ppm.

Ethyl 3-(3-[2-{2-naphthamido}-5-chlorobenzamido]phenyl)propanoate (50k): Preparation according to general procedure B using **47i** and **48a**. Yield: 60%. White solid. ^{1}H-NMR (500 MHz, DMSO-d_6): δ = 1.12 (t, ^{3}J = 7.1 Hz, 3H), 2.60 (t, ^{3}J = 7.5 Hz, 2H), 2.84 (t, ^{3}J = 7.5 Hz, 2H), 4.02 (q, ^{3}J = 7.1 Hz, 2H), 7.01 (d, ^{3}J = 7.6, Hz, 1H), 7.28 (dd, ^{3}J = 8.5, 7.8 Hz, 1H), 7.54 - 7.60 (m, 2H), 7.62 (ddd, ^{3}J = 7.4, 7.4 Hz, ^{4}J = 1.3 Hz, 1H), 7.66 (ddd, ^{3}J = 7.4, 7.4 Hz, ^{4}J = 1.3 Hz, 1H), 7.70 (dd, ^{3}J = 8.9 Hz, ^{4}J = 2.5 Hz, 1H), 7.96 (dd, ^{3}J = 8.6, Hz, ^{4}J = 1.8 Hz, 1H), 7.98 (d, ^{4}J = 2.4 Hz, 1H), 8.01 (d, ^{3}J = 8.0 Hz, 1H), 8.06 - 8.11 (m, 2H), 8.45 (d, ^{3}J = 8.9 Hz, 1H), 8.54 (d, ^{4}J = 0.7 Hz, 1H), 10.58 (s, 1H), 11.67 (s, 1H) ppm. ^{13}C-NMR (126 MHz, DMSO-d_6): δ = 14.1, 30.3, 35.0, 59.9, 118.9, 120.9, 123.48, 123.54, 124.3, 125.3, 127.1, 127.3, 127.7, 128.0, 128.2, 128.59, 128.62, 128.7, 129.1, 131.6, 131.7, 132.2, 134.5, 137.3, 138.4, 141.1, 164.9, 165.8, 172.1 ppm.

Ethyl 3-(3-[2-{2-naphthamido}-5-methoxybenzamido]phenyl)propanoate (50l): Preparation according to general procedure B using **47j** and **48a**. Yield: 92%. Brown solid. ^{1}H-NMR (500 MHz, DMSO-d_6): δ = 1.12 (t, ^{3}J = 7.1 Hz, 3H), 2.59 (t, ^{3}J = 7.5 Hz, 2H), 2.83 (t, ^{3}J = 7.5 Hz, 2H), 3.87 (s, 3H), 4.02 (q, ^{3}J = 7.1 Hz, 2H), 6.99 (d, ^{3}J = 7.7 Hz, 1H), 7.23 (dd, ^{3}J = 9.0 Hz, ^{4}J = 2.9 Hz, 1H), 7.26 (dd, ^{3}J = 8.6, 7.7 Hz, 1H), 7.42 (d, ^{4}J = 2.8 Hz, 1H), 7.54 - 7.59 (m, 2H), 7.59 - 7.67 (m, 2H), 7.95 (dd, ^{3}J = 8.6 Hz, ^{4}J = 1.7 Hz, 1H), 8.00 (d, ^{3}J = 7.8 Hz, 1H), 8.07 (d, ^{3}J = 8.2 Hz, 2H), 8.19 - 8.30 (m, 1H), 8.52 (d, ^{4}J = 1.0 Hz, 1H) 10.45 (s, 1H), 11.32 (s, 1H) ppm. ^{13}C-NMR (126 MHz, DMSO-d_6): δ = 14.1, 30.3, 35.0, 55.6, 59.8, 113.9, 117.4, 118.8, 118.9, 120.7, 120.8, 123.6, 124.0, 124.1, 127.0, 127.69, 127.74, 128.0, 128.5, 128.6, 129.0, 132.2, 134.3, 138.5, 138.6, 141.0, 155.1, 164.6, 166.7, 172.1 ppm.

Ethyl 3-(3-[2-{2-naphthamido}-5-{trifluoromethoxy}benzamido]phenyl)propanoate (50m): Preparation according to general procedure B using **47k** and **48a**. Yield: 64%. White solid. ^{1}H-NMR (300 MHz, Acetone-d_6): δ = 1.17 (t, ^{3}J = 7.1 Hz, 3H), 2.64 (t, ^{3}J = 7.5 Hz, 2H), 2.95 (t, ^{3}J = 7.5 Hz, 2H), 4.08 (q, ^{3}J = 7.1 Hz, 2H), 7.10 (ddd, ^{3}J = 7.6 Hz, ^{4}J = 1.6, 1.1 Hz, 1H), 7.33 (dd, ^{3}J = 8.7, 7.6 Hz, 1H), 7.59 - 7.71 (m, 5H), 7.98 - 8.05 (m, 2H), 8.07 - 8.14 (m, 3H), 8.61 (d, ^{4}J = 0.8 Hz, 1H), 8.97 (d, ^{3}J = 9.2 Hz, 1H), 9.96 (s, 1H), 12.19 (s, 1H) ppm. ^{13}C-NMR (75 MHz, Acetone-d_6): δ = 14.5, 31.6, 36.2, 60.7, 120.1, 121.5, 122.1, 122.3, 123.4, 123.7, 124.4, 125.8, 126.0, 127.9, 128.7, 128.97, 128.99, 129.6, 129.7, 130.1, 133.0, 133.7, 136.0, 139.2, 140.2, 142.7, 144.3, 165.8, 167.4, 172.8 ppm.

Ethyl 3-(3-[6-{2-naphthamido}benzo[1,3]dioxole-5-carboxamido]phenyl)propanoate (50n): Preparation according to general procedure B using **47l** and **48a**. Yield: 84%. Beige solid. ^{1}H-NMR (500 MHz, DMSO-d_6): δ = 1.12 (t, ^{3}J = 7.1 Hz, 3H), 2.60 (t, ^{3}J = 7.5 Hz, 2H), 2.85 (t, ^{3}J = 7.5 Hz, 2H), 4.02 (q, ^{3}J = 7.1 Hz, 2H), 6.18 (s, 2H), 7.00 (d, ^{3}J = 7.6, Hz, 1H), 7.27 (dd, ^{3}J = 7.7, 7.7 Hz, 1H), 7.52 - 7.57 (m, 2H), 7.58 (s, 1H), 7.62 (ddd, ^{3}J = 7.4, 7.4 Hz, ^{4}J = 1.2 Hz, 1H), 7.65 (ddd, ^{3}J = 7.4,

7.4 Hz, ^4J = 1.2 Hz, 1H), 7.96 (dd, ^3J = 8.6 Hz, ^4J = 1.7 Hz, 1H), 8.01 (d, ^3J = 8.0 Hz, 1H), 8.06 - 8.11 (m, 2H), 8.19 (s, 1H), 8.53 (d, ^4J = 0.8 Hz, 1H), 10.28 (s, 1H), 12.26 (s, 1H) ppm. ^{13}C-NMR (126 MHz, DMSO-d_6): δ = 14.1, 30.3, 35.0, 59.9, 102.21, 102.23, 108.1, 114.8, 119.2, 121.1, 123.3, 124.2, 127.1, 127.7, 127.8, 128.1, 128.61, 128.63, 129.1, 131.8, 132.2, 134.4, 135.6, 138.5, 141.0, 142.8, 150.1, 164.6, 167.0, 172.1 ppm.

Ethyl 3-(3-[2-{2H-chromene-3-carboxamido}benzamido]phenyl)propanoate (50o): Preparation according to general procedure C using **47n** and **49b**. Yield: 62%. Pale yellow solid. ^{1}H-NMR (400 MHz, DMSO-d_6): δ = 1.14 (t, ^3J = 7.1 Hz, 3H), 2.61 (t, ^3J = 7.5 Hz, 2H), 2.86 (t, ^3J = 7.5 Hz, 2H), 4.04 (q, ^3J = 7.1 Hz, 2H), 4.99 (d, ^4J = 1.1 Hz, 2H), 6.87 (d, ^3J = 8.1 Hz, 1H), 6.94 - 7.05 (m, 2H), 7.23 - 7.33 (m, 4H), 7.35 (s, 1H), 7.54 - 7.63 (m, 3H), 7.89 (dd, ^3J = 7.9 Hz, ^4J = 1.2 Hz, 1H), 8.30 (d, ^3J = 8.2 Hz, 1H), 10.45 (br s, 1H), 11.31 (br s, 1H) ppm. ^{13}C-NMR (126 MHz, DMSO-d_6): δ = 14.1, 30.4, 35.0, 59.9, 64.2, 115.7, 118.9, 120.87, 120.91, 121.6, 121.9, 123.3, 123.4, 124.2, 127.1, 127.9, 128.6, 128.9, 129.0, 131.6, 132.1, 138.2, 138.6, 141.0, 154.2, 162.7, 167.2, 172.1 ppm.

Ethyl 3-(3-[2-{4-chloro-2H-chromene-3-carboxamido}benzamido]phenyl)propanoate (50p): Preparation according to general procedure B using **47n** and **48c**. Yield: 38%. Yellow solid. ^{1}H-NMR (500 MHz, DMSO-d_6): δ = 1.14 (t, ^3J = 7.1 Hz, 3H), 2.60 (t, ^3J = 7.5 Hz, 2H), 2.84 (t, ^3J = 7.5 Hz, 2H), 4.03 (q, ^3J = 7.1 Hz, 2H), 5.01 (s, 2H), 6.96 (dd, ^3J = 8.1 Hz, ^4J = 0.7 Hz, 1H), 6.99 (d, ^3J = 7.7 Hz, 1H), 7.10 (ddd, ^3J = 7.7, 7.7 Hz, ^4J = 0.9 Hz, 1H), 7.26 (dd, ^3J = 7.8, 7.8 Hz, 1H), 7.31 (d, ^3J = 7.6 Hz, 1H), 7.37 (ddd, ^3J = 8.0, 8.0 Hz, ^4J = 1.5 Hz, 1H), 7.53 - 7.63 (m, 4H), 7.83 (d, ^3J = 7.7 Hz, 1H), 8.27 (d, ^3J = 8.1 Hz, 1H), 10.46 (s, 1H), 11.13 (s, 1H) ppm. ^{13}C-NMR (126 MHz, DMSO-d_6): δ = 14.1, 30.4, 35.0, 59.9, 66.6, 116.1, 118.7, 120.5, 120.6, 122.0, 122.3, 124.0, 124.1, 124.6, 125.77, 125.78, 125.9, 128.6, 128.9, 131.8, 132.2, 136.9, 138.6, 141.0, 154.5, 161.9, 166.7, 172.1 ppm.

Ethyl 3-(3-[2-{5-methyl-2H-chromene-3-carboxamido}benzamido]phenyl)propanoate (50q): Preparation according to general procedure B using **47n** and **48d**. Yield: 88%. Yellow solid. ^{1}H-NMR (400 MHz, DMSO-d_6): δ = 1.15 (t, ^3J = 7.1 Hz, 3H), 2.37 (s, 3H), 2.60 (t, ^3J = 7.6 Hz, 2H), 2.84 (t, ^3J = 7.6 Hz, 2H), 4.04 (q, ^3J = 7.1 Hz, 2H), 4.92 (s, 2H), 6.73 (d, ^3J = 8.1 Hz, 1H), 6.84 (d, ^3J = 7.5 Hz, 1H), 7.00 (d, ^3J = 7.7 Hz, 1H), 7.16 (dd, ^3J = 7.8, 7.8 Hz, 1H), 7.23 - 7.32 (m, 2H), 7.52 - 7.63 (m, 3H), 7.70 (s, 1H), 7.90 (d, ^3J = 7.7 Hz, 1H), 8.30 (d, ^3J = 8.3 Hz, 1H), 10.46 (s, 1H), 11.48 (s, 1H) ppm. ^{13}C-NMR (75 MHz, DMSO-d_6): 14.1, 17.8, 30.4, 35.1, 59.8, 63.4, 113.6, 118.7, 119.7, 120.7, 121.4, 123.3, 123.4, 124.1, 125.1, 126.8, 128.6, 128.9, 131.0, 132.1, 136.3, 138.2, 138.6, 141.0, 154.5, 162.7, 167.2, 172.0 ppm.

Ethyl 3-(3-[2-{6-methyl-2H-chromene-3-carboxamido}benzamido]phenyl)propanoate (50r): Preparation according to general procedure B using **47n** and **48e**. Yield: 94%. Yellow solid. ^{1}H-NMR (400 MHz, DMSO-d_6): δ = 1.14 (t, ^3J = 7.1 Hz, 3H), 2.23 (s, 3H), 2.61 (t, ^3J = 7.5 Hz, 2H), 2.86 (t, ^3J = 7.5 Hz, 2H), 4.04 (q, ^3J = 7.1 Hz, 2H), 4.95 (s, 2H), 6.77 (d, ^3J = 8.1 Hz, 1H), 7.01 (d, ^3J = 7.6 Hz, 1H), 7.08 (d, ^3J = 8.3 Hz, 1H), 7.10 (s, 1H), 7.23 -7.33 (m, 3H), 7.48 – 7.66 (m, 3H), 7.89 (d, ^3J = 7.2 Hz, 1H), 8.30 (d, ^3J = 8.2 Hz, 1H), 10.45 (s, 1H), 11.30 (s, 1H) ppm. ^{13}C-NMR (75 MHz, DMSO-d_6): 14.0, 20.0, 30.3, 35.0, 59.8, 64.1, 115.5, 119.0, 120.7, 120.9, 121.5, 123.1, 123.3, 124.2, 127.1, 128.0, 128.6, 129.0, 130.7, 132.0, 132.1, 138.2, 138.5, 141.0, 152.1, 162.7, 167.2, 172.1 ppm.

Ethyl 3-(3-[2-{6-fluoro-2H-chromene-3-carboxamido}benzamido]phenyl)propanoate (50s): Preparation according to general procedure B using **47n** and **48f**. Yield: 90%. Yellow solid. ^{1}H-NMR (400 MHz, DMSO-d_6): δ = 1.14 (t, ^3J = 7.1 Hz, 3H), 2.61 (t, ^3J = 7.5 Hz, 2H), 2.85 (t, ^3J = 7.5 Hz, 2H), 4.04 (q, ^3J = 7.1 Hz, 2H), 4.98 (d, ^4J = 1.0 Hz, 2H), 6.90 (dd, ^3J = 8.9 Hz, ^4J = 4.6 Hz, 1H), 7.01 (d, ^3J = 7.6 Hz, 1H), 7.10 (ddd, ^3J = 8.7, 8.7 Hz, ^4J = 3.1 Hz, 1H), 7.20 - 7.31 (m, 3H), 7.33 (s, 1H), 7.53 - 7.63 (m, 3H), 7.89 (dd, ^3J = 7.8 Hz, ^4J = 1.0 Hz, 1H), 8.27 (d, ^3J = 7.9 Hz, 1H), 10.45 (s, 1H), 11.29 (s, 1H) ppm. ^{13}C-NMR (75 MHz, DMSO-d_6): δ = 14.0, 30.3, 35.0, 59.8, 64.3, 114.6, 117.0, 117.6, 118.9, 120.8, 121.7, 122.1, 123.4, 123.5, 124.2, 127.1, 128.6, 128.7, 128.9, 132.1, 138.0, 138.5, 141.0, 150.3, 156.9, 162.4, 167.1, 172.1 ppm.

Ethyl 3-(3-[2-{6-chloro-2H-chromene-3-carboxamido}benzamido]phenyl)propanoate (50t): Preparation according to general procedure B using **47n** and **48g**. Yield: 84%. Pale yellow solid. ^{1}H-NMR (400 MHz, DMSO-d_6): δ = 1.14 (t, ^{3}J = 7.1 Hz, 3H), 2.61 (t, ^{3}J = 7.5 Hz, 2H), 2.85 (t, ^{3}J = 7.5 Hz, 2H), 4.04 (q, ^{3}J = 7.1 Hz, 2H), 5.02 (d, ^{4}J = 0.9 Hz, 2H), 6.90 (d, ^{3}J = 8.6 Hz, 1H), 7.01 (d, ^{3}J = 7.6 Hz, 1H), 7.22 - 7.31 (m, 3H), 7.33 (s, 1H), 7.42 (d, ^{3}J = 2.6 Hz, 1H), 7.53 - 7.62 (m, 3H), 7.89 (d, ^{3}J = 6.9 Hz, 1H), 8.27 (d, ^{3}J = 8.0 Hz, 1H), 10.45 (s, 1H), 11.28 (s, 1H) ppm. ^{13}C-NMR (75 MHz, DMSO-d_6): δ = 14.1, 30.3, 35.0, 59.8, 64.5, 117.4, 118.9, 120.8, 121.7, 122.6, 123.4, 123.5, 124.2, 125.4, 126.7, 127.9, 128.5, 128.6, 128.9, 130.8, 132.1, 138.0, 138.5, 141.0, 152.9, 162.4, 167.1, 172.1 ppm.

Ethyl 3-(3-[2-{7-methyl-2H-chromene-3-carboxamido}benzamido]phenyl)propanoate (50u): Preparation according to general procedure C using **47n** and **49c**. Yield: 52%. Pale yellow solid. ^{1}H-NMR (500 MHz, DMSO-d_6): δ = 1.14 (t, ^{3}J = 7.1 Hz, 3H), 2.27 (s, 3H), 2.61 (t, ^{3}J = 7.5 Hz, 2H), 2.85 (t, ^{3}J = 7.5 Hz, 2H), 4.04 (q, ^{3}J = 7.1 Hz, 2H), 4.96 (d, ^{4}J = 0.8 Hz, 2H), 6.71 (s, 1H), 6.80 (d, ^{3}J = 7.6 Hz, 1H), 7.01 (d, ^{3}J = 7.6 Hz, 1H), 7.19 (d, ^{3}J = 7.6 Hz, 1H), 7.23 - 7.30 (m, 2H), 7.32 (s, 1H), 7.52 - 7.65 (m, 3H), 7.89 (d, ^{3}J = 7.7 Hz, 1H), 8.29 (d, ^{3}J = 8.1 Hz, 1H), 10.45 (s, 1H), 11.27 (s, 1H) ppm. ^{13}C-NMR (126 MHz, DMSO-d_6): δ 14.1, 21.2, 30.3, 35.0, 59.9, 64.2, 116.2, 118.3, 118.9, 120.9, 121.5, 122.7, 123.2, 123.3, 124.2, 125.9, 128.0, 128.6, 128.7, 128.9, 132.1, 138.2, 138.6, 141.0, 141.9, 154.2, 162.7, 167.2, 172.1 ppm.

Ethyl 3-(3-[2-{8-methyl-2H-chromene-3-carboxamido}benzamido]phenyl)propanoate (50v): Preparation according to general procedure B using **47n** and **48i**. Yield: 99%. Yellow solid. ^{1}H-NMR (400 MHz, DMSO-d_6): δ = 1.14 (t, ^{3}J = 7.1 Hz, 3H), 2.14 (s, 3H), 2.61 (t, ^{3}J = 7.5 Hz, 2H), 2.86 (t, ^{3}J = 7.5 Hz, 2H), 4.04 (q, ^{3}J = 7.1 Hz, 2H), 5.01 (s, 2H), 6.87 (dd, ^{3}J = 7.5, 7.5 Hz, 1H), 7.01 (d, ^{3}J = 7.6 Hz, 1H), 7.10 - 7.20 (m, 2H), 7.21 - 7.30 (m, 2H), 7.33 (s, 1H), 7.53 - 7.65 (m, 3H), 7.89 (d, ^{3}J = 7.8 Hz, 1H), 8.31 (d, ^{3}J = 8.3 Hz, 1H), 10.45 (s, 1H), 11.30 (s, 1H) ppm. ^{13}C-NMR (75 MHz, DMSO-d_6): 14.1, 15.2, 30.3, 35.0, 59.8, 64.2, 119.0, 120.4, 120.9, 121.3, 121.5, 123.1, 123.3, 124.2, 124.6, 126.5, 126.7, 128.3, 128.6, 128.9, 132.1, 132.9, 138.2, 138.5, 141.0, 152.2, 162.6, 167.2, 172.1 ppm.

Ethyl 3-(3-[2-{8-chloro-2H-chromene-3-carboxamido}benzamido]phenyl)propanoate (50w): Preparation according to general procedure B using **47n** and **48j**. Yield: 89%. Pale yellow solid. ^{1}H-NMR (400 MHz, DMSO-d_6): δ = 1.14 (t, ^{3}J = 7.1 Hz, 3H), 2.61 (t, ^{3}J = 7.5 Hz, 2H), 2.85 (t, ^{3}J = 7.5 Hz, 2H), 4.04 (q, ^{3}J = 7.1 Hz, 2H), 5.12 (d, ^{4}J = 1.0 Hz, 2H), 6.91 - 7.05 (m, 2H), 7.22 - 7.34 (m, 3H), 7.37 (s, 1H), 7.40 (dd, ^{3}J = 8.0 Hz, ^{4}J = 1.3 Hz, 1H), 7.52 - 7.67 (m, 3H), 7.89 (d, ^{3}J = 7.9 Hz, 1H), 8.27 (d, ^{3}J = 7.9 Hz, 1H), 10.45 (s, 1H), 11.29 (s, 1H) ppm. ^{13}C-NMR (75 MHz, DMSO-d_6): 14.1, 30.3, 35.0, 59.8, 65.0, 118.9, 119.8, 120.9, 121.7, 122.5, 123.5, 124.2, 127.1, 127.6, 127.9, 128.6, 129.0, 131.5, 132.1, 137.9, 138.5, 141.0, 149.7, 162.3, 167.1, 172.1 ppm.

Ethyl 3-(3-[2-{8-methoxy-2H-chromene-3-carboxamido}benzamido]phenyl)propanoate (50x): Preparation according to general procedure C using **47n** and **49d**. Yield: 65%. Yellow solid. ^{1}H-NMR (400 MHz, DMSO-d_6): δ = 1.14 (t, ^{3}J = 7.1 Hz, 3H), 2.61 (t, ^{3}J = 7.5 Hz, 2H), 2.86 (t, ^{3}J = 7.5 Hz, 2H), 3.77 (s, 3H), 4.04 (q, ^{3}J = 7.1 Hz, 2H), 4.97 (s, 2H), 6.85 - 6.98 (m, 2H), 6.97 - 7.04 (m, 2H), 7.28 (dd, ^{3}J = 7.8, 7.8 Hz, 2H), 7.33 (s, 1H), 7.51 - 7.64 (m, 3H), 7.89 (d, ^{3}J = 7.2 Hz, 1H), 8.29 (d, ^{3}J = 8.2 Hz, 1H), 10.45 (s, 1H), 11.31 (s, 1H) ppm. ^{13}C-NMR (75 MHz, DMSO-d_6): δ = 14.1, 30.4, 35.0, 55.7, 59.9, 64.1, 114.8, 118.9, 120.5, 120.8, 121.58, 121.62, 123.3, 123.4, 124.2, 127.2, 128.1, 128.6, 129.0, 132.1, 138.2, 138.6, 141.0, 143.2, 147.6, 162.7, 167.1, 172.1 ppm.

Ethyl 3-(3-[2-{8-hydroxy-2H-chromene-3-carboxamido}benzamido]phenyl)propanoate (50y): Preparation according to general procedure N using **50x**. Yield: 19%. Yellow solid. ^{1}H-NMR (300 MHz, DMSO-d_6): δ = 1.14 (t, ^{3}J = 7.1 Hz, 3H), 2.61 (t, ^{3}J = 7.5 Hz, 2H), 2.86 (t, ^{3}J = 7.4 Hz, 2H), 4.04 (q, ^{3}J = 7.1 Hz, 2H), 4.97 (d, ^{4}J = 1.2 Hz, 2H), 6.72 - 6.86 (m, 3H), 7.01 (d, ^{3}J = 7.8 Hz, 1H), 7.21 - 7.36 (m, 3H), 7.52 - 7.63 (m, 3H), 7.89 (dd, ^{3}J = 7.9 Hz, ^{4}J = 1.3 Hz, 1H), 8.31 (dd, ^{3}J = 8.3 Hz, ^{4}J = 1.0 Hz, 1H), 9.27 (s, 1H), 10.44 (s, 1H), 11.32 (s, 1H) ppm. ^{13}C-NMR (75 MHz, DMSO-d_6):

δ = 14.1, 30.4, 35.0, 59.9, 64.1, 118.8, 118.9, 119.2, 120.9, 121.5, 121.7, 121.8, 123.1, 123.3, 124.2, 127.0, 128.4, 128.6, 128.9, 132.1, 138.2, 138.5, 141.0, 142.0, 145.3, 162.7, 167.2, 172.1 ppm.

Ethyl 3-(3-[2-{(1,1'-biphenyl)-4-carboxamido}benzamido]phenyl)propanoate (50z): Preparation according to general procedure B using **47n** and **48l**. Yield: 49%. Off-white solid. ^{1}H-NMR (300 MHz, DMSO-d_6): δ = 1.13 (t, ^{3}J = 7.1 Hz, 3H), 2.61 (t, ^{3}J = 7.4 Hz, 2H), 2.86 (t, ^{3}J = 7.4 Hz, 2H), 4.03 (q, ^{3}J = 7.1 Hz, 2H), 7.02 (d, ^{3}J = 7.8 Hz, 1H), 7.23 - 7.36 (m, 2H), 7.37 - 7.58 (m, 6H), 7.70 - 7.81 (m, 2H), 7.87 (d, ^{3}J = 8.4 Hz, 2H), 7.93 (d, ^{3}J = 6.8 Hz, 1H), 8.01 (d, ^{3}J = 8.4 Hz, 2H), 8.51 (d, ^{3}J = 8.2 Hz, 1H), 10.50 (s, 1H), 11.76 (s, 1H) ppm. ^{13}C-NMR (75 MHz, DMSO-d_6): δ = 14.1, 30.3, 35.0, 59.9, 119.2, 121.1, 121.3, 122.8, 123.3, 124.3, 126.9, 127.1, 127.7, 128.2, 128.6, 129.0, 129.1, 132.3, 133.2, 138.4, 138.7, 138.9, 141.1, 143.6, 164.3, 167.4, 172.1 ppm.

Ethyl 3-(3-[2-{4-(2-furyl)benzamido}benzamido]phenyl)propanoate (50aa): Preparation according to general procedure B using **47n** and **48m**. Yield: 48%. Brown solid. ^{1}H-NMR (300 MHz, DMSO-d_6): δ = 1.14 (t, ^{3}J = 7.1 Hz, 3H), 2.61 (t, ^{3}J = 7.4 Hz, 2H), 2.86 (t, ^{3}J = 7.4 Hz, 2H), 4.04 (q, ^{3}J = 7.1 Hz, 2H), 6.65 (dd, ^{3}J = 3.4, 1.8 Hz, 1H), 7.01 (d, ^{3}J = 7.7 Hz, 1H), 7.13 (dd, ^{3}J = 3.4 Hz, ^{4}J = 0.6 Hz, 1H), 7.24 - 7.34 (m, 2H), 7.54 - 7.66 (m, 3H), 7.83 (dd, ^{3}J = 1.7 Hz, ^{4}J = 0.6 Hz, 1H), 7.88 (d, ^{3}J = 8.6 Hz, 2H), 7.93 (dd, ^{3}J = 8.0 Hz, ^{4}J = 1.4 Hz, 1H), 7.97 (d, ^{3}J = 8.7 Hz, 2H), 8.48 (dd, ^{3}J = 8.3 Hz, ^{4}J = 0.9 Hz, 1H), 10.49 (s, 1H), 11.72 (s, 1H) ppm. ^{13}C-NMR (75 MHz, DMSO-d_6): δ = 14.1, 30.3, 35.0, 59.8, 108.0, 112.4, 119.1, 121.0, 121.4, 122.9, 123.3, 123.6, 124.2, 127.8, 128.6, 129.0, 132.2, 132.8, 133.3, 138.5, 138.6, 141.0, 144.0, 152.0, 164.1, 167.4, 172.1 ppm.

Ethyl 3-(3-[2-{4-(3-furyl)benzamido}benzamido]phenyl)propanoate (50ab): Preparation according to general procedure B using **47n** and **48n**. Yield: 65%. Beige solid. ^{1}H-NMR (300 MHz, DMSO-d_6): δ = 1.14 (t, ^{3}J = 7.1 Hz, 3H), 2.61 (t, ^{3}J = 7.5 Hz, 2H), 2.86 (t, ^{3}J = 7.4 Hz, 2H), 4.04 (q, ^{3}J = 7.1 Hz, 2H), 7.02 (d, ^{3}J = 7.7 Hz, 1H), 7.05 (dd, ^{3}J = 1.9 Hz, ^{4}J = 0.8 Hz, 1H), 7.25 - 7.33 (m, 2H), 7.49 - 7.69 (m, 3H), 7.79 (dd, ^{4}J = 1.7, 1.7 Hz, 1H), 7.82 (d, ^{3}J = 8.5 Hz, 2H), 7.93 (d, ^{3}J = 8.5 Hz, 3H), 8.33 (dd, ^{3}J = 1.4 Hz, ^{4}J = 0.9 Hz, 1H), 8.50 (dd, ^{3}J = 8.3 Hz, ^{4}J = 0.9 Hz, 1H), 10.49 (s, 1H), 11.73 (s, 1H) ppm. ^{13}C-NMR (75 MHz, DMSO-d_6): δ = 14.0, 30.3, 35.0, 59.8, 108.6, 119.1, 121.0, 121.2, 122.7, 123.2, 124.2, 125.0, 125.8, 127.6, 128.6, 129.0, 132.2, 132.6, 135.6, 138.4, 138.7, 140.6, 141.0, 144.6, 164.2, 167.4, 172.1 ppm.

Ethyl 3-(3-[2-{4-(2-thienyl)benzamido}benzamido]phenyl)propanoate (50ac): Preparation according to general procedure B using **47n** and **48o**. Yield: 90%. Yellow solid. ^{1}H-NMR (500 MHz, DMSO-d_6): δ = 1.14 (t, ^{3}J = 7.1 Hz, 3H), 2.61 (t, ^{3}J = 7.5 Hz, 2H), 2.86 (t, ^{3}J = 7.5 Hz, 2H), 4.04 (q, ^{3}J = 7.1 Hz, 2H), 7.02 (d, ^{3}J = 7.6 Hz, 1H), 7.19 (dd, ^{3}J = 5.0, 3.7 Hz, 1H), 7.26 - 7.32 (m, 2H), 7.56 (s, 1H), 7.58 (d, ^{3}J = 8.1 Hz, 1H), 7.62 (ddd, ^{3}J = 7.9, 7.9 Hz, ^{4}J = 1.3 Hz, 1H), 7.65 (dd, ^{3}J = 5.1 Hz, ^{4}J = 0.9 Hz, 1H), 7.68 (dd, ^{3}J = 3.6 Hz, ^{4}J = 0.9 Hz, 1H), 7.86 (d, ^{3}J = 8.4 Hz, 2H), 7.93 (dd, ^{3}J = 8.0 Hz, ^{4}J = 1.0 Hz, 1H), 7.95 (d, ^{3}J = 8.5 Hz, 2H), 8.49 (d, ^{3}J = 8.0 Hz, 1H), 10.50 (s, 1H), 11.74 (s, 1H) ppm. ^{13}C-NMR (126 MHz, DMSO-d_6): δ = 14.1, 30.3, 35.0, 59.9, 119.1, 121.0, 121.3, 122.9, 123.3, 124.3, 125.2, 125.6, 127.2, 128.0, 128.6, 128.8, 129.0, 132.3, 133.0, 137.1, 138.5, 138.7, 141.0, 142.0, 164.0, 167.4, 172.1 ppm.

Ethyl 3-(3-[2-{4-(3-thienyl)benzamido}benzamido]phenyl)propanoate (50ad): Preparation according to general procedure B using **47n** and **48p**. Yield: 44%. Beige solid. ^{1}H-NMR (300 MHz, DMSO-d_6): δ = 1.14 (t, ^{3}J = 7.1 Hz, 3H), 2.61 (t, ^{3}J = 7.4 Hz, 2H), 2.86 (t, ^{3}J = 7.5 Hz, 2H), 4.04 (q, ^{3}J = 7.1 Hz, 2H), 7.02 (d, ^{3}J = 7.8 Hz, 1H), 7.24 - 7.36 (m, 2H), 7.52 - 7.73 (m, 5H), 7.89 - 8.00 (m, 5H), 8.06 (dd, ^{3}J = 2.8 Hz, ^{4}J = 1.4 Hz, 1H), 8.51 (dd, ^{3}J = 8.3 Hz, ^{4}J = 0.8 Hz, 1H), 10.50 (s, 1H), 11.74 (s, 1H) ppm. ^{13}C-NMR (75 MHz, DMSO-d_6): δ = 14.0, 30.3, 35.0, 59.8, 119.1, 121.0, 121.2, 122.70, 122.74, 123.2, 124.2, 126.2, 126.3, 127.5, 127.7, 128.6, 129.0, 132.2, 132.7, 138.40, 138.43, 138.7, 140.3, 141.0, 164.2, 167.4, 172.1 ppm.

Ethyl 3-(3-[2-{4-(isoxazol-3-yl)benzamido}benzamido]phenyl)propanoate (50ae): Preparation according to general procedure B using **47n** and **48q**. Yield: 41%. Brown solid. ^{1}H-NMR (300 MHz,

DMSO-d_6): δ = 1.13 (t, ^3J = 7.1 Hz, 3H), 2.61 (t, ^3J = 7.5 Hz, 2H), 2.86 (t, ^3J = 7.4 Hz, 2H), 4.03 (q, ^3J = 7.1 Hz, 2H), 7.01 (d, ^3J = 7.7 Hz, 1H), 7.24 (d, ^4J = 1.7 Hz, 1H), 7.25 - 7.36 (m, 2H), 7.52 - 7.68 (m, 3H), 7.93 (dd, ^3J = 7.9 Hz, ^4J = 1.4 Hz, 1H), 8.03 - 8.07 (m, 2H), 8.08 - 8.13 (m, 2H), 8.47 (dd, ^3J = 8.3 Hz, ^4J = 0.9 Hz, 1H), 9.06 (d, ^4J = 1.7 Hz, 1H), 10.50 (s, 1H), 11.77 (s, 1H) ppm. ^{13}C-NMR (75 MHz, DMSO-d_6): δ = 14.0, 30.3, 35.0, 59.8, 103.2, 119.1, 121.0, 121.5, 123.2, 123.5, 124.2, 127.2, 127.9, 128.6, 129.0, 131.6, 132.2, 135.8, 138.46, 138.48, 141.0, 160.3, 161.1, 164.0, 167.3, 172.1 ppm.

Ethyl 3-(3-[2-{4-(isoxazol-4-yl)benzamido}benzamido]phenyl)propanoate (50af): Preparation according to general procedure B using **47n** and **48r**. Yield: 50%. Yellow solid. ^{1}H-NMR (300 MHz, $CDCl_3$): δ = 1.14 (t, ^3J = 7.1 Hz, 3H), 2.61 (t, ^3J = 7.4 Hz, 2H), 2.85 (t, ^3J = 7.4 Hz, 2H), 4.03 (q, ^3J = 7.1 Hz, 2H), 7.01 (d, ^3J = 8.1 Hz, 1H), 7.21 - 7.37 (m, 2H), 7.52 - 7.56 (m, 1H), 7.57 - 7.71 (m, 2H), 7.88 - 7.95 (m, 3H), 7.96 - 8.01 (m, 2H), 8.47 (dd, ^3J = 8.3 Hz, ^4J = 1.0 Hz, 1H), 9.26 (s, 1H), 9.58 (s, 1H), 10.49 (s, 1H), 11.72 (s, 1H) ppm. ^{13}C-NMR (75 MHz, DMSO-d_6): δ = 14.0, 30.3, 35.0, 59.8, 119.0, 120.0, 121.0, 121.4, 123.0, 123.4, 124.2, 126.5, 127.8, 128.6, 129.0, 132.1, 132.2, 133.5, 138.5, 138.6, 141.0, 148.2, 156.0, 164.0, 167.3, 172.1 ppm.

Ethyl 3-(3-[2-{4-(oxazol-4-yl)benzamido}benzamido]phenyl)propanoate (50ag): Preparation according to general procedure B using **47n** and **48s**. Yield: 55%. Pale yellow solid. ^{1}H-NMR (300 MHz, DMSO-d_6): δ = 1.13 (t, ^3J = 7.1 Hz, 3H), 2.61 (t, ^3J = 7.5 Hz, 2H), 2.86 (t, ^3J = 7.4 Hz, 2H), 4.03 (q, ^3J = 7.1 Hz, 2H), 7.01 (d, ^3J = 7.7 Hz, 1H), 7.23 - 7.33 (m, 2H), 7.55 - 7.66 (m, 3H), 7.94 (dd, ^3J = 7.9 Hz, ^4J = 1.3 Hz, 1H), 7.99 (s, 4H), 8.45 - 8.55 (m, 2H), 8.75 (d, ^4J = 0.9 Hz, 1H), 10.50 (s, 1H), 11.76 (s, 1H) ppm. ^{13}C-NMR (75 MHz, DMSO-d_6): δ = 14.0, 30.4, 35.0, 59.9, 119.1, 121.0, 121.4, 122.9, 123.3, 124.2, 125.5, 127.7, 128.6, 129.0, 132.2, 133.7, 134.2, 136.5, 138.4, 138.5, 138.7, 141.0, 152.9, 164.2, 167.4, 172.1 ppm.

Ethyl 3-(3-[2-{4-(oxazol-5-yl)benzamido}benzamido]phenyl)propanoate (50ah): Preparation according to general procedure B using **47n** and **48t**. Yield: 77%. White solid. ^{1}H-NMR (300 MHz, DMSO-d_6): δ = 1.14 (t, ^3J = 7.1 Hz, 3H), 2.61 (t, ^3J = 7.5 Hz, 2H), 2.86 (t, ^3J = 7.4 Hz, 2H), 4.04 (q, ^3J = 7.1 Hz, 2H), 7.01 (d, ^3J = 7.7 Hz, 1H), 7.22 - 7.36 (m, 2H), 7.54 - 7.67 (m, 3H), 7.86 (s, 1H), 7.92 (d, ^3J = 8.3 Hz, 3H), 8.02 (d, ^3J = 8.5 Hz, 2H), 8.45 (d, ^3J = 8.3 Hz, 1H), 8.53 (s, 1H), 10.49 (s, 1H), 11.72 (s, 1H) ppm. ^{13}C-NMR (75 MHz, DMSO-d_6): δ = 14.0, 30.3, 35.0, 59.8, 119.0, 121.0, 121.5, 123.2, 123.4, 123.8, 124.2, 124.4, 128.0, 128.6, 129.0, 130.5, 132.2, 134.0, 138.47, 138.48, 141.0, 149.7, 152.6, 163.9, 167.3, 172.1 ppm.

Ethyl 3-(3-[2-{4-(1H-1,2,3-triazol-1-yl)benzamido}benzamido]phenyl)propanoate (50ai): Preparation according to general procedure B using **47n** and **48u**. Yield: 30%. Yellow solid. ^{1}H-NMR (400 MHz, DMSO-d_6): δ = 1.13 (t, ^3J = 7.1 Hz, 3H), 2.61 (t, ^3J = 7.5 Hz, 2H), 2.85 (t, ^3J = 7.5 Hz, 2H), 4.03 (q, ^3J = 7.1 Hz, 2H), 7.01 (d, ^3J = 7.7 Hz, 1H), 7.24 - 7.35 (m, 2H), 7.53 - 7.60 (m, 2H), 7.61 - 7.67 (m, 1H), 7.93 (dd, ^3J = 7.0 Hz, ^4J = 0.9 Hz, 1H), 8.03 (d, ^4J = 1.1 Hz, 1H), 8.10 - 8.17 (m, 4H), 8.43 (d, ^3J = 8.2 Hz, 1H), 8.94 (d, ^4J = 0.9 Hz, 1H), 10.49 (s, 1H), 11.73 (s, 1H) ppm. ^{13}C-NMR (101 MHz, DMSO-d_6): δ = 14.0, 30.3, 35.0, 59.8, 119.0, 120.2, 121.0, 121.6, 123.4, 123.5, 123.6, 124.2, 128.6, 128.9, 129.0, 132.2, 134.2, 134.7, 138.3, 138.5, 138.9, 141.0, 163.6, 167.3, 172.1 ppm.

Ethyl 3-(3-[4-{tert-butyl}-2-{4-(2-furyl)}benzamido]phenyl)propanoate (50aj): Preparation according to general procedure B using **47h** and **48m**. Yield: 85%. Beige solid. ^{1}H-NMR (300 MHz, $CDCl_3$): δ = 1.20 (s, 9H), 1.25 (t, ^3J = 7.1 Hz, 3H), 2.64 (t, ^3J = 7.8 Hz, 2H), 2.97 (t, ^3J = 7.8 Hz, 2H), 4.15 (q, ^3J = 7.1 Hz, 2H), 6.51 (dd, ^3J = 3.3 Hz, ^4J = 1.8 Hz, 1H), 6.80 (d, ^3J = 3.3 Hz, 1H), 6.92 (dd, ^3J = 8.3 Hz, ^4J = 1.8 Hz, 1H), 7.02 (d, ^3J = 7.6 Hz, 1H), 7.32 (dd, ^3J = 7.8 Hz, 1H), 7.48 - 7.57 (m, 2H), 7.58 - 7.68 (m, 2H), 7.80 (d, ^3J = 8.4 Hz, 2H), 8.03 (d, ^3J = 8.5 Hz, 2H), 8.76 (d, ^4J = 1.7 Hz, 1H), 8.88 (s, 1H), 11.80 (s, 1H) ppm. ^{13}C-NMR (75 MHz, $CDCl_3$): δ = 14.3, 30.9, 31.1, 35.2, 36.0, 60.6, 107.1, 112.1, 118.5, 118.71, 118.74, 120.45, 120.51, 123.9, 124.7, 127.4, 128.1, 129.3, 133.1, 134.1, 138.4, 139.7, 141.8, 143.1, 153.1, 156.8, 165.4, 167.9, 173.0 ppm.

Ethyl 3-(2-[2-naphthamido]benzamido)phenoxyacetate (50ak): Preparation according to general procedure B using **47r** and **48a**. Yield: 65%. Pale yellow solid. ^{1}H-NMR (500 MHz, DMSO-d_6): δ = 1.17 (t, ^{3}J = 7.1 Hz, 3H), 4.14 (q, ^{3}J = 7.1 Hz, 2H), 4.75 (s, 2H), 6.70 (dd, ^{3}J = 8.2 Hz, ^{4}J = 1.9 Hz, 1H), 7.27 (dd, ^{3}J = 8.2, 8.2 Hz, 1H), 7.32 (ddd, ^{3}J = 7.7, 7.7 Hz, ^{4}J = 1.0 Hz, 1H), 7.37 (d, ^{3}J = 8.1 Hz, 1H), 7.42 (dd, ^{4}J = 2.1, 2.1 Hz, 1H), 7.59 - 7.70 (m, 3H), 7.91 (dd, ^{3}J = 7.8 Hz, ^{4}J = 1.2 Hz, 1H), 7.97 (dd, ^{3}J = 8.6 Hz, ^{4}J = 1.8 Hz, 1H), 8.02 (d, ^{3}J = 7.9 Hz, 1H), 8.10 (d, ^{3}J = 8.7 Hz, 2H), 8.45 (d, ^{3}J = 7.8 Hz, 1H), 8.54 (s, 1H), 10.54 (s, 1H), 11.68 (s, 1H) ppm. ^{13}C-NMR (126 MHz, DMSO-d_6): δ = 14.0, 60.6, 64.7, 107.4, 109.9, 113.8, 121.8, 123.5, 123.7, 127.1, 127.7, 127.9, 128.1, 128.6, 129.0, 129.1, 129.5, 131.9, 132.18, 132.21, 134.4, 138.4, 139.8, 157.8, 164.8, 167.4, 168.7 ppm.

Ethyl (3-[2-{2-naphthamido}benzamido]phenyl)glycinate (50al): Preparation according to general procedure C using **47s** and **49a**. Yield: 42%. Beige solid. ^{1}H-NMR (300 MHz, DMSO-d_6): δ = 1.15 (t, ^{3}J = 7.1 Hz, 3H), 3.86 (d, ^{3}J = 6.4 Hz, 2H), 4.08 (q, ^{3}J = 7.1 Hz, 2H), 6.13 (dd, ^{3}J = 6.4, 6.4 Hz, 1H), 6.34 (dd, ^{3}J = 8.0 Hz, ^{4}J = 1.3 Hz, 1H), 6.90 - 7.12 (m, 3H), 7.30 (dd, ^{3}J = 7.1, 7.1 Hz, 1H), 7.57 - 7.71 (m, 3H), 7.92 (d, ^{3}J = 6.8 Hz, 1H), 7.95 - 8.05 (m, 2H), 8.10 (d, ^{3}J = 8.3 Hz, 2H), 8.47 - 8.58 (m, 2H), 10.34 (s, 1H), 11.87 (s, 1H) ppm. ^{13}C-NMR (75 MHz, DMSO-d_6): δ = 14.1, 44.7, 60.2, 105.0, 108.4, 109.6, 121.4, 123.2, 123.3, 123.4, 127.0, 127.7, 127.8, 128.1, 128.6, 128.9, 129.0, 129.1, 131.9, 132.1, 132.2, 134.4, 138.6, 139.2, 148.5, 164.7, 167.2, 171.2 ppm.

Ethyl 2-([3-{2-(2-naphthamido)benzamido}phenyl]amino)-2-methylpropanoate (50am): Preparation according to general procedure C using **47t** and **49a**. Yield: 45%. Brown solid. ^{1}H-NMR (300 MHz, DMSO-d_6): δ = 1.03 (t, ^{3}J = 7.1 Hz, 3H), 1.48 (s, 6H), 4.04 (q, ^{3}J = 7.1 Hz, 2H), 6.00 (s, 1H), 6.28 (dd, ^{3}J = 8.1 Hz, ^{4}J = 1.5 Hz, 1H), 6.86 (d, ^{3}J = 8.0 Hz, 1H), 6.96 - 7.13 (m, 2H), 7.30 (dd, ^{3}J = 7.6, 7.6 Hz, 1H), 7.57 - 7.75 (m, 3H), 7.92 (dd, ^{3}J = 7.9 Hz, ^{4}J = 1.3 Hz, 1H), 7.96 - 8.06 (m, 2H), 8.06 - 8.19 (m, 2H), 8.50 - 8.61 (m, 2H), 10.35 (s, 1H), 11.94 (s, 1H) ppm. ^{13}C-NMR (75 MHz, DMSO-d_6): δ = 13.9, 25.9, 56.3, 60.4, 106.2, 109.3, 110.2, 121.3, 122.9, 123.25, 123.34, 127.0, 127.7, 127.8, 128.1, 128.56, 128.61, 129.0, 129.1, 131.8, 132.1, 132.2, 134.4, 138.7, 138.9, 146.8, 164.6, 167.2, 175.6 ppm.

Ethyl (3-[2-{4-(3-furyl)benzamido}-4-methoxybenzamido]phenyl)glycinate (50an): Preparation according to general procedure B using **47u** and **48n**. Yield: 89%. Beige solid. ^{1}H-NMR (300 MHz, DMSO-d_6): δ = 1.18 (t, ^{3}J = 7.1 Hz, 3H), 3.85 - 3.91 (m, 5H), 4.11 (q, ^{3}J = 7.1 Hz, 2H), 6.13 (t, ^{3}J = 6.4 Hz, 1H), 6.34 (dd, ^{3}J = 8.0 Hz, ^{4}J = 1.0 Hz, 1H), 6.83 (dd, ^{3}J = 8.9 Hz, ^{4}J = 2.6 Hz, 1H), 6.90 - 6.99 (m, 2H), 7.02 - 7.12 (m, 2H), 7.79 (dd, ^{4}J = 1.7, 1.7 Hz, 1H), 7.84 (d, ^{3}J = 8.4 Hz, 2H), 7.90 - 8.01 (m, 3H), 8.33 (s, 1H), 8.36 (d, ^{4}J = 2.6 Hz, 1H), 10.19 (s, 1H), 12.50 (s, 1H) ppm. ^{13}C-NMR (75 MHz, DMSO-d_6): δ = 14.1, 44.7, 55.5, 60.2, 105.4, 105.6, 108.3, 108.4, 108.6, 110.1, 113.3, 125.0, 125.9, 127.5, 128.9, 130.7, 132.6, 135.7, 139.0, 140.6, 141.5, 144.7, 148.4, 162.3, 164.2, 167.5, 171.2 ppm.

Ethyl 3-nitrophenoxyacetate (55): Preparation according to general procedure I using **53** and **54a**. Yield: 98%. Brown oil. ^{1}H-NMR (300 MHz, $CDCl_3$): δ = 1.31 (t, ^{3}J = 7.1 Hz, 3H), 4.29 (q, ^{3}J = 7.1 Hz, 2H), 4.70 (s, 2H), 7.26 (ddd, ^{3}J = 8.3 Hz, ^{4}J = 2.6, 0.9 Hz, 1H), 7.46 (dd, ^{3}J = 8.2, 8.2 Hz, 1H), 7.72 (dd, ^{4}J = 2.3, 2.3 Hz, 1H), 7.87 (ddd, ^{3}J = 8.1 Hz, ^{4}J = 2.1, 0.9 Hz, 1H) ppm. ^{13}C-NMR (75 MHz, $CDCl_3$): δ = 14.3, 61.9, 65.7, 109.2, 116.9, 121.9, 130.3, 149.3, 158.5, 168.1 ppm.

2-Nitro-4-(trifluoromethoxy)benzonitrile (58): Preparation according to general procedure J using **57**. Yield: 92%. Yellow liquid. ^{1}H-NMR (400 MHz, $CDCl_3$) δ = 7.66 (d, ^{3}J = 8.5 Hz, 1H), 8.00 (d, ^{3}J = 8.6 Hz, 1H), 8.18 (s, 1H). ^{13}C-NMR (75 MHz, $CDCl_3$) δ = 106.3, 114.0, 117.9, 120.2, 125.6, 137.3, 152.3 ppm.

4-(tert-Butyl)-1-methyl-2-nitrobenzene (60): Preparation according to general procedure L using **59**. Yield: 63%. Yellow liquid. ^{1}H-NMR (500 MHz, $CDCl_3$): δ = 1.34 (s, 9H), 2.56 (s, 3H), 7.26 (d, ^{3}J = 8.0 Hz, 1H), 7.52 (dd, ^{3}J = 8.0 Hz, ^{4}J = 2.0 Hz, 1H), 7.97 (d, ^{4}J = 2.0 Hz, 1H) ppm. ^{13}C-NMR (126 MHz, $CDCl_3$): δ = 20.1, 31.2, 34.8, 121.6, 130.4, 130.7, 132.6, 149.2, 150.8 ppm.

N-Hydroxy-p-toluenesulfonamide (65): Preparation according to general procedure V using **64**. Yield: 42%. White solid. ^{1}H-NMR (300 MHz, DMSO-d_6): δ = 2.42 (s, 3H), 7.44 (dd, ^{3}J = 8.6 Hz, ^{4}J = 0.6 Hz, 2H), 7.70 - 7.78 (m, 2H), 9.49 (d, ^{4}J = 3.4 Hz, 1H), 9.55 (d, ^{4}J = 3.3 Hz, 1H) ppm. ^{13}C-NMR (75 MHz, DMSO-d_6): δ = 21.0, 128.1, 129.4, 134.5, 143.5 ppm.

4-([2E]-Propenal-3-yl)benzoic acid (67): Preparation according to general procedure W using **62** and **66**. Yield: 24%. Yellow solid. ^{1}H-NMR (300 MHz, DMSO-d_6): δ = 6.95 (dd, ^{3}J = 16.0, 7.7 Hz, 1H), 7.74 - 7.92 (m, 3H), 7.95 - 8.05 (m, 2H), 9.72 (d, ^{3}J = 7.7 Hz, 1H), 13.09 (br s, 1H) ppm. ^{13}C-NMR (75 MHz, DMSO-d_6): δ = 128.8, 129.8, 130.3, 132.6, 138.0, 151.5, 166.7, 194.5 ppm.

Methyl 4-(bromoacetyl)benzoate (69): Preparation according to general procedure Y using **68**. Yield: 90%. Yellow solid. ^{1}H-NMR (300 MHz, DMSO-d_6): δ = 3.89 (s, 3H), 4.39 (s, 2H), 7.94 - 8.01 (m, 2H), 8.06 - 8.13 (m, 2H) ppm. ^{13}C-NMR (75 MHz, DMSO-d_6): δ = 30.8, 52.7, 129.0, 130.2, 134.8, 137.3, 166.1, 191.0 ppm.

Methyl 4-(oxazol-4-yl)benzoate (70): Preparation according to general procedure Z using **69**. Yield: 27%. Yellow solid. ^{1}H-NMR (300 MHz, $CDCl_3$): δ = 3.93 (s, 3H), 7.85 - 7.80 (m, 2H), 7.97 (d, ^{4}J = 0.9 Hz, 1H), 8.04 (d, ^{4}J = 0.9 Hz, 1H), 8.06 - 8.12 (m, 2H) ppm. ^{13}C-NMR (75 MHz, $CDCl_3$): δ = 52.3, 125.6, 129.8, 130.3, 135.1, 135.2, 139.8, 151.7, 166.9 ppm.

2-Hydroxy-6-methyl-benzaldehyde (72b): Preparation according to general procedure R with using **75**. Yield: 69%. Yellow oil. ^{1}H-NMR (400 MHz, $CDCl_3$): δ = 2.61 (s, 3H), 6.71 (d, ^{3}J = 7.4 Hz, 1H), 6.81 (d, ^{3}J = 8.5 Hz, 1H), 7.37 (dd, ^{3}J = 7.9, 7.9 Hz, 1H), 10.32 (s, 1H), 11.90 (s, 1H) ppm. ^{13}C-NMR (75 MHz, $CDCl_3$): 18.2, 116.3, 118.7, 122.0, 137.6, 142.3, 163.4, 195.5 ppm.

5-Methyl-2H-chromene-3-carbonitrile (73b): Preparation according to general procedure O using **71** and **72b**. Yield: 42%. Yellow solid. ^{1}H-NMR (400 MHz, $CDCl_3$): δ = 2.34 (s, 3H), 4.75 (s, 2H), 6.73 (d, ^{3}J = 8.2 Hz, 1H), 6.81 (d, ^{3}J = 7.6 Hz, 1H), 7.16 (dd, ^{3}J = 7.9, 7.9 Hz, 1H), 7.38 (s, 1H) ppm. ^{13}C-NMR (75 MHz, $CDCl_3$): 18.5, 63.8, 102.9, 114.5, 116.9, 119.3, 124.1, 132.3, 136.4, 136.6, 154.8 ppm.

6-Methyl-2H-chromene-3-carbonitrile (73c): Preparation according to general procedure O using **71** and **72c**. Yield: 72%. Pale yellow solid. ^{1}H-NMR (400 MHz, $CDCl_3$): δ = 2.27 (s, 3H), 4.77 (d, ^{4}J = 1.3 Hz, 2H), 6.77 (d, ^{3}J = 8.3 Hz, 1H), 6.90 (d, ^{4}J = 1.6 Hz, 1H), 7.07 (dd, ^{3}J = 8.3 Hz, ^{4}J = 1.6 Hz, 1H), 7.13 (s, 1H) ppm. ^{13}C-NMR (75 MHz, $CDCl_3$): 20.5, 64.5, 103.4, 116.4, 116.7, 120.0, 128.8, 132.0, 133.5, 139.1, 152.3 ppm.

6-Fluoro-2H-chromene-3-carbonitrile (73d): Preparation according to general procedure O using **71** and **72d**. Yield: 76%. Pale yellow solid. ^{1}H-NMR (400 MHz, DMSO-d_6): δ = 4.88 (d, ^{4}J = 1.3 Hz, 2H), 6.89 - 6.97 (m, 1H), 7.12 - 7.21 (m, 2H), 7.54 (s, 1H) ppm. ^{13}C-NMR (75 MHz, DMSO-d_6): δ = 63.8, 105.0, 114.5, 116.4, 117.7, 119.0, 121.2, 138.1, 150.2, 156.9 ppm.

6-Chloro-2H-chromene-3-carbonitrile (73e): Preparation according to general procedure O using **71** and **72e**. Yield: 61%. Pale yellow solid. ^{1}H-NMR (400 MHz, DMSO-d_6): δ = 4.91 (d, ^{4}J = 1.4 Hz, 2H), 6.92 (d, ^{3}J = 8.6 Hz, 1H), 7.34 (dd, ^{3}J = 8.6 Hz, ^{4}J = 2.6 Hz, 1H), 7.37 (d, ^{4}J = 2.6 Hz, 1H), 7.53 (s, 1H) ppm. ^{13}C-NMR (75 MHz, DMSO-d_6): δ = 63.8, 104.7, 116.3, 118.0, 121.5, 125.8, 127.8, 131.9, 137.7, 152.6 ppm.

7-Methyl-2H-chromene-3-carbonitrile (73f): Preparation according to general procedure O using **71** and **72f**. Yield: 73%. Pale yellow solid. ^{1}H-NMR (400 MHz, DMSO-d_6): δ = 2.26 (s, 3H), 4.83 (d, ^{4}J = 1.3 Hz, 2H), 6.72 (s, 1H), 6.82 (dd, ^{3}J = 7.7 Hz, ^{4}J = 0.8 Hz, 1H), 7.15 (d, ^{3}J = 7.7 Hz, 1H), 7.52 (s, 1H) ppm. ^{13}C-NMR (126 MHz, DMSO-d_6): δ = 21.3, 63.6, 101.8, 116.5, 116.9, 117.7, 123.1, 128.6, 139.0, 143.3, 153.9 ppm.

8-Methyl-2H-chromene-3-carbonitrile (73g): Preparation according to general procedure O using **71** and **72g**. Yield: 83%. Pale yellow solid. ^{1}H-NMR (400 MHz, $CDCl_3$): δ = 2.19 (s, 3H), 4.83 (d, ^{4}J = 1.3 Hz, 2H), 6.87 (dd, ^{3}J = 7.5, 7.5 Hz, 1H), 6.95 (dd, ^{3}J = 7.5 Hz, ^{4}J = 1.3 Hz, 1H), 7.14 (dd, ^{3}J = 7.4 Hz, ^{4}J = 0.8 Hz, 1H), 7.16 (dd, ^{4}J = 1.1, 1.1 Hz, 1H) ppm. ^{13}C-NMR (75 MHz, $CDCl_3$): 15.5, 64.5, 103.0, 116.7, 119.7, 121.9, 126.2, 126.3, 134.4, 139.4, 152.5 ppm.

8-Chloro-2H-chromene-3-carbonitrile (73h): Preparation according to general procedure O using **71** and **72h**. Yield: 35%. White solid. ^{1}H-NMR (400 MHz, DMSO-d_6): δ = 5.02 (d, ^{4}J = 1.4 Hz, 2H), 7.02 (dd, ^{3}J = 7.8, 7.8 Hz, 1H), 7.25 (dd, ^{3}J = 7.6 Hz, ^{4}J = 1.4 Hz, 1H), 7.45 (dd, ^{3}J = 8.1 Hz, ^{4}J = 1.4 Hz, 1H), 7.62 (s, 1H) ppm. ^{13}C-NMR (75 MHz, DMSO-d_6): 64.3, 104.4, 116.3, 120.2, 121.6, 122.9, 127.6, 132.7, 138.2, 149.4 ppm.

8-Methoxy-2H-chromene-3-carbonitrile (73i): Preparation according to general procedure O using **71** and **72i**. Yield: 43%. Pale yellow solid. ^{1}H-NMR (400 MHz, DMSO-d_6): δ = 3.77 (s, 3H), 4.85 (d, ^{4}J = 1.3 Hz, 2H), 6.87 (dd, ^{3}J = 7.6 Hz, ^{4}J = 1.3 Hz, 1H), 6.95 (dd, ^{3}J = 7.9, 7.9 Hz, 1H), 7.07 (dd, ^{3}J = 8.2 Hz, ^{4}J = 1.3 Hz, 1H), 7.57 (s, 1H) ppm. ^{13}C-NMR (126 MHz, DMSO-d_6): δ = 55.8, 63.5, 103.2, 115.9, 116.7, 120.3, 120.8, 122.1, 139.2, 143.0, 147.7 ppm.

2-Methoxy-6-methyl-benzaldehyde (75): Preparation according to general procedure Q using **74**. Yield: 38%. Yellow oil. ^{1}H-NMR (400 MHz, $CDCl_3$): δ = 2.57 (s, 3H), 3.90 (s, 3H), 6.76 – 6.88 (m, 2H), 7.38 (dd, ^{3}J = 8.0, 8.0 Hz, 1H), 10.65 (s, 1H) ppm. ^{13}C-NMR (75 MHz, $CDCl_3$): 21.6, 55.9, 109.2, 124.2, 126.7, 134.6, 142.2, 163.3, 192.4 ppm.

4-Chloro-2H-chromene-3-carboxaldehyde (77): Preparation according to general procedure S using **76**. Yield: 97%. Yellow solid. ^{1}H-NMR (300 MHz, $CDCl_3$): δ = 5.00 (s, 2H), 6.90 (ddd, ^{3}J = 8.2 Hz, ^{4}J = 1.1 Hz, ^{5}J = 0.3 Hz, 1H), 7.05 (ddd, ^{3}J = 7.8, 7.4 Hz, ^{4}J = 1.1 Hz, 1H), 7.37 (ddd, ^{3}J = 8.2, 7.4 Hz, ^{4}J = 1.6 Hz, 1H), 7.69 (dd, ^{3}J = 7.9 Hz, ^{4}J = 1.6 Hz, 1H), 10.16 (s, 1H) ppm. ^{13}C-NMR (75 MHz, $CDCl_3$): δ = 64.5, 116.8, 120.7, 122.3, 125.0, 126.7, 134.2, 143.4, 156.8, 187.9 ppm.

Supplementary Methods for in Vitro Characterization

Multiplex toxicity assay

Cytotoxicity was assessed in a high content screen on two different cell lines, HEK293T (ATCC® CRL-1573™) and U2OS (ATCC®HTB-96™), essentially as described previously[6]. In brief, cells were cultured in DMEM plus L-Glutamine (high glucose) supplemented with 10 % FBS (Gibco) and Penicillin/Streptomycin (Gibco). For both cell lines 750 cells were seeded in 384 well plates in culturing medium (Cell culture microplate, PS, f-bottom, µClear®, 781091, Greiner). Cells were stained prior to compound treatment with 0.3 µL/well Annexin V Alexa488 (Invitrogen), 1 µM Yo-Pro3 (Invitrogen) and 75 nM Hoechst33342 (Thermo Scientific). Each compound (OCA, **41**, elafibranor) was tested at seven different concentrations (0.1 µM, 0.3 µM, 1 µM, 3 µM, 10 µM, 30 µM, 100 µM) in triplicates in two independent biological repeats.

After 2 h, 12 h and 24 h, cellular shape and fluorescence was analyzed on an automated, high-content confocal microscope (CQ1, Yokogawa). Images were acquired with the following setup parameters: Ex 405 nm/Em 447/60 nm, 500ms, 50%; Ex 561 nm/Em 617/73 nm, 100 ms, 40%; Ex 488/Em 525/50 nm, 50 ms, 40%; bright field, 300ms, 100% transmission, one centered field per well, 7 z stacks per well with 55 µm spacing. Images were analyzed with the Pathfinder software (Yokogawa). Cells were detected and gated as previously described[6]. Data of tested compounds was normalized against the average of DMSO (0.1%) wells.

Microsomal stability assay

The solubilized test compound (5 µL, final concentration 10 µM in phosphate buffer (0.1 M, pH 7.4)) was preincubated at 37 °C in 432 µL of phosphate buffer (0.1 M, pH 7.4) together with a 50 µL NADPH regenerating system (30 mM glucose-6-phosphate, 4 U/mL glucose-6-phosphate dehydrogenase, 10 mM NADP, 30 mM $MgCl_2$). After 5 min, the reaction was started by the addition of 13 µL of microsome mix from the liver of Sprague−Dawley rats (Invitrogen; 20 mg protein/mL in 0.1 M phosphate buffer) in a shaking water bath at 37 °C. The reaction was stopped by addition of 250 µL of ice-cold methanol at 0, 15, 30 and 60 min. The samples were diluted with 250 µL of DMSO and centrifuged at 10000 g for 5 min at 4 °C. The supernatants were analyzed and test compound was quantified by HPLC: mobile phase: MeOH 83%/H_2O 17%/formic acid 0.1%; flow-rate: 1 mL/min; stationary phase: MultoHigh Phenyl phase, 5 µm, 250×4, precolumn, phenyl, 5 µm, 20×4; detection wavelength: 330 and 254 nm; injection volume: 50 µL. Control samples were performed to check the test compound's stability in the reaction mixture: first control was without NADPH, which is needed for the enzymatic activity of the microsomes, second control was with inactivated microsomes (incubated for 20 min at 90 °C), third control was without test compound (to determine the baseline). The amounts of the test compound were quantified by an external calibration curve, where data are expressed as mean ± SEM of single determinations obtained in three independent experiments. The metabolism experiment showed the following results (expressed as mean percent of remaining compound ± SEM; n = 3): **41**: 0 min: 100%, 15 min: 95 ± 3%, 30 min: 95 ± 2%, 60 min: 93 ± 2%; 7-EC (n = 1): 0 min: 100%, 15 min: 87% , 30 min: 75%, 60 min: 55%.

Molecular Docking

Docking was performed using AutoDock Vina[4] with default preferences unless stated otherwise. X-ray structures of the FXR (PDB code 4QE8[1]), PPARα (PDB code 2P54[2]), and PPARδ (PDB code 5U3R[3]) ligand binding domains were chosen based on similarity of the co-crystallized ligand to **41**. A 3D structure of **41** was generated using Chem3D 19.1 and saved as a pdb file. For the preparation of the receptors and the ligand with AutoDockTools 1.5.7, the co-crystallized ligands and water molecules were removed from the structures, polar hydrogens were added, and Gasteiger charges computed for the protein. Torsional root and the rotatable bonds of **41** were identified and default settings used. The grid box was placed manually at the center of the ligand binding pocket based on the position of the co-crystallized ligands. AutoDock Vina was finally employed setting the exhaustiveness of the search to 16. The top-ranked binding mode with the carboxylate forming neutralizing contacts was used and visualized using PyMOL.[7]

Supplementary References

1. Merk, D. *et al.* Molecular tuning of farnesoid X receptor partial agonism. *Nat. Commun.* **10**, 2915 (2019).
2. Sierra, M. L. *et al.* Substituted 2-[(4-aminomethyl)phenoxy]-2-methylpropionic acid PPARα agonists. 1. Discovery of a novel series of potent HDLc raising agents. *J. Med. Chem.* **50**, 685–695 (2007).
3. Wu, C.-C. *et al.* Structural basis for specific ligation of the peroxisome proliferator-activated receptor δ. *Proc. Natl. Acad. Sci.* **114**, E2563–E2570 (2017).
4. Trott, O. & Olson, A. J. AutoDock Vina: Improving the speed and accuracy of docking with a new scoring function, efficient optimization, and multithreading. *J. Comput. Chem.* **31**, NA-NA (2009).
5. Merk, D. *et al.* Anthranilic acid derivatives as nuclear receptor modulators - Development of novel PPAR selective and dual PPAR/FXR ligands. *Bioorg. Med. Chem.* **23**, 499–514 (2015).
6. Howarth, A. *et al.* HighVia—A Flexible Live-Cell High-Content Screening Pipeline to Assess Cellular Toxicity. *SLAS Discov.* **25**, 801–811 (2020).
7. Schrodinger LLC. The PyMOL Molecular Graphics System, Version 2.0. (2015).
