# Supplementary material for: A triple farnesoid X receptor and peroxisome proliferator-activated receptor α/δ activator reverses hepatic fibrosis in diet-induced NASH in mice
Source: Commun Chem. 2020 Nov 13;3:174. doi: 10.1038/s42004-020-00411-z (PMC9814779; doi:10.1038/s42004-020-00411-z)
Supplement: Supplementary file 2 — Reporting Summary [file 42004_2020_411_MOESM2_ESM.pdf]

nature research

Corresponding author(s): Daniel Merk

Last updated by author(s): Oct 10, 2020

Reporting Summary

Nature Research wishes to improve the reproducibility of the work that we publish. This form provides structure for consistency and transparency in reporting. For further information on Nature Research policies, see our Editorial Policies and the Editorial Policy Checklist.

Statistics

For all statistical analyses, confirm that the following items are present in the figure legend, table legend, main text, or Methods section.

n/a	Confirmed	
☐	☒	The exact sample size (*n*) for each experimental group/condition, given as a discrete number and unit of measurement
☐	☒	A statement on whether measurements were taken from distinct samples or whether the same sample was measured repeatedly
☐	☒	The statistical test(s) used AND whether they are one- or two-sided<br>*Only common tests should be described solely by name; describe more complex techniques in the Methods section.*
☐	☒	A description of all covariates tested
☒	☐	A description of any assumptions or corrections, such as tests of normality and adjustment for multiple comparisons
☐	☒	A full description of the statistical parameters including central tendency (e.g. means) or other basic estimates (e.g. regression coefficient) AND variation (e.g. standard deviation) or associated estimates of uncertainty (e.g. confidence intervals)
☐	☒	For null hypothesis testing, the test statistic (e.g. *F*, *t*, *r*) with confidence intervals, effect sizes, degrees of freedom and *P* value noted<br>*Give P values as exact values whenever suitable.*
☒	☐	For Bayesian analysis, information on the choice of priors and Markov chain Monte Carlo settings
☒	☐	For hierarchical and complex designs, identification of the appropriate level for tests and full reporting of outcomes
☒	☐	Estimates of effect sizes (e.g. Cohen's *d*, Pearson's *r*), indicating how they were calculated

Our web collection on statistics for biologists contains articles on many of the points above.

Software and code

Policy information about availability of computer code

Data collection	No software was used.
Data analysis	MS Excel and GraphPad Prism 7 were used for data analysis and statistical tests.

For manuscripts utilizing custom algorithms or software that are central to the research but not yet described in published literature, software must be made available to editors and reviewers. We strongly encourage code deposition in a community repository (e.g. GitHub). See the Nature Research guidelines for submitting code & software for further information.

Data

Policy information about availability of data

All manuscripts must include a data availability statement. This statement should provide the following information, where applicable:

- Accession codes, unique identifiers, or web links for publicly available datasets
- A list of figures that have associated raw data
- A description of any restrictions on data availability

The datasets generated and analyzed during the current study are available from the corresponding author on reasonable request.

Field-specific reporting

Life sciences study design

All studies must disclose on these points even when the disclosure is negative.

Sample size	All in vitro experiments were performed in at least three biologically independent repeats with at least technical duplicates. Sample size in the DIO NASH model was defined based on a power analysis which was performed before the study.
Data exclusions	No data were excluded.
Replication	All experiments have been repeated in biologically independent replicates as decribed in the manuscript and Supplementary Information.
Randomization	Animals in the DIO NASH study were randomized after NASH was confirmed by pre-biopsy to receive either treatment or vehicle.
Blinding	Investigators were blinded to group allocation during analysis of samples from the animal studies.

Reporting for specific materials, systems and methods

We require information from authors about some types of materials, experimental systems and methods used in many studies. Here, indicate whether each material, system or method listed is relevant to your study. If you are not sure if a list item applies to your research, read the appropriate section before selecting a response.

Materials & experimental systems

n/a	Involved in the study	
☒	☐	Antibodies
☐	☒	Eukaryotic cell lines
☒	☐	Palaeontology and archaeology
☐	☒	Animals and other organisms
☒	☐	Human research participants
☒	☐	Clinical data
☒	☐	Dual use research of concern

Methods

n/a	Involved in the study	
☒	☐	ChIP-seq
☒	☐	Flow cytometry
☒	☐	MRI-based neuroimaging

Eukaryotic cell lines

Policy information about cell lines

Cell line source(s)	Cell lines were obtained from the German Collection of Micoorganisms and Cell Cultures (DSMZ) or the American Type Culture Collection (ATCC)
Authentication	Cell lines were not further authenticated.
Mycoplasma contamination	Cell lines were tested negative for mycoplasma contamination.
Commonly misidentified lines (See ICLAC register)	n/a

Animals and other organisms

Policy information about studies involving animals; ARRIVE guidelines recommended for reporting animal research

Laboratory animals	Male RjOrl:Swiss (CD 1) mice (purchased from Janvier Labs, France) were used for the pilot animal study. Male C57BL/6JRj mice (purchased from Janvier Labs, France) were used for the DIO NASH study.
Wild animals	No wild animals were involved in this study.
Field-collected samples	The study did not involve field-collected samples.
Ethics oversight	All studies involving animals were cfully compliant to internationally accepted principles for the care and use of laboratory animals and approved by ethics authorities as described in the manuscript and Supplementary Information.

Note that full information on the approval of the study protocol must also be provided in the manuscript.
